# Gestational exposure to chlordecone promotes transgenerational changes in the murine reproductive system of males

**DOI:** 10.1038/s41598-018-28670-w

**Published:** 2018-07-06

**Authors:** Aurore Gely-Pernot, Chunxiang Hao, Louis Legoff, Luc Multigner, Shereen Cynthia D’Cruz, Christine Kervarrec, Bernard Jégou, Sergei Tevosian, Fatima Smagulova

**Affiliations:** 1 University Rennes, EHESP, Inserm, Irset (Institut de recherche en santé, environnement et travail) - UMR_S 1085, F-35000 Rennes, France; 2 College of Pharmacy, Linyi University, 276000 Linyi, China; 3 University of Florida, Department of Physiological Sciences Box 100144, 1333 Center Drive, 32610 Gainesville, FL USA

## Abstract

Environmental factors can affect epigenetic events during germline reprogramming and impose distinctive transgenerational consequences onto the offspring. In this study, we examined the transgenerational effects of chlordecone (CD), an organochlorine insecticide with well-known estrogenic properties. We exposed pregnant mice to CD from embryonic day 6.5 to 15.5 and observed a reduction in spermatogonia (SG) numbers in F3, meiotic defects in spermatocytes and decrease in spermatozoa number in the first and third generation of male progeny. The RNA qRT-PCR expression analysis in F1 and transcriptomics analysis in F3 males using the whole testes revealed changes in the expression of genes associated with chromosome segregation, cell division and DNA repair. The expression of the master regulator of pluripotency, *Pou5f1*, decreased in foetal and increased in adult F1, but not in F3 adult testes. Analysis of histone H3K4me3 distribution revealed widespread changes in its occupancy in the genome of F1 and F3 generations. We established that 7.1% of altered epigenetic marks were conserved between F1 and F3 generations. The overlapping changes common to F1 and F3 include genes implicated in cell adhesion and transcription factor activities functions. Differential peaks observed in F1 males are significantly enriched in predicted ESR1 binding sites, some of which we confirmed to be functional. Our data demonstrate that CD-mediated impairment of reproductive functions could be transmitted to subsequent generations.

## Introduction

During development, germ cells undergo comprehensive global somatic-to-germline reprogramming. Epigenetic modifications, such as DNA methylation and histone modifications are involved in this process. The H3K4me3 and H3K27me3 histone marks are postulated to be key epigenetic modifications that are located at the promoters of pluripotent genes in embryonic stem (ES) cells^1^. Despite their common occurrence in the regulatory regions, it was determined that the mere presence of H3K4me3 does not indicate or predict transcriptional activity^1–3^. Instead, it was hypothesized that enrichment for histone marks could serve to define cell identity, with subsets of differentially marked genes kept in a poised state in the germline during foetal development throughout post-meiotic stage^4^. In this case, H3K4me3 marks positioned at developmentally relevant genes are thought to be essential for germ cell identity, to define the developmental window where transcription factors are recruited^4^.

Importantly, while the bulk of histones are replaced by protamine in sperm, 1% in mice and up to 10% of histones in humans are retained in genomic DNA^5^. It is now widely accepted that remodelling of chromatin architecture plays an important role in transgenerational inheritance (reviewed in^6^). Moreover, ectopic overexpression of the human histone lysine demethylase, KDM1a, during mouse spermatogenesis resulted in the transgenerational inheritance of abnormalities in the limbs, skeleton and skin development. This data supports the idea that histone methylation could be a mediator of transgenerational effects^7^. The whole-genome datasets cataloguing human and bovine sperm nucleosome-retaining fractions provide evidence that genes encoding proteins with signal transduction and mitochondrial functions are enriched in the histone-containing parts of the genome^8^. The enrichment of the same set of genes in both human and bovine genomes suggests a conserved mechanism for histone retention^8^. Subsequent work confirmed histone inheritance in sperm and is in agreement with the results of previous studies (e.g.^9^). Until recently, the ultimate fate of paternal histones in zygotes was unclear. The ChIP STAR protocol, which allows the use of a very limited amount of nuclear material, was used to explore the fate and dynamics of paternal histones in early embryos^9,10^. Importantly, a low but detectable level of paternal H3K4me3 was present in early embryos, with histone peaks organized in large domains. It was postulated that these paternal H3K4me3 histones existed during a poised state and that the genes corresponding to these marks were waiting to be activated^10^. In line with this prediction, during the early embryonic stage, bivalently marked histones are absent at the regulatory regions governing developmental genes and subsequently become re-established in post-implantation embryos^9^.

Skinner and colleagues demonstrated that several toxic compounds could promote epigenetic changes that are detected in subsequent generations^11–13^. In our recent study, we showed that embryonic exposure to the herbicide atrazine (ATZ) causes global changes in gene expression in several tissues in the third (F3) generation, with the testis harbouring the largest number of differentially expressed transcripts^14^. We also found that altered RNA expression in the testes is associated with global changes in histone H3K4me3 localization, providing further support for the idea that transgenerational effects can be mediated by the retention of histones in a small fraction of the genome^14^. Differential epigenetic marks were found at the *Pou5f1, Phf2, Ezh2* and *Zhx2* genes, which are essential for establishing the pluripotent state of germ cells^14^. These data suggest a possible role for epigenetic modifications in the transmission of ATZ effects to subsequent generations. Epigenetic mechanisms are presumed to include other heritable alterations including noncoding RNA and DNA methylation^15^. There is evidence that all of these mechanisms can be used to transmit environmentally-induced modifications across several generations via gamete inheritance (reviewed in^16^).

In this study, we examined whether the transgenerational effects could be promoted by the insecticide CD, also known as Kepone. CD was extensively used in the USA, Latin America, Africa and Eastern Europe until 1975, and, until 1993, in the French West Indies. Because CD does not undergo biotic or abiotic degradation in the environment, permanently polluted soil and waters are the primary source of food contamination, and humans remain exposed to this chemical in the French West Indies. CD causes hepatic tumours in laboratory rats and mice^17–19^ and has neurotoxic effects in rats^20^. Furthermore, CD is capable of binding with oestrogen receptors ESR1 and ESR2, eliciting agonistic and antagonistic effects respectively^21,22^. Activation of ESR1 mediates adverse effects of oestrogen, such as aberrant proliferation, inflammation, and malignancy; whereas ESR2 is thought to exert the opposite effects, such as anti-proliferative, anti-inflammatory, and potentially anti-carcinogenic activities^23^. Hence, CD’s engagement of ESRs can trigger aberrant responses by engaging oestrogen signalling cascades. In humans, occupational exposure to CD at high doses causes neurological problems and decreases sperm cell production and motility^24,25^. Epidemiological studies have suggested that continuous exposure to CD at environmental levels in adults could also increase the risk of prostate cancer^26^.

Of particular concern is the ability of CD to cross the placenta and affect the embryo, as many developmental processes are vulnerable to its effects. Gestational exposure to CD affects prenatal and postnatal development in rats and mice (reviewed in^27^). Recent data from the TIMOUN Mother-Child Cohort Study conducted in French West Indies show that prenatal exposure to CD at environmental levels is associated with a decreased length of gestation^28^ and may affect foetal and infant growth^29^ as well as neurodevelopment during infancy^30,31^.

Because CD is persistent in nature, and human populations are still exposed to the compound, it is important to evaluate its ability to affect transgenerational inheritance. We hypothesized that developmental exposure to a low dose of CD could affect germ cells during the reprogramming window and that these changes could be transmitted to subsequent generations. During reprograming window germ cells are known to undergo extensive epigenetic modification of their genome and chromatin and to acquire the pluripotent state. In mice, this process occurs between embryonic days (E) E5.5 and E15, and is initiated by the expression of transcription factor BLIMP1/PRDM1 (for review, see^32^. PRDM1 represses the somatic program and promotes the progression towards germ cell differentiation^33^.

Here, we show that gestational exposure to CD decreases SG cell numbers, affects meiosis, leads to changes in RNA expression and alters H3K4me3 occupancy at many genes in the first and third generation of male mice. We found that at least some modified histone peaks were conserved between F1 and F3 generations, corresponding to 76 genes with the overlapping peak regions. Among these genes, ten are predicted targets of ZFP57, the transcriptional repressor. In F1 generation, developmental genes with regions of altered H3K4me3 occupancy are also significantly enriched in ESR1 binding sites (~290 sequences) within their promoters. Exposure to CD leads to altered binding capacity of ESR1 in embryonic testis of these mice, implicating the endocrine disrupting action of CD in gonads. Additionally, we found that H3K27me3 and H4K5Ac marks have altered occupancies in F3 generation at least at some common differential H3K4me3 peaks.

Our data suggest that embryonic exposure to CD affects germ cells in mice and that the changes induced by CD are preserved for up to three generations. Our observations may be informative to better assess the risk associated with CD exposure, particularly during pregnancy, and to improve public health policies.

## Results

### Research design

To analyse the transgenerational effect of CD, we follow the conventional protocol (e.g.^16^) where F0 mice are CD-exposed pregnant dams, F1 population males is directly exposed progeny from F0, F2 males are derived from the gametes of CD-exposed germ cells of F1, and the F3 generation is the first not directly exposed males (Supplementary Fig. 1).

In this study, we used an oral dose of 100 μg/kg bw/day of CD, a dose that has no effect on murine health^34^.We treated pregnant female mice from E6.5 to E15.5 with CD. We conducted the treatment during a reprograming window of E6.5 to E15.5, which is essential for normal development of germ cells^35,36^. According to previous studies, germ cells are particularly vulnerable to environmental assault (e.g., by endocrine disruptors such as vinclozolin or ATZ) during this stage^37,38^. We assessed the effects of CD on germ cell numbers, RNA expression and ESR1 binding in E15.5 foetuses. The progeny of the CD-treated mice (F1 and F3 adults) were analysed. Specifically, we studied the effects of CD on germ cell number, meiotic synapsing and DNA repair processes, spermatozoa number, RNA expression and H3K4me3 histone distribution.

### Gestational exposure to CD affects gene expression in foetal germ cells

**Figure 1:**
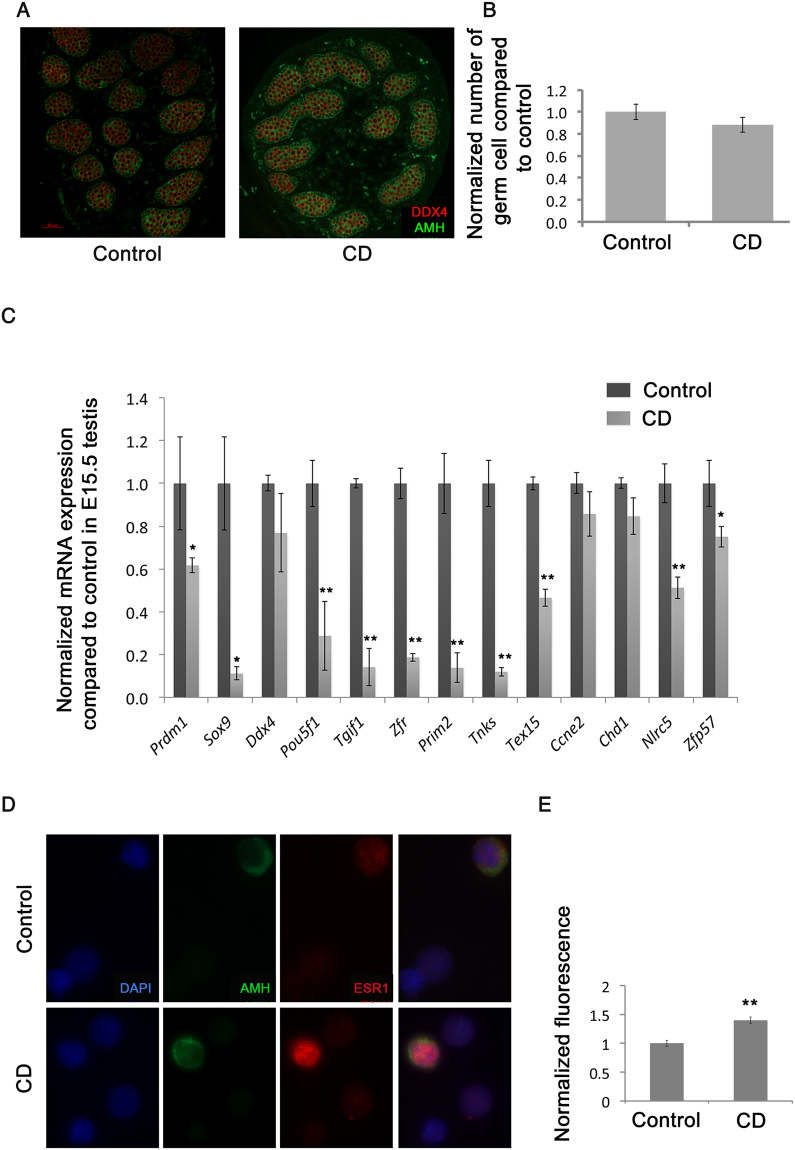
Gestational CD exposure affects F1 embryonic germ cells. (**A**) Representative images of testes sections from the control (left panel) and CD lineage (right panel) animals. Sertoli and prospermatogonial germ cells were immunostained using anti-Anti Müllerian Hormone (AMH, green) or anti-DDX4 (red) antibodies, respectively, scale bar 50 µm. (**B**) A quantitative analysis of germ cells was performed by manually counting the cells in the seminiferous tubules in E15.5 mouse testes. The contour of each tubule section was measured using Adobe Photoshop. The values indicate the germ cell numbers compared to control. The bar indicates the mean value +/−SEM, (p = 0.176, n = 4 control, n = 5 CD samples, t-test). (**C**) The RT–qPCR analysis in F1 E15.5 testis. RNA was extracted and reverse transcribed as described in material and methods, qPCR was performed using primers indicated in SI. The gene copy numbers were normalized against *Hprt* gene and expressed as fold changes compared to control, *p < 0.5, **p < 0.01, t-test, n = 4, for control and CD, each biological replicate represents pooled testes. (**D**) A representative image of isolated E15.5 cells from testes. Testis cells were immunostained using anti-AMH1 (green) or anti-ESR1 (red) antibodies. In control (top panel) or treated (lower panel), nuclei are stained with DAPI and are shown in blue. Germ cells are negative for AMH, whereas Sertoli cells are positive. DAPI-stained cells represent all cells. In control, ESR1 staining appears as red nuclear and cytoplasmic; in CD-treated, as preferential nuclear staining. (**E**) Quantitative analysis of ESR1 signal in nuclei was performed using ImageJ software and presented as relative fluorescence +/−SEM, (p = 0.001, n = 3 control, n = 3 CD samples, t-test).

We first asked whether any CD effect on germ cells could be detected at the time of the exposure. To answer this question, we counted germ cells in E15.5 F1 testis by immunostaining for a germ cell marker, DDX4 (also known as MVH); we also stained testis for AMH, which is specific for Sertoli cells (Fig. 1A). The analysis of cell markers revealed that there is no significant change in germ cell numbers per unit area of seminiferous tubule in the CD-treated animals (Fig. 1B).

To establish whether the exposure to CD induces changes in gene expression, we performed the analysis of RNA in E15.5 testes. For the analysis we chose genes, which are essential for germ cell specification (*Prdm1*), maintaining the pluripotent state (*Pou5f1*), and Sertoli cells function (*Sox9*). In addition, we checked by qPCR some genes essential for germ cell development (*Tgif1, Zfr, Nlrc5, Tnks, Tex15, Ccne2 and Chd1*).

We found that several genes important for cell division (e.g. *Tgif1, Zfr, Prim2, Tex15, Tnks*) have decreased expression in F1 embryos (Fig. 1C). Moreover, we found that the expression of the master regulator of pluripotency, *Pou5f1*, decreased in testis exposed to CD during embryonic stage*;* this finding is consistent with previous observation of the CD effect on *Pou5f1* expression^39^.

To explore whether CD can directly affect the binding capacity of ESR1, we performed the analysis of ESR1 binding at the time of the CD exposure in E15.5 F1 testis. First, we verified that ESR1 is present at E15.5 by analysing the cell fractions from the testis. We found that ESR1 is strongly expressed in Sertoli cells, where it showed the nuclear and cytoplasmic pattern of expression. In contrast, the expression of ESR1 in SG was weak. The exposure to CD leads to an increase in nuclear staining of ESR1 in Sertoli cells (Fig. 1D,E).

### Gestational exposure to CD leads to decreased germ cell number, reduced epididymal sperm reserve and affects meiosis in the F1 and F3 generation males

**Figure 2:**
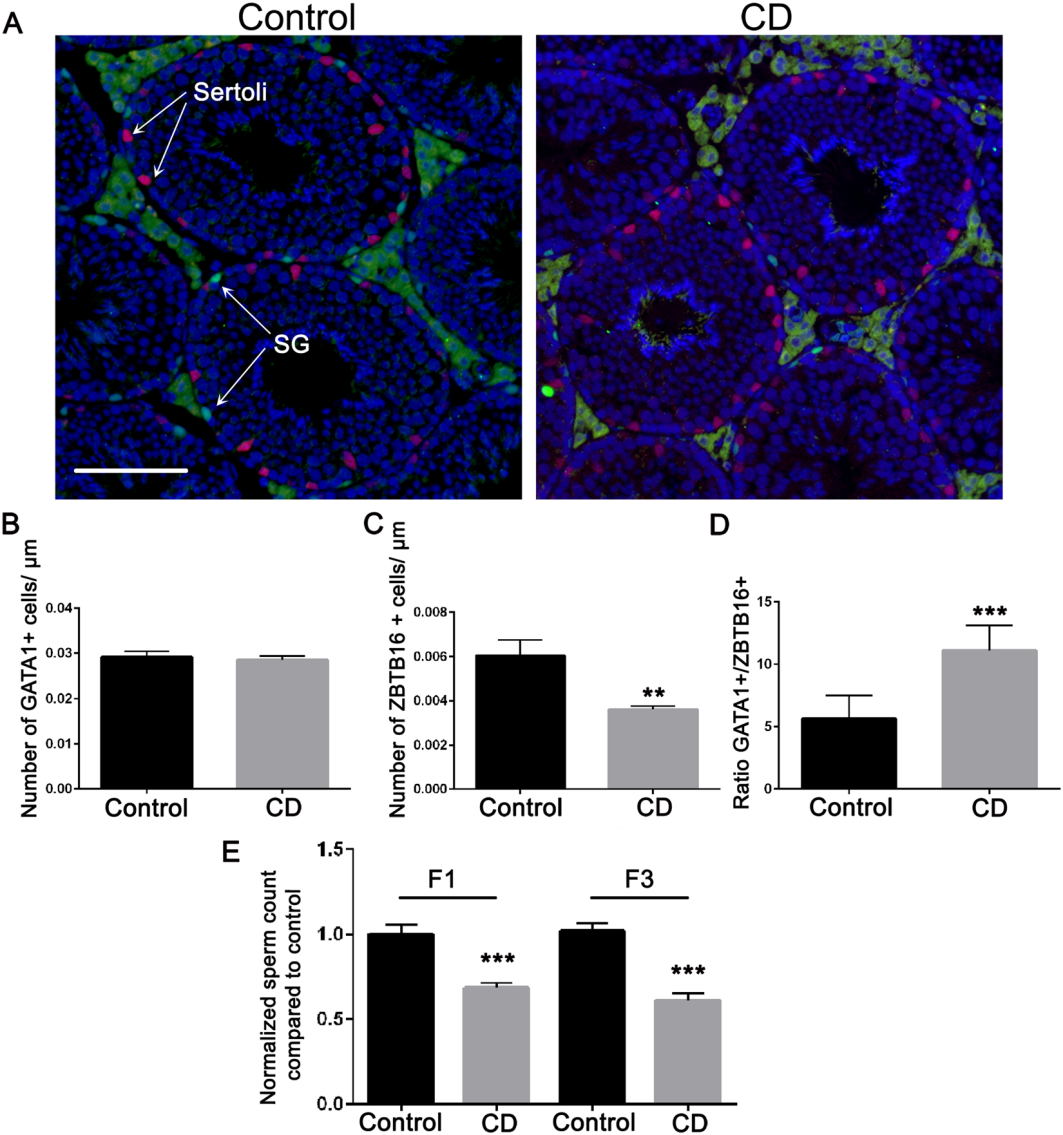
Gestational exposure to CD decreases the number of undifferentiated SG in the F3 generation males and leads to a decrease in spermatozoa number in F1 and F3. (**A**) Representative images of testes sections from the control (left panel) and CD lineage (right panel) animals: Sertoli and SG cells were immunostained using anti-GATA1 (red) or anti-ZBTB16 (green) antibodies, respectively. The ZBTB16 antibody staining of Leydig cells located outside of seminiferous tubules is non-specific, scale bar 150 µm. A quantitative analysis of (**B**) Sertoli cells and (**C**) SG was performed by manually counting the cells at stage VII of the seminiferous epithelium in F3 mouse testes. The contour of each tubule section was measured using ImageJ. The values shown indicate the cell counts per micrometre of tubule circumference. (**D**) The ratio of the number of Sertoli cells per SG is also indicated (**p < 0.01, n = 7 control, n = 6 CD, t-test). The immunostaining of the testis sections was performed as described in the Methods section. (**E**) Spermatozoa numbers in F1 and F3 have decreased. Spermatozoa were counted as described in the Methods section, by using epididymis of at least 8 biological replicates for control and CD and data presented as normalised value compared to control +/−SEM, (F1: *p* = 3.69E-4, n = 9 control and CD; F3: *p* = 1.64E-5, n = 8 control, n = 9 CD, t-test).

To determine whether the relative proportions of germ and Sertoli cells were altered, we immunostained F1 and F3 testis sections using antibodies against proteins specific to these cell types (i.e., ZBTB16 and GATA1, respectively) (Fig. 2A and Supplementary Fig. 2A). We observed a significant increase in the number of Sertoli cells in CD-derived F1 males (Supplementary Fig. 2B) and no significant changes in CD-derived F3 males (Fig. 2B). On the contrary, we did not observe any significant changes in SG number in F1 (Supplementary Fig. 2C and D), but we detected 2-fold decrease in the number of SG in CD-derived F3 males when compared to the control samples (Fig. 2C,D).

To reveal whether exposure to CD leads to changes in spermatozoa numbers, we counted the spermatozoa in epididymis of F1 and F3 males. We found that spermatozoa numbers have significantly decreased in both F1 and F3 (Fig. 2E) by nearly 30%. Moreover, the testis weight has been reduced in F3 but not in F1 males (Supplementary Fig. 3).

**Figure 3:**
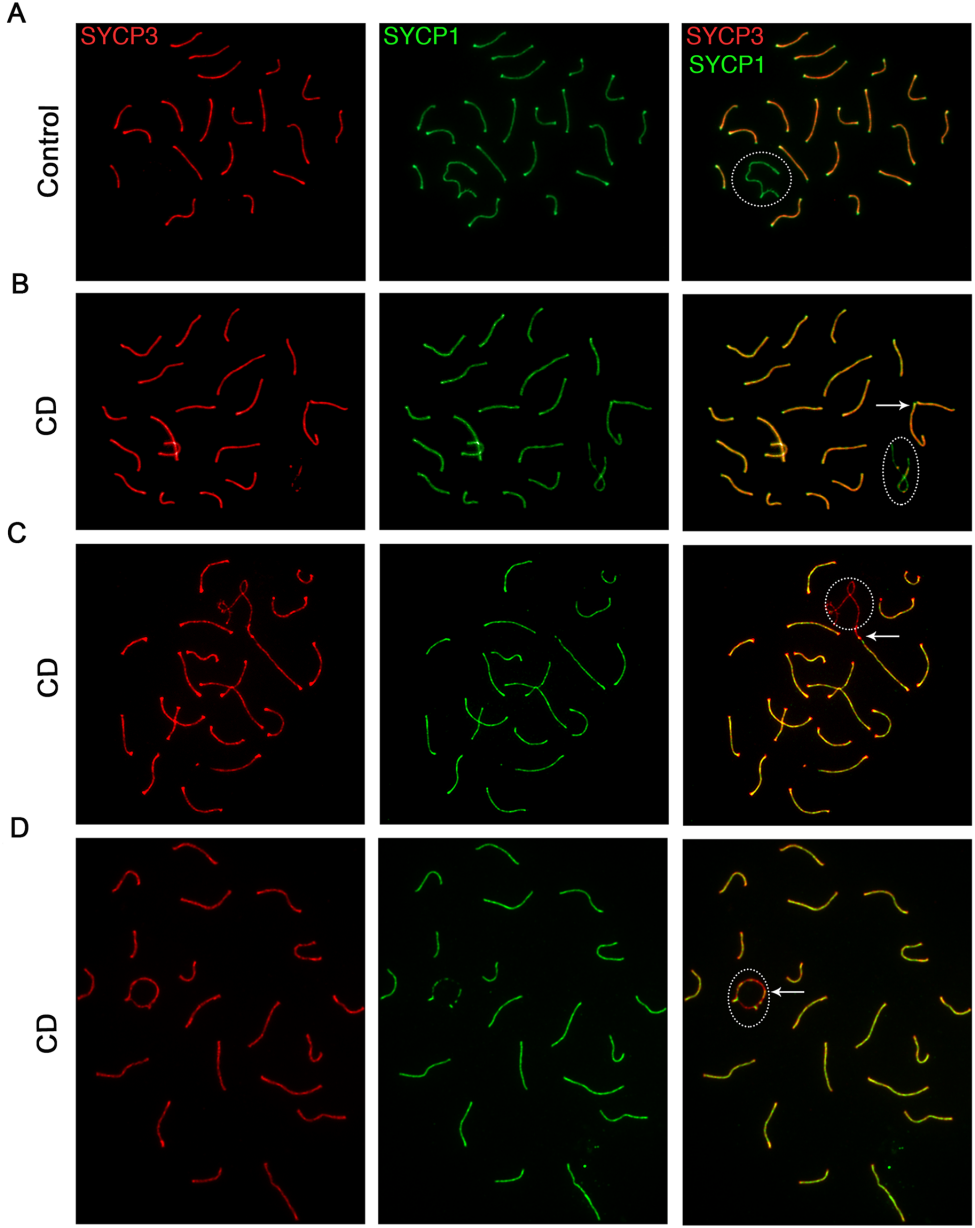
Gestational exposure to CD affects meiotic synapsing in F1 and F3 CD-derived males. Surface spreads were prepared from F3 generation testes obtained from control (**A**) or CD-derived males (**B**–**D**) males as described in the Methods section. The spreads were immunostained for a major protein of the lateral element of the synaptonemal complex (SC), SYCP3 (red, left panel), and for a protein expressed in the SC central element, SYCP1 (green, middle panel); the overlap between the two is also shown (right panel). (**A**) Representative images of a control testis spread. Normally, SYCP3 is detected along the length of every chromosome, while SYCP1 is expressed on all autosomal chromosomes and is visible as a punctate signal or absent immunostaining pattern in sex chromosomes (dashed circle, right panel). (**B**,**C**) In CD-derived males, meiotic cells with telomere connections and ring-like structure of sex chromosomes are notable. (**D**) The formation of “ring” like structures in sex chromosomes. X and Y sex chromosomes (sex bodies) are shown (dashed circle). The arrows point to chromosomes where telomeres are completely closed or fused.

Because SG give rise to other cell types, such as meiotic cells, in the testis, we sought to determine whether ancestral exposure to CD affects key differentiation processes during gametogenesis and meiosis. We isolated testes from F1 and F3 generation males and prepared testis surface spreads to analyse the formation of synaptonemal complex (SC), a structure essential for proper chromosome segregation before the first meiotic division^40^. We immunostained the surface cell spreads using antibodies against SYCP3 (a marker of chromosomal cores) and SYCP1 (a marker of fully synapsed chromosomes) and analysed the chromosome synapsing pattern during the *pachytene* stage (Fig. 3A–D). The analysis of SCs in the F1 and F3 generation of CD-derived males showed that there were several synapsing defects. Specifically, we identified the telomere-to-telomere connections (Fig. 3B,C) and the formation of ring-like structures between the X and Y sex chromosomes (Fig. 3D) as well as incomplete synapsing. The quantitative analysis of control- and CD-derived cells showed that there was a significant increase in meiotic defects in the germline of F1 and F3 generation animals that descended from CD-exposed males (p = 0.008 and p = 0.004, respectively, Chi-squared test, Table 1). Specifically, we found that telomere end-to-end connections increased 2.7 times in F1 and F3, and we observed the appearance of the ring chromosomes in 3.2% in F1 and 8.5% in F3 of cells in CD-derived samples, which were 2.9% in F1 and 0.37% in F3 in control mice. We also detected an increase in branched structures where several chromosomes were connected (2.1% in F1 and 2.5% in F3 CD), in addition to incomplete synapsing (16.5% in F1 and 5.8% in F3 compared to 1.2% in both control F1 and F3, Table 1).

**Table 1:** A quantitative analysis of meiotic synapsing defects.

	Fully synapsed	End-to-end	Ring sex chr.	Incomp.	Multiple con.	p-value
Cont F1	85.8 +/− 2.64	9.8 +/− 1.89	2.9 +/− 1.15	1.2 +/− 0.92	0.3 +/− 0.26	
CD F1	51.3 +/− 3.28	26.9 +/− 3.00	3.2 +/− 1.35	16.5 +/− 2.05	2.1 +/− 1.19	0.008
Cont F3	86.9 +/− 3.27	11.2 +/− 2.55	0.37 +/− 0.33	1.2 +/− 0.64	0.8 +/− 0.72	
CD F3	52.8 +/− 1.96	30.4 +/− 2.33	8.5 +/− 2.11	5.8 +/− 1.82	2.5 +/− 0.90	0.004

The classification in the table denotes fully synapsed chromosomes, telomere end-to-end connections, ring chromosomes (X and Y chromosomes form a ring), incomplete synapsing (one or two chromosomes have un-synapsed part), and multiple connections (several chromosomes are connected) (F1, *p*-value = 0.008, n = 5 control, n = 5 CD; F3, *p*-value = 0.004, n = 4 control, n = 4 CD, Pearson’s Chi-squared test).

To determine whether synapsing defects are due to inefficient DNA repair, we immunostained slides for the DMC1 protein, which specifically binds to single-strand DNA (ssDNA)^41^. Meiotic ssDNA is formed at the sites of double-strand breaks (DSBs), a critical step during meiosis because the formation of DSBs occurs throughout the genome. DMC1 binds to ssDNA and facilitates the search for a homologous chromosome. Once the homologous sequence is found, the break is repaired and DMC1 is removed. Therefore, in the *pachytene* stage, the number of DMC1 foci in normal cells is limited (Supplementary Fig. 4A). We found that in CD lineage-derived cells there was an increase in the DMC1 presence in chromosomes in *pachytene* stage; we counted the DMC1 foci in *pachytene* stage of prophase 1 in F1 and F3 progeny. The average number of DMC1 foci per cell has increased in F3 males 2,8 times (p < 0.0004, t-test) and 4 times in F1 (p = 0.06, t-test) (Supplementary Fig. 4E). For example, ring-like sex chromosomes exhibited a high intensity of DMC1 staining (Supplementary Fig. 4B–C), with DMC1 present in their subtelomeric regions (Supplementary Fig. 4D), suggesting that DNA repair is less efficient in some regions in the progeny of treated mice.

In summary, our data demonstrate that in F1 and F3 generation males derived from the CD-treated animals, meiosis have synapsing and DNA repair problems.

### Gestational exposure to CD is associated with the dysregulation of genes encoding proteins with roles in chromosome segregation, cell division and DNA repair

**Figure 4:**
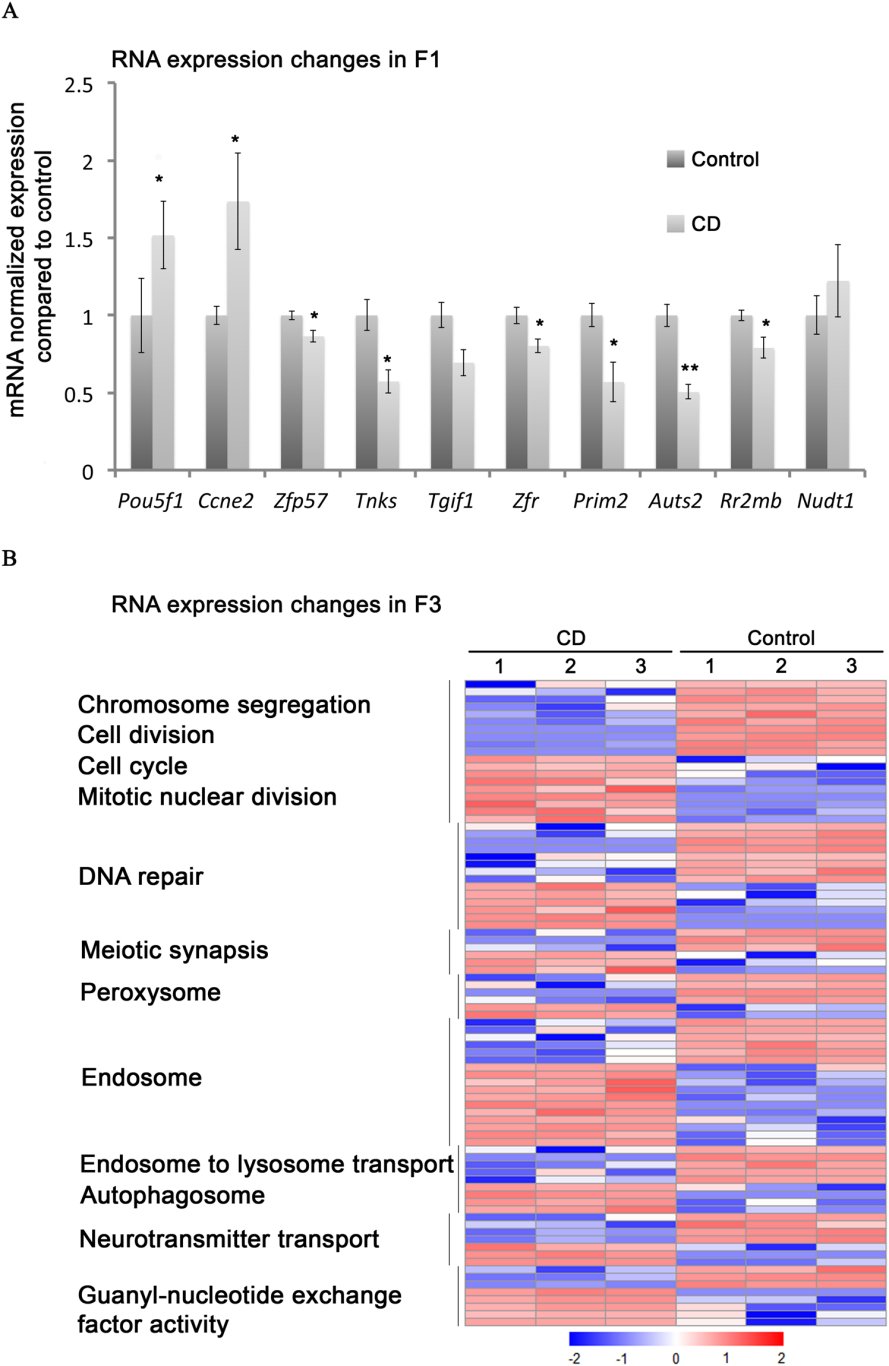
RNA expression changes in F1 and F3 CD-derived adult males. (**A**) The RT-QPCR data of RNA expression in F1. The RNA from 5 animals for each condition was extracted, reverse transcribed and analysed by qPCR for each target gene. The copy numbers of each target gene were normalised to *Actb* copy numbers, and data were presented as normalised values compared to control +/−SEM, (*p < 0.05, **p < 0.001, t-test). (**B**) A heatmap of the differentially expressed genes in the testes. The hierarchical cluster analysis identified 8 gene groups that could be segregated according to their expression levels in testis fractions. Red indicates high and blue indicates low expression.

To reveal the molecular mechanism of CD action, we performed analysis of RNA expression in F1 by RTq-PCR and transcriptomics analysis in F3 males, which were not directly exposed to CD. We found that genes that are implicated in the control of cell division were altered in F1 (Fig. 4A) and F3 (Supplementary Tables 1 and 2), including *Ccne2, Tnks, and Prim2*. We found that *Pou5f1* has increased expression 1.5 times in F1 males (p-value = 0.048). The analysis of RNA-seq of F3 males showed that *Pou5f1* is not differentially expressed. Based on our qPCR data, we found that master regulator of imprinting genes, *Zfp*57, has significantly reduced expression levels in F1 adult males (p-value = 0.046) but not in F3 males.

The transcriptomics analysis in the F3 generation whole testis using strand-specific paired-end high-throughput RNA sequencing revealed the changes of total 377 genes corresponding to 412 differentially expressed transcripts (Fold Change (FC) > 2 and FDR < 0.1) (Supplementary Tables 1 and 2, Supplementary Fig. 5). To determine the processes that could be perturbed in testis as a result of these changes, we performed a functional annotation of the differentially expressed genes (DEGs) using the Gene Ontology (GO) terms identified by DAVID. We found significant enrichment in expression of genes associated with cell division and chromosome segregation (Fig. 4B) including *Nek3, Phf13, Cenpc1, Ctcf, Pttg1, Mis12*, and *Stag2* (Supplementary Tables 1 and 2). Other enriched GO terms include genes associated with DNA repair, meiotic synapsis and autophagocytosis functions.

In summary, our data shows that exposure to low dose of CD during gestation causes a global perturbation of the transcriptional network that can be detected in the F3 generation. The expression of *Pou5f1* gene, known to be essential for establishment of pluripotent state in germ cells was affected by CD exposure.

### Analysis of H3K4 trimethylation reveals a global change in the distribution of these marks in CD-derived testes of F1 and F3 generation males

**Figure 5:**
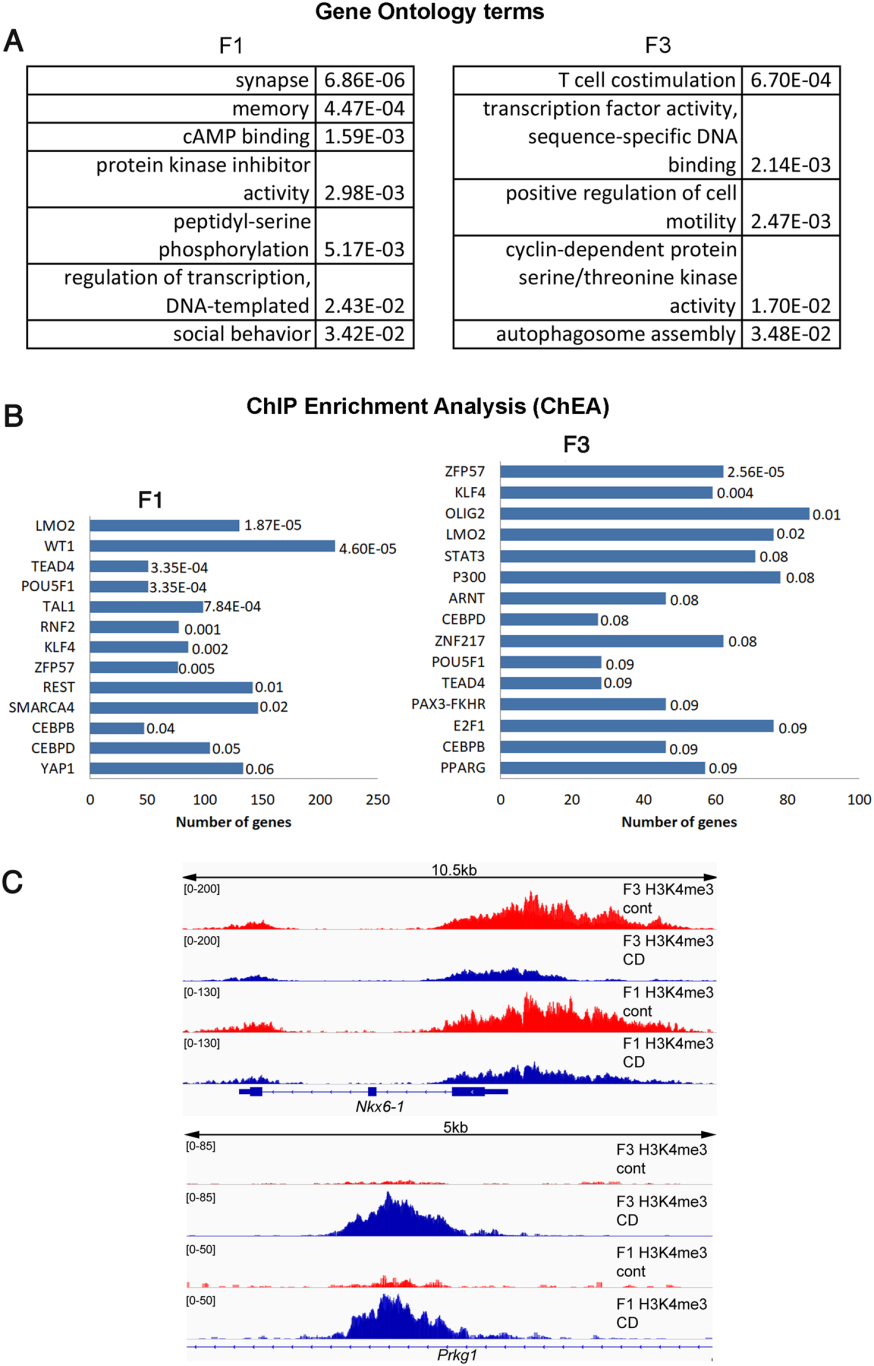
Histone H3K4me3 occupancy is globally affected in F1 and F3 generations following CD treatment. (**A**) The gene ontology (GO) terms are sorted based on *p*-value, calculated by DAVID, *p*-values are indicated against GO terms in the table, Fisher’s exact test. (**B**) Enrichment in GO terms located within the differential peaks identified by ChEA. Terms are sorted based on adjusted *p*-values and are indicated at the end of each bar. (**C**) The H3K4me3 occupancies in *Nkx6-1* and *Prkg1* have been altered in both F1 and F3 generations males.

To determine whether gestational exposure to CD affects histone epigenetic marks in F3 generation, we performed a genome-wide distribution analysis of methylated histone H3K4me3, which is a pivotal mark of transcriptional start sites (TSSs)^42,43^. We performed ChIP-seq on the F1 and F3 generation of CD-derived and control male whole testes. Using similar parameters for the analyses of F1 and F3, we identified 1365 and 761 peaks that were differential between control and CD-derived males in F1 (Supplementary Table 3) and F3 (Supplementary Table 4), respectively. We assigned the genes to associated regions with GREAT and performed the functional annotation of the genes using DAVID. We found that F1 and F3 differential peaks are significantly enriched in several GO terms (Fig. 5A). The common features that we identified in F1 and F3 males were the over-representation of genes associated with transcription factor activities (*Isl1*, *Nkx6-1*, *Tgif2*, *Wwc1*, *Insm1*, *Tshz3*, *Trim24*) (Fig. 5A and Supplementary Tables 3 and 4)

Next, we used ChEA (Chip Enrichment Analysis) to compare the differential regions with published datasets. The ChEA analysis revealed that the targets of POU5F1 and ZFP57 were significantly enriched both in F1 and F3 differential peaks (Fig. 5B). For example, master regulator of pluripotent genes, POU5F1 has 50 targets in F1 generation (*p*-value = 2.09E-06, adj. *p*-value = 3.35E-04), out of the 559 targets identified in mouse mesoderm cells^44^ (Supplementary Table 5). In F3, POU5F1 has fewer (28 target genes) located in differential peaks (*p*-value = 1.74E-03, adj. *p*-value = 9.23E-02) (Supplementary Table 6). Among targets of POU5F1 that are found in the vicinity of differential peaks in F1, there are genes involved in cell adhesion (*Cdh2*, *Cdh6*, *Cdh11*, *Hapln1*), signal transduction (*Lhx5*, *Lingo5*, *Tiam2*) or Wnt- signalling pathway (*Disc1*, *Ror1*), whereas in F3 peaks, there are enrichment in genes encoding proteins which are essential for stem cell maintenance (*Kitl)*, canonical Wnt*-*signaling (*Ctnnd2*) pathways and proteins with receptor functions (*Mc2r, Tlr4*). ZFP57 has 76 targets out of 1088 in F1 (*p*-value = 5.65E-05, adj. *p*-value = 4.54E-03) (Supplementary Table 7), and 62 targets for the differential peaks observed in the testis of F3 progeny (*p-*value = 4.01E-08, adj. *p*-value = 2.56E-05) (Supplementary Table 8). Common to F1 and F3 are the ZFP57 target genes encoding proteins with cell adhesion function such as cadherins (*Cdh2* and *Itga6*, both having increased H3K4me3 occupancy). Although F1 males have altered histone occupancy in genes encoding for different adhesions molecules (e.g. *Cdh7, Cdh11, Cdh13, Pcdh17, lsamp, Itga6, Hapln1 and Col19a1)* compared to F3 males (*Cdh6, Cdh9, Ctnnd2, Cntnap5a* and *Pcdh9*), in both generations, genes for adhesion molecules are the most over-represented. We asked whether the targets of ZFP57 are preserved in paternal histone-containing portion of sperm, and early zygotes. We compared all known target genes of ZFP57 with recently available datasets of sperm histone H3K4me3 containing fraction^10^. We found that 692 out of 1088 (63.6%) of all known targets of ZFP57 found in mouse genome retain their histones in sperm (Supplementary Fig. 6A) and 47.8% are preserved in paternal histone fraction in 8-cell embryo (Supplementary Fig. 6B)^10^.

To determine whether *Zfp57* is differentially expressed at the time of imprint establishment during embryonic stage, we compared *Zfp57* expression in control and CD-exposed E15.5 testis by RT-qPCR. We found that *Zfp57* expression was significantly reduced by 25% compared to control testis (Fig. 1C). In addition, we asked whether target genes of ZFP57 are differentially expressed in embryonic testes. We performed RT-qPCR of RNA from embryonic testes and confirmed that at least some of the ZFP57 target genes (e.g., *Itga6, Ctnnbl1*) have altered expression suggesting possible role for ZFP57 in their regulation (Supplementary Fig. 7).

In F1, we found decreased H3K4me3 occupancy near *Prdm1* gene (Supplementary Fig. 8). In contrast, in F3 males, there are no changes in H3K4me3 peak occupancy in this location. We found that expression of the *Prdm1* gene has also decreased in embryonic gonads (Fig. 1B), and it is not expressed in adult testes.

In addition to altered peaks, we identified 476 peaks in F1 and 182 peaks in F3 that appeared *de novo* or disappeared in F3 progeny of treated mice, including peaks near *Ppp2r3a* (associated with canonical Wnt-signaling pathway), *Foxa2 (*Notch signalling pathway) and *Agtr1b*, *Gpr139, Olfr720* (G–protein coupled receptor signalling) (Supplementary Tables 9–12).

To examine the relationship between differential peaks in F1 and F3 generations, we intersected the two datasets. Out of 761 regions, we found 54 peaks (7.1%) of altered H3K4me3 occupancy in F3 progeny that could be detected in F1 males as well. While the number of overlapping peaks is relatively low, they represented 76 genes with important cellular functions (Supplementary Table 13). The common peaks identified have either decreased or increased H3K4me3 occupancies. For example, genes *Nkx6-1* and *Prkg1* have decreased and increased peak occupancies in both generations, respectively (Fig. 5C). The overall pattern of differential peak locations relative to TSS is similar in both generations (Supplementary Fig. 9).

In summary, our data suggest that multiple altered H3K4me3 peaks are found in the F1 generation of CD-exposed males and that a limited number of these changes is preserved in non-exposed F3 generation, with ZFP57 being a strong candidate for mediating the transgenerational inheritance.

### ESR1 binding motifs are enriched in H3K4me3 peaks in genes associated with transcription factor activities and chemokine signalling

Because CD is capable of binding to oestrogen receptors, we next asked whether the binding sites for ESR1 are located within altered peaks. Using FIMO tool^45^, we identified 287 sites in F1 and 279 potential ESR1 binding sites within differential peaks in the F3 generation (Supplementary Tables 14 and 15). With Motif Enrichment Analysis tool (AME^46^), we found that ESR1 motif is significantly enriched in differential H3K4me3 peaks of F1 generation males (*p-*value = 0.0171) and the number of ESR1 sites in differential H3K4me3 peaks is reduced in F3 peaks but still high (*p*-value = 0.066).

We found that differential peaks containing ESR1 motifs were enriched in genes associated with myosin II complex in F1. In F3, differential peaks containing ESR1 motifs were enriched in genes associated with chemokine receptor-binding functions and in genes with transcription factor activities. Specifically, altered peaks containing ESR1-binding motifs in F3 were organized in a large domain on chromosome 4 and were located in a region where a family of chemokine genes (including *Ccl19, Ccl27b* and *Ccl27a* among others) resides.

**Figure 6:**
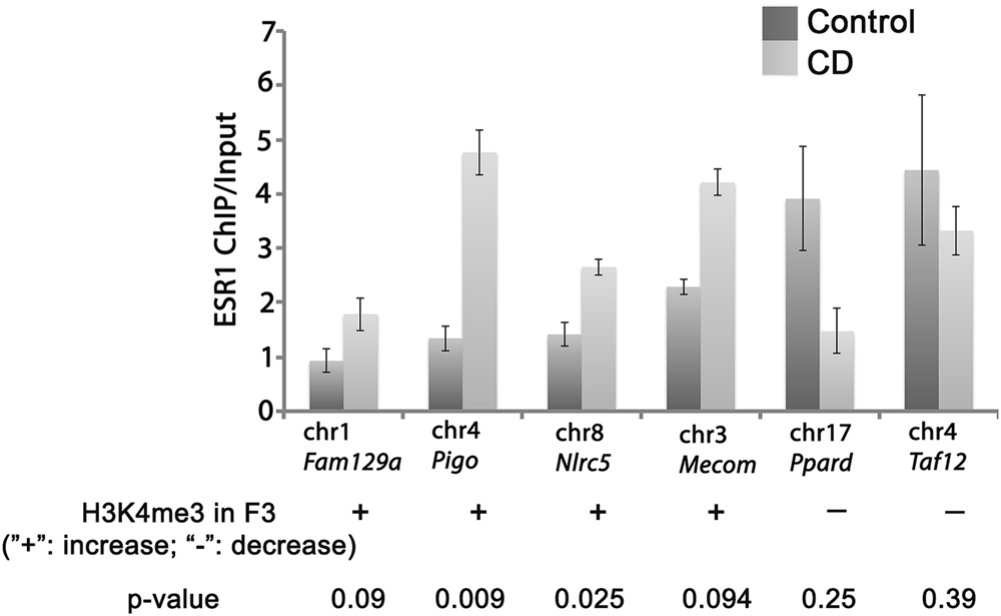
ESR1 binding is altered in CD treated testes. The ChIP was performed using antibody against ESR1 in E15.5 testis. The immunoprecipitated DNA was quantitated with fluorescent method and equal amount of ChIP and Input DNAs was taken for each PCR using the primers indicated in SI. The ESR1 predicted binding sites were obtained by FIMO analysis and primers were designed after repeat masking of each target sequence. The copy number for each gene was normalized for quantity to control region without ESR1 binding sites (*Gapdh* gene) and compared to Input. The data was expressed as ChIP-to-input enrichment.

To analyse ESR1 binding in embryonic testis, we performed ChIP-qPCR using ESR1 antibody. For the analysis of ESR1 binding sites, we chose primers within differential H3K4me3 regions sites with several predicted ESR1 binding sites located close to each other (FC > 1.5, FDR < 0.1). ChIP-qPCR analysis showed that at least some predicted sequences are bona fide ESR1 binding sites (Fig. 6). We found a significant increase in ESR1 binding in regions of *Pigo, Nlrc5* genes. In general, the change in ESR1 binding positively correlates with H3K4me3 occupancy.

In aggregate, our data strongly support the hypothesis that CD effects are, at least partially, mediated through altered ESR1 binding.

### Histone H3K27me3 and H4K5Ac occupancies are altered in F3 males

We also asked whether other epigenetic histone marks are affected upon CD exposure. We performed ChIP-qPCR analysis of H3K27me3 and H4K5Ac using whole testes of F3 males. We chose the common regions with altered H3K4me3 occupancy in both F1 and F3 males and regions with ESR1- binding sites for qPCR target analysis. We found that changes in H3K4me3 occupancies in F3 males have stronger overlap with changes in H3K27me3 when compared to H4K5Ac occupancies (9 out 16 sites were changed in H4K27me3 occupancy (e.g. *Myo1d, Ppp2r2d, Elac2, RNase10*) (Supplementary Fig. 10). In contrast, only 5 out of 16 targets have altered H4K5Ac occupancy (Supplementary Fig. 11). H4K5Ac mark occupancies increased in vicinity of *Cdh2* and *Ppp2r2d* genes, similar to changes in H3K4me3 in F3. In summary, our data suggests that other epigenetic histone marks are also affected due to ancestral CD exposure and these changes are still evident up to third generation.

### An integrative analysis of ChIP-Seq and RNA-seq data in F3 generation reveals CD-mediated changes in gene expression and histone H3K4me3 occupancy in a subset of genes associated with cell division, ATP binding and transcription

It was previously suggested that the presence of H3K4me3 marks in the regulatory regions of genes has low correlation with their expression^4^. Instead, it was argued that histone marks provide memory of recent transcriptional activity and that marks not coupled with active transcription are involved in transcriptional regulation processes (reviewed in^47^).

Nonetheless, H3K4me3 marks are lineage-specific and are highly conserved in mammals, similar to the binding sites for transcription factors (which are determined by these marks) that are also organized in conserved domains^48,49^. It is, thus, conceivable that in some cases histone marks could inform gene expression. To explore the correlation between histone marks and DEGs, we identified peaks overlapping DEGs. We found that 25 genes out of 377 DEGs were present in both datasets. These genes are associated with cell division (*Prim2, Rb1*), ATP binding and transcriptional regulation (*Nkx6-1, Zfp735*) (Supplementary Fig. 12).

In summary, our data confirm the notion that, while a subset of DEGs harbours H3K4me3 peaks, the mere presence of a differential H3K4me3 peak is not strongly correlated with transcriptional activity.

## Discussion

### The adverse effects of CD on germ cell development

In this study, we aimed to reveal the potential effects of CD on reproductive system. Particularly, we were interested to see whether the changes in germ cells observed in the F1 generation could be detected in F3 generation males, a progeny with no direct CD exposure.

The developmental potency of a mammalian embryo is substantially dependent on transcription factors, POU5F1 and ZFP57. They play an important role in the control of embryonic stem cell fate^50^ and are key components of the regulatory processes that govern pluripotency, activate stemness-related genes and repress genes required for differentiation^51^. During embryogenesis, POU5F1 is highly expressed in pluripotent cells, becomes downregulated upon differentiation, and gets confined to the primordial germ cells^52,53^. Similarly, ZFP57 is expressed in the pluripotent embryonic stem cells and is a transcriptional target of POU5F1^54,55^, reviewed in^56^. It is a transcriptional repressor that plays a key role in the maintenance of imprinted DNA methylation. The interaction between transcriptional regulation, epigenetic states and chromatin structure has been amply documented in various developmental and diseased states; however, its importance for transgenerational inheritance remains poorly understood. In our study, we found that expression of *Pou5F1* and *Zfp57* is affected by CD. We suggest that this could impact the epigenetic state of their targets in embryonic germ cells and modify all future cell types derived from them.

We observed a decrease in SG numbers in the F3 generation of CD lineage males. We propose that decrease in SG numbers in F3 adult mice resulted from CD-mediated ancestral exposure. However, we did not detect a decrease in gonocytes which suggests that the observed changes did not arise from changes in germ cell number rather from alterations in gene expression or/and chromatin epigenetic state. The decrease in germ cell number in F3 generation could possibly be explained by altered epigenetic state of POU5F1 target genes that included genes involved in regulation of cell division (e.g., decrease in H3K4me3 occupancy at *Kitl*, *Ctnnd2, Fntb and Creb)* and increase in H3K4me3 occupancy of ZFP57 target genes (such as *Nr4a2*, *Pik3C3* and cadherins: *Cdh2, Cdh6, Cdh9)*.

The morphological analysis of the testis sections by using hematoxylin and eosin staining did not show any obvious changes in cell morphology or changes in the proportion of cell types. However, we cannot completely rule out the possibility that at least some of the observed changes in F1 and F3 males could be due to the changes in testicular cell types other than germ cells. For example, we cannot exclude the possibility that CD-induced changes in Sertoli cells could also contribute to the phenotypes that we observed. Indeed, we found that expression of *Sox9* was reduced in embryonic gonads. *Sox9* is expressed in Sertoli cells and plays an important role in male sex determination. It is suggested that *Sox9* regulates SF1, also known as *Nr5a1*^57^. NOTCH signalling in Sertoli cells plays an essential role in gonocytes’ fate through regulation of cell cycle proteins and KIT ligand^58^.

The synapsing defects detected in the meiotic SC fraction in F1 corroborate the notion that the changes we observed originated from the alterations in the gonocyte transcriptional network and persisted, to affect the function in their descendants. A number of impaired telomere connections during meiosis was notably different between the control and the CD-derived samples. The telomeres are important for meiosis initiation, and many DNA repair proteins are located at telomere ends. We observed altered H3K4me3 peaks in the F1 and F3 CD lineage for genes associated with telomere function, such as *Tnks*, *Rad51d, Pinx1*; we also determined that, in both F1 and F3, RNA expression of *Tnks* gene has been reduced. We postulate that these changes could be inherited through preservation of epigenetic histone marks and, therefore, become a major factor resulting in deficient meiosis. The dysregulation of telomere functions leads to severe genomic instability and chromosome end-to-end fusions^59^, suggesting the essential role of the telomeric proteins in safeguarding genomic stability. We previously noted similar outcomes in a transgenerational study with ATZ^14^, and conclude here that telomere-maintaining proteins are especially sensitive to toxic compounds and that toxicant-induced changes are associated with meiosis synapsing defects.

We found that ancestral CD exposure leads to meiotic DNA repair defects in F1 and F3 males. We suggest that dysregulation of important DNA repair genes such as *Mre11a, Dmc1, Xrn2, Fancg, Smug1* and *Rdm1* could contribute to delay in DNA repair process in meiotic cells. For example, *Dmc1*, the gene important for meiotic DNA repair, is upregulated in CD-treated male progeny; the upregulation of *Dmc1* transcription was consistent with the increase in DMC1 foci in meiotic cells. We further suggest that changes identified in earlier cell types, both premeiotic and meiotic, could contribute to observed spermatozoa decline. It has been shown that exposure to CD causes decrease in epididymal sperm in rats without any changes in morphology of reproductive organs^60^. It was suggested that any drug that affect spermatogonia could lead to defects in sperm maturation and spermatogenesis^61^. We did not perform fertility tests, as studies have shown that even a 50–60% decline in sperm count will have no appreciable effect on fertility in mice (Schurmann *et al*., 2002; Zhang *et al*., 2006).

### CD-mediated changes in H3K4me3 and other epigenetic marks

This study identified over 700 regions where the H3K4me3 peaks are altered between the control and experimental animals in F3. However, only a fraction of these corresponds to DEGs. Similar to Page and colleagues, we now favour the explanation that altered H3K4me3 peaks correspond to genes that are maintained in a poised state^4^.

Recent studies that examined the dynamics of histone epigenetic marks in early zygotes provided evidence that H3K4me3 proteins transmit the memory of paternal histones^9,10^. The enrichment of targets for ZFP57 in altered peaks in both F1 and F3 generations implicates ZFP57 as a potential key mediator of transgenerational inheritance. ZFP57 is a master regulator involved in the establishment and maintenance of the imprinting marks (^55,62^; reviewed in^63^). ZFP57 can also bind to non–imprinted genes at poised enhancers and can repress *cis*-acting regulatory elements whose activity has to be silenced during development^64^. The genes encoding the adhesion molecules are over-represented in both F1 and F3 datasets. Given the role of cell adhesion molecules in controlling cell division (e.g.^65^), it is possible that their increased expression in CD-lineage can hamper this process. The H3K4me3-containing peaks that overlap between F1 and F3 generations are likely to be the best candidates for conferring the epigenetic memory of CD effects even though they represent less than 10% of all altered peaks. This data lends further support to the idea that modifications imposed during developmental exposure to CD could be transmitted to next generations through ZFP57-mediated epigenetic regulation. We suggest that a decrease in ZFP57 (a repressor) could lead to increased expression of its targets (for example, expression of cadherin genes), as observed.

However, further work is required to establish whether change in ZFP57 activity directly mediates the transgenerational effects. A ChIP with an anti-ZFP57 antibody could be used to identify its targets in embryonic CD-treated progeny.

We found that histone modifications other than H3K4me3 could be contributing to CD promoted phenotypes. For example, we determined that H3K27me3 and H4K5ac have altered occupancies at least at several genes with changed H3K4me3 peaks. H3K27me3 mark is suggested to be a strong candidate for mediating transgenerational inheritance as it is found at many poised developmental genes^4^ and is enriched in embryonic stem cells^1^ and sperm^5^. Similarly, H4K5Ac is an important mark for bromodomain-containing protein (BRDT) binding and it is critical for histone-to-protamine transition^66^. We suggest an interplay between these epigenetic marks in the establishment of a new chromatin state in CD-derived mice, which promote changes in gene expression at certain developmental period. However, the direct interactions between these marks are yet to be established.

### The ESR1-binding potential of CD

Oestrogen receptor (ESR1) is a key component of cellular differentiation that regulates gene transcription through its cognate DNA oestrogen response elements (ERE). There are more than 70,000 EREs in the human genome, in addition to the regions that contain half EREs or degenerate EREs, that can also recruit ESR1^67^. However, oestrogen-induced ESR1 DNA binding is restricted to only a subset of the EREs (e.g.^68^) in different cells, implicating factors other than DNA sequence in guiding its recruitment. It has been demonstrated that ESR1 preferentially binds to EREs located in the open chromatin^69^ and relies on the pioneer transcription factor for chromatin access (reviewed in^70^). The pioneer factors often require methylation of H3K4 for their binding to guide activated ESR1 to its ERE targets.

As discussed in the introduction, CD can bind to ESR1 and mediate its adverse effects. We provide evidence which suggests that CD can use an ESR1-dependent mechanism to mediate transgenerational effects. Particularly noteworthy is the presence of potential ESR1 binding sites in the regulatory elements of chemokine encoding genes. Chemokine networks regulate a variety of cellular, physiological, and immune processes (reviewed in (51)). Among these are inflammatory (Il-1) chemokines as well as homeostatic chemokines that serve to direct cell movement towards a target tissue. The homeostatic group includes chemokines such as CCL9, CCL20, CCL21 and CCL27. Importantly, chemokines play a role in primordial germ cell development. For example, CXCR4 is a crucial chemokine receptor that controls primordial germ cell (PGC) homing (54). We suggest the possibility that altered chemokine expression following CD exposure could be one of the reasons for the observed decrease in epididymal sperm count in CD lineage.

In addition to genes encoding for chemokines, we identified developmental genes that harbour evolutionarily conserved ESR1 sites in their regulatory regions. *Nkx6-1* is important for suppressing epithelial-to-mesenchymal transition (EMT)^71^. EMT could also be mediated by chemokines, at least in some cases^72^, suggesting the possibility of the interplay between chemokines and *Nkx6-1* in the ESR1-dependent EMT control. Exposure to CD is associated with an increased risk of prostate cancer^26^ and a decrease in *Nkx6-1* gene expression and deregulated EMT could be contributing factors. Whether there is a connection between the decreased expression of *Nkx6-1* and the risk of cancer remains to be established. The nuclear receptors (RARB and THRB) that are known to cooperate with ESR1 signalling^73,74^ also harbours the binding motifs for ESR1 within differential peaks, suggesting the potential role for these receptors in mediating the CD effects. In summary, our data suggest that the interplay between POU5F1, ZFP57 and ESR1 network is important for transgenerational inheritance of epigenetic changes caused by endocrine disrupting chemicals (e.g., CD).

### Does CD promote inheritable changes in germline?

It stands to reason that a large number of altered peaks that we observed are secondary to a limited number of heritable primary changes in the genome, as most of the histone marks disappear during the histone-to-protamine transition. Since altered H3K4me3 peaks are located proximal to the binding sites of important transcription factor (TF) regulators, perturbed TF binding could globally affect the germ cell gene expression program, and eventually, all cells types that originate from SG^75^.

A limitation of this study was the use of the whole testis for identifying the epigenetic and expression changes in F1 and F3 generations. While changes in the F3 generation can only arise through the germline, we cannot currently attribute specific F1 changes to any single testis cell type. To better assign and directly relate changes between generations, ChIP in sperm fraction of mouse genome would be required. Sperm contains up to 10% of the histones^5^, and the recent data on human and bovine sperm histone distribution show that sperm contains a limited number of histone-associated promoters including genes associated with RNA and protein processing functions^8^. We compared the genes, which are retained in human histone containing fraction and the genes located in our differential peaks. We found that 40% of the genes located in differential peaks in F1 and F3 males are preserved in the human histone-associated sperm DNA (Supplementary Fig. 13). Specifically, some of the ZFP57 targets and TF-associated genes are preserved in the histone-containing fraction of the sperm. Since histone-containing fraction is mostly evolutionary conserved in mammalian species, we suggest that at least some of the observed changes in epigenetic marks could have arisen from sperm histones and got transmitted to next generation. However, we cannot exclude the fact that some changes could be derived from somatic tissues, which is also derived from paternal sperm.

How long would these effects persist? From our current and previous studies, we arrive at the conclusion that epigenetic changes decrease from F1 to F3 generations. For example, POU5F1 has fewer targets in F3 compared to F1, suggesting that epigenetic changes are erased with every generation.

It is also possible that some of the changes we observed could be accounted for by other epigenetic mechanisms such as noncoding RNA. The analysis of our RNA-seq data showed that expression of several noncoding RNAs was modified (Supplementary Table 16) and these (or additional RNA species that we have not examined) could also contribute to the observed phenotype in testis or other organs. It has been shown that sperm contains a large number of noncoding RNAs^76^, which potentially could contribute to transgenerational inheritance. For example, long non-coding RNA *1700001G01Rik* which was up-regulated in F3 testis was found in spermatozoa fraction of RNA in a previous study (72).

In summary, addressing the limitations of current study in future experiments will undoubtedly lead to a better understanding of transgenerational effects mediated by CD in the germline.

## Conclusion

Our data suggest that the current toxicological index used to establish CD safety reference value for protecting human health could be underestimated. This index is based on the highest oral dose of CD (50 µg/kg bw/day), chronically administered to adult rats for at least one year. This dose does not significantly increase the incidence of the critical toxic effect observed in rats (proteinuria^77^) and it is close to the dose used in our study. Since mechanisms involving epigenetic memory are highly conserved between rodents and humans, and because our observed effects result from a relatively short exposure time during gestation, current CD safety reference values with a relevant margin of safety may need to be reconsidered.

## Methods

### Ethics statement

Studies using animals including euthanasia, and other experimental procedures, were conducted in accordance with the French regulation for Laboratory Animal Care, issued from the transposition of the European Directive 2010/63/UE. The animal facility used for the present study is licensed by the French Ministry of Agriculture (C35-238-19). All animal procedures were licensed by French Ministry of Agriculture and conducted by A. Gely-Pernot and F. Smagulova (agreement number 01861.02).

### Animal experimentation

Outbred Swiss mice were used for all experiments. After breeding, the day of vaginal plug detection was considered embryonic day 0.5 (E0.5). CD (Santa Cruz, Dallas, Texas 75220, U.S.A) was suspended in olive oil and administered at 100 μg/kg/day in a volume of 150 μl for each mouse. CD was administered via oral gavage to 8-week-old pregnant mice from E6.5 to E15.5. The control mice were treated with the same volume of oil. These control and CD-treated mice are called F0. Male progeny of F0 females are called F1 and were crossed with non-littermate and untreated female to give rise to F2 generation. Male progeny of F2 were crossed with non-littermate and untreated female to give rise to F3 generation.

Animals from at least three cofounders from independent F0 pregnancies were used for most data points. Specifically, for RNA-seq analysis, germ cell and for meiosis analysis we used at least three independent biological replicates. For Chip-seq, we used two independent biological replicates from two treatment groups. For the analyses of F1 embryos (for ChIP), we pooled fetal testes from 5 gestated females to have one biological replicate for ChIP. Embryonic testes were pooled for ChIP- qPCR and RNA expression analysis. For the analyses of F1 adults, we used three independent treatments and the animals were randomized and used for ChIP and for RNA analysis. For RT-qPCR, we used 5 control and 5 F1 progeny males.

The mice were sacrificed via euthanasia using a CO_2_ gradient. The reproductive organs were dissected out and either fixed or kept at −80 °C until use.

### Germ cell/Sertoli cell numbers

For immunohistochemistry (IHC), the animals were perfused, and the testes were excised and fixed for 24 hours in 4% (wt/vol) paraformaldehyde (PFA), dehydrated and embedded in paraffin. For IHC, 5 µm-thick testis sections were incubated overnight at 4 °C with goat anti-ZBTB16 (diluted at 1:500) and rat anti-GATA1 (diluted at 1:50) antibodies. The sections were counterstained with 0.001% (vol/vol) 4,6-diamidino-2-phenylindole dihydrochloride (DAPI) and mounted in Vectashield (Vector Laboratories, UK). To quantify the number of Sertoli cells (GATA1-positive cells) and undifferentiated SG (ZBTB16-positive cells), we manually count the cells on around 30 sections of seminiferous epithelium at stage VII in F3 mouse testes taking in 3 different areas of the testis for each biological replicate (n = 7 for F3 control and n = 6 for CD condition). The perimeter of each tubule section was measured using ImageJ. The values shown indicate the cell counts per micrometre of tubule circumference. The ratio of the number of Sertoli cells per SG is also indicated. For embryonic germ cell counting, we dissected embryos at E15.5 and fixed testes in 4% PFA, dehydrated and embedded in paraffin. 5 µm-thick testis sections were incubated overnight at 4 °C with rabbit anti-DDX4 (diluted at 1:300) and goat anti-AMH (diluted at 1:300) antibodies. The germ cells were counted at least for 20 seminiferous tubules in 4 biological replicate control and 5 for CD, numbers of germ cells were expressed as number of SG per/square unit of tubule surface and the ratios were compared between control and treatment samples.

#### Spermatozoa counts

Spermatozoa were counted from one epididymis as previously described^78^ using minimum 8 different animals for each condition.

### Antibodies

The following commercial antibodies were used: rat anti-GATA1 (sc-265), rabbit anti-DMC1 (sc-22768), goat anti-MIS (AMH) (sc-6886) and mouse SYCP3 (sc-74569) were obtained from Santa Cruz; rabbit anti-H3K4me3 (07–473), rabbit anti-H4K5ac (07–327) and rabbit anti-H3K27me3 (07–327) were obtained from Millipore; goat anti-PLZF (AF2944) was obtained from R&D systems; and rabbit anti-SYCP1(ab15087), anti-rabbit DDX4 (ab13840) and anti-rabbit ESR1 (ab32063) were obtained from Abcam. Fluorescent secondary Alexa antibodies were from Invitrogen.

### Meiotic surface spreads

The surface spreads were prepared in 5 independent experiments from F3 generation control or CD progeny as described in a previous study^79^. For synapsing defects analysis, images of at least 30 randomly chosen cells in the *pachytene* stage of prophase I of meiosis were taken for each sample. We used Chi-squared test to assess the statistical significance. The slides and images were independently reanalysed by two researchers.

For DNA repair efficiency analysis, surface spreads cells were co-immunostained against DMC1 and SYCP3 antibodies. Minimum 20 cells in pachytene stages were randomly taken with detectable DMC1 foci and counted. The data were averaged and presented as number of DMC1 foci per cell. We used t-test to assess the statistical significance.

### RNA extraction, reverse transcription

Total RNAs were prepared from adult or embryonic frozen testis tissue using TRIzol reagent (Life Technologies) or RNAeasy plus kit (Qiagen). The quantity of RNAs were estimated by nanodrop. Reverse transcription or total was performed using 700 ng (embryonic gonad) or 2 ug (adult testis) RNA using QuantiTect Reverse Transcription (Qiagen) according protocol provided by manufacture.

### RNA-seq library sequencing and data processing

A strand-specific library preparation protocol for RNA sequencing was performed using the sequencing technological platform at IGBMC, Strasbourg, France. We used three biological replicates for these experiments. The sequencing was performed in massive parallel sequencing paired-end mode, and the size of the sequencing tag was 50 bp.

Reads in FASTQ format were processed for quality control using the FastQC tool (http://www.bioinformatics.babraham.ac.uk/projects/fastqc/). To analyse differentially expressed genes, the quality-checked reads for each condition were processed using TopHat version 2.2.7^80^. The reads were mapped to the reference genome [*Mus musculus* Ensembl mm10 sequence], and the alignment files were generated as BAM files. These files were used as the input for Cufflinks, a complementary method used to generate assembled transcripts for each condition, and the abundance was evaluated using read data. The assembled transcripts were compared and annotated using Cuffmerge against the Ensembl (mm10) gene annotations^81^. These assemblies were used in Cuffquant and Cuffnorm in the Cufflinks 2.2.1 package to calculate expression levels. To identify transcripts that were differentially expressed between chlordecone- and control-derived samples, we first selected the cases for which we obtained values greater than the 50th centile of all values in at least one condition, and we then filtered the transcripts for which there was a more than a 2-fold difference between the CD− and control-derived samples. Finally, we used a statistical test implemented in the R package, Limma, with the false-discovery rate set at less than 10%^82^. RNA-Seq data were visualized using the Integrated Genomics Viewer (version 2.3.36)^83^.

### Chromatin immunoprecipitation and high-throughput sequencing

Chromatin immunoprecipitation in adult tissue was performed as previously described^84^. We used whole testis from one animal for each biological replicate.

For embryonic ChIP-qPCR, we pooled 30 testes from E15.5 of F1 foetuses. The protocol was similar to immunoprecipitation in adult tissue except that chromatin was not dialysed after sonication but instead diluted 6 times with ChIP-dilution buffer. We performed ChIP using antibody against ESR1. The immunoprecipitated DNA was purified, quantitated, and equal amount of ChIP and Input DNA were taken for qPCR. We used as negative control, the region of *Gapdh* gene located far from TSS. We normalized ESR1 chip data for *Gapdh* and found the enrichment as ChIP to input ratio for each target gene and for each sample. The Chip/Input difference was presented in Excel plot. We used Limma tests to assess the statistical significance.

An Illumina Hiseq. 2000 Genome Analyser was used to perform massively parallel 50-bp sequencing in Single-End mode. We sequenced two biological replicates per condition in multiplexing mode. The reads were demultiplexed and passed through quality control, at which point reads shorter than 50 nucleotides were removed. FastQ files were generated using a genomic platform in Strasbourg, France.

### Peak calling and identification of differential peaks

Approximately 115 million tags were derived from the anti-H3K4me3 ChIP. The resulting sequences were mapped back to the mouse mm10/Ensembl genome using Bowtie 2.2.7 with a seed length of 20. Only tags that passed the quality filter and mapped uniquely to the genome were used. ChIP enrichment was further verified using CHANCE^85^. H3K4me3 mark peaks were identified using MACS 2.1.1^86^ with two biological replicate samples, including the corresponding input, using a shift-size window of 73 bp, no model, and a *p*-value threshold <10E-5. The number of mapped reads was multiplied by a scale factor to normalize the total number of reads in different samples. To compare the H3K4me3 ChIP datasets of the CD-treated and control samples, differential peak calling was performed using the following steps. First, from all of the peaks called using the protocol described above, we retained only those peaks with average values above the 50th quintile. Second, we selected peaks with fold changes above 1.5 or 2 and FDR < 10%. Statistical significance was calculated using a Limma test^82^. The annotation of significantly differential peaks was performed using CEAS^87^.

The results were visualized using the Integrative genomics viewer IGV version 2.4.2^83^.

### Gene Ontology (GO) term analyses

Gene Ontology (GO) term analyses were performed using DAVID v6.7 with default parameters^88^.

### Functional annotation of ChIP-seq data

Functional annotation was performed using GREAT version 3.0.0^89^ or EnrichR^90,91^ with default parameters.

We used ChEA to functionally annotate differential peaks. ChEA is a database of genes potentially regulated by specific transcription factors, where the data were extracted manually from different ChIP-seq experiments for each transcription factor^92^. We used for the analysis the recently updated version of ChEA 2016 provided by EnrichR.

### FIMO motif search

FIMO (Find Individual Motif Occurrences) with the sequences of differential peaks was used to scan for ESR1 motif-binding sites^45^.

### QPCR

QPCR was performed as previously described^14^. Primers used for ChIP-qPCR and RT-QPCR are indicated in the Supplementary Table 17.

### Data access

All sequencing data from this study are publicly available and have been deposited in the National Centre for Biotechnology Information Gene Expression Omnibus. The following samples from ChIP-seq data were deposited at GEO: GSE number GSE86440.

## Electronic supplementary material

Supplementary information
