# Supplementary material for: Gestational exposure to chlordecone promotes transgenerational changes in the murine reproductive system of males
Source: Sci Rep. 2018 Jul 6;8:10274. doi: 10.1038/s41598-018-28670-w (PMC6035262; doi:10.1038/s41598-018-28670-w)
Supplement: Supplementary file 1 — Supplementary information [file 41598_2018_28670_MOESM1_ESM.pdf]

Gestational exposure to chlordecone promotes transgenerational changes in the murine reproductive system

Aurore Gely-Pernot[1]*, Chunxiang Hao[2*], Louis Legoff[1*], Luc Multigner[1], Shereen Cynthia D'Cruz[1], Christine Kervarrec[1], Bernard Jégou[1], Sergei Tevosian[3] and Fatima Smagulova[1]#

Supplementary information

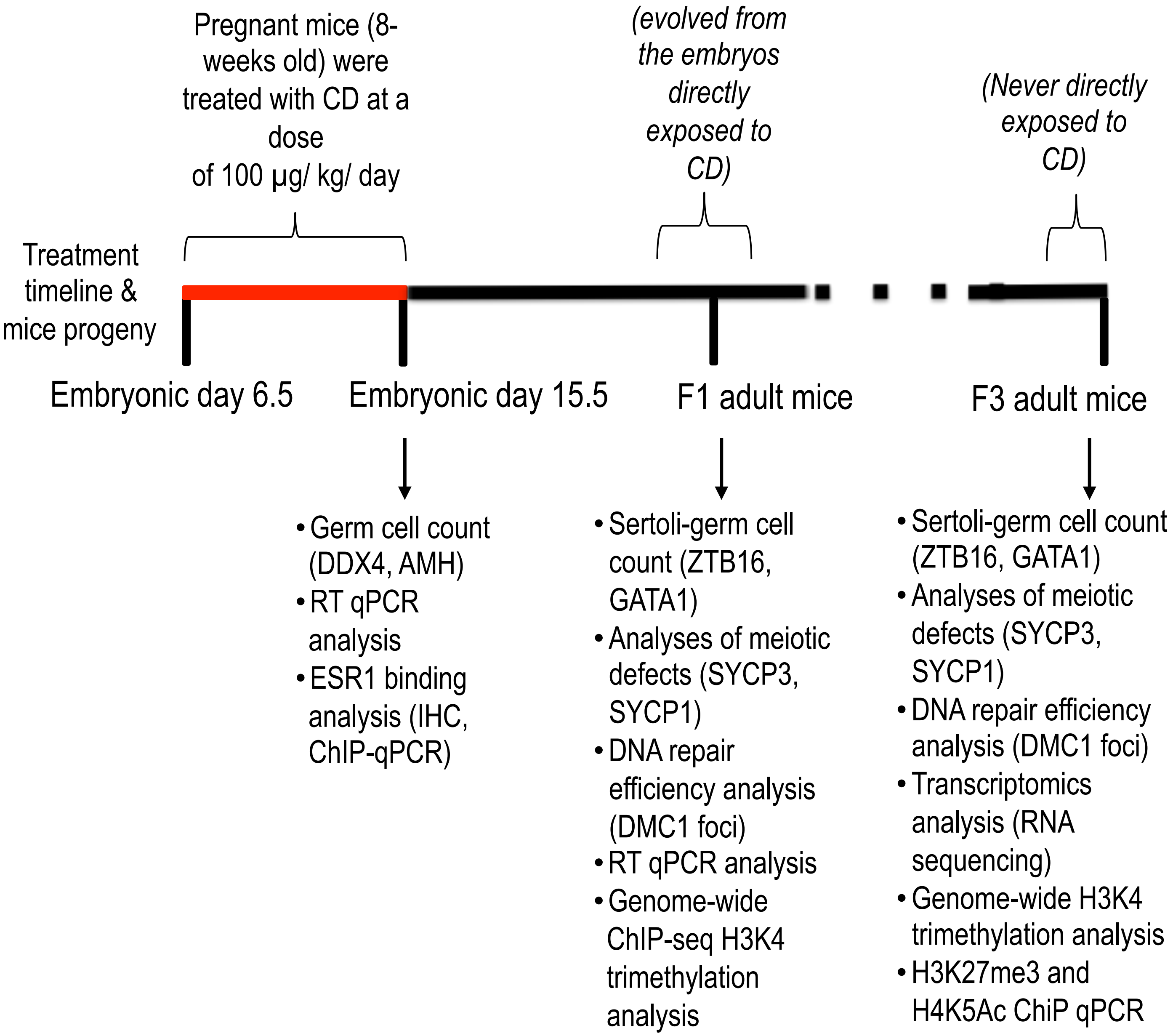

Supplementary Figure 1. A schematic diagram representing the treatment timeline and the experimental analyses carried out in mouse progenies.

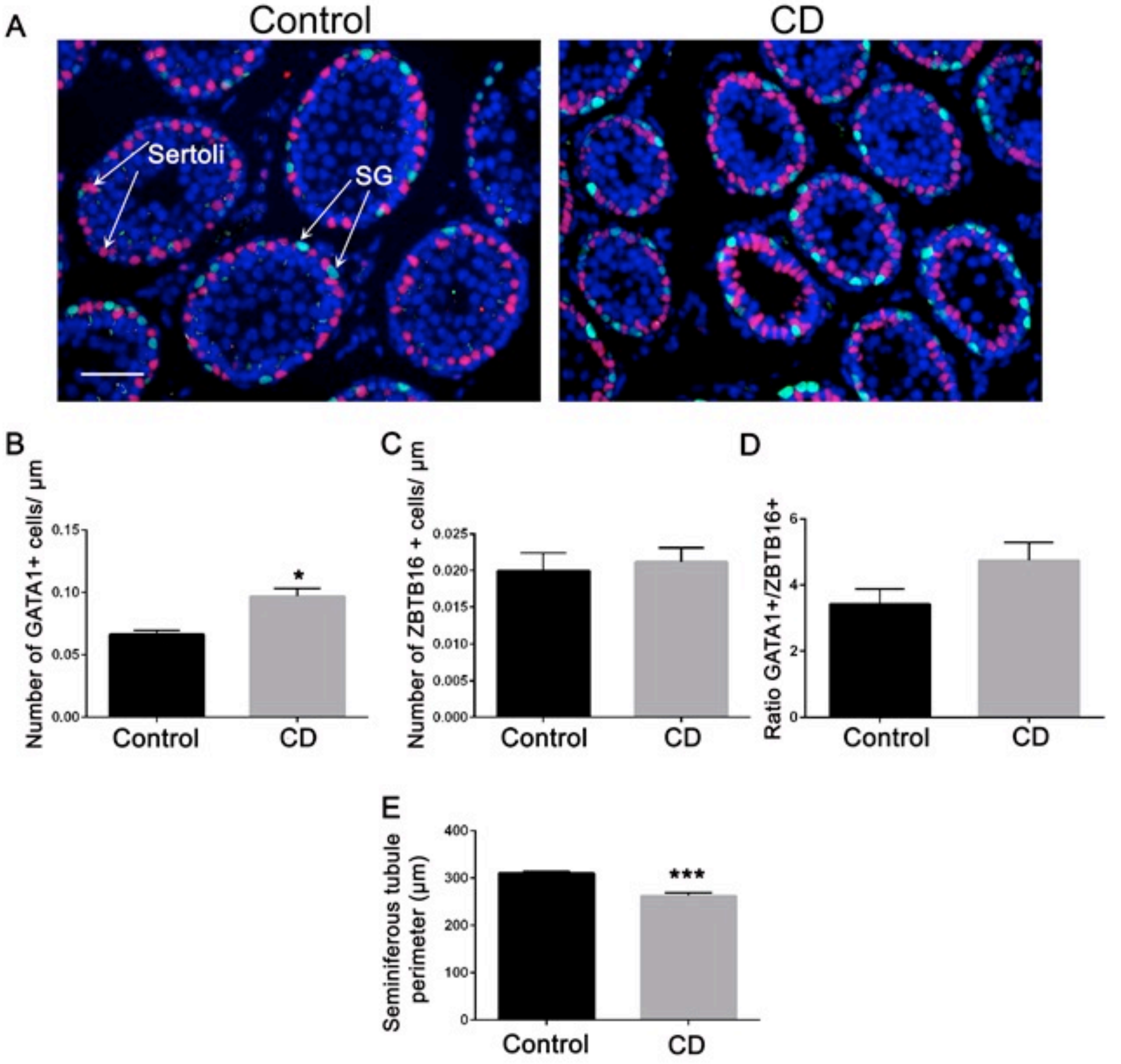

Supplementary Figure 2. Gestational exposure to CD does not affect SG numbers but increases the Sertoli cells in 17 day old F1 males.

Representative images of testes sections from the control (left panel) and CD lineage (right panel) animals: Sertoli and SG cells were immunostained using anti-GATA1 (red) or anti-ZBTB16 (green) antibodies, respectively. A quantitative analysis of (B) Sertoli cells and (C) SG. The contour of each tubule section was measured using ImageJ. The values shown indicate the cell counts per micrometre of tubule circumference. (D) The ratio of the number of Sertoli cells per SG is also indicated (Sertoli p=0.0137, SG p= 0.7150, n=3 control, n=5 CD, t-test, scale bar 50 µm). (E) is Seminiferous tubule perimeter.

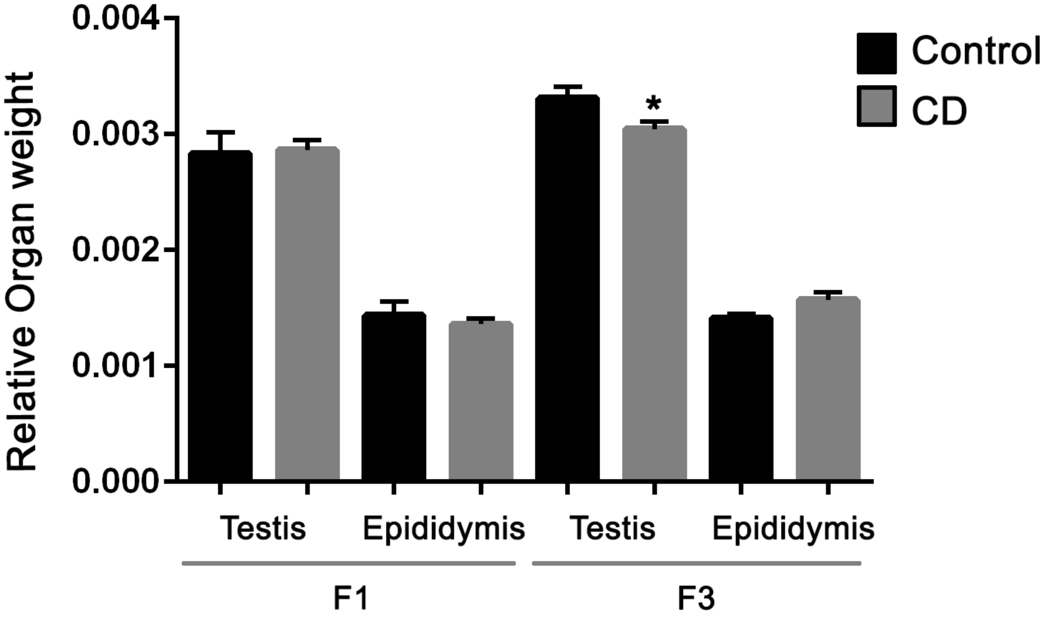

Supplementary Figure 3. Relative weight of testis and epididymis normalised to body weight in control and treated samples in F1 and F3. We measured the reproductive organ weight and found that it does not change in F1 (n=5 for control and n=7 for chlordecone). In F3, the testis weight has decreased (p=0.0029, n=8, control and n=10 , CD, t-test);

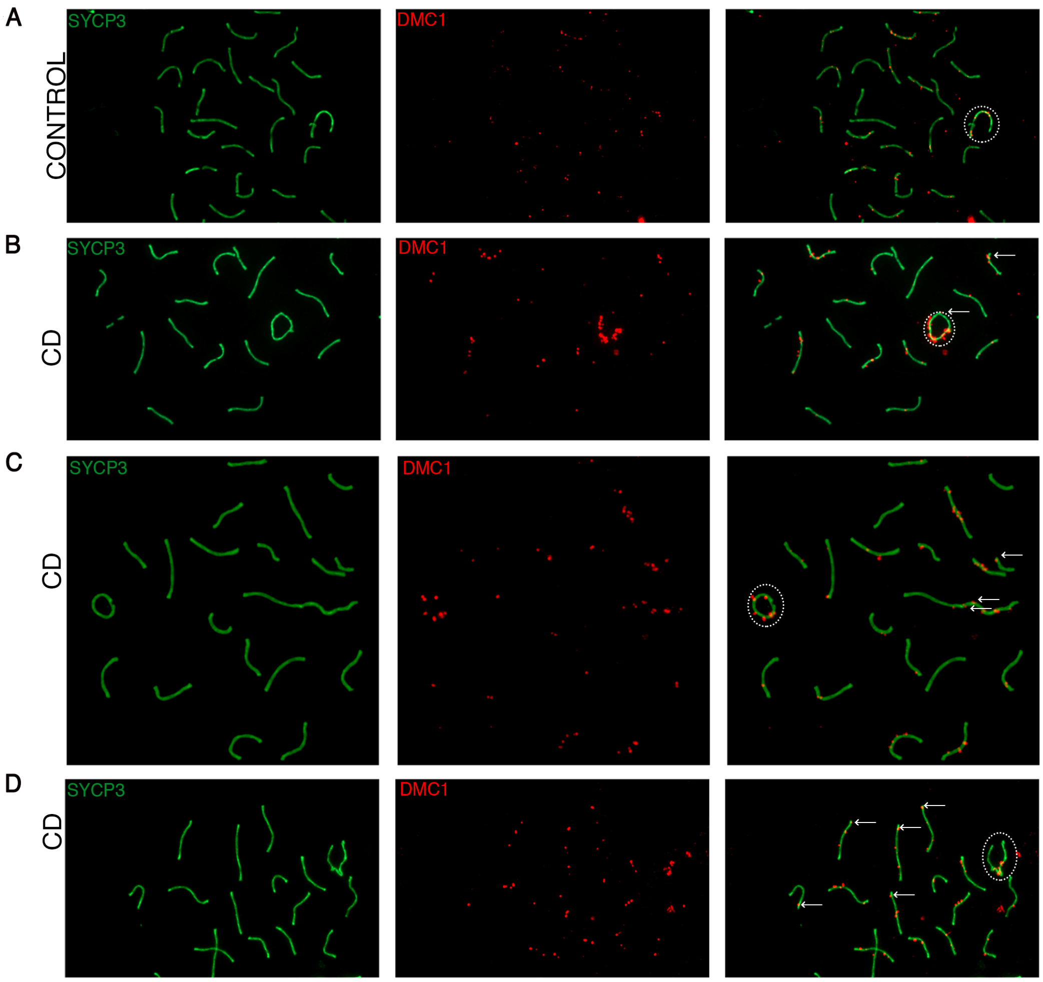

E

sample	DMC1 foci+/-SEM	p-value
Cont F1	12.68+/−2.1	
CD F1	52.13+/−15.7	0.06

Cont F3	14.54+/−3.6	
CD F3	40.73+/−3.4	0.0004

Supplementary Figure 4. Gestational exposure to CD increases DMC1 in F3 CD-derived males.
Surface spreads were prepared from F3-generation testes obtained from control or CD-derived mice. The spreads were immunostained for a major protein of the lateral element of the synaptonemal complex (SC), SYCP3 (green), and DMC1. In the control (A). DMC1 foci are nearly absent in pachytene-stage cells, and only a few foci are detectable. In CD-derived males, the cells with rings are sex chromosomes (B.C), which strongly stained for DMC1. (D) A number of chromosomes had subtelomeric DMC1 foci. The sex chromosomes are outlined with a white oval. The subtelomeric DMC1 foci are indicated by arrows. (E) Quantitative analysis of average DMC1 foci per cell. F1: n=3, control and CD, F3, n=5 control, n= 4, CD, p-values indicated in tables, t-test

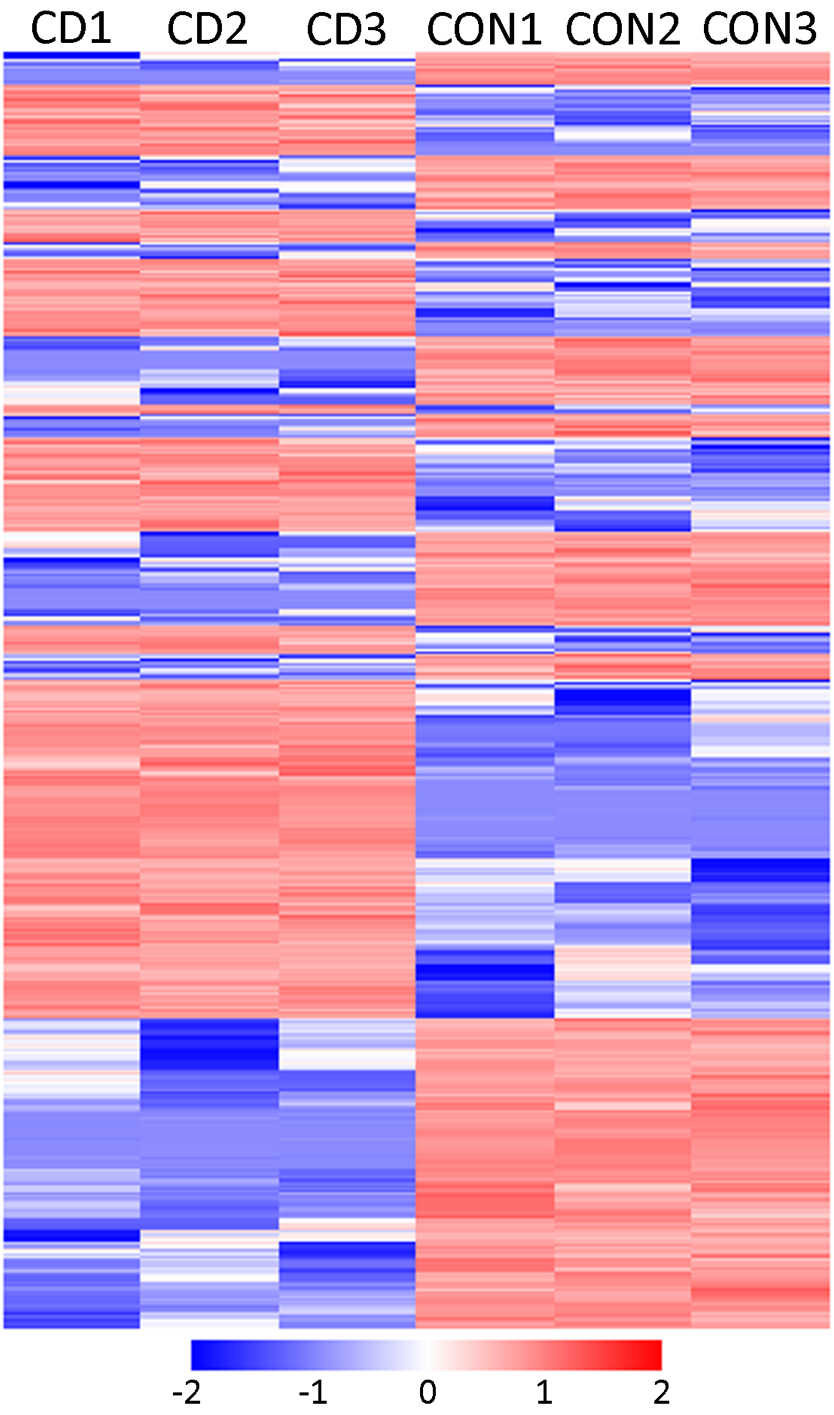

Supplementary Figure 5. Heatmap of all differentially expressed transcripts in F3.

The log2 FC of all differentially expressed transcripts (FC>2, FDR<0.1) were plotted in R and presented as heatmap. Con1-3 are control samples, CD1-3 , CD-derived samples

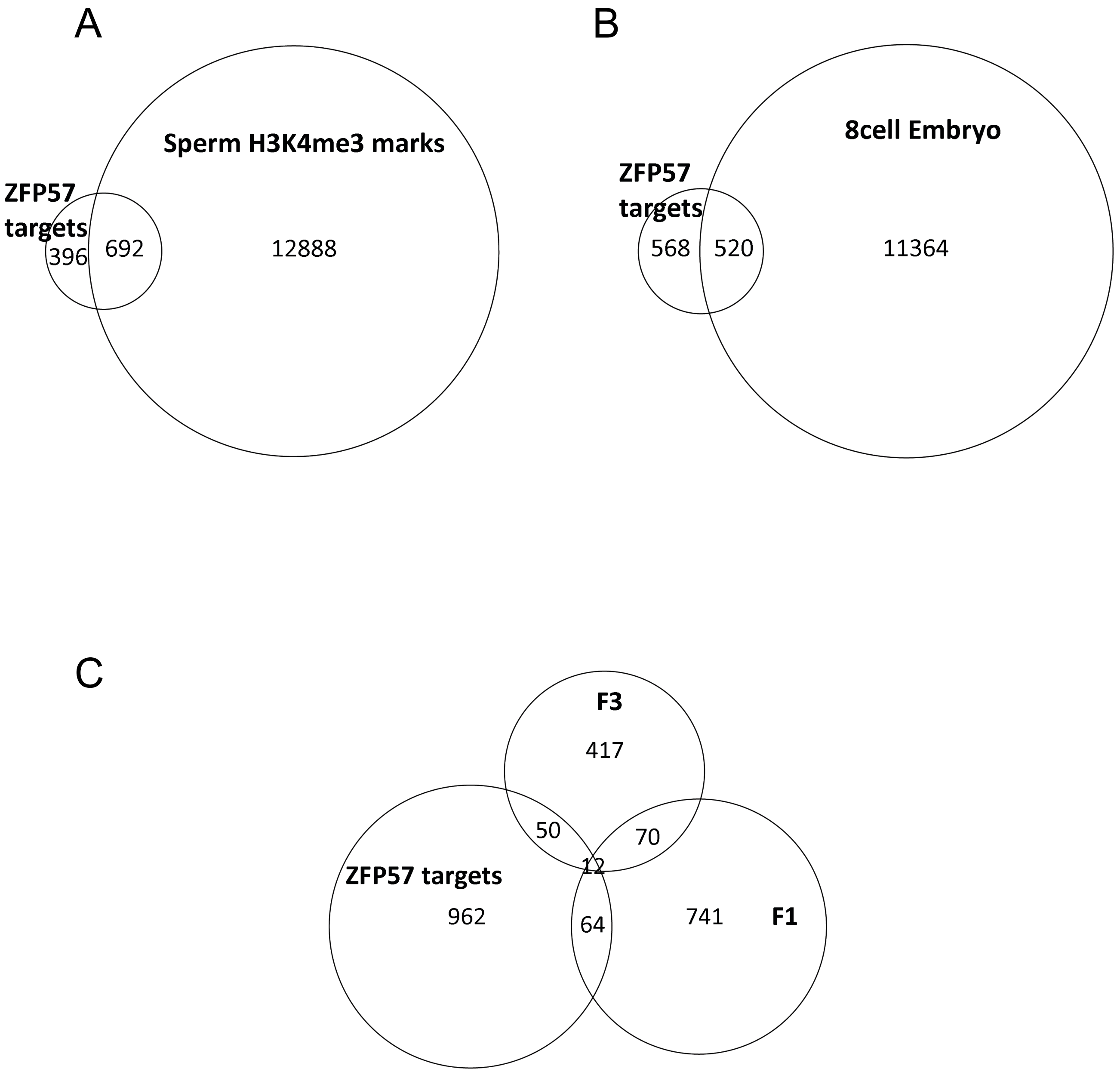

Supplementary Figure 6. Venn diagrams of ZFP57 targets obtained from previously described public datasets shows the preservation of paternal histones in sperm and in early 8-cell embryos.

(A) Venn diagram shows that 63.6% of ZFP57 target genes preserved their histones in sperm. (B) 47.8% of all target genes of ZFP57 preserves paternal H3K4me3 histone in 8-cell embryo. (C) 8.6% and 11.3% of genes are ZFP57 targets in F1 and F3, respectively.

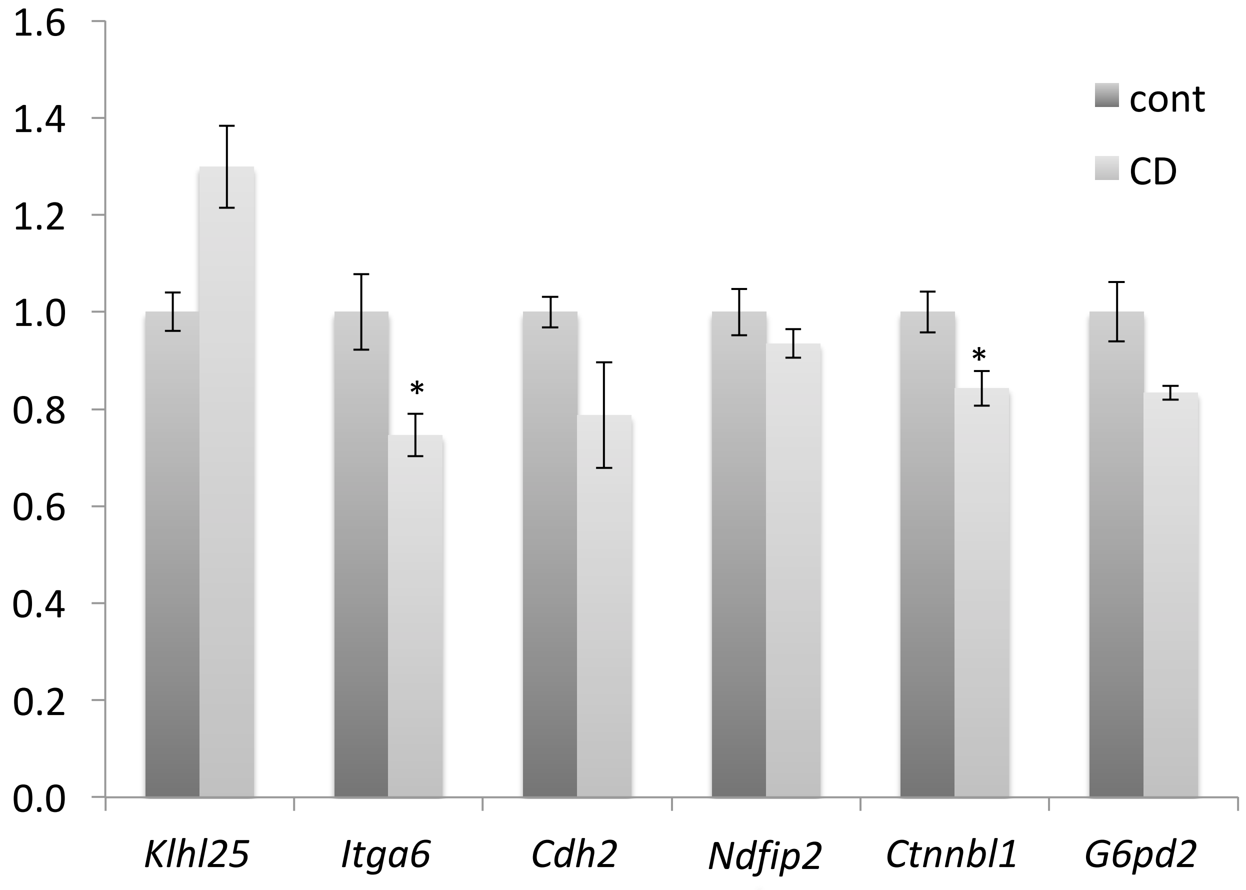

H3k4me3 in F1	+	+	-	-	-	-

Supplementary Figure 7. The expression of ZFP57 target genes in E15.5 testis have been altered.

RT-qPCR was performed as described in the methods section. Copy numbers for each gene were normalised to *Hprt* gene and values were averaged and plotted as normalized expression compared to control +/- SEM, *p<0.05, t-test, n=4 for cont. and CD.

H3K4me3 in F1 and F3 near *Prdm1*

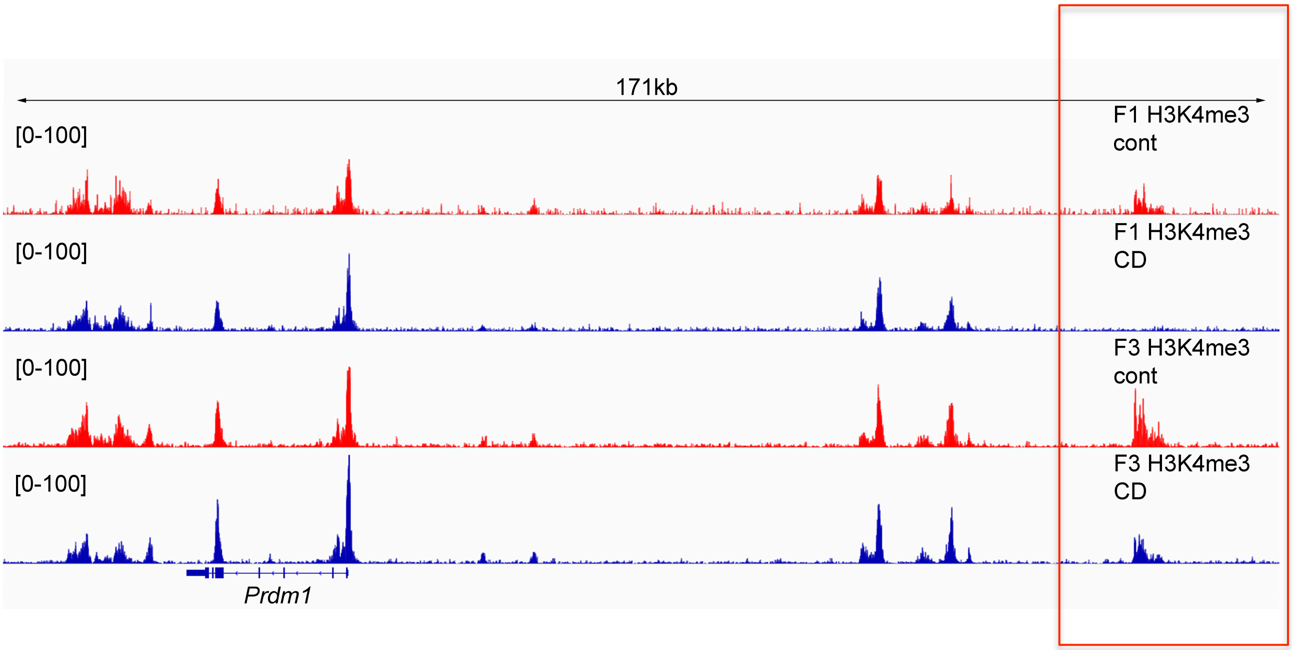

Supplementary Figure 8. Gestational exposure to CD causes decrease in H3K4me3 occupancy near *Prdm1* gene in F1 adult males. The differential peaks were annotated by GREAT, which assigned the region with altered peaks to *Prdm1.* The altered peaks are shown in rectangle. In F1, the H3K4me3 peaks have decreased significantly (p-value=0.01), but not in F3 (p-value=0.67). Two biological replicates for F1 and F3 were fused for simplicity; control peaks are marked by red and CD peaks are shown in blue.

F1

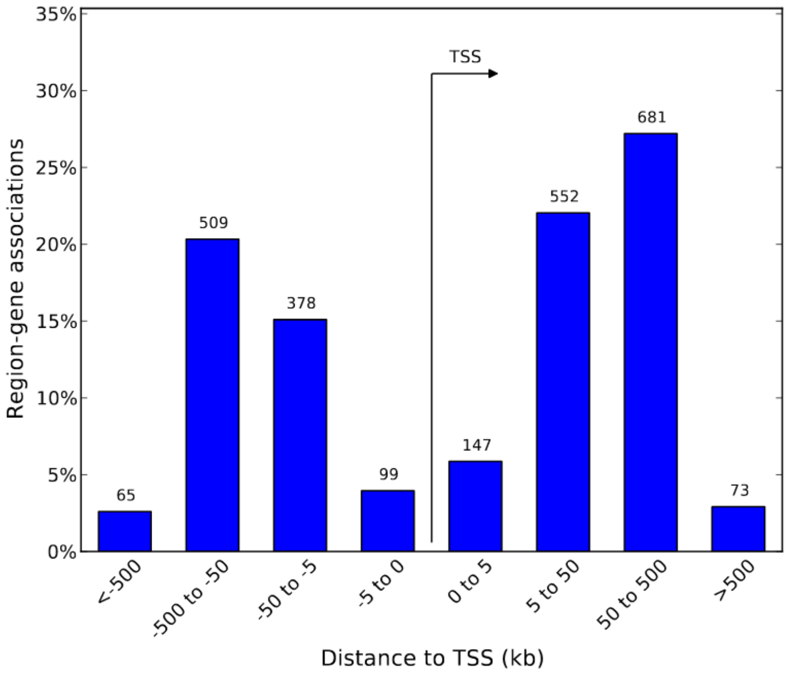

F3

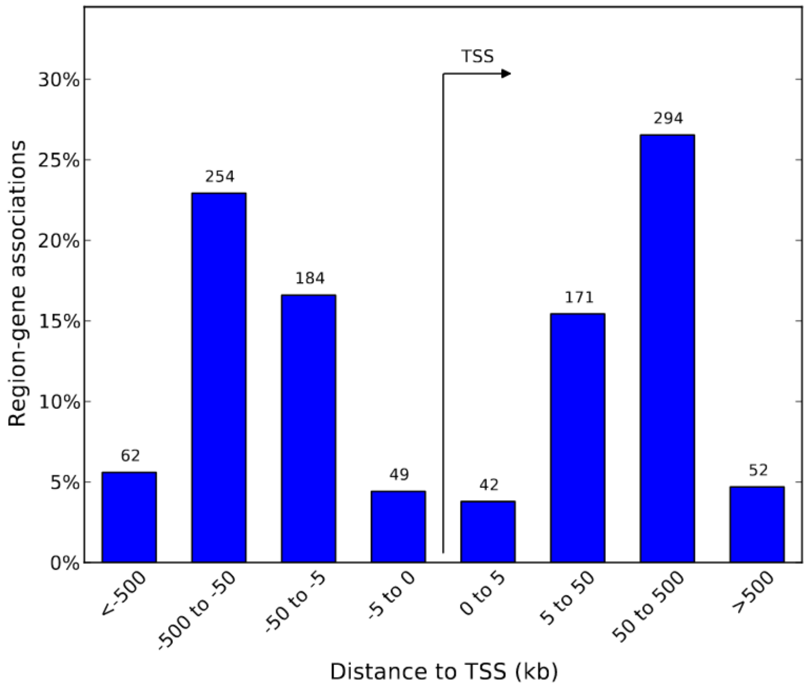

Supplementary Figure 9. The altered peak profiles of F1 and F3 have a similar pattern of the distance to TSS.

The distance to TSS was calculated by GREAT. Most of differential peaks in F1 and F3 are located far from TSS (between 50 to 500kb).

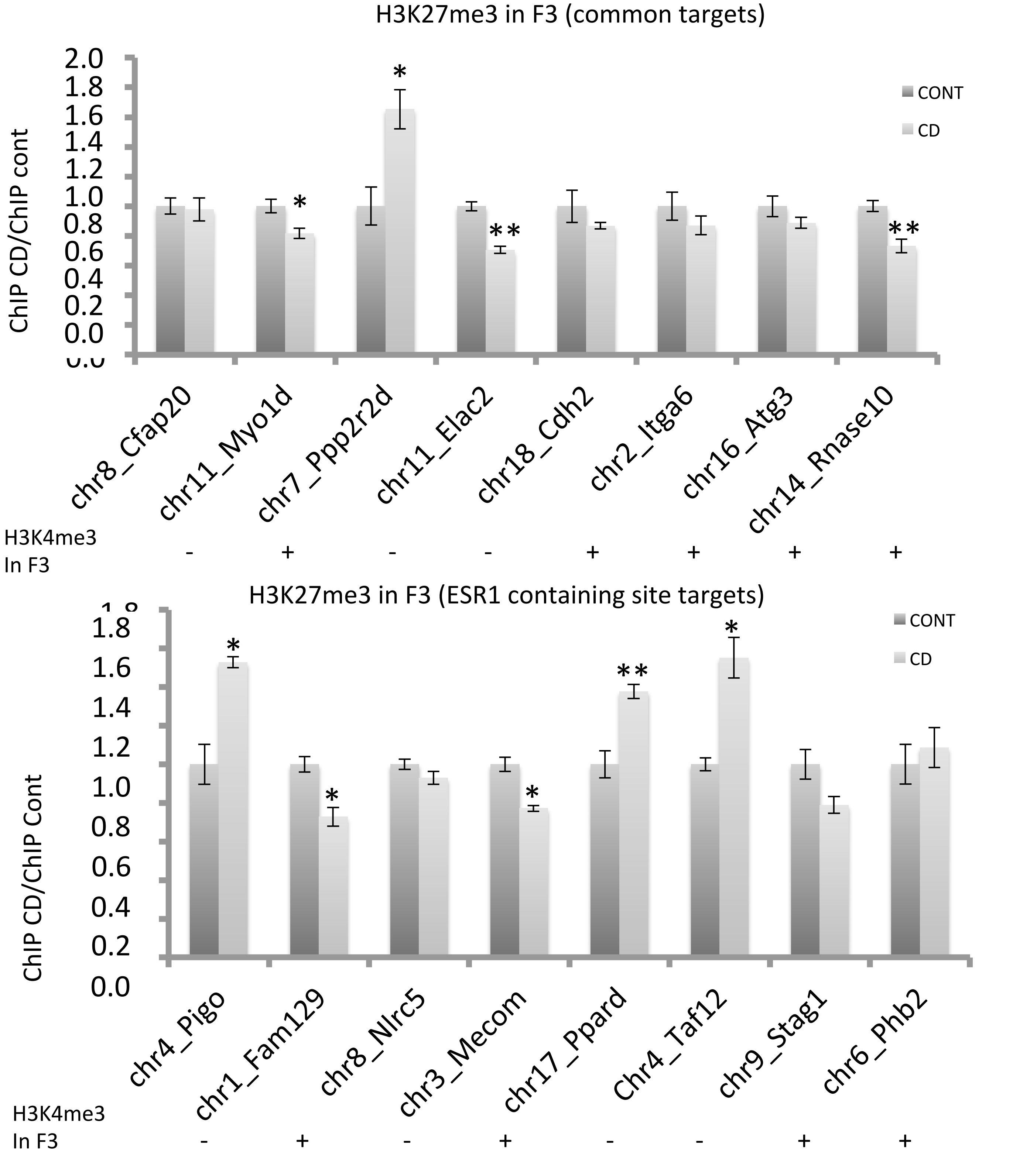

Supplementary Figure 10. H3K27me3 marks were altered at some differential H3K4me3 peaks. ChIP was performed as described in the Method section. Equal amount of ChIP and input DNAs were taken for qPCR. Each value for target gene was normalized for *Gapdh* region located far from promoter (background). The data were averaged and presented as normalized ChIP in CD samples compared to ChIP in control. H3K4me3 from F3 data are presented as "–" for decreased and "+" for increased peaks, * $p<0.05$, ** $p<0.01$, n=4 for cont and 4= CD, t-test.

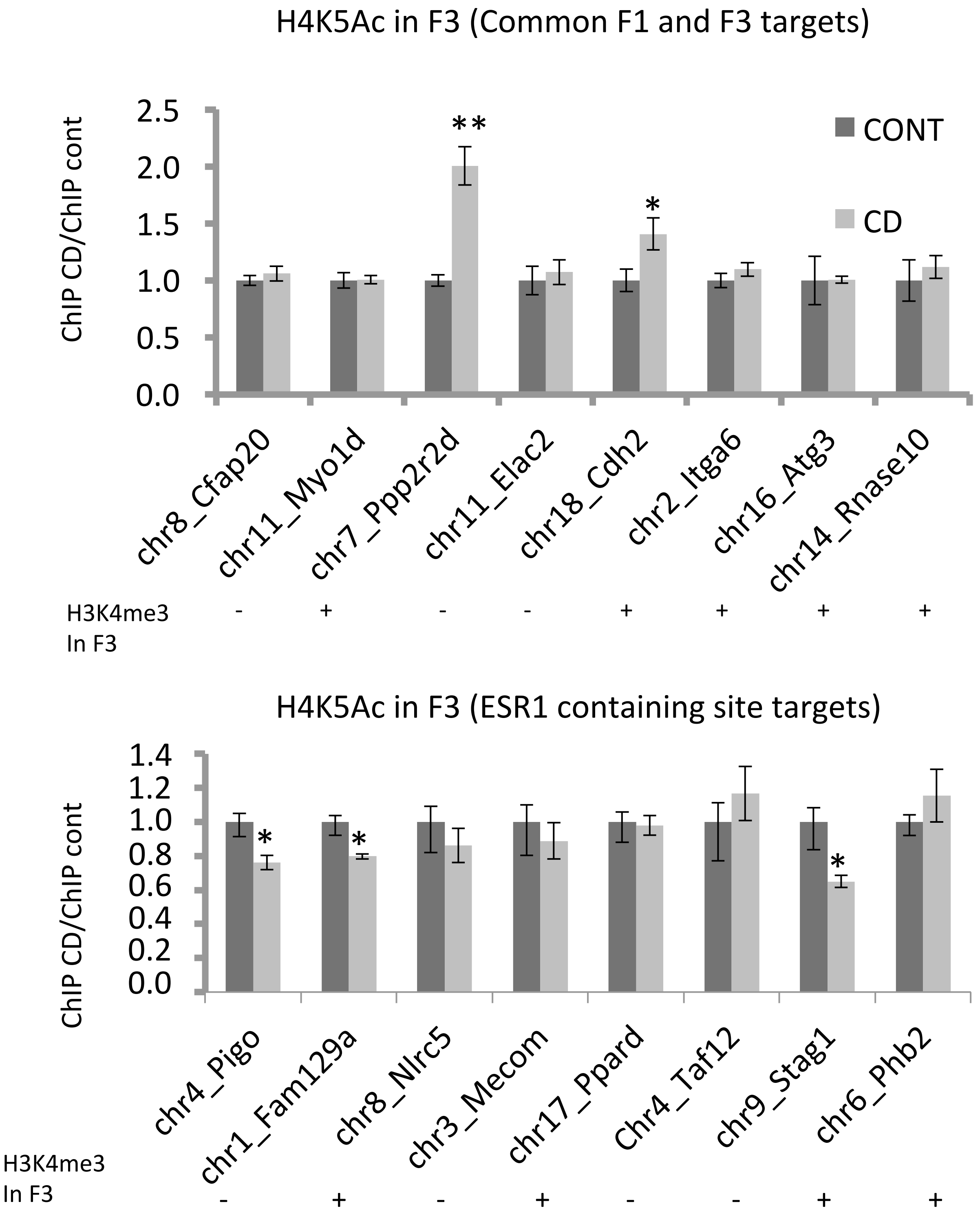

Supplementary Figure 11. H4K5Ac marks were altered at some differential H3K4me3 peaks.

ChIP was performed as described in the Method section. Equal amount of ChIP and input DNAs were taken for qPCR. Each value for target gene was normalized for *Gapdh* region located far from promoter (background). The data were averaged and presented as normalized ChIP in CD samples compared to ChIP in control control. H3K4me3 from F3 data are presented as "–" for decreased and "+" for increased peaks, * p<0.05, ** p<0.01, n=4 for cont and for CD, t-test.

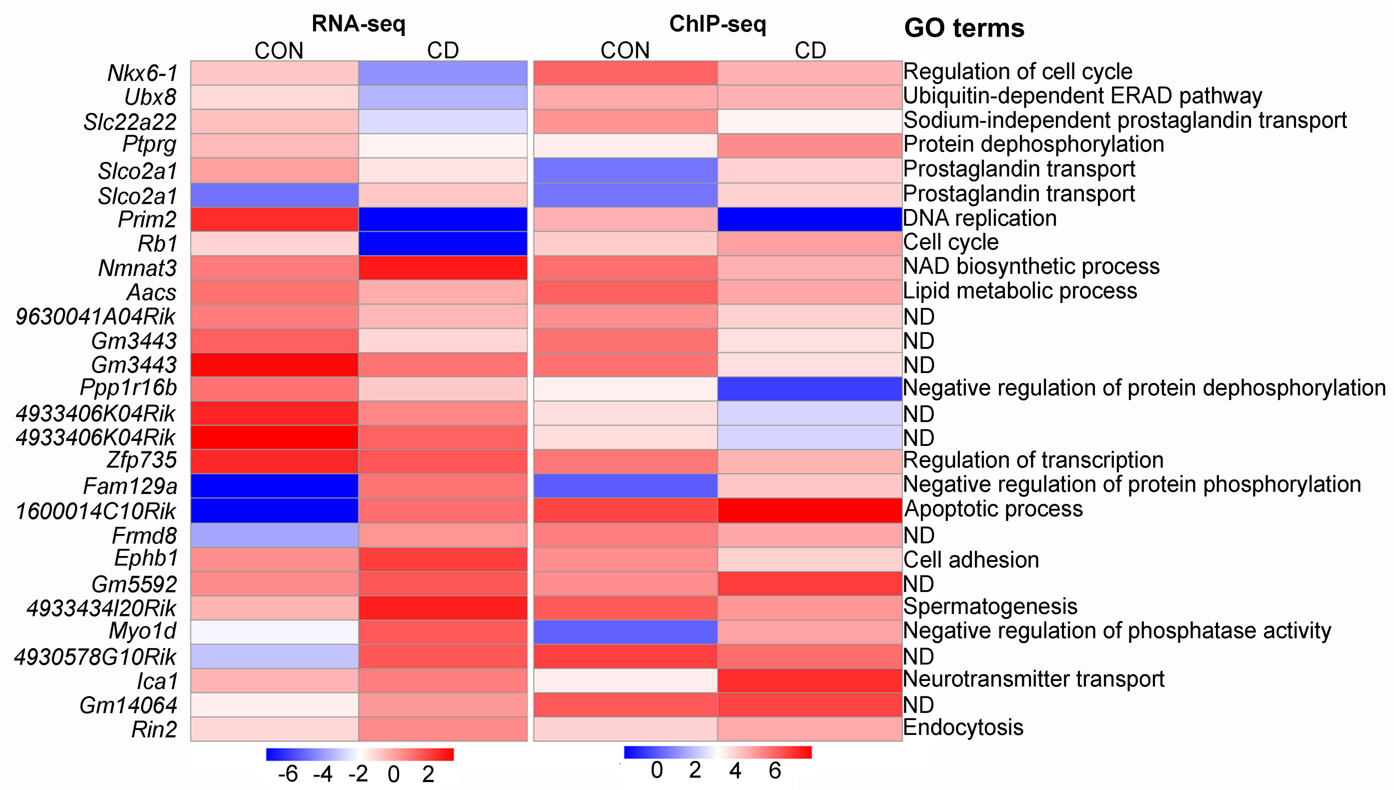

Supplementary Figure 12. The subset of altered genes in CD-derived F3 males have altered peaks nearby them.

The FPKM values for RNA-seq (FC>2, FDR<0.1) and ChIP-seq data of biological replicates for control or CD (FC>2, FDR<0.1) were averaged and plotted in R independently as a heatmaps.

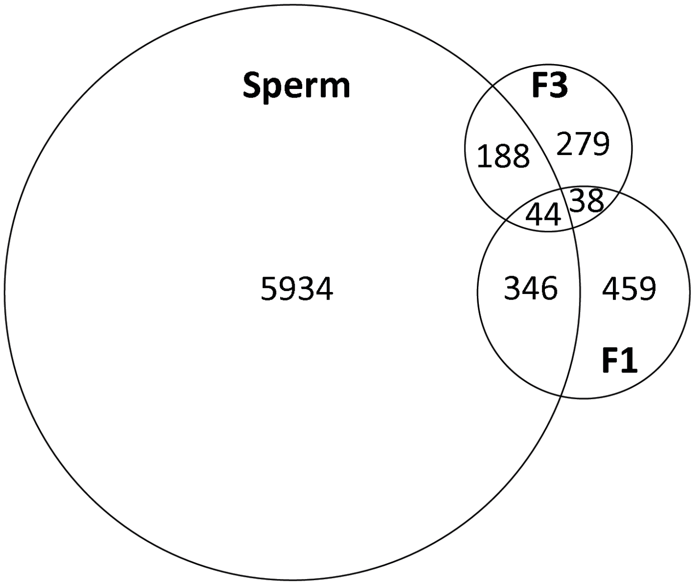

Supplementary Figure 13. The Venn diagram of overlap of genes retaining H3K4me3 in human sperm and genes located in differential H3K4me3 in F1 and F3.

The sperm data were obtained from the published dataset (Samans et al, 2014). The comparison of data shows that about 40% of genes located nearby differential peaks in F1 and F3 retain H3K4me3 marks in sperm.

Supplementary Table 1. Up-regulated transcripts in F3 (RNA-seq)

chr	start	end	strand	class	gene	gene biotype	length	log2(FC)	p-value	adj p-value
chr1	33745484	33757794	-	j	*Bag2*	protein_coding	1771	2.95	1.86E-04	3.01E-02
chr1	55131282	55152999	+	=	*Mob4*	protein_coding	745	1.15	5.35E-03	9.58E-02
chr1	67792450	67821976	+	j	*Gm15669*	lincRNA	1011	6.16	9.28E-04	5.72E-02
chr1	67792481	67805324	+	=	*Gm15669*	lincRNA	526	7.45	9.80E-05	2.44E-02
chr1	74600532	74636894	+	j	*Stk36*	protein_coding	5579	6.30	2.61E-03	8.37E-02
chr1	151677167	151719342	+	=	*Fam129a*	processed_transcript	3245	8.57	3.18E-04	3.68E-02
chr1	157497813	157510162	-	j	*Gm15486*	antisense	787	1.96	6.35E-03	9.96E-02
chr10	33512286	33550919	-	j	*Clvs2*	protein_coding	1542	4.19	6.07E-03	9.84E-02
chr10	79540245	79555199	-	=	*Mier2*	protein_coding	2676	1.20	3.25E-04	3.68E-02
chr10	79804636	79820907	+	j	*Palm*	protein_coding	2770	2.11	3.59E-03	8.82E-02
chr10	91119775	91124022	-	=	*Slc25a3*	retained_intron	4039	1.59	2.79E-03	8.47E-02
chr10	116964707	116967396	+	=	*D630029K05Rik*	lincRNA	457	2.30	1.57E-03	6.76E-02
chr10	121598955	121624796	-	j	*Xpot*	protein_coding	3031	2.57	1.12E-03	6.09E-02
chr10	122805785	122945795	+	j	*Ppm1h*	protein_coding	5968	1.73	6.35E-03	9.96E-02
chr11	5100157	5106093	+	j	*Rhbdd3*	protein_coding	1617	4.27	4.67E-03	9.22E-02
chr11	38360874	38544022	-	j	*Gm12130*	lincRNA	810	1.54	4.41E-03	9.12E-02
chr11	51813451	51857125	-	j	*Phf15*	protein_coding	6028	3.38	5.78E-03	9.81E-02
chr11	58792811	58801960	-	=	*Fam183b*	protein_coding	493	2.20	6.15E-05	2.06E-02
chr11	60409215	60417036	-	=	*Atpaf2*	retained_intron	522	2.36	4.53E-03	9.22E-02
chr11	62605767	62607023	+	=	*4930443B20Rik*	antisense	446	1.51	3.86E-03	9.00E-02
chr11	62761236	62765554	+	=	*Gm12283*	antisense	713	1.79	2.20E-04	3.30E-02
chr11	69601358	69603613	-	=	*Atp1b2*	processed_transcript	711	5.60	5.01E-03	9.51E-02
chr11	71019611	71027371	+	=	*Mis12*	protein_coding	2721	1.19	2.69E-03	8.41E-02
chr11	74383356	74400141	-	j	*Rap1gap2*	protein_coding	5361	1.49	5.04E-03	9.52E-02
chr11	80482126	80766579	-	j	*Myo1d*	protein_coding	5271	3.81	6.22E-05	2.06E-02
chr11	87109251	87124634	+	=	*Ska2*	processed_transcript	861	3.44	5.14E-03	9.58E-02
chr11	98082551	98128963	-	j	*Fbxl20*	protein_coding	9846	5.47	4.63E-03	9.22E-02
chr11	98082556	98149612	-	=	*Fbxl20*	protein_coding	8601	6.74	3.84E-03	9.00E-02
chr11	102402985	102407544	-	j	*Slc25a39*	protein_coding	1470	1.74	5.93E-03	9.81E-02
chr11	102887336	102900912	-	j	*Gfap*	protein_coding	2328	5.97	5.44E-03	9.64E-02
chr11	110176820	110251776	-	=	*Abca6*	protein_coding	5240	1.40	3.29E-03	8.74E-02
chr11	110286819	110337622	-	=	*Abca5*	protein_coding	3829	1.13	1.31E-03	6.42E-02
chr11	120708858	120709445	+	=	*Aspscr1*	retained_intron	491	2.32	1.20E-03	6.27E-02
chr12	81573696	81574746	-	=	*Med6*	retained_intron	487	4.35	6.58E-05	2.06E-02
chr12	100199529	100203937	+	=	*Calm1*	retained_intron	582	1.44	6.00E-03	9.81E-02
chr12	108825873	108832534	-	j	*Slc25a29*	protein_coding	1768	1.71	3.91E-03	9.00E-02
chr12	113098278	113137206	+	j	*Mta1*	protein_coding	2692	2.41	7.71E-04	5.24E-02
chr13	3478243	3488058	+	j	*2810429I04Rik*	lincRNA	3213	2.80	1.54E-03	6.76E-02
chr13	34978702	34988822	-	=	*Eci2*	processed_transcript	943	1.32	5.29E-03	9.58E-02
chr13	51838107	51848468	+	j	*Gadd45g*	protein_coding	2003	1.16	6.28E-03	9.95E-02
chr13	54789463	54796187	+	=	*Tspan17*	protein_coding	712	3.52	2.34E-03	7.98E-02
chr13	98489348	98492001	-	=	*Gm10320*	protein_coding	527	5.16	1.17E-04	2.58E-02
chr13	114818286	114828731	+	=	*Mocs2*	protein_coding	962	3.21	3.52E-03	8.76E-02
chr14	3188255	3196667	+	=	*Gm5795*	protein_coding	984	6.45	3.01E-05	1.83E-02
chr14	3188313	3196681	+	=	*Gm5795*	protein_coding	1758	2.50	1.76E-03	6.97E-02
chr14	3251194	3262297	-	=	*Gm17188*	processed_transcript	2078	4.11	3.26E-04	3.68E-02
chr14	3873855	3884074	-	=	*Gm26886*	processed_transcript	1293	7.52	2.02E-03	7.35E-02
chr14	3931819	3934803	+	=	*Gm3029*	protein_coding	618	2.62	3.26E-03	8.74E-02
chr14	4165088	4173503	+	=	*Gm3127*	protein_coding	1758	2.97	2.25E-04	3.31E-02
chr14	4664192	4670850	+	=	*Gm3239*	protein_coding	1751	3.24	2.46E-03	8.21E-02
chr14	4709238	4717664	+	=	*Gm7876*	protein_coding	984	4.41	5.19E-03	9.58E-02
chr14	4983994	4992409	+	=	*Gm8297*	protein_coding	1759	2.26	4.43E-03	9.13E-02
chr14	5131519	5139959	+	=	*Gm8279*	protein_coding	998	5.61	6.18E-03	9.92E-02
chr14	5501674	5522927	+	=	*Gm3488*	protein_coding	1945	4.18	2.89E-03	8.54E-02
chr14	6296381	6304843	-	=	*Gm21103*	protein_coding	984	1.76	3.72E-04	3.73E-02
chr14	6314876	6343678	-	=	*Gm3618*	protein_coding	384	3.12	1.26E-04	2.58E-02
chr14	6613967	6622418	-	=	*Gm3460*	protein_coding	1809	2.52	1.89E-04	3.01E-02
chr14	6765963	6774411	-	=	*Gm8362*	protein_coding	997	3.55	5.06E-04	4.40E-02
chr14	7323988	7332395	-	=	*Gm5797*	protein_coding	997	3.65	7.33E-05	2.13E-02

Supplementary Table 1. Up-regulated transcripts in F3 (RNA-seq) - *(Continued)*

chr	start	end	strand	class	gene	gene biotype	length	log2(FC)	p-value	adj p-value
chr14	7498713	7506148	-	=	*Gm3760*	unprocessed_pseudogene	804	1.81	1.71E-05	1.38E-02
chr14	60732906	60764549	+	=	*Spata13*	protein_coding	5469	2.71	6.17E-04	4.65E-02
chr14	63436394	63465498	+	j	*Fam167a*	protein_coding	1655	1.54	5.60E-03	9.76E-02
chr14	79515674	79582475	+	=	*Elf1*	protein_coding	3869	2.28	3.74E-03	8.93E-02
chr15	9684842	9748820	-	=	*Spef2*	protein_coding	1917	3.75	2.57E-03	8.32E-02
chr15	79561500	79605135	-	j	*Dmc1*	protein_coding	2350	1.23	3.34E-03	8.74E-02
chr15	85885164	85896426	+	=	*Trmu*	nonsense_mediated_decay	665	2.17	3.67E-03	8.87E-02
chr15	89100717	89102014	-	=	*Tubgcp6*	nonsense_mediated_decay	759	1.58	5.34E-03	9.58E-02
chr15	90487482	90524845	-	j	*Cpne8*	protein_coding	2297	1.75	9.13E-04	5.72E-02
chr15	102518455	102521749	+	=	*Tarbp2*	protein_coding	370	5.90	4.49E-03	9.19E-02
chr16	34013146	34096028	-	=	*Kalrn*	protein_coding	2693	2.83	5.89E-03	9.81E-02
chr16	89140944	89141099	+	=	*Gm6358*	protein_coding	156	1.25	2.83E-03	8.54E-02
chr16	90925809	90934927	-	j	*1110004E09Rik*	protein_coding	1435	1.15	2.72E-04	3.54E-02
chr17	15705625	15756087	+	j	*Chd1*	protein_coding	4055	5.39	3.82E-04	3.73E-02
chr17	35821684	35823000	+	j	*Ier3*	protein_coding	1088	6.87	1.13E-03	6.09E-02
chr17	42792948	42876522	-	j	*Cd2ap*	protein_coding	5464	1.35	1.26E-03	6.41E-02
chr17	48389224	48393459	-	=	*Nfya*	protein_coding	701	2.90	1.50E-03	6.71E-02
chr17	79626632	79682152	+	j	*Rmdn2*	protein_coding	2336	1.90	2.98E-03	8.65E-02
chr17	87254658	87265935	-	j	*Mcfd2*	protein_coding	1866	1.20	5.11E-04	4.40E-02
chr17	88626555	88645724	+	=	*Ston1*	protein_coding	2983	1.17	3.22E-03	8.74E-02
chr18	12189693	12236400	-	=	*Npc1*	protein_coding	5224	1.04	6.43E-04	4.80E-02
chr18	13687013	13733283	-	j	*Zfp521*	protein_coding	2637	2.53	4.20E-03	9.11E-02
chr18	17019051	17137559	+	=	*1700001G01Rik*	lincRNA	630	11.23	5.80E-04	4.56E-02
chr18	17019076	17047568	+	=	*1700001G01Rik*	lincRNA	928	2.99	4.70E-04	4.28E-02
chr18	17035508	17137559	+	j	*1700001G01Rik*	lincRNA	2097	2.67	5.16E-05	2.03E-02
chr18	17035508	17137559	+	j	*1700001G01Rik*	lincRNA	1249	3.02	1.44E-03	6.56E-02
chr18	76978461	77006872	-	=	*Katnal2*	processed_transcript	792	4.00	3.50E-03	8.74E-02
chr19	5848744	5875274	-	j	*Frmd8*	protein_coding	3288	4.26	1.03E-03	5.87E-02
chr19	10668953	10671458	-	j	*Pga5*	protein_coding	869	2.83	2.07E-03	7.43E-02
chr19	10668953	10670976	-	j	*Pga5*	protein_coding	648	3.02	1.16E-03	6.20E-02
chr19	10668953	10670976	-	j	*Pga5*	protein_coding	721	3.48	1.41E-03	6.56E-02
chr19	11586604	11604849	-	j	*Ms4a6d*	protein_coding	1283	4.78	3.43E-03	8.74E-02
chr19	18671459	18704788	+	j	*2410127L17Rik*	protein_coding	1711	2.63	2.05E-03	7.43E-02
chr19	53012211	53038560	-	=	*Xpnpep1*	protein_coding	698	5.94	3.02E-03	8.67E-02
chr2	25498656	25500255	-	=	*C8g*	processed_transcript	868	2.89	3.92E-03	9.00E-02
chr2	26914858	26916332	-	=	*Surf1*	processed_transcript	389	5.35	4.28E-03	9.11E-02
chr2	29955507	29958217	+	=	*Gle1*	retained_intron	650	1.21	2.09E-04	3.27E-02
chr2	37066202	37201384	-	j	*Gm13434*	lincRNA	1207	1.04	3.28E-03	8.74E-02
chr2	68582413	68616387	+	=	*4933409G03Rik*	protein_coding	1263	3.13	6.23E-03	9.93E-02
chr2	75894109	75926148	+	=	*Agps*	nonsense_mediated_decay	586	3.92	2.14E-04	3.30E-02
chr2	95394161	95508038	+	j	*Gm13794*	lincRNA	768	7.00	2.66E-04	3.54E-02
chr2	120456019	120504913	+	=	*Capn3*	processed_transcript	2681	5.57	1.18E-03	6.26E-02
chr2	120718621	120720553	-	=	*Cdan1*	processed_transcript	458	7.07	3.05E-03	8.70E-02
chr2	137663390	137688239	+	j	*Gm14064*	lincRNA	788	2.42	4.11E-03	9.11E-02
chr2	145766378	145887616	+	j	*Rin2*	nonsense_mediated_decay	4853	1.68	2.64E-03	8.38E-02
chr2	147013297	147078000	+	j	*Xrn2*	protein_coding	3277	1.24	1.57E-03	6.76E-02
chr2	155382202	155389850	+	j	*Trp53inp2*	protein_coding	4383	3.38	7.14E-04	5.02E-02
chr2	166934782	166941454	+	=	*Cse1l*	nonsense_mediated_decay	718	2.90	4.91E-03	9.44E-02
chr2	167932372	167979385	+	=	*Ptpn1*	processed_transcript	4292	1.07	4.51E-03	9.21E-02
chr2	181696793	181698442	-	j	*4930526D03Rik*	protein_coding	706	2.46	2.88E-03	8.54E-02
chr3	88329046	88334268	-	=	*Tmem79*	protein_coding	2053	1.20	5.83E-03	9.81E-02
chr3	88368622	88372743	-	=	*Bglap3*	protein_coding	2051	1.95	1.72E-03	6.90E-02
chr3	90269016	90271697	+	=	*Dennd4b*	nonsense_mediated_decay	1073	6.86	1.45E-03	6.56E-02
chr3	123507937	123508066	-	=	*Snora24*	snoRNA	130	8.90	7.36E-04	5.09E-02
chr3	137864487	137866922	+	=	*H2afz*	processed_transcript	1304	1.90	5.62E-04	4.53E-02
chr3	153907289	153912301	-	j	*Rabggtb*	protein_coding	1310	6.36	1.70E-04	2.87E-02
chr4	11191354	11204675	+	=	*Ccne2*	protein_coding	3006	1.97	4.30E-03	9.11E-02
chr4	41767799	41769474	+	=	*Il11ra1*	retained_intron	848	2.35	6.23E-03	9.93E-02
chr4	41769467	41774142	-	=	*Ccl27a*	protein_coding	590	1.46	1.00E-03	5.85E-02

Supplementary Table 1. Up-regulated transcripts in F3 (RNA-seq) - *(Continued)*

chr	start	end	strand	class	gene	gene biotype	length	log2(FC)	p-value	adj p-value
chr4	41773188	41774169	-	=	*Ccl27a*	processed_transcript	657	2.71	2.13E-05	1.46E-02
chr4	42735545	42767287	+	=	*4930578G10Rik*	processed_transcript	426	5.48	2.57E-03	8.32E-02
chr4	42969946	42983639	+	=	*1700022I11Rik*	nonsense_mediated_decay	1017	1.87	1.62E-07	4.42E-04
chr4	43002343	43010506	-	j	*Fancg*	protein_coding	2917	5.93	2.12E-03	7.54E-02
chr4	43663096	43669145	-	=	*Fam221b*	retained_intron	3913	1.26	5.54E-03	9.74E-02
chr4	62634378	62703018	+	=	*Rgs3*	retained_intron	2911	1.28	1.11E-03	6.09E-02
chr4	62965573	63115748	+	=	*Zfp618*	protein_coding	1582	6.76	1.28E-04	2.58E-02
chr4	62979695	63139810	+	j	*Zfp618*	protein_coding	9044	2.63	8.62E-04	5.56E-02
chr4	63895545	63896099	-	=	*Rpl17-ps4*	processed_pseudogene	555	1.91	3.30E-03	8.74E-02
chr4	96723647	96786845	-	j	*Gm12695*	protein_coding	1816	5.10	1.22E-04	2.58E-02
chr4	109087410	109108031	-	=	*Osbpl9*	processed_transcript	491	2.55	4.06E-03	9.09E-02
chr4	120854819	120896579	+	=	*Rims3*	protein_coding	6505	4.33	4.39E-03	9.11E-02
chr4	129626078	129640960	-	j	*Txlna*	protein_coding	4881	2.23	3.40E-03	8.74E-02
chr4	143107379	143212995	-	j	*Prdm2*	protein_coding	7336	1.90	2.58E-03	8.32E-02
chr4	144526411	144527042	-	=	*Gm13121*	processed_pseudogene	632	1.65	5.51E-05	2.03E-02
chr4	152274232	152285337	+	j	*Gpr153*	protein_coding	3658	1.89	6.21E-03	9.93E-02
chr5	24319589	24351604	-	=	*Kcnh2*	protein_coding	4221	1.19	6.15E-03	9.92E-02
chr5	24996019	24998168	+	j	*1500035N22Rik*	protein_coding	1002	8.76	4.38E-05	2.03E-02
chr5	30588170	30625270	+	=	*Kcnk3*	protein_coding	3810	1.52	3.77E-03	8.95E-02
chr5	37242080	37292131	+	=	*Crmp1*	protein_coding	3049	2.05	5.90E-03	9.81E-02
chr5	37246691	37292132	+	j	*Crmp1*	protein_coding	2627	1.69	5.20E-04	4.41E-02
chr5	37446828	37664356	-	j	*Stk32b*	protein_coding	4955	1.56	2.12E-05	1.46E-02
chr5	66968566	67030853	+	=	*Limch1*	retained_intron	2457	2.04	5.96E-03	9.81E-02
chr5	89834848	89870586	-	j	*Adamts3*	protein_coding	1044	3.03	3.06E-04	3.68E-02
chr5	102912354	103100081	-	=	*Mapk10*	protein_coding	2357	1.45	1.38E-03	6.53E-02
chr5	108518554	108521383	-	j	*Cplx1*	protein_coding	2050	7.12	1.19E-03	6.27E-02
chr5	111743487	111761725	-	j	*E130006D01Rik*	lincRNA	652	1.63	6.35E-03	9.96E-02
chr5	131437333	132449360	-	j	*Auts2*	processed_transcript	5845	5.41	3.68E-03	8.87E-02
chr5	135106913	135137922	+	=	*Mlxipl*	nonsense_mediated_decay	3195	3.29	4.76E-03	9.30E-02
chr5	138363719	138388547	+	j	*Gm10874*	protein_coding	1206	1.06	1.18E-03	6.26E-02
chr5	140332395	140337808	+	=	*Nudt1*	protein_coding	606	6.07	3.72E-04	3.73E-02
chr5	151063546	151233774	-	=	*Stard13*	protein_coding	449	6.37	8.43E-04	5.52E-02
chr6	8630527	8778439	-	=	*Ica1*	protein_coding	1876	1.19	5.57E-03	9.76E-02
chr6	30546642	30564476	+	j	*Cpa2*	protein_coding	1114	4.45	7.19E-04	5.02E-02
chr6	47943208	47953153	+	=	*Zfp783*	processed_transcript	2586	4.68	5.40E-03	9.58E-02
chr6	56714713	56761911	+	j	*Avl9*	protein_coding	6977	1.04	4.55E-03	9.22E-02
chr6	71128398	71144338	-	=	*Thnsl2*	protein_coding	1812	2.43	5.95E-03	9.81E-02
chr6	83702491	83702677	+	=	*Vax2os1_1*	misc_RNA	187	1.57	6.18E-03	9.92E-02
chr6	90369492	90385084	+	=	*Zxdc*	protein_coding	4864	4.81	5.86E-03	9.81E-02
chr6	124815019	124829484	-	j	*Usp5*	protein_coding	3639	2.61	5.84E-03	9.81E-02
chr6	124858444	124863597	-	j	*Gpr162*	protein_coding	2608	1.07	4.30E-03	9.11E-02
chr6	142149026	142157298	-	=	*Slco1a6*	processed_transcript	458	1.15	1.46E-04	2.86E-02
chr7	6286979	6307883	+	j	*Zfp667*	protein_coding	3646	1.64	5.09E-05	2.03E-02
chr7	25284670	25285684	+	=	*Cic*	nonsense_mediated_decay	745	1.07	4.55E-03	9.22E-02
chr7	25766518	25774678	-	=	*Axl*	protein_coding	563	6.16	2.66E-04	3.54E-02
chr7	29843936	29853648	-	=	*Zfp940*	protein_coding	2764	1.89	5.67E-03	9.76E-02
chr7	38183898	38194972	+	=	*1600014C10Rik*	processed_transcript	568	8.72	3.37E-03	8.74E-02
chr7	41284327	41290183	+	=	*Gm5592*	protein_coding	3033	1.16	2.72E-03	8.41E-02
chr7	44900373	44928643	-	j	*Ap2a1*	protein_coding	3399	2.22	6.39E-03	1.00E-01
chr7	45303155	45334554	-	j	*Trpm4*	protein_coding	5355	1.43	2.29E-03	7.89E-02
chr7	68275972	68363089	+	j	*Fam169b*	lincRNA	2835	6.57	5.23E-03	9.58E-02
chr7	105690259	105690567	-	=	*Mrps36-ps2*	processed_pseudogene	309	1.54	3.09E-03	8.70E-02
chr7	109523911	109617147	-	=	*St5*	protein_coding	3004	2.01	3.28E-03	8.74E-02
chr7	114354640	114415021	-	=	*4933406I18Rik*	lincRNA	905	1.87	2.87E-03	8.54E-02
chr7	127865717	127876780	-	=	*Zfp668*	protein_coding	3132	1.93	4.82E-03	9.35E-02
chr7	139086027	139101795	+	=	*Dpysl4*	retained_intron	4196	1.01	2.45E-03	8.21E-02
chr7	139923778	139941537	+	=	*Kndc1*	protein_coding	3813	1.24	3.26E-03	8.74E-02
chr8	8617434	8660773	-	j	*Efnb2*	protein_coding	4231	5.78	3.46E-03	8.74E-02
chr8	33536597	33585616	+	j	*Tex15*	nonsense_mediated_decay	7957	2.47	3.96E-03	9.01E-02

Supplementary Table 1. Up-regulated transcripts in F3 (RNA-seq) - *(Continued)*

chr	start	end	strand	class	gene	gene biotype	length	log2(FC)	p-value	adj p-value
chr8	33732339	33777173	+	=	*Gtf2e2*	protein_coding	1664	1.46	3.27E-03	8.74E-02
chr8	83332604	83386874	+	j	*4933434I20Rik*	protein_coding	4195	3.28	1.37E-05	1.30E-02
chr8	83934577	83937496	+	=	*Lphn1*	retained_intron	909	1.09	3.94E-03	9.01E-02
chr8	94600142	94660275	+	j	*Rspry1*	protein_coding	3382	6.50	1.67E-03	6.78E-02
chr8	104511028	104534636	-	=	*Nae1*	protein_coding	1735	2.42	1.34E-03	6.47E-02
chr8	105654739	105682922	+	j	*Ctcf*	protein_coding	3607	1.59	4.79E-03	9.32E-02
chr8	110847024	110882227	+	j	*Cog4*	protein_coding	2453	2.98	2.63E-03	8.38E-02
chr8	117349401	117461503	+	j	*Cmip*	protein_coding	4257	2.65	5.77E-03	9.81E-02
chr9	20888250	20890341	+	=	*Ppan*	retained_intron	1157	2.48	3.88E-03	9.00E-02
chr9	21723576	21749916	+	=	*Ldlr*	protein_coding	4531	1.34	7.83E-04	5.25E-02
chr9	31387007	31421333	+	j	*Nfrkb*	nonsense_mediated_decay	5208	1.43	1.28E-03	6.42E-02
chr9	42469518	42505776	+	j	*Gm16322*	antisense	1001	1.48	2.70E-04	3.54E-02
chr9	42469532	42505776	+	j	*Gm16322*	antisense	942	1.15	8.65E-04	5.56E-02
chr9	45945669	45951772	-	=	*Sidt2*	protein_coding	661	5.48	4.81E-03	9.35E-02
chr9	57412668	57440114	-	=	*Ppcdc*	protein_coding	2747	1.16	2.33E-03	7.97E-02
chr9	75828022	75843600	+	=	*Bmp5*	processed_transcript	541	2.63	2.22E-03	7.76E-02
chr9	78235003	78242595	+	=	*Gsta1*	retained_intron	683	3.06	5.06E-04	4.40E-02
chr9	82866159	82975503	-	j	*Phip*	protein_coding	10806	2.39	3.10E-03	8.71E-02
chr9	98296662	98411391	+	=	*Nmnat3*	protein_coding	1898	2.15	1.57E-03	6.76E-02
chr9	101922128	101940997	-	j	*Ephb1*	protein_coding	2069	1.77	6.60E-05	2.06E-02
chr9	102618817	102626509	-	=	*Cep63*	protein_coding	536	2.15	3.50E-03	8.74E-02
chr9	102999010	103088315	+	j	*Slco2a1*	protein_coding	8757	4.31	3.88E-04	3.76E-02
chr9	105643397	105687655	+	j	*Pik3r4*	protein_coding	4327	2.31	1.20E-03	6.27E-02
chr9	111182293	111225668	+	j	*Lrrfip2*	protein_coding	2852	1.99	4.16E-03	9.11E-02
chr9	120128780	120128935	+	=	*Gm24044*	snoRNA	156	3.08	5.18E-03	9.58E-02
chrX	8185329	8187174	-	j	*Ebp*	protein_coding	928	3.65	5.04E-03	9.52E-02
chrX	12587787	12617049	+	j	*Atp6ap2*	protein_coding	2334	6.41	3.62E-03	8.86E-02
chrX	42071325	42109656	+	=	*Xiap*	protein_coding	6374	2.67	5.51E-03	9.70E-02
chrX	57920038	58041736	+	j	*Zic3*	protein_coding	2152	3.09	1.58E-03	6.76E-02
chrX	74297097	74304721	+	j	*Atp6ap1*	protein_coding	1929	1.30	4.85E-03	9.36E-02
chrX	78517305	78583891	-	=	*Prrg1*	retained_intron	3725	4.23	4.38E-03	9.11E-02
chrX	103493558	103525214	+	j	*Jpx*	lincRNA	584	1.71	3.50E-04	3.68E-02
chrX	136765607	136803364	+	=	*BC065397*	processed_transcript	768	2.14	4.68E-03	9.22E-02
chrX	139210042	139238333	+	=	*Mum1l1*	protein_coding	4600	1.35	2.00E-03	7.35E-02
chrX	154109633	154120685	-	=	*Gm15140*	protein_coding	984	1.84	9.90E-04	5.85E-02
chrX	161124268	161258213	+	j	*Scml2*	protein_coding	4517	1.47	6.07E-03	9.84E-02
chrX	169320622	169370003	+	=	*Gm15246*	processed_transcript	2353	1.72	1.64E-03	6.78E-02

Supplementary Table 2. Down-regulated transcripts in F3 (RNA-seq)

chr	start	end	strand	class	gene	gene biotype	length	log2(FC)	p-value	adj p-value
chr1	24219205	24252684	+	j	*Col9a1*	protein_coding	2162	-1.63	4.48E-03	9.19E-02
chr1	33453811	33638139	-	j	*Prim2*	protein_coding	1507	-10.15	5.69E-03	9.76E-02
chr1	34439905	34443948	+	=	*Imp4*	nonsense_mediated_decay	750	-2.70	9.31E-04	5.72E-02
chr1	60180507	60338328	+	=	*Nbeal1*	protein_coding	15823	-2.42	1.85E-03	7.06E-02
chr1	74541437	74567794	+	j	*Plcd4*	nonsense_mediated_decay	5185	-1.12	1.28E-03	6.42E-02
chr1	92916805	92919216	+	=	*Rnpepl1*	retained_intron	822	-7.00	1.58E-03	6.76E-02
chr1	134293306	134329429	-	j	*Ppfia4*	protein_coding	3480	-1.27	4.16E-03	9.11E-02
chr1	143739615	143746776	+	=	*Glrx2*	protein_coding	580	-1.66	5.95E-03	9.81E-02
chr1	155794343	155795417	-	=	*Qsox1*	retained_intron	373	-2.94	3.28E-04	3.68E-02
chr1	179668210	179687189	+	j	*Sccpdh*	protein_coding	1891	-2.53	5.31E-03	9.58E-02
chr1	191894074	191899256	-	j	*1700034H15Rik*	nonsense_mediated_decay	3716	-1.82	4.95E-03	9.46E-02
chr10	7119062	7169961	-	j	*Cnksr3*	protein_coding	2985	-3.26	9.06E-05	2.36E-02
chr10	39118810	39133848	-	=	*Fam229b*	protein_coding	700	-4.02	7.58E-05	2.13E-02
chr10	97482337	97518162	+	j	*Dcn*	protein_coding	1638	-7.43	1.58E-04	2.87E-02
chr11	6597892	6599392	-	=	*Nacad*	nonsense_mediated_decay	521	-2.97	1.77E-03	6.97E-02
chr11	43420269	43426202	-	=	*Pttg1*	protein_coding	700	-2.03	2.88E-03	8.54E-02
chr11	43474306	43491525	+	=	*C1qtnf2*	protein_coding	1211	-2.97	2.98E-05	1.83E-02
chr11	54304022	54325660	+	=	*Acsl6*	protein_coding	691	-3.83	2.59E-04	3.54E-02
chr11	61208621	61218421	+	=	*Aldh3a1*	protein_coding	1721	-2.84	4.10E-03	9.11E-02
chr11	61951739	62223040	+	j	*Specc1*	protein_coding	7274	-6.30	4.46E-04	4.13E-02
chr11	69670469	69671450	-	=	*Eif4a1*	retained_intron	802	-1.29	3.87E-03	9.00E-02
chr11	72155393	72175238	-	=	*4933427D14Rik*	processed_transcript	780	-1.62	6.09E-04	4.63E-02
chr11	73693923	73710763	+	=	*Zfp735*	processed_transcript	454	-1.01	2.50E-04	3.51E-02
chr11	82952102	82962941	+	=	*Slfn5*	protein_coding	4080	-2.53	5.90E-03	9.81E-02
chr11	89390223	89696887	-	j	*Ankfn1*	protein_coding	4788	-4.97	1.42E-03	6.56E-02
chr11	97162510	97164222	-	=	*Kpnb1*	retained_intron	643	-5.40	5.70E-03	9.76E-02
chr11	97452130	97502402	+	=	*Arhgap23*	nonsense_mediated_decay	4531	-2.97	5.29E-03	9.58E-02
chr11	101627195	101630900	+	=	*Rdm1*	processed_transcript	434	-2.09	2.76E-03	8.45E-02
chr11	103026988	103028263	-	=	*Dcakd*	retained_intron	549	-1.99	4.59E-03	9.22E-02
chr11	103821782	103954056	-	j	*Nsf*	protein_coding	3814	-7.06	1.97E-03	7.30E-02
chr11	106814223	106819930	+	j	*Cep95*	protein_coding	1935	-5.45	3.89E-03	9.00E-02
chr11	115607918	115612927	-	=	*Mif4gd*	processed_transcript	1271	-1.44	6.97E-04	5.01E-02
chr11	115644045	115708597	-	=	*Grb2*	protein_coding	2709	-1.75	2.87E-03	8.54E-02
chr11	116537672	116562726	-	j	*Ube2o*	protein_coding	4986	-5.66	5.10E-03	9.57E-02
chr12	106695716	106716362	-	j	*4933406K04Rik*	lincRNA	1208	-2.11	1.37E-03	6.53E-02
chr12	106706049	106716362	-	j	*4933406K04Rik*	lincRNA	1495	-2.14	5.13E-03	9.58E-02
chr13	3478243	3488058	+	j	*2810429I04Rik*	lincRNA	3251	-6.10	3.47E-03	8.74E-02
chr13	54789645	54795148	+	=	*Tspan17*	protein_coding	433	-6.39	2.53E-03	8.32E-02
chr14	6301794	6304780	-	=	*Gm21103*	protein_coding	618	-2.23	1.13E-03	6.09E-02
chr14	7084348	7090812	-	=	*Gm3696*	protein_coding	612	-5.65	1.36E-03	6.53E-02
chr14	11553527	12242041	+	j	*Ptprg*	protein_coding	9007	-1.24	4.57E-03	9.22E-02
chr14	33377898	33447158	-	=	*Mapk8*	protein_coding	5756	-1.73	1.87E-04	3.01E-02
chr14	46761482	46771525	+	j	*Cdkn3*	protein_coding	690	-8.70	3.30E-04	3.68E-02
chr14	55561067	55569931	+	=	*Dcaf11*	protein_coding	2311	-6.25	5.66E-03	9.76E-02
chr14	73143110	73166840	+	=	*Rcbtb2*	protein_coding	465	-1.93	2.02E-03	7.35E-02
chr14	73195502	73330575	-	j	*Rb1*	protein_coding	4371	-6.49	7.20E-04	5.02E-02
chr14	103081684	103098975	-	=	*Fbxl3*	protein_coding	2534	-1.59	5.28E-04	4.43E-02
chr15	7129559	7197489	+	=	*Lifr*	protein_coding	9889	-2.66	5.91E-03	9.81E-02
chr15	12215203	12272240	+	j	*Mtmr12*	protein_coding	4335	-2.64	4.41E-03	9.12E-02
chr15	37923952	37939616	-	j	*Rrm2b*	protein_coding	3821	-5.57	2.28E-03	7.89E-02
chr15	39091229	39107860	-	j	*Slc25a32*	protein_coding	5559	-2.05	1.98E-03	7.32E-02
chr15	57243767	57477625	-	j	*Slc22a22*	protein_coding	2434	-3.64	7.72E-04	5.24E-02
chr15	73518614	73572242	+	j	*Dennd3*	nonsense_mediated_decay	4895	-5.55	1.55E-03	6.76E-02
chr15	102326116	102338139	+	j	*Myg1*	protein_coding	2893	-2.22	5.69E-03	9.76E-02
chr15	103153297	103163368	-	j	*Smug1*	protein_coding	3375	-1.42	4.22E-03	9.11E-02
chr16	4886100	4938296	+	=	*Mgrn1*	protein_coding	3293	-2.50	1.03E-03	5.87E-02
chr16	18498897	18544225	+	=	*Gnb1l*	protein_coding	637	-3.14	2.05E-03	7.43E-02
chr16	36359382	36371251	-	j	*BC100530*	protein_coding	580	-5.61	5.11E-03	9.58E-02
chr16	66663093	66953334	-	=	*Cadm2*	protein_coding	1308	-1.08	5.06E-03	9.55E-02

Supplementary Table 2. Down-regulated transcripts in F3 (RNA-seq) - *(Continued)*

chr	start	end	strand	class	gene	gene biotype	length	log2(FC)	p-value	adj p-value
chr16	90826719	90904695	+	=	*Eva1c*	protein_coding	1948	-1.09	5.08E-03	9.55E-02
chr16	97564684	97578438	-	j	*Tmprss2*	protein_coding	2776	-1.63	4.64E-03	9.22E-02
chr17	24900544	24901718	-	=	*Mapk8ip3*	retained_intron	751	-5.62	5.40E-03	9.58E-02
chr17	25828867	25831840	+	=	*Jmjd8*	protein_coding	2265	-1.19	1.30E-03	6.42E-02
chr17	31944769	32036193	-	j	*Hsf2bp*	protein_coding	3086	-7.20	5.74E-04	4.56E-02
chr17	34950238	34952471	-	j	*1110038B12Rik*	lincRNA	797	-2.28	6.00E-03	9.81E-02
chr17	35235980	35238767	+	=	*Atp6v1g2*	retained_intron	2517	-1.20	2.18E-03	7.72E-02
chr17	47371712	47377819	+	=	*Mrps10*	processed_transcript	405	-1.31	1.01E-03	5.87E-02
chr17	66492241	66519377	-	j	*Rab12*	protein_coding	1918	-1.28	1.61E-03	6.76E-02
chr17	78377885	78405458	-	j	*Fez2*	protein_coding	1644	-1.63	4.83E-03	9.36E-02
chr18	67616396	67641436	-	j	*Cep76*	protein_coding	2659	-1.97	1.63E-03	6.76E-02
chr18	75049714	75286966	+	j	*Dym*	protein_coding	2091	-1.32	3.50E-03	8.74E-02
chr18	80229758	80247102	-	=	*Hsbp1l1*	protein_coding	1251	-1.67	1.00E-03	5.85E-02
chr18	80606205	80682913	-	j	*Nfatc1*	protein_coding	3171	-1.96	1.63E-03	6.76E-02
chr19	7275396	7341819	-	j	*Mark2*	protein_coding	4406	-1.58	1.84E-03	7.06E-02
chr19	18631950	18651927	+	=	*Nmrk1*	protein_coding	1357	-3.67	3.14E-03	8.71E-02
chr19	21551957	21557863	+	j	*Gm3443*	protein_coding	903	-2.60	4.64E-03	9.22E-02
chr19	21551957	21557863	+	j	*Gm3443*	protein_coding	931	-2.52	5.55E-05	2.03E-02
chr19	21552295	21557863	+	j	*Gm3443*	protein_coding	565	-2.53	6.26E-03	9.95E-02
chr19	21552295	21557863	+	j	*Gm3443*	protein_coding	593	-2.19	8.21E-04	5.41E-02
chr2	5809555	5844972	-	j	*Cdc123*	protein_coding	1017	-1.87	5.19E-05	2.03E-02
chr2	26888628	26902879	-	j	*Surf6*	protein_coding	1431	-1.30	1.65E-03	6.78E-02
chr2	32778215	32779146	-	=	*1700019L03Rik*	processed_transcript	269	-2.01	5.91E-05	2.06E-02
chr2	39132979	39190728	-	=	*Scai*	protein_coding	368	-6.35	5.38E-03	9.58E-02
chr2	60842297	60888055	-	=	*Rbms1*	processed_transcript	224	-6.42	2.95E-03	8.62E-02
chr2	90847231	90866640	+	=	*Mtch2*	protein_coding	2377	-1.13	2.19E-03	7.72E-02
chr2	93311263	93334505	-	=	*Tspan18*	processed_transcript	674	-2.87	1.70E-03	6.87E-02
chr2	93955810	94010807	-	j	*Alkbh3*	protein_coding	2158	-3.72	3.55E-03	8.80E-02
chr2	104704950	104712166	-	=	*Tcp11l1*	processed_transcript	630	-1.19	1.83E-03	7.06E-02
chr2	119288787	119298326	+	=	*Vps18*	processed_transcript	3914	-3.15	2.99E-04	3.68E-02
chr2	121140428	121156680	+	=	*Adal*	protein_coding	2393	-2.87	6.24E-03	9.93E-02
chr2	127040561	127040685	+	=	*Gm24081*	miRNA	125	-8.89	3.65E-03	8.87E-02
chr2	130424404	130440296	+	=	*Vps16*	processed_transcript	907	-3.83	5.76E-04	4.56E-02
chr2	145903297	145911998	+	=	*Naa20*	retained_intron	417	-3.20	4.16E-04	3.96E-02
chr2	158698523	158766334	+	j	*Ppp1r16b*	protein_coding	6007	-1.88	6.27E-03	9.95E-02
chr2	167503446	167516164	+	=	*Rnf114*	nonsense_mediated_decay	1985	-1.01	2.95E-03	8.62E-02
chr3	17782070	17786898	-	=	*Gm10742*	protein_coding	4829	-1.86	3.73E-04	3.73E-02
chr3	40502708	40523038	-	j	*1700017G19Rik*	lincRNA	3104	-3.71	4.48E-03	9.19E-02
chr3	51661179	51682675	+	=	*Mgst2*	protein_coding	603	-1.88	4.96E-03	9.46E-02
chr3	79598709	79602328	+	=	*Ppid*	retained_intron	512	-3.69	3.78E-03	8.96E-02
chr3	87290284	87371074	+	j	*Cd5l*	protein_coding	2747	-3.99	1.23E-04	2.58E-02
chr3	87745252	87748594	-	=	*Lrrc71*	retained_intron	842	-1.24	1.96E-03	7.30E-02
chr3	92620081	92621660	-	=	*Lce6a*	protein_coding	795	-4.18	5.85E-03	9.81E-02
chr3	95070826	95078516	-	=	*Pip5k1a*	processed_transcript	632	-3.92	7.21E-04	5.02E-02
chr3	96637053	96643800	+	=	*Pex11b*	retained_intron	506	-2.27	3.36E-03	8.74E-02
chr3	130041256	130061553	-	=	*Sec24b*	protein_coding	306	-8.09	5.46E-03	9.64E-02
chr3	138277651	138290698	+	=	*Adh1*	nonsense_mediated_decay	1115	-3.03	1.05E-04	2.55E-02
chr4	9449079	9669162	-	=	*Asph*	protein_coding	6694	-5.85	4.02E-04	3.86E-02
chr4	10479962	10797848	-	j	*1700123O12Rik*	lincRNA	1299	-4.69	1.55E-03	6.76E-02
chr4	41769467	41774097	-	=	*Ccl27a*	protein_coding	841	-2.50	6.60E-04	4.85E-02
chr4	42158843	42159876	-	=	*Gm13305*	retained_intron	522	-1.37	2.77E-03	8.45E-02
chr4	43578958	43583130	+	=	*Rgp1*	protein_coding	1292	-2.43	4.36E-03	9.11E-02
chr4	45300434	45315142	+	=	*Trmt10b*	protein_coding	983	-1.01	2.85E-03	8.54E-02
chr4	62619520	62703018	+	=	*Rgs3*	protein_coding	3888	-6.36	1.88E-03	7.10E-02
chr4	63086985	63139810	+	j	*Zfp618*	protein_coding	8778	-5.51	2.88E-03	8.54E-02
chr4	111875429	111884254	-	=	*Slc5a9*	processed_transcript	2814	-3.23	4.61E-03	9.22E-02
chr4	120930353	120949434	-	=	*Zfp69*	protein_coding	1764	-3.35	5.30E-04	4.43E-02
chr4	126609818	126614371	+	=	*5730409E04Rik*	protein_coding	2886	-5.95	9.20E-04	5.72E-02
chr4	129600668	129608528	+	j	*Tmem234*	nonsense_mediated_decay	1777	-1.86	2.06E-03	7.43E-02

Supplementary Table 2. Down-regulated transcripts in F3 (RNA-seq) - *(Continued)*

chr	start	end	strand	class	gene	gene biotype	length	log2(FC)	p-value	adj p-value
chr4	131861710	131867601	+	=	*Mecr*	processed_transcript	532	-2.56	2.93E-03	8.60E-02
chr4	133266830	133277662	-	=	*Tmem222*	nonsense_mediated_decay	537	-4.48	2.27E-04	3.31E-02
chr4	151989624	151994203	-	j	*Phf13*	protein_coding	2806	-1.81	1.39E-03	6.53E-02
chr5	23852210	23852280	+	=	*Snord93*	snoRNA	71	-13.68	5.23E-03	9.58E-02
chr5	24996019	24998168	+	j	*1500035N22Rik*	protein_coding	911	-1.78	4.23E-03	9.11E-02
chr5	65761445	65763357	-	=	*1700022K14Rik*	antisense	235	-3.20	2.37E-06	3.24E-03
chr5	86014398	86065285	-	j	*Cenpc1*	protein_coding	2670	-3.54	9.22E-04	5.72E-02
chr5	92736205	92737442	+	=	*Gm20500*	processed_transcript	321	-1.33	1.62E-03	6.76E-02
chr5	101658139	101665226	-	=	*Nkx6-1*	protein_coding	3368	-3.60	1.81E-03	7.05E-02
chr5	104070064	104072957	-	j	*Gm17660*	protein_coding	720	-2.13	1.39E-03	6.53E-02
chr5	115466266	115474722	+	=	*Pla2g1b*	protein_coding	557	-5.73	3.72E-03	8.89E-02
chr5	121378631	121385409	-	=	*Trafd1*	protein_coding	627	-2.07	3.03E-03	8.67E-02
chr5	125515245	125518591	+	j	*Aacs*	protein_coding	2479	-1.26	4.35E-03	9.11E-02
chr5	137629121	137642902	+	j	*Gm20605*	nonsense_mediated_decay	4476	-1.65	3.69E-03	8.87E-02
chr6	8208502	8236274	+	j	*Mios*	protein_coding	3551	-1.09	6.45E-04	4.80E-02
chr6	9157231	9253892	+	j	*Nxph1*	protein_coding	930	-1.78	4.33E-04	4.05E-02
chr6	14712531	14781050	-	j	*Ppp1r3a*	protein_coding	676	-1.54	4.75E-04	4.29E-02
chr6	38551334	38606367	+	j	*Luc7l2*	nonsense_mediated_decay	7720	-3.30	2.72E-03	8.41E-02
chr6	48739054	48743790	+	=	*Gimap1*	protein_coding	1487	-5.82	3.46E-03	8.74E-02
chr6	121183667	121196185	+	=	*Pex26*	protein_coding	3778	-1.23	1.61E-03	6.76E-02
chr6	131284523	131293131	-	=	*Magohb*	nonsense_mediated_decay	460	-3.52	9.12E-04	5.72E-02
chr6	136923523	136941756	-	=	*Arhgdib*	protein_coding	1325	-1.02	3.87E-03	9.00E-02
chr6	145211175	145215771	+	=	*Lyrm5*	protein_coding	842	-3.91	3.99E-03	9.04E-02
chr6	148877915	148895459	-	=	*Caprin2*	protein_coding	739	-3.67	4.68E-03	9.22E-02
chr6	148921035	148944881	-	=	*Fam60a*	protein_coding	2653	-6.95	2.07E-03	7.43E-02
chr7	19671584	19673920	-	=	*Apoc2*	protein_coding	569	-2.87	5.64E-03	9.76E-02
chr7	24301796	24316666	-	=	*Zfp94*	protein_coding	2529	-1.05	1.73E-03	6.90E-02
chr7	27653915	27656208	+	=	*Ttc9b*	protein_coding	914	-1.83	1.73E-03	6.90E-02
chr7	29893311	29906121	-	j	*Zfp27*	protein_coding	3567	-2.56	4.59E-03	9.22E-02
chr7	29893716	29906104	-	=	*Zfp27*	protein_coding	3150	-3.49	1.13E-04	2.58E-02
chr7	31116525	31126945	-	=	*Scn1b*	protein_coding	1514	-1.03	1.44E-03	6.56E-02
chr7	38262820	38271348	-	=	*Pop4*	protein_coding	1214	-1.73	6.18E-03	9.92E-02
chr7	41667979	41694902	+	j	*Vmn2r-ps54*	processed_transcript	1511	-4.31	2.94E-04	3.68E-02
chr7	41667979	41727193	+	j	*Vmn2r-ps54*	processed_transcript	2905	-1.46	5.67E-03	9.76E-02
chr7	45056805	45062403	-	=	*Prrg2*	protein_coding	362	-7.72	9.69E-04	5.79E-02
chr7	68300113	68363087	+	=	*Fam169b*	lincRNA	2776	-4.01	4.27E-03	9.11E-02
chr7	75863017	75874137	+	j	*Klhl25*	protein_coding	3258	-2.08	3.86E-03	9.00E-02
chr7	80026228	80092751	+	=	*Zfp710*	protein_coding	4532	-3.43	6.79E-04	4.95E-02
chr7	80311105	80315517	+	=	*Prc1*	protein_coding	733	-4.17	5.62E-03	9.76E-02
chr7	85221371	85235710	-	j	*Vmn2r68*	protein_coding	3631	-1.15	1.19E-03	6.27E-02
chr7	109523920	109616568	-	=	*St5*	protein_coding	3176	-5.92	7.98E-05	2.13E-02
chr7	113513811	113571511	+	j	*Far1*	protein_coding	4981	-6.09	3.50E-04	3.68E-02
chr7	124355017	124398989	+	j	*Hs3st4*	protein_coding	3221	-1.47	1.07E-04	2.55E-02
chr7	127843598	127848677	+	=	*Stx4a*	retained_intron	659	-4.18	3.46E-04	3.68E-02
chr8	22128283	22166435	-	j	*Nek3*	protein_coding	1990	-3.27	4.25E-03	9.11E-02
chr8	23226610	23237714	-	j	*Gins4*	protein_coding	1335	-2.20	3.42E-03	8.74E-02
chr8	27123388	27128191	-	j	*Brf2*	protein_coding	1644	-1.67	4.26E-03	9.11E-02
chr8	33516704	33585616	+	j	*Tex15*	nonsense_mediated_decay	9549	-1.95	4.51E-03	9.21E-02
chr8	33536617	33585616	+	j	*Tex15*	nonsense_mediated_decay	8014	-4.06	5.16E-03	9.58E-02
chr8	33615786	33642710	-	j	*Ubxn8*	protein_coding	1799	-2.50	3.87E-03	9.00E-02
chr8	33729712	33731842	-	j	*1700104B16Rik*	protein_coding	1567	-5.18	9.50E-06	1.04E-02
chr8	33730549	33731814	-	=	*1700104B16Rik*	protein_coding	555	-7.89	7.43E-08	3.60E-04
chr8	33784066	33853673	-	=	*Rbpms*	processed_transcript	643	-1.94	3.58E-03	8.80E-02
chr8	34090563	34108536	-	=	*Dctn6*	protein_coding	962	-2.58	1.68E-04	2.87E-02
chr8	34090578	34107814	-	=	*Dctn6*	processed_transcript	1050	-2.55	1.06E-03	5.96E-02
chr8	34826457	34964539	-	j	*Tnks*	protein_coding	8569	-8.05	2.70E-03	8.41E-02
chr8	54629055	54724504	-	j	*Wdr17*	protein_coding	4917	-1.18	2.18E-03	7.72E-02
chr8	102632995	102700399	-	j	*Cdh11*	protein_coding	4310	-1.42	1.61E-03	6.76E-02
chr9	8221781	8275071	+	j	*1700128F08Rik*	protein_coding	873	-2.40	1.58E-03	6.76E-02

Supplementary Table 2. Down-regulated transcripts in F3 (RNA-seq) - *(Continued)*

chr	start	end	strand	class	gene	gene biotype	length	log2(FC)	p-value	adj p-value
chr9	14784654	14837123	+	j	*Mre11a*	protein_coding	5813	-6.00	9.52E-04	5.79E-02
chr9	54165928	54183297	-	j	*Cyp19a1*	protein_coding	2267	-1.41	3.30E-05	1.90E-02
chr9	58196298	58204319	-	j	*Islr2*	protein_coding	4070	-6.32	2.55E-03	8.32E-02
chr9	72532735	72612486	+	=	*Rfx7*	protein_coding	1554	-4.35	1.74E-03	6.90E-02
chr9	78175914	78178879	+	j	*C920006O11Rik*	lincRNA	1441	-5.73	5.38E-03	9.58E-02
chr9	78230656	78242684	+	=	*Gsta1*	protein_coding	875	-3.00	2.78E-03	8.47E-02
chr9	88789285	88830908	+	j	*Gm10634*	protein_coding	934	-1.10	4.35E-03	9.11E-02
chr9	88843510	88858801	-	=	*9330159M07Rik*	lincRNA	1044	-1.48	1.33E-03	6.46E-02
chr9	89826944	89838978	+	=	*RP23-184F1.2*	lincRNA	2719	-1.09	1.84E-03	7.06E-02
chr9	98003420	98012175	+	=	*4921534H16Rik*	antisense	2838	-4.09	5.16E-03	9.58E-02
chr9	98287455	98368160	+	=	*Nmnat3*	processed_transcript	1059	-1.11	5.36E-03	9.58E-02
chr9	101941026	101943227	+	j	*9630041A04Rik*	protein_coding	1479	-1.25	5.21E-04	4.41E-02
chr9	103008489	103087849	+	=	*Slco2a1*	protein_coding	4033	-1.44	4.62E-03	9.22E-02
chr9	108513109	108515158	+	=	*Qars*	protein_coding	459	-8.51	3.31E-03	8.74E-02
chr9	110939610	110946158	-	=	*Tdgf1*	protein_coding	1925	-3.86	4.79E-03	9.32E-02
chr9	123022037	123023630	+	j	*Tmem42*	protein_coding	973	-1.95	3.55E-03	8.80E-02
chrX	42149412	42277175	+	=	*Stag2*	protein_coding	5955	-5.03	5.46E-03	9.64E-02
chrX	73494892	73495926	+	=	*Bgn*	processed_transcript	548	-1.29	3.42E-03	8.74E-02
chrX	96247203	96293438	-	=	*Vsig4*	protein_coding	1432	-1.77	1.32E-03	6.42E-02
chrX	150157415	150336343	-	j	*Gm15104*	lincRNA	1460	-4.97	1.67E-03	6.78E-02
chrX	169320403	169370003	+	j	*Gm15246*	processed_transcript	3204	-1.20	5.97E-03	9.81E-02

Supplementary Table 3. H3K4me3 differential peaks identified in F1

chr	start	stop	gene 1	gene 2
chr1	8047160	8047240	*Sntg1*	*Pcmtd1*
chr1	13012823	13013146	*Slco5a1*	*Prdm14*
chr1	16161821	16163027	*Rdh10*	*Stau2*
chr1	24594621	24594700	*Col19a1*	*Gm10222*
chr1	24595283	24595414	*Col19a1*	*Gm10222*
chr1	33680809	33681260	*Rab23*	*1700001G17Rik*
chr1	34510163	34511436	*Cfc1*	*Prss39*
chr1	36594435	36594717	*Sema4c*	*Fam178b*
chr1	36602907	36602995	*Sema4c*	*Fam178b*
chr1	42494538	42494663	*Pou3f3*	
chr1	52991209	52992140	*Mstn*	*1700019D03Rik*
chr1	53987709	53987783	*Stk17b*	*Hecw2*
chr1	57588464	57588548	*Spats2l*	*Tyw5*
chr1	58420043	58420575	*Clk1*	*Bzw1*
chr1	59249275	59250478	*Als2*	*Cdk15*
chr1	59448367	59449923	*Fzd7*	*Cdk15*
chr1	61796329	61797311	*Nrp2*	*Pard3b*
chr1	67055668	67056368	*Cps1*	*Lancl1*
chr1	67792405	67794349	*Cps1*	
chr1	82931171	82931257	*Agfg1*	*Slc19a3*
chr1	82943147	82943245	*Slc19a3*	*Agfg1*
chr1	83535245	83536357	*Sphkap*	*Pid1*
chr1	86087766	86088809	*Psmd1*	*Htr2b*
chr1	86983217	86984594	*Alppl2*	*Dis3l2*
chr1	87438202	87439176	*Kcnj13*	*3110079O15Rik*
chr1	88499299	88499864	*Glrp1*	*Spp2*
chr1	106168970	106169059	*Phlpp1*	
chr1	108977513	108978338		
chr1	113772971	113773106		
chr1	119896643	119897300	*Ptpn4*	*Tmem177*
chr1	130175036	130175573	*Daf2*	*Thsd7b*
chr1	130175715	130175977	*Daf2*	*Thsd7b*
chr1	130547656	130548581	*Cd55*	*Zp3r*
chr1	131678597	131680572	*5430435G22Rik*	*Ctse*
chr1	134670841	134672129	*Syt2*	*Kdm5b*
chr1	138525618	138525840	*Nek7*	*Atp6v1g3*
chr1	139650242	139650347	*Cfhr3*	*Gm4788*
chr1	141823268	141823407	*Gm4845*	
chr1	145164981	145165333	*Rgs18*	
chr1	151329180	151329260	*Hmcn1*	*Ivns1abp*
chr1	151329348	151329596	*Hmcn1*	*Ivns1abp*
chr1	153431546	153432066	*Shcbp1l*	*Dhx9*
chr1	155028130	155028247	*Cacna1e*	*Ier5*
chr1	155663186	155663480	*Lhx4*	*Acbd6*
chr1	155693506	155694522	*Lhx4*	*Acbd6*
chr1	158744720	158744896	*Pappa2*	*Astn1*
chr1	164443480	164444698	*Atp1b1*	*Nme7*
chr1	174212415	174212752	*Olfr419*	*Spta1*
chr1	175385704	175386525	*Grem2*	*Rgs7*
chr1	177110741	177111370	*Akt3*	*Sdccag8*
chr1	180403308	180403879	*Gm5069*	*6330403A02Rik*
chr1	182719530	182719664	*4922505E12Rik*	*Capn8*
chr1	182983087	182983856	*Tlr5*	*Disp1*
chr1	185255109	185256201	*Rab3gap2*	*Iars2*
chr1	185332844	185332951	*Iars2*	*Bpnt1*
chr1	190896550	190896835	*Prox1*	*Rps6kc1*
chr1	194977704	194977849	*Cd34*	*Cd46*
chr10	19293741	19293946	*Tnfaip3*	*Olig3*
chr10	19294335	19294488	*Tnfaip3*	*Olig3*

chr	start	stop	gene 1	gene 2
chr10	30781330	30781429	*Hint3*	*Ncoa7*
chr10	31413866	31413946	*Tpd52l1*	*Hddc2*
chr10	36225431	36225546	*Amd2*	*Hs3st5*
chr10	39583426	39584347	*Traf3ip2*	*Fyn*
chr10	39715865	39715986	*Rev3l*	*Traf3ip2*
chr10	40569747	40569896	*Slc22a16*	
chr10	43296756	43297673	*Bend3*	*Pdss2*
chr10	44563557	44563816	*Prep*	*Prdm1*
chr10	44564079	44564441	*Prep*	*Prdm1*
chr10	45133067	45133167	*Popdc3*	*Prep*
chr10	52767176	52767253	*Pln*	*Slc35f1*
chr10	53492261	53492543	*Cep85l*	*Asf1a*
chr10	60618744	60619827	*Cdh23*	*4632428N05Rik*
chr10	60662646	60663654	*Cdh23*	*4632428N05Rik*
chr10	60966428	60967455	*Unc5b*	*Pcbd1*
chr10	64492630	64493433	*Lrrtm3*	
chr10	68563541	68564578	*Tmem26*	*1700040L02Rik*
chr10	70949329	70950468	*4930533K18Rik*	*Bicc1*
chr10	73158341	73158886	*Pcdh15*	*Zwint*
chr10	75526433	75526527	*Snrpd3*	*Fam211b*
chr10	77837173	77837823	*Gm10318*	*Tspear*
chr10	78203767	78205578	*Pwp2*	*Trappc10*
chr10	83443024	83443936	*D10Wsu102e*	*Aldh1l2*
chr10	86248449	86248524	*Timp3*	*Fbxo7*
chr10	88252905	88252984	*Ccdc53*	*Dram1*
chr10	88937502	88937674	*Slc5a8*	*Ano4*
chr10	95316707	95318787	*Plxnc1*	*Cradd*
chr10	96391679	96392961	*Gm20091*	*Gm5426*
chr10	103313080	103313445	*Lrriq1*	*Slc6a15*
chr10	105314157	105316044	*Tmtc2*	
chr10	107561183	107561261	*Myf6*	*Ptprq*
chr10	112697209	112697392	*Atxn7l3b*	*Kcnc2*
chr10	129041945	129042049	*Olfr765*	*Olfr763*
chr10	129674638	129675601	*Olfr801*	
chr10	130543654	130544413	*Vmn2r87*	
chr11	5530606	5531525	*Xbp1*	*Ccdc117*
chr11	5534241	5534384	*Ccdc117*	*Xbp1*
chr11	5534772	5534994	*Ccdc117*	*Xbp1*
chr11	5900854	5900934	*Myl7*	
chr11	19701246	19702369	*Meis1*	*Spred2*
chr11	19834176	19834431	*Meis1*	*Spred2*
chr11	19853471	19855276	*Meis1*	*Spred2*
chr11	19876934	19880377	*Meis1*	*Spred2*
chr11	20989851	20989927	*Lgalsl*	*Peli1*
chr11	21580547	21582250	*Wdpcp*	*Otx1*
chr11	21891915	21892086	*Otx1*	*Wdpcp*
chr11	21892285	21893123	*Otx1*	*Wdpcp*
chr11	22762137	22763475	*B3gnt2*	*Tmem17*
chr11	22825696	22826348	*B3gnt2*	*Tmem17*
chr11	22974886	22975125	*Zrsr1*	*Commd1*
chr11	22975436	22975941	*Zrsr1*	*Commd1*
chr11	32101854	32103495	*Il9r*	*Nsg2*
chr11	33930514	33932001	*Kcnip1*	*Kcnmb1*
chr11	33932167	33935868	*Kcnip1*	*Kcnmb1*
chr11	36149738	36150825	*Wwc1*	
chr11	43455906	43456019	*C1qtnf2*	*Slu7*
chr11	43456287	43456383	*C1qtnf2*	*Slu7*
chr11	49592660	49592763	*Flt4*	*Olfr1380*
chr11	50262740	50263680	*Ltc4s*	*Maml1*

Supplementary Table 3. H3K4me3 differential peaks identified in F1 - *(Continued)*

chr	start	stop	gene 1	gene 2
chr11	52190074	52190260	*Olfr1373*	*Olfr1371*
chr11	52477862	52477956	*Fstl4*	*9530068E07Rik*
chr11	54508810	54508904	*Rapgef6*	*Fnip1*
chr11	54599921	54600143	*Rapgef6*	*Cdc42se2*
chr11	54600219	54600613	*Rapgef6*	*Cdc42se2*
chr11	58053265	58053355	*Cnot8*	*Larp1*
chr11	58751289	58751383	*Olfr317*	*Olfr316*
chr11	59009921	59010956	*Trim11*	*Obscn*
chr11	59302706	59302973	*Wnt9a*	
chr11	60639482	60639571	*Llgl1*	*Alkbh5*
chr11	63676075	63676224	*Hs3st3b1*	*Pmp22*
chr11	64016792	64017689	*Hs3st3b1*	*Cox10*
chr11	64725791	64725863	*Elac2*	*Hs3st3a1*
chr11	64781105	64781603	*Elac2*	*Hs3st3a1*
chr11	64781680	64781968	*Elac2*	*Hs3st3a1*
chr11	65827388	65828431	*Zkscan6*	*Dnahc9*
chr11	68522624	68523375	*Mfsd6l*	*Pik3r6*
chr11	69993403	69993538	*Phf23*	
chr11	71421940	71422643	*Wscd1*	*Nlrp1b*
chr11	72981380	72982275	*P2rx1*	*Atp2a3*
chr11	76608198	76608270	*Abr*	*Bhlha9*
chr11	77959071	77959363	*Phf12*	*Sez6*
chr11	80474884	80475291	*Cdk5r1*	
chr11	80604663	80605689	*Cdk5r1*	*Myo1d*
chr11	80766308	80767133	*Myo1d*	*Cdk5r1*
chr11	83585370	83586431	*Ccl9*	*Ccl6*
chr11	84917605	84917694	*Znhit3*	
chr11	95149732	95150314	*Dlx4*	
chr11	97222904	97223201	*Kpnb1*	*Npepps*
chr11	98068505	98068597	*Stac2*	*Fbxl20*
chr11	98860376	98862163	*Wipf2*	
chr11	99596257	99596699	*Krtap1-3*	*Krtap9-3*
chr11	99597396	99597753	*Krtap9-3*	
chr11	101172429	101172507	*Cntnap1*	*Plekhh3*
chr11	101352960	101353303	*G6pc*	*Aoc3*
chr11	104176745	104177470	*Sppl2c*	*Crhr1*
chr11	106026325	106027075	*Dcaf7*	*Kcnh6*
chr11	113174135	113174283	*Slc39a11*	*Sox9*
chr11	113561461	113562150	*Slc39a11*	*Sox9*
chr11	114198258	114199292	*Rpl38*	*Sdk2*
chr11	115266730	115266813	*Grin2c*	
chr11	116446987	116447459	*Qrich2*	*Ubald2*
chr11	117518475	117519581	*Tnrc6c*	*Gm11733*
chr11	118333567	118333702	*Usp36*	*BC100451*
chr11	119285728	119286752	*Eif4a3*	*Gaa*
chr12	5250574	5251059	*Klhl29*	*Atad2b*
chr12	9396553	9396652	*Osr1*	*Ttc32*
chr12	10095451	10095629	*Nt5c1b*	*Osr1*
chr12	24424938	24425258	*9030624G23Rik*	*Gm16372*
chr12	25488858	25488998	*Id2*	*Rnf144a*
chr12	34595285	34595875	*Hdac9*	*4921508M14Rik*
chr12	41333753	41334135	*Lrrn3*	*Immp2l*
chr12	41379337	41379429	*Lrrn3*	*Immp2l*
chr12	42016276	42017888	*Lrrn3*	
chr12	42568070	42568238		
chr12	44928743	44931037	*Stxbp6*	*Nrcam*
chr12	57950547	57952347	*Foxa1*	*Sstr1*
chr12	58966414	58966674	*Clec14a*	*Sec23a*
chr12	61994834	61994988	*Spanxn4*	*Lrfn5*
chr12	71736402	71737733	*Daam1*	*Dact1*
chr12	71962162	71963124	*Gpr135*	*Daam1*
chr12	72820737	72821883	*Ppm1a*	*4930447C04Rik*
chr12	84208688	84208928	*Ptgr2*	*Elmsan1*
chr12	85537534	85537844	*Jdp2*	*Fos*
chr12	87862654	87862759	*Gm2016*	*BB287469*
chr12	87863205	87863313	*Gm2016*	*BB287469*
chr12	100499241	100500154	*Ttc7b*	*Calm1*
chr12	101118244	101118329	*Catsperb*	*Smek1*
chr12	105518296	105518753	*Bdkrb2*	*D430019H16Rik*
chr12	108224225	108224388	*Ccnk*	*Ccdc85c*
chr12	108487670	108487844	*Evl*	*Eml1*
chr12	109921530	109922640	*Dio3*	*Rtl1*
chr12	113661896	113661976	*Adam6a*	
chr13	4124518	4124619	*Akr1c18*	*Akr1c14*
chr13	10986424	10986534		
chr13	11689037	11689157	*Ryr2*	
chr13	12540041	12540730	*Ero1lb*	*Edaradd*
chr13	17428577	17428712	*5033411D12Rik*	
chr13	17989547	17989647	*Rala*	*Yae1d1*
chr13	19887591	19888086	*Elmo1*	*Gpr141*
chr13	20646031	20646144	*Aoah*	*Elmo1*
chr13	20646300	20646391	*Aoah*	*Elmo1*
chr13	21878199	21879679	*Zfp184*	*Hist1h2br*
chr13	29782167	29782596	*Sox4*	*Cdkal1*
chr13	29988651	29989436	*E2f3*	
chr13	31065589	31065856	*Foxq1*	*Gm5447*
chr13	34227087	34227192	*Psmg4*	*Slc22a23*
chr13	37706653	37707165	*Rreb1*	*Ly86*
chr13	37847801	37848208	*Rreb1*	*Ssr1*
chr13	41240128	41240213	*Elovl2*	*Smim13*
chr13	42948976	42949229	*Tbc1d7*	*Phactr1*
chr13	46758070	46758380	*Nup153*	*Kif13a*
chr13	50341483	50341563	*Gm906*	*Fbxw17*
chr13	52015490	52016354	*Gadd45g*	*Diras2*
chr13	52757576	52757927	*Auh*	*Syk*
chr13	53603825	53604988	*Gm5449*	*Drd1a*
chr13	53776089	53776453	*Gm5449*	*Drd1a*
chr13	54019382	54019745	*Drd1a*	*Gm5449*
chr13	56748958	56749470	*Smad5*	*Trpc7*
chr13	56749522	56750117	*Smad5*	*Trpc7*
chr13	56961924	56962011	*Trpc7*	*Spock1*
chr13	57087703	57088647	*Trpc7*	*Spock1*
chr13	57698266	57698648	*Trpc7*	*Spock1*
chr13	63191846	63192178	*2010111I01Rik*	*Fancc*
chr13	63192954	63193087	*2010111I01Rik*	*Fancc*
chr13	63390838	63392093	*Fancc*	*2010111I01Rik*
chr13	63710949	63711238	*Ptch1*	*Ercc6l2*
chr13	63892599	63893000	*Ercc6l2*	*Hsd17b3*
chr13	65380062	65380159	*Gm10139*	*Zfp369*
chr13	65781307	65781399	*Gm10324*	*Gm10139*
chr13	65867179	65867290	*Gm10324*	*Gm10139*
chr13	65982971	65983101	*Gm10324*	*Gm10139*
chr13	66062466	66062543	*Gm10324*	*Gm10139*
chr13	66144879	66145006	*Gm10324*	*Gm10772*
chr13	66497928	66498126	*2410141K09Rik*	*Zfp640*
chr13	66631994	66632076	*2410141K09Rik*	*Zfp640*
chr13	66632183	66632278	*2410141K09Rik*	*Zfp640*
chr13	66640031	66640108	*2410141K09Rik*	*Zfp640*

Supplementary Table 3. H3K4me3 differential peaks identified in F1 - *(Continued)*

chr	start	stop	gene 1	gene 2
chr13	89605026	89605564	*Hapln1*	*Vcan*
chr13	90414974	90415258	*Xrcc4*	*Gm21726*
chr13	90885374	90888086	*Atp6ap1l*	*Gm21726*
chr13	92504993	92505076	*Fam151b*	*Zfyve16*
chr13	94228802	94228982	*Scamp1*	*Lhfpl2*
chr13	97290384	97291840	*Enc1*	*Gm10260*
chr13	116731254	116731359	*Isl1*	*Parp8*
chr14	3826275	3826354	*Gm3099*	*D830030K20Rik*
chr14	14782108	14782214	*Nek10*	*Slc4a7*
chr14	19754903	19756593	*Nid2*	*2700060E02Rik*
chr14	21489971	21490087	*Kat6b*	*Adk*
chr14	21744483	21744561	*Dusp13*	
chr14	21744616	21745055	*Dusp13*	
chr14	21787418	21788586	*Vdac2*	*Samd8*
chr14	21788788	21789442	*Vdac2*	*Samd8*
chr14	21789571	21789967	*Vdac2*	*Samd8*
chr14	21790051	21790180	*Vdac2*	*Samd8*
chr14	24073141	24074165	*Kcnma1*	*Dlg5*
chr14	25422394	25423200	*Zmiz1*	*Rps24*
chr14	27425155	27425264	*D14Abb1e*	
chr14	30129561	30129669	*Dcp1a*	*Chdh*
chr14	30688251	30688575	*Sfmbt1*	*Rft1*
chr14	30688648	30689177	*Sfmbt1*	*Rft1*
chr14	30689280	30690015	*Sfmbt1*	*Rft1*
chr14	31299716	31301175	*Dnahc1*	*Bap1*
chr14	36088826	36089429	*4930596D02Rik*	*Gm7853*
chr14	36089510	36089726	*4930596D02Rik*	*Gm7853*
chr14	36089975	36090047	*4930474N05Rik*	*Gm7853*
chr14	36093762	36095248	*Gm7853*	*4930474N05Rik*
chr14	41234728	41234846	*Sftpd*	*Gm7945*
chr14	41235189	41235359	*Sftpd*	*Gm7945*
chr14	41278801	41279032	*Sftpd*	*Gm7945*
chr14	41279089	41279302	*Sftpd*	*Gm7945*
chr14	41279924	41280218	*Sftpd*	*Gm7945*
chr14	41322970	41323055	*Sftpd*	*Gm7945*
chr14	41347935	41348026	*Sftpd*	*Gm7945*
chr14	41383703	41383776	*Sftpd*	*Gm7945*
chr14	41384333	41384409	*Sftpd*	*Gm7945*
chr14	41384486	41384632	*Sftpd*	*Gm7945*
chr14	41384936	41385040	*Sftpd*	*Gm7945*
chr14	41385345	41385453	*Gm7945*	
chr14	41385716	41385791	*Gm7945*	
chr14	41407306	41407408	*Gm6482*	
chr14	41407829	41407918	*Gm6482*	
chr14	41452668	41452770	*Gm6482*	*Gm3486*
chr14	41453735	41453815	*Gm6482*	*Gm3486*
chr14	41555430	41555519	*Gm7970*	
chr14	41575070	41575153	*Gm7970*	*Gm3072*
chr14	41592430	41592562	*Gm7970*	*Gm3072*
chr14	41592670	41592759	*Gm7970*	*Gm3072*
chr14	41623175	41623263	*Gm7970*	*Gm3072*
chr14	41623443	41623535	*Gm7970*	*Gm3072*
chr14	41623591	41623736	*Gm3072*	
chr14	41624002	41624097	*Gm3072*	
chr14	41645408	41645496	*Gm3072*	*Gm3676*
chr14	41646371	41646473	*Gm3676*	
chr14	41700386	41700471	*Gm3676*	*Gm7929*
chr14	41700801	41700899	*Gm3676*	*Gm7929*
chr14	41701265	41701477	*Gm3676*	*Gm7929*
chr14	41771182	41771269	*Gm7929*	*Gm7980*
chr14	41772005	41772098	*Gm7929*	*Gm7980*
chr14	41772152	41772255	*Gm7929*	*Gm7980*
chr14	41966161	41966323	*Gm6401*	*1700024B05Rik*
chr14	41981383	41981486	*Gm6401*	*Gm3543*
chr14	41983342	41983491	*Gm3543*	
chr14	41983655	41983763	*Gm3543*	
chr14	42023195	42023282	*Gm3543*	*Gm7951*
chr14	42039287	42039385	*Gm3543*	*Gm7951*
chr14	42039973	42040066	*Gm3543*	*Gm7951*
chr14	42040844	42040941	*Gm3543*	*Gm7951*
chr14	42093738	42093815	*Gm3543*	*Gm7951*
chr14	42094413	42094503	*Gm3543*	*Gm7951*
chr14	42094794	42094958	*Gm3543*	*Gm7951*
chr14	42095199	42095272	*Gm3543*	*Gm7951*
chr14	42115553	42115768	*Gm3543*	*Gm7951*
chr14	42161799	42161876	*Gm7951*	*Gm3573*
chr14	42162700	42162782	*Gm7951*	*Gm3573*
chr14	42189416	42189526	*Gm7951*	*Gm3573*
chr14	42189652	42189724	*Gm7951*	*Gm3573*
chr14	42190691	42190865	*Gm3573*	
chr14	42219598	42219670	*Gm3573*	*Gm9611*
chr14	42259129	42259225	*Gm3573*	*Gm9611*
chr14	42259339	42259463	*Gm3573*	*Gm9611*
chr14	42296194	42296400	*Gm3573*	*Gm9611*
chr14	42373224	42373312	*Gm7995*	*Gm8005*
chr14	42373486	42373569	*Gm7995*	*Gm8005*
chr14	42374084	42374170	*Gm7995*	*Gm8005*
chr14	42374478	42374573	*Gm7995*	*Gm8005*
chr14	42438246	42438339	*Gm8005*	*Gm7995*
chr14	42463784	42463968	*Gm8005*	*Gm8020*
chr14	42464488	42464581	*Gm8005*	*Gm8020*
chr14	42500472	42500546	*Gm8005*	*Gm8020*
chr14	42502279	42502362	*Gm8020*	
chr14	42523475	42523680	*Gm8024*	
chr14	42524116	42524231	*Gm8024*	
chr14	42599009	42599109	*Gm8024*	*Gm3633*
chr14	42599190	42599262	*Gm8024*	*Gm3633*
chr14	42699355	42699573	*Gm10378*	*Gm8094*
chr14	42699717	42699817	*Gm10378*	*Gm8094*
chr14	42700732	42700831	*Gm10378*	*Gm8094*
chr14	42797668	42797761	*Gm10378*	*Gm8094*
chr14	42853699	42853776	*Gm10378*	*Gm8094*
chr14	43014970	43015189	*Gm8094*	*Gm10378*
chr14	43015498	43015585	*Gm8094*	*Gm10378*
chr14	43101391	43101507	*Gm8094*	*Gm8122*
chr14	43101908	43102004	*Gm8094*	*Gm8122*
chr14	43102282	43102373	*Gm8094*	*Gm8122*
chr14	43108877	43108951	*Gm8094*	*Gm8122*
chr14	43123714	43123790	*Gm8094*	*Gm8122*
chr14	43123882	43124326	*Gm8094*	*Gm8122*
chr14	43149433	43149515	*Gm8094*	*Gm8122*
chr14	43149572	43149668	*Gm8094*	*Gm8122*
chr14	43149768	43149852	*Gm8094*	*Gm8122*
chr14	43150104	43150190	*Gm8094*	*Gm8122*
chr14	43258936	43259047	*Gm8122*	*Gm8127*
chr14	43259112	43259224	*Gm8122*	*Gm8127*
chr14	43259550	43259642	*Gm8122*	*Gm8127*
chr14	43259847	43259957	*Gm8122*	*Gm8127*

Supplementary Table 3. H3K4me3 differential peaks identified in F1 - *(Continued)*

chr	start	stop	gene 1	gene 2
chr14	43260106	43260290	*Gm8122*	*Gm8127*
chr14	43274997	43275249	*Gm8122*	*Gm8127*
chr14	43275322	43275625	*Gm8122*	*Gm8127*
chr14	43293606	43293926	*Gm8127*	
chr14	43294143	43294292	*Gm9732*	*Gm8127*
chr14	43299523	43299625	*Gm9732*	
chr14	43344973	43345048	*1700001F09Rik*	*Gm9732*
chr14	43345449	43345601	*1700001F09Rik*	*Gm9732*
chr14	43346067	43346280	*1700001F09Rik*	*Gm9732*
chr14	43346412	43346521	*1700001F09Rik*	*Gm9732*
chr14	43346948	43347058	*1700001F09Rik*	
chr14	43347200	43347309	*1700001F09Rik*	
chr14	43347450	43347560	*1700001F09Rik*	
chr14	43414919	43415025	*1700001F09Rik*	*Gm8138*
chr14	43415299	43415552	*Gm8138*	
chr14	43544212	43544306	*Gm8138*	*Gm10375*
chr14	43544527	43544674	*Gm8138*	*Gm10375*
chr14	43544960	43545058	*Gm8138*	*Gm10375*
chr14	43577690	43577786	*Gm8138*	*Gm10375*
chr14	43577978	43578229	*Gm8138*	*Gm10375*
chr14	43578401	43578630	*Gm8138*	*Gm10375*
chr14	43578773	43579007	*Gm8138*	*Gm10375*
chr14	43606904	43606986	*Gm8138*	*Gm10375*
chr14	43607284	43607377	*Gm10375*	
chr14	43607837	43607933	*Gm10375*	
chr14	43636431	43636504	*Gm10375*	*Gm8165*
chr14	43674892	43675086	*Gm10375*	*Gm8165*
chr14	43675473	43676184	*Gm10375*	*Gm8165*
chr14	43676286	43676939	*Gm8165*	
chr14	43677418	43677633	*Gm8165*	
chr14	43677732	43677825	*Gm8165*	
chr14	43677955	43678027	*Gm8165*	
chr14	43697984	43698161	*Gm8165*	*Gm16506*
chr14	43736201	43736407	*Gm16506*	*Ear1*
chr14	43747603	43747675	*Gm16506*	*Ear1*
chr14	43747742	43749293	*Gm16506*	*Ear1*
chr14	43749370	43749800	*Gm16506*	*Ear1*
chr14	43783730	43784069	*Gm16506*	*Ear1*
chr14	43784132	43784214	*Gm16506*	*Ear1*
chr14	43784350	43784600	*Gm16506*	*Ear1*
chr14	43785025	43785181	*Gm16506*	*Ear1*
chr14	43796766	43797555	*Gm16506*	*Ear1*
chr14	43799553	43799681	*Gm16506*	*Ear1*
chr14	43925452	43925615	*Ear10*	*Gm8113*
chr14	44113401	44113499	*Gm3327*	*Ear2*
chr14	44114432	44114749	*Gm3327*	*Ear2*
chr14	44124006	44124184	*Gm3327*	
chr14	44124689	44125159	*Gm3327*	*4930503E14Rik*
chr14	44125219	44125468	*Gm3327*	*4930503E14Rik*
chr14	44169419	44169702	*4930503E14Rik*	*Gm3327*
chr14	44169847	44170264	*4930503E14Rik*	*Gm3327*
chr14	44170600	44171234	*4930503E14Rik*	
chr14	44171305	44171453	*4930503E14Rik*	
chr14	44196710	44196784	*Gm8212*	
chr14	44196841	44196936	*Gm8212*	
chr14	44197316	44197431	*Gm8212*	
chr14	44197491	44197563	*Gm8212*	
chr14	44197725	44198001	*Gm8212*	
chr14	44198064	44198160	*Gm8212*	
chr14	44198231	44198568	*Gm8212*	
chr14	44199206	44199628	*Gm8220*	*Gm8212*
chr14	44212557	44213301	*Gm8220*	*Gm8212*
chr14	44285166	44285304	*Gm8220*	
chr14	44285461	44285559	*Gm8220*	
chr14	44285666	44286078	*Gm8220*	
chr14	44286168	44286472	*Gm8220*	
chr14	44286605	44286700	*Gm8229*	*Gm8220*
chr14	44286825	44287105	*Gm8229*	*Gm8220*
chr14	44287220	44287336	*Gm8229*	*Gm8220*
chr14	44287395	44287626	*Gm8229*	*Gm8220*
chr14	44311592	44311701	*Gm8229*	*Gm8220*
chr14	44311939	44312028	*Gm8229*	*Gm8220*
chr14	44312107	44312302	*Gm8229*	*Gm8220*
chr14	44364844	44365464	*Gm8229*	
chr14	44365589	44365876	*Gm8229*	
chr14	44365982	44366687	*Gm8229*	
chr14	44366771	44366878	*Gm8229*	*Gm3371*
chr14	44367024	44367399	*Gm8229*	*Gm3371*
chr14	44384136	44384315	*Gm8229*	*Gm3371*
chr14	44384369	44384468	*Gm8229*	*Gm3371*
chr14	44433133	44433361	*Gm8232*	
chr14	44433532	44433732	*Gm8232*	
chr14	44433898	44434267	*Gm8232*	
chr14	44434409	44434775	*Gm8232*	
chr14	44434899	44435118	*BC061237*	*Gm8232*
chr14	44435198	44435711	*BC061237*	*Gm8232*
chr14	44500265	44500594	*BC061237*	
chr14	44500654	44501087	*BC061237*	
chr14	44501245	44501761	*Gm8247*	*BC061237*
chr14	44501884	44502578	*Gm8247*	*BC061237*
chr14	44518562	44518644	*Gm8247*	*BC061237*
chr14	44519035	44519199	*Gm8247*	*BC061237*
chr14	44519414	44519523	*Gm8247*	*BC061237*
chr14	44582731	44583178	*Gm8247*	
chr14	44583335	44583543	*Gm8247*	
chr14	44583685	44584066	*Gm8247*	
chr14	44584352	44585478	*Gm8247*	*Gm8267*
chr14	44613111	44613184	*Gm8247*	*Gm8267*
chr14	44613347	44613590	*Gm8247*	*Gm8267*
chr14	44613662	44614120	*Gm8247*	*Gm8267*
chr14	44614355	44614685	*Gm8247*	*Gm8267*
chr14	44614957	44615053	*Gm8247*	*Gm8267*
chr14	44615199	44615624	*Gm8247*	*Gm8267*
chr14	44627606	44627963	*Gm8247*	*Gm8267*
chr14	44656042	44656564	*Gm8267*	*Gm8247*
chr14	44656861	44657202	*Gm8267*	*Gm8247*
chr14	44685968	44686149	*Gm8267*	*Gm8247*
chr14	44686201	44686277	*Gm8267*	*Gm8247*
chr14	44686367	44686681	*Gm8267*	*Gm8247*
chr14	44686741	44686964	*Gm8267*	*Gm8247*
chr14	44687044	44687827	*Gm8267*	*Gm8247*
chr14	44770274	44770377	*Gm8267*	*Ptgdr*
chr14	44790397	44790527	*Gm8267*	*Ptgdr*
chr14	44926791	44926918	*Ptgdr*	*Ptger2*
chr14	44948704	44948895	*Ptgdr*	*Ptger2*
chr14	44948952	44949300	*Ptgdr*	*Ptger2*
chr14	46415525	46415603	*Cdkn3*	*Bmp4*
chr14	47993252	47993719	*Peli2*	*Ktn1*

Supplementary Table 3. H3K4me3 differential peaks identified in F1 - *(Continued)*

chr	start	stop	gene 1	gene 2
chr14	48073229	48074062	*Peli2*	*Ktn1*
chr14	49238254	49241134	*1700011H14Rik*	*Naa30*
chr14	49243111	49243485	*1700011H14Rik*	*Naa30*
chr14	49828443	49829152	*3632451O06Rik*	*Olfr722*
chr14	50378896	50380601	*Olfr736*	*Tlr11*
chr14	50380659	50382413	*Olfr736*	*Tlr11*
chr14	51007713	51009043	*Rnase10*	
chr14	51182174	51182533	*Ang2*	*Ear5*
chr14	51343898	51344324	*Gm7247*	*Gm21718*
chr14	51344435	51346040	*Gm7247*	*Gm21718*
chr14	51363694	51363767	*Vmn2r88*	*Gm7247*
chr14	51363989	51364111	*Vmn2r88*	*Gm7247*
chr14	51381809	51382100	*Vmn2r88*	*Gm7247*
chr14	51382216	51383474	*Vmn2r88*	*Gm7247*
chr14	51572076	51572586	*Gm5622*	*Gm4181*
chr14	51572868	51572973	*Gm5622*	*Gm4181*
chr14	51573101	51573192	*Gm5622*	*Gm4181*
chr14	51574689	51575521	*Gm5622*	*Gm4181*
chr14	51591475	51591583	*Gm5622*	*Gm4181*
chr14	51591791	51592038	*Gm5622*	*Gm4181*
chr14	51592290	51592504	*Gm5622*	*Gm4181*
chr14	51592739	51592899	*Gm5622*	*Gm4181*
chr14	51593153	51593275	*Gm5622*	*Gm4181*
chr14	51611197	51611655	*Gm4181*	*Gm5622*
chr14	51612200	51612306	*Gm4181*	*Gm5622*
chr14	51633576	51633748	*Gm4181*	*Gm5622*
chr14	51634094	51634289	*Gm4181*	*Gm5622*
chr14	51635143	51635299	*Gm4181*	
chr14	51635901	51635979	*Gm4181*	
chr14	51677392	51678009	*Gm4181*	*Gm5800*
chr14	51692800	51692960	*Gm4181*	*Gm5800*
chr14	51693714	51693788	*Gm4181*	*Gm5800*
chr14	51694232	51694331	*Gm4181*	*Gm5800*
chr14	51695676	51695970	*Gm4181*	*Gm5800*
chr14	51748663	51748747	*Gm5800*	*Ang4*
chr14	53715790	53716086	*Dad1*	*Trav9n-1*
chr14	54780000	54780489	*Cebpe*	*Slc7a8*
chr14	55152571	55153722	*Dhrs2*	*Jph4*
chr14	56484336	56486183	*Rnf17*	*Cenpj*
chr14	56832228	56833156	*Zmym2*	*Zmym5*
chr14	56886514	56886598	*Zmym2*	
chr14	57545562	57545684	*Il17d*	*N6amt2*
chr14	66785203	66785550	*Gm10032*	
chr14	68103170	68103259	*Nefl*	*Nefm*
chr14	70588684	70588803	*Nudt18*	*Fam160b2*
chr14	72559072	72559145	*Fndc3a*	
chr14	75830433	75830506	*Slc25a30*	*Tpt1*
chr14	76425801	76426730	*Tsc22d1*	*Serp2*
chr14	76736821	76737745	*Serp2*	*Lacc1*
chr14	78332806	78333261	*Tnfsf11*	*Akap11*
chr14	84016667	84018188	*Pcdh17*	
chr14	84018491	84018856	*Pcdh17*	
chr14	84073107	84075274	*Pcdh17*	
chr14	84075463	84077318	*Pcdh17*	
chr14	84309955	84310041	*Pcdh17*	
chr14	84794675	84795508	*Pcdh17*	
chr14	86421865	86422212	*Diap3*	
chr14	96585960	96586213	*Klhl1*	
chr14	97051875	97053042	*Klhl1*	

chr	start	stop	gene 1	gene 2
chr14	97063200	97063529	*Klhl1*	
chr14	98672567	98673025	*Dach1*	*Mzt1*
chr14	99176580	99176710	*Klf5*	*Dis3*
chr14	102968940	102969020	*Irg1*	
chr14	105218188	105218288	*Rbm26*	*Ndfip2*
chr14	106464593	106464844	*Trim52*	
chr14	120317178	120317715	*Rap2a*	*Mbnl2*
chr15	8595426	8595516	*Gm2310*	*Slc1a3*
chr15	9093025	9096593	*Nadkd1*	*Skp2*
chr15	35436908	35436996	*Vps13b*	*Cox6c*
chr15	35932855	35933663	*Cox6c*	*Vps13b*
chr15	38405609	38405710	*Klf10*	*Azin1*
chr15	38696604	38697716	*Baalc*	*Atp6v1c1*
chr15	53274622	53274752	*Ext1*	*Med30*
chr15	60992367	60993108	*Myc*	*A1bg*
chr15	63918533	63918608	*Gsdmc4*	*Fam49b*
chr15	66694476	66695740	*Tg*	*Sla*
chr15	73208438	73209099	*Ago2*	*Ptk2*
chr15	73496236	73496374	*Ptk2*	*Dennd3*
chr15	74653698	74655236	*Mroh4*	*Arc*
chr15	76046148	76047158	*Fam83h*	*Scrib*
chr15	76501086	76502517	*Dgat1*	*Hsf1*
chr15	76782894	76784169	*C030006K11Rik*	*Gm17271*
chr15	77193977	77194410	*Rbfox2*	*Apol6*
chr15	77468932	77469029	*Apol7b*	*Apol10a*
chr15	77786773	77787896	*Apol8*	*Myh9*
chr15	79509897	79510615	*Kdelr3*	*Kcnj4*
chr15	79721103	79721756	*Sun2*	*Gtpbp1*
chr15	80193746	80193851	*Smcr7l*	*Mgat3*
chr15	80194033	80194120	*Smcr7l*	*Mgat3*
chr15	84202718	84204064	*Parvb*	*Samm50*
chr15	84905336	84907444	*Phf21b*	*Nup50*
chr15	84907500	84908321	*Phf21b*	*Nup50*
chr15	84912749	84912877	*Phf21b*	*Nup50*
chr15	84916112	84916476	*Phf21b*	*Nup50*
chr15	84917771	84918539	*Phf21b*	*Nup50*
chr15	85063183	85063447	*Gm10923*	*Fam118a*
chr15	86414224	86415378	*Tbc1d22a*	
chr15	86582134	86582355	*Tbc1d22a*	
chr15	88595929	88596861	*Zdhhc25*	
chr15	94814398	94815438	*Tmem117*	*Nell2*
chr15	94817748	94818118	*Tmem117*	*Nell2*
chr15	97458414	97458594	*Amigo2*	*Rpap3*
chr15	97743179	97743341	*Endou*	*Rapgef3*
chr15	98037053	98037176	*Col2a1*	*Senp1*
chr15	99057063	99057164	*Troap*	*Prph*
chr15	99416366	99416502	*Tmbim6*	*Nckap5l*
chr15	99417834	99418614	*Tmbim6*	*Nckap5l*
chr15	102259618	102259788	*Rarg*	
chr15	102428048	102428141	*Amhr2*	*Sp1*
chr16	3542114	3543122	*Olfr161*	
chr16	3974690	3974774	*Nlrc3*	*Cluap1*
chr16	11539282	11539756	*Gm9961*	*Snx29*
chr16	12688216	12688619	*Gm6327*	*Shisa9*
chr16	14250163	14251038	*Myh11*	*Nde1*
chr16	17596352	17597219	*Smpd4*	*Slc7a4*
chr16	17858074	17859034	*Car15*	*Gm20518*
chr16	18355329	18356137	*Arvcf*	*Comt*
chr16	21352898	21354117	*Vps8*	*Ephb3*

Supplementary Table 3. H3K4me3 differential peaks identified in F1 - *(Continued)*

chr	start	stop	gene 1	gene 2
chr16	30524239	30524357	*Atp13a3*	*Tmem44*
chr16	31547348	31548403	*Dlg1*	*Bdh1*
chr16	35167602	35167817	*Adcy5*	*Sec22a*
chr16	36032042	36032200	*Wdr5b*	*Kpna1*
chr16	38014924	38018914	*Gsk3b*	*Gpr156*
chr16	38415914	38417299	*Pla1a*	*Popdc2*
chr16	39180632	39181003	*Igsf11*	
chr16	41548211	41548840	*Lsamp*	*Gap43*
chr16	45031928	45032154	*Ccdc80*	*Cd200r3*
chr16	45174276	45174643	*Btla*	*Atg3*
chr16	48189836	48190992	*Dppa4*	*Gm7275*
chr16	48425418	48425491	*Morc1*	*Dppa2*
chr16	56059347	56059465	*Trmt10c*	*Senp7*
chr16	58578367	58578789	*St3gal6*	*Gm813*
chr16	69868259	69868346	*Speer2*	
chr16	86747789	86750521	*Adamts5*	*N6amt1*
chr16	87573845	87574621	*Bach1*	*Map3k7cl*
chr16	87574803	87577631	*Bach1*	*Map3k7cl*
chr16	91774234	91774329	*Itsn1*	*Atp5o*
chr16	92173588	92174633	*Kcne2*	*Slc5a3*
chr16	97543237	97544083	*Mx2*	*Tmprss2*
chr16	97778338	97778415	*Ripk4*	*Prdm15*
chr16	98082665	98082742	*B230307C23Rik*	
chr17	3313986	3314069	*Tiam2*	*Scaf8*
chr17	3662466	3662980	*Tfb1m*	*Nox3*
chr17	3663413	3663497	*Tfb1m*	*Nox3*
chr17	5267861	5268568	*Tmem242*	*Arid1b*
chr17	5268687	5269718	*Tmem242*	*Arid1b*
chr17	5829576	5829948	*Snx9*	*Zdhhc14*
chr17	6493646	6493743	*Dynlt1b*	*Gm2792*
chr17	6947536	6947639	*Rsph3b*	
chr17	6962732	6962830	*Tagap1*	
chr17	7466172	7468502	*Gm9992*	*Fndc1*
chr17	7468830	7469424	*Gm9992*	*Fndc1*
chr17	7985759	7985840	*Rsph3a*	*Rnaset2a*
chr17	7986475	7986643	*Rsph3a*	*Rnaset2a*
chr17	7987023	7987114	*Rsph3a*	*Rnaset2a*
chr17	8147883	8147988	*Rnaset2a*	
chr17	8148354	8148531	*Rnaset2a*	
chr17	8166909	8167154	*Ccr6*	*Fgfr1op*
chr17	8468497	8469829	*Pde10a*	*T*
chr17	8580945	8581710	*Pde10a*	*T*
chr17	8653730	8655919	*Pde10a*	*T*
chr17	8657682	8659360	*Pde10a*	*T*
chr17	10198524	10199579	*Pabpc6*	*Qk*
chr17	10279302	10280076	*Pabpc6*	*Qk*
chr17	10280136	10280426	*Pabpc6*	*Qk*
chr17	10323370	10323967	*Qk*	*Qk*
chr17	10325502	10325669	*Qk*	*Pacrg*
chr17	11143810	11145681	*Park2*	*Park2*
chr17	11298585	11298666	*Park2*	*Park2*
chr17	11325552	11325819	*Park2*	*Park2*
chr17	11326163	11326445	*Park2*	*Park2*
chr17	13104651	13104907	*Unc93a*	*Tcp10b*
chr17	13104983	13105178	*Unc93a*	*Tcp10b*
chr17	13106288	13106418	*Unc93a*	*Tcp10b*
chr17	13131624	13131706	*Unc93a*	
chr17	23945564	23945680	*Sbp*	*Sbpl*
chr17	28750395	28751180	*Mapk13*	*Mapk14*

chr	start	stop	gene 1	gene 2
chr17	28833449	28833531	*Pnpla1*	*Brpf3*
chr17	29072272	29074617	*Cdkn1a*	*Srsf3*
chr17	30647815	30648762	*Glp1r*	*Dnahc8*
chr17	30888594	30888706	*Glp1r*	*Dnahc8*
chr17	31231268	31233320	*Ubash3a*	*Rsph1*
chr17	31366464	31366710	*Pde9a*	*Slc37a1*
chr17	31366838	31367571	*Pde9a*	*Slc37a1*
chr17	31411710	31412628	*Pde9a*	*Wdr4*
chr17	31471637	31473014	*Wdr4*	*Pde9a*
chr17	31943173	31944561	*Sik1*	*Hsf2bp*
chr17	32051114	32052567	*Rrp1b*	*Notch3*
chr17	33875124	33875230	*Kifc1*	*Cd320*
chr17	37563339	37563581	*Olfr112*	*Olfr111*
chr17	42455005	42455103	*Ptchd4*	*Opn5*
chr17	42455574	42455796	*Ptchd4*	*Opn5*
chr17	44180109	44181524	*Enpp4*	*Clic5*
chr17	45661199	45661287	*Capn11*	
chr17	46537759	46538803	*Cul9*	*Dnph1*
chr17	46778796	46778884	*Rpl7l1*	*2310039H08Rik*
chr17	47174728	47175307	*Mrps10*	*Trerf1*
chr17	47446355	47447258	*1700001C19Rik*	*Taf8*
chr17	48598610	48600182	*Lrfn2*	*Unc5cl*
chr17	56388105	56388267	*Kdm4b*	*Ptprs*
chr17	56686215	56686756	*Ranbp3*	*Vmac*
chr17	57891921	57892031	*Cntnap5c*	
chr17	62715862	62715940	*Efna5*	*Efna5*
chr17	63962597	63962886	*Fer*	*Pja2*
chr17	64137872	64138884	*Pja2*	*Fer*
chr17	75695549	75696757	*Fam98a*	
chr17	80113990	80114062	*Hnrpll*	*Galm*
chr17	80114911	80115720	*Hnrpll*	*Galm*
chr17	82955429	82957273	*Pkdcc*	*Gm6594*
chr17	82957340	82957724	*Pkdcc*	*Gm6594*
chr17	84547736	84547846	*Dync2li1*	*Plekhh2*
chr17	87012178	87012442	*Pigf*	*Rhoq*
chr17	87014347	87014481	*Pigf*	*Rhoq*
chr17	87257904	87258423	*Mcfd2*	*Socs5*
chr17	87258790	87260022	*Mcfd2*	*Socs5*
chr18	4320605	4320783	*Map3k8*	*Lyzl1*
chr18	16463548	16465148	*Cdh2*	*Gm10036*
chr18	16766786	16766980	*Cdh2*	*Gm10036*
chr18	16767045	16767983	*Cdh2*	*Gm10036*
chr18	18483634	18483869		
chr18	18567579	18567980		
chr18	18568072	18568483		
chr18	24726772	24727215	*Fhod3*	*Tpgs2*
chr18	36919423	36919502	*Gm10545*	*Zmat2*
chr18	39319019	39319140	*Nr3c1*	*Arhgap26*
chr18	39595202	39595334	*Pabpc2*	*Nr3c1*
chr18	43195072	43195144	*Ppp2r2b*	*Stk32a*
chr18	47913556	47913665	*Sema6a*	*Gm5506*
chr18	55859863	55860042	*Zfp608*	*Gramd3*
chr18	58691286	58691359	*Adamts19*	*Isoc1*
chr18	60593657	60593752	*Myoz3*	
chr18	61413101	61413195	*Ppargc1b*	*Arhgef37*
chr18	62568686	62569022	*Spink10*	*Spink7*
chr18	70052867	70053797	*Rab27b*	*Ccdc68*
chr18	72096578	72096746	*Dcc*	
chr18	72222914	72223098	*Dcc*	

Supplementary Table 3. H3K4me3 differential peaks identified in F1 - *(Continued)*

chr	start	stop	gene 1	gene 2
chr19	6404588	6405606	*Nrxn2*	*Rasgrp2*
chr19	9836734	9836832	*Stxbp3b*	*Scgb2a2*
chr19	10051921	10052845	*Fads3*	*Fads2*
chr19	10520220	10520809	*Cpsf7*	*Sdhaf2*
chr19	10669544	10670393	*Pga5*	*Vwce*
chr19	15972263	15973445	*Psat1*	*Cep78*
chr19	18274224	18274748	*Pcsk5*	*Ostf1*
chr19	21804329	21804854	*Trpm3*	*Tmem2*
chr19	25628854	25628960	*Dmrt2*	*Dmrt3*
chr19	31126529	31127638	*Cstf2t*	*Prkg1*
chr19	32799873	32800058	*Pten*	*Rnls*
chr19	33390658	33390749	*Rnls*	*Pten*
chr19	36427837	36428947	*Hectd2*	*Pcgf5*
chr19	38263151	38263266	*Lgi1*	
chr19	42100176	42100441	*Pi4k2a*	*Avpi1*
chr19	42223069	42223510	*Golga7b*	*Sfrp5*
chr19	43267527	43268977	*Hps1*	*Hpse2*
chr19	56774952	56775044	*A630007B06Rik*	*Adrb1*
chr19	60539451	60540328	*Prlhr*	*Cacul1*
chr19	61084874	61085308	*Gm7102*	*Grk5*
chr19	61152702	61152794	*Gm7102*	*Grk5*
chr19	61153130	61153220	*Gm7102*	*Grk5*
chr19	61153890	61154078	*Gm7102*	*Grk5*
chr2	4522194	4522267	*Prpf18*	*Frmd4a*
chr2	22067360	22068004	*Myo3a*	*Gpr158*
chr2	22069726	22070117	*Myo3a*	*Gpr158*
chr2	23012194	23012335	*Abi1*	*Pdss1*
chr2	23015004	23016641	*Abi1*	*Pdss1*
chr2	23694816	23694895	*Spopl*	*Hnmt*
chr2	24625157	24626487	*Pax8*	*Cacna1b*
chr2	26395577	26395803	*Pmpca*	*Inpp5e*
chr2	28868203	28869474	*Ddx31*	*Barhl1*
chr2	29000112	29000193	*Barhl1*	*1700101E01Rik*
chr2	29127176	29127260	*Setx*	*Ntng2*
chr2	29975470	29976621	*Sptan1*	*Wdr34*
chr2	36023679	36024526	*Ttll11*	*Ndufa8*
chr2	36184586	36184680	*Rbm18*	*Ptgs1*
chr2	38136954	38138561	*Dennd1a*	*Crb2*
chr2	38571494	38571568	*Nek6*	*Psmb7*
chr2	39116753	39117848	*Golga1*	*Scai*
chr2	59379060	59379140	*Dapl1*	*Pkp4*
chr2	71822438	71822569	*Pdk1*	*Itga6*
chr2	71838264	71838446	*Pdk1*	*Itga6*
chr2	76528483	76528556	*Prkra*	*Osbpl6*
chr2	77364290	77364377	*Sestd1*	*Zfp385b*
chr2	77897522	77901041	*Zfp385b*	*Cwc22*
chr2	77901196	77901949	*Zfp385b*	*Cwc22*
chr2	77902071	77902776	*Zfp385b*	*Cwc22*
chr2	77918997	77919655	*Zfp385b*	*Cwc22*
chr2	77924980	77926668	*Zfp385b*	*Cwc22*
chr2	77944303	77947084	*Cwc22*	
chr2	77947145	77947689	*Cwc22*	
chr2	77947774	77948277	*Cwc22*	
chr2	78010851	78012436	*Ube2e3*	*Cwc22*
chr2	78012535	78012744	*Ube2e3*	*Cwc22*
chr2	79678832	79680495	*Ppp1r1c*	*Ssfa2*
chr2	81857094	81857525	*Zfp804a*	
chr2	86900641	86900776	*Olfr1097*	*Olfr1098*
chr2	87322966	87323095	*Olfr1120*	*Olfr1118*

chr	start	stop	gene 1	gene 2
chr2	91490840	91490959	*Ckap5*	*Lrp4*
chr2	92099508	92099582	*Phf21a*	*Creb3l1*
chr2	92124022	92124303	*Creb3l1*	*Phf21a*
chr2	92655357	92656145	*Syt13*	*Chst1*
chr2	93448041	93448960	*Tspan18*	*Cd82*
chr2	94286213	94287017	*Hsd17b12*	*Ttc17*
chr2	98107922	98108935	*Gm10801*	
chr2	98979365	98982132	*Gm10800*	
chr2	99483580	99483689	*Gm10800*	
chr2	119673023	119673152	*Rtf1*	
chr2	125530439	125530682	*Fbn1*	*Cep152*
chr2	126130864	126131744	*Fam227b*	*Fgf7*
chr2	128178999	128179814	*Gm355*	*Bcl2l11*
chr2	132161528	132162340	*Slc23a2*	*Tmem230*
chr2	132656935	132657073	*Gpcpd1*	*1110034G24Rik*
chr2	135932687	135932761	*Lamp5*	*Plcb4*
chr2	136945715	136946876	*Slx4ip*	*Jag1*
chr2	140919291	140920579	*Flrt3*	
chr2	141103614	141103692	*Flrt3*	
chr2	142826909	142827938	*Kif16b*	
chr2	144206675	144209505	*Mgme1*	*Banf2*
chr2	144739734	144740711	*Scp2d1*	*Dtd1*
chr2	146282651	146283152	*Insm1*	*Ralgapa2*
chr2	147248978	147249147	*Pax1*	*6430503K07Rik*
chr2	148822190	148822345	*Cst9*	*Cst13*
chr2	150589336	150590507	*Apmap*	*Cst7*
chr2	151095451	151095611	*Gm14151*	*Gm14147*
chr2	151095664	151095807	*Gm14151*	*Gm14147*
chr2	151301070	151301259	*4921509C19Rik*	*Gm14147*
chr2	151301405	151301561	*4921509C19Rik*	*Gm14147*
chr2	151302321	151302410	*4921509C19Rik*	*Gm14147*
chr2	151432643	151432732	*4921509C19Rik*	*Gm14147*
chr2	151748313	151748389	*Rspo4*	*Psmf1*
chr2	153426337	153426575	*4930404H24Rik*	*Asxl1*
chr2	154887048	154887287	*Eif2s2*	*C130057N11Rik*
chr2	155538988	155539061	*Acss2*	*Gss*
chr2	155781927	155782207	*Mmp24*	*Eif6*
chr2	155782342	155783252	*Mmp24*	*Eif6*
chr2	155783310	155785302	*Mmp24*	*Eif6*
chr2	156003653	156004364	*Ergic3*	*6430550D23Rik*
chr2	156310973	156311169	*Cnbd2*	
chr2	156311229	156311646	*Cnbd2*	
chr2	156314369	156315062	*Scand1*	
chr2	156391806	156391888	*Scand1*	*Epb4.1l1*
chr2	156392021	156392130	*Scand1*	*Epb4.1l1*
chr2	156392907	156393823	*Scand1*	*Epb4.1l1*
chr2	156682391	156684196	*Myl9*	*Dlgap4*
chr2	156779834	156780892	*Tgif2*	*Myl9*
chr2	156781559	156781640	*Tgif2*	*Myl9*
chr2	156810125	156810525	*Tgif2*	*Myl9*
chr2	157347599	157348369	*Ghrh*	
chr2	157596775	157597293	*Ctnnbl1*	*Blcap*
chr2	157597345	157597457	*Ctnnbl1*	*Blcap*
chr2	157597632	157597846	*Ctnnbl1*	*Blcap*
chr2	157806074	157806675	*Vstm2l*	*Ctnnbl1*
chr2	157937354	157939663	*Vstm2l*	*Tti1*
chr2	157940163	157941577	*Vstm2l*	*Tti1*
chr2	157988953	157990355	*Tti1*	*Vstm2l*
chr2	158841032	158841115	*Dhx35*	

Supplementary Table 3. H3K4me3 differential peaks identified in F1 - *(Continued)*

chr	start	stop	gene 1	gene 2
chr2	161247854	161248851	*Chd6*	
chr2	162911723	162912586	*Ptprt*	*Srsf6*
chr2	164813766	164814005	*Zswim1*	*Zswim3*
chr2	168433956	168434199	*Kcng1*	*Nfatc2*
chr2	169632857	169632931	*Tshz2*	
chr2	180875322	180876215	*Bhlhe23*	*Ythdf1*
chr2	181336926	181338117	*Zgpat*	*Rtel1*
chr2	181811354	181812877	*Pcmtd2*	*Myt1*
chr3	7415871	7418077	*Zc2hc1a*	*Pkia*
chr3	7964294	7964907	*Stmn2*	*Il7*
chr3	18291223	18291365	*Cyp7b1*	*Armc1*
chr3	18480282	18480482	*Cyp7b1*	*Armc1*
chr3	22131514	22134556	*Tbl1xr1*	
chr3	22299842	22303763	*Tbl1xr1*	
chr3	22413525	22413598	*Tbl1xr1*	
chr3	37773148	37773250	*Gm5148*	*Ankrd50*
chr3	57696668	57696930	*Gm410*	
chr3	61943713	61944611	*B430305J03Rik*	*Arhgef26*
chr3	61944753	61944986	*B430305J03Rik*	*Arhgef26*
chr3	64932090	64932297	*Vmn2r7*	*Kcnab1*
chr3	73532286	73532837	*Slitrk3*	*Bche*
chr3	75183415	75183547	*Zbbx*	*Serpini2*
chr3	76578189	76578423	*Fstl5*	
chr3	76731885	76732303	*Fstl5*	
chr3	78925295	78925478	*Gm5277*	*Rapgef2*
chr3	87184048	87184326	*Kirrel*	*Fcrls*
chr3	87595997	87597607	*Arhgef11*	*Etv3*
chr3	87758250	87759923	*Lrrc71*	*Pear1*
chr3	89760578	89761995	*Chrnb2*	*Adar*
chr3	95395268	95395995	*Setdb1*	*Gm5070*
chr3	95410009	95410390	*Gm5070*	
chr3	95410679	95410893	*Gm5070*	
chr3	95411032	95412387	*Gm5070*	
chr3	95734762	95736097	*Adamtsl4*	*Ecm1*
chr3	95945677	95946388	*Anp32e*	*Plekho1*
chr3	96176110	96176658	*Sv2a*	
chr3	96400818	96400890	*Fcgr1*	*BC107364*
chr3	96964952	96965303	*Gja5*	*Gja8*
chr3	100183916	100183999	*Wdr3*	*Fam46c*
chr3	100184079	100184607	*Wdr3*	*Fam46c*
chr3	105070224	105071149	*Kcnd3*	*4930564D02Rik*
chr3	107427102	107428199	*Rbm15*	*Kcnc4*
chr3	107616063	107617705	*Gm10961*	*Alx3*
chr3	108992175	108993454	*Gm9857*	*Fam102b*
chr3	117458931	117460088	*4833424O15Rik*	*D3Bwg0562e*
chr3	120490240	120490324	*Ptbp2*	*Rwdd3*
chr3	136482841	136483265	*Ppp3ca*	*Bank1*
chr3	144399764	144399841	*Lmo4*	*Hs2st1*
chr3	155086037	155086120	*Tnni3k*	*Fpgt*
chr4	12026436	12026518	*Gm10604*	*Tmem67*
chr4	15241466	15241538	*Necab1*	*Tmem64*
chr4	21693020	21693545	*Ccnc*	*Prdm13*
chr4	32964765	32964873	*Ankrd6*	*Rragd*
chr4	34942057	34942142	*Ifnk*	*Cga*
chr4	37083471	37083548		
chr4	40668991	40669068	*Aptx*	*Tmem215*
chr4	40669246	40669392	*Aptx*	*Tmem215*
chr4	43261585	43261808	*Atp8b5*	*Unc13b*
chr4	53524422	53525061	*Fsd1l*	*Slc44a1*
chr4	63019293	63019971	*Zfp618*	*Ambp*
chr4	65126472	65126544	*Trim32*	*Pappa*
chr4	65307544	65307802	*Trim32*	*Pappa*
chr4	65823241	65823446	*Trim32*	*Astn2*
chr4	71795866	71796049	*Gm11232*	*Tle1*
chr4	84710973	84711590	*Cntln*	*Bnc2*
chr4	96396144	96396224	*Cyp2j11*	*Cyp2j8*
chr4	96396358	96396571	*Cyp2j11*	*Cyp2j8*
chr4	100033326	100035419	*Ror1*	*Pgm2*
chr4	100904678	100905241	*Gm10577*	*Cachd1*
chr4	101329411	101330331	*Ak4*	*Jak1*
chr4	104354437	104354555	*Dab1*	*Gm10304*
chr4	107512934	107513086	*Glis1*	*Dmrtb1*
chr4	107976377	107980153	*Slc1a7*	*Podn*
chr4	109217809	109218645	*Calr4*	*Osbpl9*
chr4	115970366	115970901	*Dmbx1*	*Faah*
chr4	120242455	120244244	*Foxo6*	*Edn2*
chr4	120538569	120539219	*Slfnl1*	*Ctps*
chr4	120582443	120583587	*Ctps*	*Gm8439*
chr4	123875881	123875953	*Mycbp*	*4933427I04Rik*
chr4	128378014	128378094	*Hmgb4*	*Zscan20*
chr4	128772619	128773439	*A3galt2*	*Zfp362*
chr4	132701598	132701843	*Xkr8*	*Eya3*
chr4	134252419	134252664	*Zfp593*	*Grrp1*
chr4	134392442	134392842	*Pafah2*	
chr4	135097930	135100487	*Runx3*	*Syf2*
chr4	135299485	135299835	*Clic4*	*Srrm1*
chr4	135355316	135356101	*Srrm1*	
chr4	137472697	137474259	*Hspg2*	*Ldlrad2*
chr4	137561709	137562790	*Ldlrad2*	*Hspg2*
chr4	137609760	137609894	*Rap1gap*	*Usp48*
chr4	137787589	137788100	*Alpl*	*Rap1gap*
chr4	137830295	137830533	*Alpl*	*Ece1*
chr4	138361517	138361664	*Pink1*	*Cda*
chr4	138361758	138362093	*Pink1*	*Cda*
chr4	139364512	139366278	*Ubr4*	*Emc1*
chr4	140567459	140568157	*Gm9867*	*Arhgef10l*
chr4	140902291	140902933	*Padi2*	
chr4	143324651	143324734	*Pdpn*	*Lrrc38*
chr4	144978207	144979495	*Vps13d*	
chr4	145298269	145302117	*Tnfrsf1b*	*Tnfrsf8*
chr4	145573983	145574212	*Gm13212*	*Gm13225*
chr4	146206795	146206871	*Gm13251*	*Zfp600*
chr4	146263525	146263615	*Gm13251*	*Zfp600*
chr4	147124521	147124620	*Gm13139*	*Rex2*
chr4	147219393	147219652	*Gm13151*	*Gm13139*
chr4	147219832	147219937	*Gm13151*	*Gm13139*
chr4	147274665	147274810	*Gm13151*	*Gm13139*
chr4	147275048	147275152	*Gm13151*	*Gm13139*
chr4	147398450	147398531	*Gm13152*	*Gm13145*
chr4	147401440	147401681	*Gm13152*	*Gm13145*
chr4	148165635	148166075	*Fbxo2*	*Ptchd2*
chr4	148457892	148458817	*Mtor*	*Angptl7*
chr4	149548607	149549266	*Clstn1*	*Ctnnbip1*
chr4	151334974	151335542	*Gm13090*	*Camta1*
chr5	3691446	3693161	*4930511M11Rik*	*Ankib1*
chr5	3730881	3733108	*Ankib1*	*4930511M11Rik*
chr5	3896921	3897018	*Mterf*	
chr5	4191778	4192192	*Gm9897*	

Supplementary Table 3. H3K4me3 differential peaks identified in F1 - *(Continued)*

chr	start	stop	gene 1	gene 2
chr5	5076906	5077000	*Fzd1*	*Cdk14*
chr5	5504555	5505379	*1700015F17Rik*	*Cldn12*
chr5	5795909	5796354	*Steap1*	
chr5	5796453	5796543	*Steap1*	
chr5	5796676	5796766	*Steap1*	
chr5	6315128	6315246	*Steap1*	*Zfp804b*
chr5	6760553	6760891	*Zfp804b*	
chr5	7096569	7097503	*Zfp804b*	*Tubb4b-ps1*
chr5	7098207	7099303	*Zfp804b*	*Tubb4b-ps1*
chr5	7099360	7100488	*Zfp804b*	*Tubb4b-ps1*
chr5	7100759	7102205	*Zfp804b*	*Tubb4b-ps1*
chr5	7622254	7622350	*Steap4*	*4921511H03Rik*
chr5	10253099	10253190	*Gm5861*	*Gm10482*
chr5	10827158	10828934	*Gm5861*	*Gm10482*
chr5	10945905	10945988	*Gm5861*	*Gm10482*
chr5	10948630	10948766	*Gm5861*	*Gm10482*
chr5	10948962	10949070	*Gm5861*	*Gm10482*
chr5	10949957	10950093	*Gm5861*	*Gm10482*
chr5	11041586	11041676	*Gm5861*	*Gm10482*
chr5	11126473	11126576	*Gm5861*	*Gm10482*
chr5	11126962	11127062	*Gm5861*	*Gm10482*
chr5	11253630	11253954	*Gm6460*	*Gm5861*
chr5	11254169	11254552	*Gm6460*	*Gm5861*
chr5	11254742	11254876	*Gm6460*	*Gm5861*
chr5	11255148	11255240	*Gm6460*	*Gm5861*
chr5	11255479	11255654	*Gm6460*	*Gm5861*
chr5	11256175	11256271	*Gm6460*	*Gm5861*
chr5	11256359	11256455	*Gm6460*	*Gm5861*
chr5	11339946	11340053	*Gm6460*	*Gm5861*
chr5	11340712	11340814	*Gm6460*	*Gm5861*
chr5	11343202	11343280	*Gm6460*	*Gm5861*
chr5	11415985	11416073	*Gm6460*	*Gm5861*
chr5	11417757	11417846	*Gm6460*	*Gm5861*
chr5	11500443	11500517	*Gm6460*	*Gm5861*
chr5	11502528	11502744	*Gm6460*	*Gm5861*
chr5	11502821	11502995	*Gm6460*	*Gm5861*
chr5	11503327	11503414	*Gm6460*	*Gm5861*
chr5	11503529	11503603	*Gm6460*	*Gm5861*
chr5	11504685	11504797	*Gm6460*	*Gm5861*
chr5	11596664	11596745	*4933402N22Rik*	*Gm6460*
chr5	11844632	11844735	*4933402N22Rik*	*Gm6460*
chr5	11846485	11846586	*4933402N22Rik*	*Gm6460*
chr5	11847638	11847710	*4933402N22Rik*	*Gm6460*
chr5	11907380	11907583	*4933402N22Rik*	*Gm6460*
chr5	11916692	11916800	*4933402N22Rik*	
chr5	11918482	11918695	*4933402N22Rik*	
chr5	14946098	14946176	*Speer4e*	*Gm10354*
chr5	14977955	14978029	*Gm10354*	
chr5	21495389	21496468	*Lrrc17*	*Fam185a*
chr5	22723924	22724137	*Orc5*	*5031425E22Rik*
chr5	23314000	23314919	*Orc5*	*5031425E22Rik*
chr5	23315360	23316750	*Orc5*	*5031425E22Rik*
chr5	23338027	23338108	*Orc5*	*5031425E22Rik*
chr5	23847725	23847810	*Tomm7*	
chr5	23859625	23861061	*Tomm7*	*Fam126a*
chr5	23908258	23911559	*Tomm7*	*Fam126a*
chr5	23911994	23913057	*Tomm7*	*Fam126a*
chr5	24051818	24051926	*Klhl7*	*Fam126a*
chr5	24052038	24052178	*Klhl7*	*Fam126a*

chr	start	stop	gene 1	gene 2
chr5	24204915	24205009	*Nupl2*	*Kcnh2*
chr5	27486049	27489355	*Speer4b*	*Dpp6*
chr5	27497802	27498018	*Speer4b*	*Dpp6*
chr5	27500139	27500321	*Speer4b*	*Dpp6*
chr5	30059760	30059894	*Tyms*	*Il6*
chr5	30060052	30060191	*Tyms*	*Il6*
chr5	34285779	34285912	*Mxd4*	*Zfyve28*
chr5	34628963	34630193	*Mfsd10*	*Add1*
chr5	37981238	37981328	*Msx1*	*Stx18*
chr5	38234925	38235766	*Tmem128*	*Lyar*
chr5	43946684	43947132	*Fgfbp1*	*Cd38*
chr5	45321013	45322393	*4930435H24Rik*	*Qdpr*
chr5	48369161	48369233	*Pacrgl*	
chr5	52237322	52237509	*Sod3*	*Dhx15*
chr5	52521349	52521559	*Ccdc149*	*Lgi2*
chr5	54428272	54428382	*Stim2*	
chr5	61575077	61577681	*G6pd2*	
chr5	64380600	64381313	*Klf3*	*Tbc1d1*
chr5	66713558	66714236	*Limch1*	*Uchl1*
chr5	67381593	67383891	*Bend4*	*Slc30a9*
chr5	70561957	70562029	*Gnpda2*	*Gabrg1*
chr5	73478209	73478451	*Dcun1d4*	
chr5	73780921	73783113	*Spata18*	*Usp46*
chr5	73801646	73802438	*Spata18*	*Usp46*
chr5	73864980	73865109	*Usp46*	*Spata18*
chr5	73912261	73914593	*Usp46*	*Spata18*
chr5	73914671	73915510	*Usp46*	*Spata18*
chr5	73944706	73945161	*Usp46*	*Spata18*
chr5	73945944	73946876	*Usp46*	*Spata18*
chr5	74028030	74030338	*Usp46*	*Spata18*
chr5	74030731	74031081	*Usp46*	*Spata18*
chr5	74142564	74143996	*Usp46*	*Rasl11b*
chr5	74144075	74145469	*Usp46*	*Rasl11b*
chr5	74145521	74146934	*Usp46*	*Rasl11b*
chr5	74845987	74846174	*Lnx1*	*Chic2*
chr5	75049718	75050790	*Gsx2*	*Chic2*
chr5	76572202	76572689	*Cep135*	*Exoc1*
chr5	76572806	76573013	*Cep135*	*Exoc1*
chr5	76573161	76573497	*Cep135*	*Exoc1*
chr5	76573894	76574928	*Cep135*	*Exoc1*
chr5	76575322	76575535	*Cep135*	*Exoc1*
chr5	76575629	76575856	*Cep135*	*Exoc1*
chr5	76575955	76576232	*Cep135*	*Exoc1*
chr5	76576355	76578522	*Cep135*	*Exoc1*
chr5	77021650	77021723	*1700023E05Rik*	*Hopx*
chr5	77311672	77311842	*Noa1*	*Polr2b*
chr5	77373415	77373964	*Igfbp7*	*Polr2b*
chr5	77408459	77408815	*Igfbp7*	
chr5	77666623	77667805	*Igfbp7*	
chr5	80589010	80589109	*Lphn3*	
chr5	92242756	92243741	*Ppef2*	*Uso1*
chr5	93748742	93748892	*Gm3106*	*C87414*
chr5	101667480	101667870	*Nkx6-1*	
chr5	108346962	108347808	*Gm10419*	*Pigg*
chr5	109907101	109907186	*4930522L14Rik*	*Gm15446*
chr5	110509037	110509188	*Fbrsl1*	*Galnt9*
chr5	110845537	110846209	*Ttc28*	*Chek2*
chr5	111552692	111553690	*C130026L21Rik*	*Mn1*
chr5	111979881	111980789	*Cryba4*	*C130026L21Rik*

Supplementary Table 3. H3K4me3 differential peaks identified in F1 - *(Continued)*

chr	start	stop	gene 1	gene 2
chr5	111981034	111982019	*Cryba4*	*C130026L21Rik*
chr5	112119127	112122659	*Cryba4*	*C130026L21Rik*
chr5	113155458	113156130	*Crybb3*	*2900026A02Rik*
chr5	113569741	113570054	*Wscd2*	*Cmklr1*
chr5	113570111	113571896	*Cmklr1*	*Wscd2*
chr5	114318000	114318288	*Myo1h*	*Kctd10*
chr5	115066679	115067684	*Acads*	*Sppl3*
chr5	116406121	116406941	*Ccdc60*	*Hspb8*
chr5	117781382	117782501	*Nos1*	*Ksr2*
chr5	118085125	118086380	*Gm9754*	*Fbxw8*
chr5	118503275	118504221	*Med13l*	*2410131K14Rik*
chr5	119594135	119594811	*Tbx3*	
chr5	119958671	119959496	*Rbm19*	*Tbx5*
chr5	119960238	119960765	*Rbm19*	*Tbx5*
chr5	120339988	120340132	*Lhx5*	*Rbm19*
chr5	121213668	121214141	*Gm15800*	*Rpl6*
chr5	121214329	121214583	*Gm15800*	*Rpl6*
chr5	121919319	121919608	*Fam109a*	*Cux2*
chr5	122030901	122031058	*Cux2*	*Fam109a*
chr5	126064498	126064581	*Gm4868*	
chr5	126092970	126093450	*Gm4868*	
chr5	128340751	128342291	*Tmem132d*	*Glt1d1*
chr5	129532396	129534029	*Mmp17*	*Sfswap*
chr5	130409246	130410171	*A330070K13Rik*	*Wbscr17*
chr5	134005888	134007441	*Gatsl2*	
chr5	134608318	134608412	*Lat2*	*Rfc2*
chr5	134905199	134905284	*Eln*	*Wbscr28*
chr5	136201453	136201542	*Prkrip1*	
chr5	140842017	140842105	*Gna12*	*Card11*
chr5	140842169	140842278	*Gna12*	*Card11*
chr5	142398381	142398994	*Foxk1*	
chr5	142857644	142858756	*Tnrc18*	*Fbxl18*
chr5	144132093	144132199	*Bhlha15*	*Lmtk2*
chr5	144767193	144767596	*Trrap*	
chr5	145082679	145083345	*Arpc1a*	
chr6	8045143	8045401	*Gm9825*	*Col28a1*
chr6	23532976	23534134	*Fezf1*	*Rnf133*
chr6	28817314	28818475	*Lrrc4*	*Snd1*
chr6	29485027	29485140	*Gm9047*	*Kcp*
chr6	29539586	29539678	*Irf5*	*Tnpo3*
chr6	37779318	37780337	*Trim24*	*Akr1d1*
chr6	38136786	38136982	*Atp6v0a4*	*D630045J12Rik*
chr6	39640647	39641014	*Ndufb2*	*Braf*
chr6	54606761	54607003	*2410066E13Rik*	*Plekha8*
chr6	56238926	56239032	*Pde1c*	*Ppp1r17*
chr6	58191216	58191288	*Vmn1r26*	*Vmn1r27*
chr6	58191784	58191869	*Vmn1r26*	*Vmn1r27*
chr6	69388107	69388298	*Gm11143*	*Igkv4-71*
chr6	70681729	70681806	*Rpia*	
chr6	76237723	76237930	*Gm9008*	
chr6	83539951	83540023	*Actg2*	
chr6	87112082	87112168	*D6Ertd527e*	*Antxr1*
chr6	88429792	88430835	*Eefsec*	*Dnajb8*
chr6	89416568	89417429	*Plxna1*	*Chchd6*
chr6	89876102	89876234	*Vmn1r44*	*Vmn1r43*
chr6	90881855	90882891	*Iqsec1*	*Nup210*
chr6	91640446	91641303	*Slc6a6*	*Lsm3*
chr6	92534436	92534846	*Trh*	*Prickle2*
chr6	92588703	92589341	*Trh*	*Prickle2*

chr	start	stop	gene 1	gene 2
chr6	93715023	93715163	*Adamts9*	*Magi1*
chr6	95351594	95351695	*Gm10234*	*Suclg2*
chr6	96876824	96876933	*1700123L14Rik*	*Fam19a4*
chr6	101399379	101399949	*Pdzrn3*	
chr6	110260859	110261938	*Grm7*	
chr6	111137885	111139409	*Grm7*	
chr6	111538828	111538971	*Lmcd1*	*Grm7*
chr6	112722557	112723499	*Rad18*	*Srgap3*
chr6	115848969	115849069	*Ift122*	*Mbd4*
chr6	119559006	119559847	*Wnt5b*	*Erc1*
chr6	119818592	119818999	*Wnt5b*	*Erc1*
chr6	120971320	120971846	*Bid*	*Mical3*
chr6	121059678	121063265	*Mical3*	*Mical3*
chr6	133988632	133988792	*Kap*	*Etv6*
chr6	133988844	133989224	*Kap*	*Etv6*
chr6	134363397	134365611	*Bcl2l14*	*Etv6*
chr6	135578144	135579498	*Emp1*	*Grin2b*
chr6	137837875	137838749	*Dera*	*Slc15a5*
chr6	139520516	139520737	*Pik3c2g*	*Rergl*
chr6	139630446	139631825	*Pik3c2g*	*Pik3c2g*
chr6	139631898	139632464	*Pik3c2g*	*Pik3c2g*
chr6	139633726	139633990	*Pik3c2g*	*Pik3c2g*
chr6	140242318	140242850	*Plekha5*	*Capza3*
chr6	140274507	140275408	*Plekha5*	*Capza3*
chr6	140289893	140290517	*Plekha5*	*Capza3*
chr6	140295061	140297217	*Plekha5*	*Capza3*
chr6	140590200	140590564	*Aebp2*	*Plekha5*
chr6	140590682	140591999	*Aebp2*	*Plekha5*
chr6	141074758	141076564	*Pde3a*	*Gm11077*
chr6	141141175	141141432	*Pde3a*	*Gm11077*
chr6	141728395	141729575	*Gm5724*	*Slco1b2*
chr6	142946218	142946537	*Gm766*	*St8sia1*
chr6	146666392	146667549	*Stk38l*	*Med21*
chr6	146685774	146686589	*Stk38l*	*Med21*
chr6	147690253	147690346	*Far2*	*Ccdc91*
chr6	147690417	147690536	*Far2*	*Ccdc91*
chr6	147823230	147824303	*Far2*	*Ccdc91*
chr6	149365011	149365123	*Bicd1*	*2810474O19Rik*
chr7	7103296	7103384	*Zfp954*	*Aurkc*
chr7	11733882	11734412	*Vmn1r72*	*Vmn1r73*
chr7	11734620	11735213	*Vmn1r72*	*Vmn1r73*
chr7	11971737	11972301	*Vmn1r77*	*Vmn1r76*
chr7	12030509	12030583	*Vmn1r76*	*Vmn1r77*
chr7	12132490	12132565	*Vmn1r78*	*Vmn1r77*
chr7	12950562	12950758	*Zfp324*	*2310014L17Rik*
chr7	14484804	14485296	*2810007J24Rik*	*Sult2a7*
chr7	16339050	16339524	*Bbc3*	*Sae1*
chr7	17423588	17423670	*Ceacam5*	*Psg29*
chr7	18680950	18681045	*Psg21*	*Psg20*
chr7	19935527	19935600	*Pvr*	*Igsf23*
chr7	24062623	24063602	*Zfp180*	*Vmn1r183*
chr7	25021976	25023072	*Atp1a3*	*Grik5*
chr7	26267386	26267482	*Vmn1r184*	
chr7	26267586	26268779	*Cyp2a4*	*Vmn1r184*
chr7	27922980	27923758	*1700049G17Rik*	*Zfp607*
chr7	30348998	30354064	*Sdhaf1*	*Lrfn3*
chr7	31162188	31162944	*Gramd1a*	*Scgb1b2*
chr7	31163386	31163489	*Gramd1a*	*Scgb1b2*
chr7	36395964	36396699	*Zfp507*	*Tshz3*

Supplementary Table 3. H3K4me3 differential peaks identified in F1 - *(Continued)*

chr	start	stop	gene 1	gene 2
chr7	39179674	39179761	*Gm5591*	*Gm5114*
chr7	39180998	39181073	*Gm5591*	*Gm5114*
chr7	39224209	39224310	*Gm5591*	*Gm5114*
chr7	41040145	41040522	*Gm2128*	*Gm4884*
chr7	41041561	41041645	*Gm2128*	*Gm4884*
chr7	41286131	41286369	*Gm5592*	*AI987944*
chr7	43927762	43929506	*Klk15*	*Klk4*
chr7	44707899	44707988	*Izumo2*	
chr7	45127000	45127085	*Rps11*	
chr7	45972952	45973063	*Ccdc114*	*Abcc6*
chr7	46286907	46288020	*Myod1*	*Otog*
chr7	49615970	49616065	*Dbx1*	*Nav2*
chr7	50789251	50790294	*Ano5*	*4933405O20Rik*
chr7	50930028	50930539	*Ano5*	*4933405O20Rik*
chr7	50931503	50931860	*Ano5*	*4933405O20Rik*
chr7	50932225	50932731	*Ano5*	*4933405O20Rik*
chr7	74057780	74059006	*St8sia2*	*Slco3a1*
chr7	75868167	75868275	*Agbl1*	*Klhl25*
chr7	84891126	84891208	*Olfr290*	*Olfr291*
chr7	88266025	88266517	*Ctsc*	*Grm5*
chr7	99092583	99093481	*Uvrag*	*Wnt11*
chr7	100011732	100012861	*Chrdl2*	*Pold3*
chr7	110509171	110509245	*Sbf2*	*Swap70*
chr7	112034752	112034831	*Usp47*	*Dkk3*
chr7	115075289	115076172	*Gm6816*	*Sox6*
chr7	117456191	117456282	*Xylt1*	*Rps15a*
chr7	119513257	119513830	*Umod*	*Pdilt*
chr7	119926296	119927434	*Lyrm1*	*Dnahc3*
chr7	125846222	125846795	*D430042O09Rik*	*Gsg1l*
chr7	126084942	126085844	*Gsg1l*	
chr7	126358996	126359790	*Lat*	*Sbk1*
chr7	126600843	126602287	*Il27*	*Nupr1*
chr7	128148658	128148753	*Itgad*	*Itgax*
chr7	128270247	128270336	*Slc5a2*	*BC017158*
chr7	129621155	129621844	*Wdr11*	*Fgfr2*
chr7	132997443	132998544	*Ctbp2*	*Zranb1*
chr7	138868417	138868496	*Ppp2r2d*	*Bnip3*
chr7	138869686	138869847	*Ppp2r2d*	*Bnip3*
chr7	138870390	138870506	*Ppp2r2d*	*Bnip3*
chr7	138872877	138873043	*Ppp2r2d*	*Bnip3*
chr7	138874128	138874279	*Ppp2r2d*	*Bnip3*
chr7	138876463	138876708	*Ppp2r2d*	*Bnip3*
chr7	138882243	138882748	*Bnip3*	*Ppp2r2d*
chr7	139017316	139017390	*Dpysl4*	*Jakmip3*
chr7	139987968	139988939	*6430531B16Rik*	*Adam8*
chr7	141178260	141178455	*Rnh1*	*Hras1*
chr7	142864767	142864851	*Gm6471*	*Th*
chr7	143568137	143568843	*Nap1l4*	*Cars*
chr7	143845663	143846801	*Gm498*	*Dhcr7*
chr7	144118511	144118717	*Shank2*	*Gm498*
chr7	144118811	144118929	*Shank2*	*Gm498*
chr7	144177275	144178288	*Shank2*	*Gm498*
chr7	144909510	144909965	*Oraov1*	*Fgf15*
chr8	15370476	15370688	*Myom2*	
chr8	17790269	17790391	*Mcph1*	*Csmd1*
chr8	24286466	24286557	*A730045E13Rik*	*1810011O10Rik*
chr8	29407823	29407899	*Unc5d*	
chr8	33852342	33853373	*Smim18*	*Rbpms*
chr8	33853436	33854129	*Smim18*	*Rbpms*

chr	start	stop	gene 1	gene 2
chr8	34119348	34120833	*Mboat4*	*Leprotl1*
chr8	36329712	36329821	*6430573F11Rik*	*Lonrf1*
chr8	36441080	36441172	*Lonrf1*	*6430573F11Rik*
chr8	40591325	40591493	*Mtmr7*	*Vps37a*
chr8	47001798	47003223	*Enpp6*	*Stox2*
chr8	47004536	47005941	*Enpp6*	*Stox2*
chr8	62480679	62481284	*Spock3*	*Anxa10*
chr8	70341294	70342067	*Upf1*	*Cers1*
chr8	72069021	72069265	*Olfr373*	*Olfr372*
chr8	72069453	72069718	*Olfr373*	*Olfr372*
chr8	72440994	72441081	*1700030K09Rik*	*Calr3*
chr8	72512065	72512420	*Slc35e1*	*Med26*
chr8	72512505	72512608	*Slc35e1*	*Med26*
chr8	74998835	74998945	*Tom1*	*Hmgxb4*
chr8	74999558	74999699	*Tom1*	*Hmgxb4*
chr8	74999996	75000102	*Tom1*	*Hmgxb4*
chr8	75000662	75001552	*Tom1*	*Hmgxb4*
chr8	75024024	75024142	*Tom1*	*Hmgxb4*
chr8	75029505	75029586	*Tom1*	
chr8	75712364	75712496	*1700007B14Rik*	
chr8	78211637	78211741	*Ttc29*	
chr8	80868122	80868216	*Smarca5*	*Gab1*
chr8	92742104	92743416	*Mmp2*	*Irx6*
chr8	93398883	93400354	*Ces1h*	*Ces5a*
chr8	93547691	93548118	*Gnao1*	*Ces5a*
chr8	95436366	95441043	*Gtl3*	
chr8	95455655	95456550	*Gtl3*	*Csnk2a2*
chr8	95680571	95681674	*Ndrg4*	*Gins3*
chr8	96512749	96513089	*Got2*	
chr8	97427438	97430489		
chr8	101666091	101666202		
chr8	105522481	105522578	*Hsd11b2*	*Atp6v0d1*
chr8	106969477	106969723	*Vps4a*	*Sntb2*
chr8	106969797	106970700	*Vps4a*	*Sntb2*
chr8	107064545	107064674	*Tmed6*	*Nip7*
chr8	107064920	107066171	*Tmed6*	
chr8	110127366	110129542	*Gm21964*	*Calb2*
chr8	110741557	110742943	*Mtss1l*	*Il34*
chr8	111340066	111340151	*Mlkl*	
chr8	117007726	117008963	*Gcsh*	*Pkd1l2*
chr8	118617621	118619077	*Hsbp1*	*Cdh13*
chr8	122312206	122312887	*Zfpm1*	*Trhr2*
chr8	124573750	124573886	*Agt*	*Capn9*
chr8	125176371	125177365	*Disc1*	*Sipa1l2*
chr8	125188880	125189408	*Disc1*	*Sipa1l2*
chr8	125583155	125586750	*Sipa1l2*	*Map10*
chr8	125960120	125960652	*Kcnk1*	*BC021891*
chr8	128372468	128373675	*Itgb1*	*Nrp1*
chr8	129264263	129264659	*2610044O15Rik8*	
chr8	129264758	129264867	*2610044O15Rik8*	
chr9	15363222	15363294	*Smco4*	*5830418K08Rik*
chr9	22353575	22354374	*Zfp810*	*Anln*
chr9	22354512	22354741	*Zfp810*	*Anln*
chr9	22667520	22667595	*Bmper*	*Bbs9*
chr9	22827534	22828778	*Bmper*	*Bbs9*
chr9	28020605	28021248	*Opcml*	
chr9	35013372	35013614	*St3gal4*	*Kirrel3*
chr9	36138572	36140039	*Gm7257*	*Gm5916*
chr9	38935536	38935621	*Olfr933*	*Olfr930*

Supplementary Table 3. H3K4me3 differential peaks identified in F1 - *(Continued)*

chr	start	stop	gene 1	gene 2
chr9	44622598	44623629	*Treh*	*Ddx6*
chr9	50146082	50146676	*Ncam1*	*Rpl10-ps3*
chr9	51273809	51274233	*Gm684*	*Pou2af1*
chr9	52920736	52920808	*AI593442*	*Ddx10*
chr9	56732880	56733694	*Lingo1*	*Odf3l1*
chr9	58911036	58912725	*Hcn4*	*Neo1*
chr9	60284349	60285944	*Nr2e3*	*Thsd4*
chr9	60286045	60287117	*Nr2e3*	*Thsd4*
chr9	61032196	61032401	*Gm9869*	*Gm10655*
chr9	62086290	62087114	*Glce*	*Spesp1*
chr9	62593084	62593170	*Itga11*	*Coro2b*
chr9	62642201	62642399	*Coro2b*	*Itga11*
chr9	62648513	62649538	*Coro2b*	*Itga11*
chr9	62649710	62650520	*Coro2b*	*Itga11*
chr9	62857087	62860267	*Calml4*	
chr9	63641219	63641388	*Aagab*	*Smad3*
chr9	63793816	63794624	*Smad3*	*Smad6*
chr9	64158660	64158795	*Zwilch*	*Lctl*
chr9	64472981	64474053	*Megf11*	*Rab11a*
chr9	64611395	64611801	*Rab11a*	*Megf11*
chr9	64687773	64688985	*Rab11a*	*Megf11*
chr9	65546602	65547008	*Ankdd1a*	*Plekho2*
chr9	66215432	66215969	*Herc1*	*Dapk2*
chr9	74712249	74712401	*Onecut1*	*Wdr72*
chr9	75789855	75789978	*Hmgcll1*	*Bmp5*
chr9	76933504	76933584	*Fam83b*	*Tinag*
chr9	80814241	80814619	*Impg1*	*Htr1b*
chr9	82550105	82550890	*Irak1bp1*	*Mei4*
chr9	89066216	89066293	*Trim43b*	*Bcl2a1a*
chr9	89066352	89066444	*Trim43b*	*Bcl2a1a*
chr9	89066588	89066730	*Trim43b*	*Bcl2a1a*
chr9	89066984	89067270	*Trim43b*	*Bcl2a1a*
chr9	89187144	89187387	*Bcl2a1b*	*4930579C12Rik*
chr9	99324949	99325045	*Esyt3*	*Cep70*
chr9	99489351	99489428	*1600029I14Rik*	*Armc8*
chr9	102554805	102555292	*Ky*	*Cep63*
chr9	104228259	104229039	*Ccrl1*	*Dnajc13*
chr9	105690471	105691043	*Pik3r4*	*Col6a6*
chr9	107693586	107693798	*Gnat1*	*Sema3f*
chr9	107693883	107694006	*Gnat1*	*Sema3f*
chr9	111173266	111174502	*Lrrfip2*	*Mlh1*
chr9	111291889	111297085	*Trank1*	*Epm2aip1*
chr9	111438943	111439774	*Dclk3*	
chr9	112691032	112691165	*Arpp21*	
chr9	113588488	113588614	*Pdcd6ip*	
chr9	117860956	117862436	*Rbms3*	*Zcwpw2*
chr9	118157572	118157778	*Eomes*	*Cmc1*
chr9	120021262	120023572	*Csrnp1*	*Xirp1*
chr9	120191140	120191212	*Myrip*	*Mobp*
chr9	121443529	121444764	*Cck*	*Trak1*
chrX	31716423	31716501	*Gm21637*	*Gm21492*
chrX	48274809	48275160	*Bcorl1*	*Utp14a*
chrX	48277192	48277608	*Bcorl1*	*Utp14a*
chrX	48282074	48282392	*Bcorl1*	*Utp14a*
chrX	49660859	49660946	*Olfr1320*	*Arhgap36*
chrX	52898616	52898723	*Phf6*	*Ccdc160*
chrX	52898835	52898922	*Phf6*	*Ccdc160*
chrX	74678676	74678763	*Gm6890*	*Gm5640*
chrX	96713325	96713556	*Gpr165*	

chr	start	stop	gene 1	gene 2
chrX	101792199	101792361	*8030474K03Rik*	
chrX	105579216	105579333	*Fgf16*	*Cypt2*
chrX	126018531	126018642	*4932411N23Rik*	
chrX	136958644	136958743	*Tmsb15b2*	*H2bfm*
chrY	5697119	5697208	*Gm20914*	*Gm21854*
chrY	6871131	6871204	*Gm21719*	*Gm20830*
chrY	29619054	29619130	*Gm20810*	
chrY	34062373	34062450	*Gm21184*	*Gm21745*

Supplementary Table 4. H3K4me3 differential peaks identified in F3

chr	start	end	gene 1	gene 2
chr1	19258172	19259650	*Tfap2b*	
chr1	23784996	23786721	*B3gat2*	*Smap1*
chr1	31730565	31730853	*Khdrbs2*	*Gm6489*
chr1	31734097	31734742	*Khdrbs2*	*Gm6489*
chr1	33637464	33638413	*Prim2*	
chr1	48333013	48333508	*C230029F24Rik*	
chr1	48737800	48738377	*C230029F24Rik*	
chr1	48740527	48742060	*C230029F24Rik*	
chr1	53190626	53190718	*1700019A02Rik*	
chr1	53249021	53249270	*1700019A02Rik*	*Pms1*
chr1	61170218	61170374	*Pard3b*	*Icos*
chr1	63091346	63091454	*Ino80d*	*Nrp2*
chr1	67307486	67307993	*Cps1*	
chr1	67441866	67442535	*Cps1*	
chr1	89859114	89859200	*D130058E05Rik*	*Agap1*
chr1	89862837	89862960	*D130058E05Rik*	*Agap1*
chr1	90632306	90632378	*Gm9991*	*Cops8*
chr1	101091993	101093753		
chr1	114211442	114212225		
chr1	114215169	114215477		
chr1	132744914	132745242	*Nfasc*	
chr1	135251859	135251948	*Elf3*	*Gm4204*
chr1	139179256	139179787	*Crb1*	*Dennd1b*
chr1	141140091	141141605	*Gm4845*	*Kcnt2*
chr1	146724807	146729233	*Fam5c*	*Gm9931*
chr1	146869053	146869282	*Fam5c*	*Gm9931*
chr1	148884572	148886692		
chr1	151221636	151225072	*Hmcn1*	*Ivns1abp*
chr1	151581277	151582302	*Edem3*	*Fam129a*
chr1	151582361	151583127	*Edem3*	*Fam129a*
chr1	151676507	151677520	*Edem3*	*Fam129a*
chr1	151997581	152002069	*1700025G04Rik*	*Edem3*
chr1	153012025	153013614	*Nmnat2*	*Lamc2*
chr1	153031617	153032390	*Nmnat2*	*Lamc2*
chr1	153396418	153397320	*Lamc1*	*E330020D12Rik*
chr1	165188397	165189701	*Tbx19*	*Sft2d2*
chr1	165669424	165670524	*Mpzl1*	*Rcsd1*
chr1	166189211	166191233	*Mael*	*Gpa33*
chr1	166240968	166241137	*Mael*	
chr1	167182968	167183156	*Uck2*	*Fam78b*
chr1	171049137	171049894	*Fcgr3*	*Fcgr4*
chr1	171546704	171546783	*Cd244*	*Itln1*
chr1	179644344	179644658	*Sccpdh*	*Cnst*
chr1	185086944	185087085	*Rab3gap2*	*Mark1*
chr1	190171777	190171866	*Prox1*	
chr1	192717266	192717340	*Hhat*	*Kcnh1*
chr10	18302491	18303619	*Nhsl1*	*Ccdc28a*
chr10	22058022	22059681	*4930444G20Rik*	*Sgk1*
chr10	23726547	23726645	*Eya4*	*Rps12*
chr10	41715134	41717123	*Ccdc162*	*Cep57l1*
chr10	46881602	46882270		
chr10	50814538	50814616	*Sim1*	*Ascc3*
chr10	54887371	54887699	*Man1a*	
chr10	69574415	69574604	*Ccdc6*	*Ank3*
chr10	76341395	76342540	*Dip2a*	*S100b*
chr10	85097932	85098078	*AI597468*	*Fhl4*
chr10	95317213	95318626	*Plxnc1*	*Cradd*
chr10	99677345	99678720	*Csl*	*Gad1-ps*
chr10	99747992	99748495	*Csl*	*Gad1-ps*
chr10	99754955	99755367	*Csl*	*Gad1-ps*
chr10	99928409	99930599	*Csl*	*Kitl*
chr10	100179952	100180940	*Gm9476*	*Kitl*
chr10	100595957	100596811	*1700017N19Rik*	
chr10	101894089	101894242	*Mgat4c*	*Nts*
chr10	103566974	103567889	*Gm6763*	*Slc6a15*
chr10	105314917	105315854	*Tmtc2*	
chr10	116902701	116903657	*Myrfl*	*Rab3ip*
chr10	119295766	119295977	*Grip1*	*Cand1*
chr10	120074788	120074922	*Helb*	*Grip1*
chr10	122815553	122815654	*Ppm1h*	*Mon2*
chr10	125957197	125958510	*Slc16a7*	*Lrig3*
chr10	130174698	130175780	*Olfr825*	*Olfr826*
chr11	8270618	8271029	*Tns3*	
chr11	8273771	8274215	*Tns3*	
chr11	18636288	18636577	*Etaa1*	*Gm16140*
chr11	27135093	27135282	*Vrk2*	
chr11	30784838	30786047	*Gpr75*	*Psme4*
chr11	36170610	36172483	*Wwc1*	
chr11	52577870	52582487	*Fstl4*	*9530068E07Rik*
chr11	61026202	61028653	*Kcnj12*	*Map2k3*
chr11	61714897	61715184	*Slc5a10*	*Fam83g*
chr11	64017259	64017399	*Hs3st3b1*	*Cox10*
chr11	64780645	64782566	*Elac2*	*Hs3st3a1*
chr11	65712785	65713566	*Myocd*	*Map2k4*
chr11	67002140	67002267	*Tmem220*	*Pirt*
chr11	68790129	68790227	*Ndel1*	*Myh10*
chr11	71968510	71970580	*Aipl1*	*Wscd1*
chr11	73167687	73167803	*P2rx5*	*Emc6*
chr11	73693349	73695139	*Zfp735*	*Olfr389*
chr11	77497894	77499269	*Git1*	*Trp53i13*
chr11	80766304	80767093	*Myo1d*	*Cdk5r1*
chr11	82888599	82889054	*Fndc8*	*Rad51d*
chr11	84911258	84911604	*Pigw*	*Znhit3*
chr11	86630510	86631028	*Vmp1*	*Tubd1*
chr11	88290413	88290671	*1700106J16Rik*	
chr11	92075095	92075264	*Kif2b*	
chr11	95532485	95532596	*Nxph3*	*Ngfr*
chr11	105910714	105910902	*Cyb561*	*Tanc2*
chr11	109217693	109221007	*E030025P04Rik*	*Rgs9*
chr11	111079171	111080576	*Kcnj2*	
chr11	111080795	111081718	*Kcnj2*	
chr11	118135581	118137738	*Dnahc17*	*Cyth1*
chr11	119435214	119435346	*Endov*	*Rnf213*
chr11	120908842	120909094	*Fasn*	*Ccdc57*
chr12	3113975	3114537	*Rab10*	
chr12	5613822	5614243	*2810032G03Rik*	
chr12	5616722	5618099	*2810032G03Rik*	
chr12	12658802	12659058	*Rpl36-ps3*	*Fam49a*
chr12	13168903	13169949	*Mycn*	*Ddx1*
chr12	18283912	18284030	*5730507C01Rik*	*B430203G13Rik*
chr12	18286146	18286410	*5730507C01Rik*	*B430203G13Rik*
chr12	18320868	18321053	*5730507C01Rik*	*B430203G13Rik*
chr12	18363629	18363984	*5730507C01Rik*	*B430203G13Rik*
chr12	18514054	18515585	*5730507C01Rik*	
chr12	18609610	18611122	*Gm3944*	*5730507C01Rik*
chr12	19243246	19243423	*Gm5784*	*Gm3944*
chr12	19249067	19249280	*Gm5784*	*Gm3944*
chr12	27577666	27577821	*Sox11*	*Dcdc2c*

Supplementary Table 4. H3K4me3 differential peaks identified in F3 - *(Continued)*

chr	start	end	gene 1	gene 2
chr12	54995721	54995860	*Baz1a*	*2700097O09Rik*
chr12	56103197	56103760	*Mbip*	*Brms1l*
chr12	70492610	70493509	*Frmd6*	*Tmx1*
chr12	76887759	76887864	*Fntb*	*Max*
chr12	76920126	76921380	*Max*	*Fntb*
chr12	77630160	77630428	*Gphn*	*Fut8*
chr12	84960542	84961631	*Ltbp2*	*Fcf1*
chr12	85340075	85340168	*Nek9*	
chr12	85809536	85809652	*0610007P14Rik*	*Mfsd7c*
chr12	89579044	89579356	*Nrxn3*	
chr12	91338578	91339179	*Dio2*	*Cep128*
chr12	93538662	93538756	*Gm9726*	
chr12	95563306	95563380	*Flrt2*	
chr12	96400511	96400949	*1700019M22Rik*	
chr12	106714760	106718300	*3110018I06Rik*	*Vrk1*
chr12	108075447	108076841	*Bcl11b*	*Setd3*
chr12	108164332	108164692	*Bcl11b*	*Setd3*
chr12	110568237	110568909	*Dync1h1*	*Ppp2r5c*
chr12	113150565	113150820	*Crip1*	
chr13	12291169	12293696	*Mtr*	*Actn2*
chr13	12293763	12294497	*Mtr*	*Actn2*
chr13	15788101	15788601	*Inhba*	*Gli3*
chr13	17857862	17861641	*Cdk13*	*Rala*
chr13	47006505	47006638	*Kif13a*	*Nhlrc1*
chr13	47007391	47007643	*Kif13a*	*Nhlrc1*
chr13	56492771	56493369	*Fbxl21*	*Il9*
chr13	56664280	56664411	*Smad5*	*Tgfbi*
chr13	59855698	59855879	*Zcchc6*	*Gas1*
chr13	59873640	59874473	*Zcchc6*	*Gas1*
chr13	64760876	64762089	*1190003K10Rik*	*Cntnap3*
chr13	77233924	77234136	*Fam172a*	*2210408I21Rik*
chr13	87396476	87396923		
chr13	87668496	87669430		
chr13	93197833	93200412	*Homer1*	*Papd4*
chr13	93202271	93202717	*Homer1*	*Papd4*
chr13	104607987	104609032	*Cwc27*	*Adamts6*
chr13	114993706	114994450	*Itga2*	*Pelo*
chr13	116738327	116739268	*Isl1*	*Parp8*
chr13	116927655	116928323	*Isl1*	*Parp8*
chr13	119597680	119598116	*Gm21967*	*Gm7120*
chr13	119674005	119675320	*Hmgcs1*	*Ccl28*
chr13	119675859	119676152	*Hmgcs1*	*Ccl28*
chr13	119860511	119863512	*Zfp131*	*Tcstv1*
chr14	5899234	5899314	*Gm3424*	*Gm3453*
chr14	11235739	11236281	*Rpl21-ps4*	*ENSMUSG00000097148*
chr14	11583389	11585159	*3830406C13Rik*	*Ptprg*
chr14	12911452	12911833	*Cadps*	*Gm5087*
chr14	14194758	14198833	*Olfr31*	*Olfr720*
chr14	15110130	15111040	*Nek10*	*Lrrc3b*
chr14	15111491	15111585	*Nek10*	*Lrrc3b*
chr14	16068114	16068870	*Lrrc3b*	*Ngly1*
chr14	16803157	16804176	*Rarb*	
chr14	17025589	17025933	*Thrb*	*Rarb*
chr14	18895334	18895599	*Ube2e2*	
chr14	18895928	18896770	*Ube2e2*	
chr14	19519292	19519923	*Gm9637*	*Gm2244*
chr14	42426069	42426141	*Gm8005*	*Gm7995*
chr14	42826555	42826685	*Gm10378*	*Gm8094*
chr14	43043326	43043426	*Gm8094*	*Gm10378*
chr14	44500044	44501513	*BC061237*	
chr14	44518245	44519509	*Gm8247*	*BC061237*
chr14	44561874	44562096	*Gm8247*	*BC061237*
chr14	44669223	44669656	*Gm8267*	*Gm8247*
chr14	46795161	46795424	*Cnih*	*Gmfb*
chr14	46802511	46803860	*Cnih*	*Gmfb*
chr14	47300653	47300898	*Mapk1ip1l*	*Socs4*
chr14	49238231	49239714	*1700011H14Rik*	*Naa30*
chr14	49239904	49240971	*1700011H14Rik*	*Naa30*
chr14	51007629	51008901	*Rnase10*	
chr14	51344022	51346048	*Gm7247*	*Gm21718*
chr14	51381823	51383470	*Vmn2r88*	*Gm7247*
chr14	51571955	51572067	*Gm5622*	*Gm4181*
chr14	51592314	51592575	*Gm5622*	*Gm4181*
chr14	51611321	51611473	*Gm4181*	*Gm5622*
chr14	51612192	51612320	*Gm4181*	*Gm5622*
chr14	51633405	51633738	*Gm4181*	*Gm5622*
chr14	51633840	51634062	*Gm4181*	*Gm5622*
chr14	51654741	51655063	*Gm4181*	*Gm5800*
chr14	51677420	51678184	*Gm4181*	*Gm5800*
chr14	54296318	54296945	*Oxa1l*	*Olfr49*
chr14	60342634	60345166	*Amer2*	*Mtmr6*
chr14	61090467	61091931	*Sacs*	*Tnfrsf19*
chr14	63720546	63721970	*Pinx1*	*Xkr6*
chr14	65543747	65543848	*Pnoc*	*Elp3*
chr14	72965313	72965866	*Fndc3a*	*Cysltr2*
chr14	73238202	73238390	*Lpar6*	
chr14	83143921	83144938		
chr14	90277907	90278174	*Gm10110*	
chr14	90278351	90278623	*Gm10110*	
chr14	90278772	90279434	*Gm10110*	
chr14	90659136	90659985	*Gm10110*	
chr14	93366967	93367537	*Pcdh9*	
chr14	93930740	93931270	*Pcdh9*	
chr14	93933673	93934520	*Pcdh9*	
chr14	94734207	94735051	*Pcdh9*	
chr14	94735164	94736752	*Pcdh9*	
chr14	95556815	95558223	*4921530L21Rik*	
chr14	98781668	98782151	*Dach1*	*Mzt1*
chr14	105217977	105218581	*Rbm26*	*Ndfip2*
chr14	112299873	112299946	*Slitrk5*	
chr14	117111888	117112569	*Gpc6*	*Dct*
chr14	117113172	117113892	*Gpc6*	*Dct*
chr14	117116137	117116223	*Gpc6*	*Dct*
chr14	117664459	117664558	*Dct*	*Gpc6*
chr14	123347506	123348337	*Tmtc4*	*Nalcn*
chr14	123844592	123844747	*Itgbl1*	*Fgf14*
chr15	12104577	12105431	*Sub1*	*Zfr*
chr15	12109262	12109380	*Sub1*	*Zfr*
chr15	14484690	14484778		
chr15	15784810	15785205	*Cdh9*	
chr15	15787173	15789336	*Cdh9*	
chr15	25407570	25407645	*9230109A22Rik*	*Basp1*
chr15	27854195	27857658	*Fam105a*	*Trio*
chr15	30030181	30030408	*Ctnnd2*	
chr15	30030570	30035316	*Ctnnd2*	
chr15	46519988	46521104	*4930548G14Rik*	
chr15	57475995	57479482	*Slc22a22*	
chr15	58001248	58002815	*Fam83a*	*9130401M01Rik*

Supplementary Table 4. H3K4me3 differential peaks identified in F3 - *(Continued)*

chr	start	end	gene 1	gene 2
chr15	58011860	58012192	*9130401M01Rik*	*Fam83a*
chr15	58012534	58012606	*9130401M01Rik*	*Fam83a*
chr15	58300789	58302081	*Fbxo32*	*Klhl38*
chr15	60794567	60795986	*4933412E24Rik*	*Fam84b*
chr15	73623688	73624666	*Slc45a4*	
chr15	76270050	76270149	*Smpd5*	*Spatc1*
chr15	77708418	77709375	*Apol9b*	*Apol7e*
chr15	77709682	77709811	*Apol9b*	*Apol7e*
chr15	83793034	83793193	*Mpped1*	*Efcab6*
chr15	88779513	88780738	*Zbed4*	*Alg12*
chr15	88780809	88783182	*Zbed4*	*Alg12*
chr15	97625462	97626789	*Amigo2*	*Rpap3*
chr16	8769240	8769389	*1810013L24Rik*	*Usp7*
chr16	10191845	10191964	*Atf7ip2*	
chr16	16111694	16111958	*Mzt2*	*2310008H04Rik*
chr16	19647984	19648240	*Olfr171*	*Lamp3*
chr16	19652017	19652426	*Olfr171*	*Lamp3*
chr16	32282577	32282785	*Ubxn7*	*Rnf168*
chr16	45172507	45173373	*Btla*	*Atg3*
chr16	45173426	45174784	*Btla*	*Atg3*
chr16	45592559	45594032	*Gcsam*	*Slc9c1*
chr16	57113662	57115239	*2310005G13Rik*	*Tomm70a*
chr16	58932237	58933130	*Olfr181*	
chr16	62957513	62957949	*Pros1*	*Epha3*
chr16	63289287	63290730	*Pros1*	*Epha3*
chr16	63291285	63292567	*Pros1*	*Epha3*
chr16	64599877	64601693	*Csnka2ip*	*4930453N24Rik*
chr16	68626118	68626272		
chr16	68928345	68928791	*Speer2*	
chr16	69866935	69869269	*Speer2*	
chr16	78056689	78057221	*Gm11146*	*Cxadr*
chr16	78088795	78090286	*Gm11146*	*Cxadr*
chr16	78281729	78282778	*Gm11146*	*Cxadr*
chr16	78454540	78457270	*D16Ertd472e*	*Cxadr*
chr16	82210495	82212586		
chr16	91117420	91117510	*4932438H23Rik*	*Gm9881*
chr17	25079593	25081127	*Tmem204*	
chr17	33541472	33542477	*Myo1f*	*Adamts10*
chr17	33873670	33874431	*Kifc1*	*Cd320*
chr17	33874501	33875283	*Kifc1*	*Cd320*
chr17	33905122	33906145	*Daxx*	
chr17	35310722	35310799	*H2-Q1*	*H2-D1*
chr17	36041985	36042692	*Gm6034*	*H2-T22*
chr17	36167582	36167703	*Gm8909*	
chr17	36189318	36189551	*H2-T3*	
chr17	55558213	55558285	*Vmn2r118*	*St6gal2*
chr17	56901969	56902075	*1700061G19Rik*	*Mllt1*
chr17	57673431	57673550	*Vmn2r120*	*Cntnap5c*
chr17	58693460	58694004	*2610034M16Rik*	*Cntnap5c*
chr17	71717597	71717679	*BC027072*	*Fam179a*
chr17	74949916	74951292	*Ltbp1*	*Ttc27*
chr17	74951518	74952318	*Ltbp1*	*Ttc27*
chr17	83448390	83449040	*Cox7a2l*	*Eml4*
chr17	84837858	84841829	*Ppm1b*	*Lrpprc*
chr17	86495874	86496983	*Gm10309*	*Prkce*
chr17	87942288	87945587	*Msh6*	*Rpl36-ps4*
chr17	88338602	88341424	*Fbxo11*	*Foxn2*
chr17	89501333	89501694	*Fshr*	*Gm10184*
chr18	6989654	6989731	*Mkx*	*Rab18*
chr18	7318743	7319393	*Armc4*	*Mpp7*
chr18	7319445	7319553	*Armc4*	*Mpp7*
chr18	12316939	12318856	*Lama3*	*Ankrd29*
chr18	16463291	16465963	*Cdh2*	*Gm10036*
chr18	16850154	16852282	*Cdh2*	
chr18	17326233	17327919	*Cdh2*	
chr18	17454036	17454251	*Cdh2*	
chr18	18483607	18484229		
chr18	18567379	18568631		
chr18	24022936	24023162	*Zfp191*	
chr18	24763456	24763708	*Fhod3*	*Tpgs2*
chr18	30011514	30012122	*Pik3c3*	
chr18	30014094	30016142	*Pik3c3*	
chr18	58028275	58028352	*Slc12a2*	*Fbn2*
chr18	68471476	68475249	*Mc2r*	*4930546C10Rik*
chr18	74686521	74689649	*Acaa2*	*Myo5b*
chr18	75046471	75047672	*Smad7*	*Dym*
chr18	82518080	82518305	*Mbp*	*Zfp236*
chr18	84123978	84124614	*Zadh2*	*Zfp407*
chr18	85001972	85002297	*Timm21*	
chr19	6126939	6127273	*Sac3d1*	*Snx15*
chr19	15158159	15158655	*Tle4*	*Psat1*
chr19	18918943	18921991	*Rorb*	*Trpm6*
chr19	21551752	21553031	*Gm3443*	
chr19	21804380	21804838	*Trpm3*	*Tmem2*
chr19	31126491	31127533	*Cstf2t*	*Prkg1*
chr19	43267086	43268986	*Hps1*	*Hpse2*
chr19	44056920	44057698	*Cyp2c44*	*Erlin1*
chr19	44512847	44512946	*Wnt8b*	*Sec31b*
chr19	58878592	58878673	*Eno4*	*Hspa12a*
chr2	18505029	18505430	*Commd3*	*Dnajc1*
chr2	18505833	18506069	*Commd3*	*Dnajc1*
chr2	20905434	20905639	*Arhgap21*	*Etl4*
chr2	21836323	21837112	*Myo3a*	*Gpr158*
chr2	29593653	29593908	*Rapgef1*	*Med27*
chr2	30325279	30325351	*Sh3glb2*	*Nup188*
chr2	34608914	34609175	*Gapvd1*	*Mapkap1*
chr2	35518811	35519467	*Dab2ip*	*Ggta1*
chr2	38647052	38647887	*Psmb7*	
chr2	40127781	40127905	*Ppp6c*	
chr2	40934990	40936383		
chr2	56067555	56069229	*Kcnj3*	
chr2	57068870	57069850	*Nr4a2*	
chr2	68349486	68350561	*Stk39*	*B3galt1*
chr2	68674560	68675161	*Cers6*	*4932414N04Rik*
chr2	71820038	71820196	*Pdk1*	*Itga6*
chr2	71825582	71825661	*Pdk1*	*Itga6*
chr2	71838329	71838434	*Pdk1*	*Itga6*
chr2	71838579	71838679	*Pdk1*	*Itga6*
chr2	72184828	72185821	*B230120H23Rik*	*Rapgef4*
chr2	78048690	78053570	*Ube2e3*	*Cwc22*
chr2	88578453	88578561	*Olfr1189*	*Olfr1188*
chr2	88578941	88579131	*Olfr1189*	*Olfr1188*
chr2	88615725	88617994	*Olfr1193*	*Olfr1189*
chr2	91712211	91712348	*Atg13*	*Harbi1*
chr2	100053469	100054309		
chr2	104699463	104700358	*Tcp11l1*	*Cstf3*
chr2	108420569	108421859	*Mettl15*	
chr2	118997802	118997898	*Ccdc32*	*Chst14*

Supplementary Table 4. H3K4me3 differential peaks identified in F3 - *(Continued)*

chr	start	end	gene 1	gene 2
chr2	119169704	119169874	*Gm14137*	
chr2	125522003	125522229	*Fbn1*	*Cep152*
chr2	125530309	125531388	*Fbn1*	*Cep152*
chr2	127021816	127023514	*Blvra*	*Ap4e1*
chr2	127182539	127183523	*Itpripl1*	*1810024B03Rik*
chr2	128458378	128458450	*Gm355*	*Bcl2l11*
chr2	132945899	132946029	*Fermt1*	
chr2	137663246	137664518	*Btbd3*	*Jag1*
chr2	145640371	145643771	*Rin2*	*Slc24a3*
chr2	145766335	145767131	*Rin2*	*Slc24a3*
chr2	145767384	145768325	*Rin2*	*Slc24a3*
chr2	146281648	146283254	*Insm1*	*Ralgapa2*
chr2	146490211	146490653	*Ralgapa2*	*Insm1*
chr2	148341483	148343294	*Foxa2*	*Sstr4*
chr2	148375367	148376209	*Foxa2*	*Sstr4*
chr2	149821764	149821857	*Syndig1*	*Cst10*
chr2	155282883	155283411	*Map1lc3a*	*Pigu*
chr2	155376178	155377171	*Pigu*	*Trp53inp2*
chr2	155402042	155403697	*Trp53inp2*	*Ncoa6*
chr2	155782340	155785361	*Mmp24*	*Eif6*
chr2	155969683	155970554	*Cep250*	*6430550D23Rik*
chr2	156003073	156004413	*Ergic3*	*6430550D23Rik*
chr2	156142015	156142415	*Romo1*	
chr2	156361778	156363318	*Epb4.1l1*	*Scand1*
chr2	156682444	156684390	*Myl9*	*Dlgap4*
chr2	156780034	156781622	*Tgif2*	*Myl9*
chr2	156783822	156784583	*Tgif2*	*Myl9*
chr2	156809770	156810548	*Tgif2*	*Myl9*
chr2	156948350	156948814	*Sla2*	*Ndrg3*
chr2	157652298	157652583	*Blcap*	*Ctnnbl1*
chr2	157655134	157658133	*Blcap*	*Ctnnbl1*
chr2	158698495	158699301	*Fam83d*	*Ppp1r16b*
chr2	163973175	163973880	*Rims4*	*Ywhab*
chr2	164963570	164963642	*Slc12a5*	
chr2	166256610	166256763	*Sulf2*	*Prex1*
chr2	167204062	167204209	*Kcnb1*	*Ptgis*
chr2	167318205	167319415	*Ptgis*	*B4galt5*
chr2	169234079	169234375	*1700101G07Rik*	*Zfp64*
chr2	177455359	177455856	*Gm14418*	*Gm14420*
chr2	178502616	178502741	*Cdh4*	*Cdh26*
chr3	6798644	6800664	*Pkia*	*1700008P02Rik*
chr3	10453856	10454011	*Snx16*	
chr3	10454066	10454252	*Snx16*	
chr3	20374205	20374685	*Agtr1b*	
chr3	20381054	20381237	*Agtr1b*	
chr3	20381431	20381687	*Agtr1b*	
chr3	20382288	20382360	*Agtr1b*	
chr3	20383052	20383327	*Agtr1b*	
chr3	20384198	20385728	*Agtr1b*	
chr3	28891636	28892123	*Gm1527*	
chr3	30437753	30438397	*Gm10258*	*Actrt3*
chr3	31636258	31636501	*Slc7a14*	*Kcnmb2*
chr3	31909220	31909325	*Kcnmb2*	*Zmat3*
chr3	42182818	42183769	*D3Ertd751e*	
chr3	43091029	43091194		
chr3	51101071	51102354	*Slc7a11*	*Ccrn4l*
chr3	51104722	51105178	*Slc7a11*	*Ccrn4l*
chr3	64051478	64051709	*Vmn2r1*	*Gmps*
chr3	70571607	70573173	*Otol1*	

chr	start	end	gene 1	gene 2
chr3	73300899	73301698	*Slitrk3*	*Bche*
chr3	74324218	74324817	*Bche*	*Zbbx*
chr3	78307280	78307931	*Gm5277*	
chr3	80512142	80512220	*Gria2*	*Fam198b*
chr3	83307184	83308211	*Sfrp2*	*Gm10710*
chr3	92570843	92572713	*Sprr4*	*Ivl*
chr3	96273528	96274193	*Hist2h2bb*	*Fcgr1*
chr3	96489780	96489880	*BC107364*	*Hfe2*
chr3	96983949	96985415	*Gja8*	*Gja5*
chr3	97351378	97354167	*Bcl9*	*Olfr1402*
chr3	97751226	97752296	*Pde4dip*	*Prkab2*
chr3	99553834	99555692	*Spag17*	*Tbx15*
chr3	120730052	120730189	*Ptbp2*	*Rwdd3*
chr3	120753979	120754701	*Ptbp2*	*Rwdd3*
chr3	121100065	121100357	*Rwdd3*	
chr3	135585108	135585703	*Manba*	*Nfkb1*
chr3	159800609	159800733	*Wls*	*Rpe65*
chr4	5460820	5461245	*Impad1*	*Fam110b*
chr4	5463660	5466209	*Impad1*	*Fam110b*
chr4	10845857	10847285	*2610301B20Rik*	
chr4	10863935	10865276	*2610301B20Rik*	
chr4	10871458	10871864	*2610301B20Rik*	
chr4	11180310	11183218	*Ccne2*	*Trp53inp1*
chr4	15054570	15054742	*Necab1*	*Tmem55a*
chr4	27270809	27271357	*Manea*	
chr4	34960529	34963823	*Ifnk*	*Cga*
chr4	41698620	41699244	*Cntfr*	
chr4	41775035	41775187	*Ccl27a*	
chr4	41775565	41776212	*Ccl27a*	
chr4	41866557	41870310	*Ccl27a*	*Gm21541*
chr4	42035352	42035902	*Gm21093*	*Gm10597*
chr4	42053107	42057892	*Gm13304*	*Gm10597*
chr4	42082367	42086089	*Gm13304*	*Gm10597*
chr4	42150641	42155714	*Gm13306*	
chr4	42169373	42174487	*Gm13305*	
chr4	42184162	42185406	*Ccl21b*	*Gm13305*
chr4	42215720	42219542	*Gm13305*	*Ccl21b*
chr4	42458341	42459098	*Gm3883*	*Gm10597*
chr4	42550742	42555264	*Gm10591*	*Gm13298*
chr4	42579870	42580129	*Gm13298*	*Gm10591*
chr4	42618910	42620510	*Gm2564*	*Gm10591*
chr4	42647723	42652682	*Ccl27b*	*Gm2564*
chr4	42666543	42671264	*4930578G10Rik*	*Il11ra2*
chr4	42715217	42718307	*Il11ra2*	*4930578G10Rik*
chr4	42794526	42795256	*Ccl21a*	*Gm12394*
chr4	42852446	42853145	*Ccl21a*	*Gm12394*
chr4	55810833	55811000	*Klf4*	*Actl7b*
chr4	58174331	58177649	*Txndc8*	*Svep1*
chr4	58325232	58326350	*Musk*	*Lpar1*
chr4	58639211	58639554	*Lpar1*	*Olfr267*
chr4	58639651	58640541	*Lpar1*	*Olfr267*
chr4	60038139	60038230	*Mup7*	*Mup6*
chr4	67361892	67362511	*Tlr4*	
chr4	115719168	115719920	*Cyp4b1*	*Efcab14*
chr4	117731956	117732133	*Dmap1*	*Klf17*
chr4	119929760	119930546	*Edn2*	*Hivep3*
chr4	120848669	120849534	*Nfyc*	*Rims3*
chr4	130839175	130840242	*Laptm5*	*Sdc3*
chr4	131070173	131070359	*Matn1*	*Ptpru*

Supplementary Table 4. H3K4me3 differential peaks identified in F3 - *(Continued)*

chr	start	end	gene 1	gene 2
chr4	132263711	132263802	*Gmeb1*	*Taf12*
chr4	132264465	132265717	*Gmeb1*	*Taf12*
chr4	134159739	134160320	*Sh3bgrl3*	*Cep85*
chr4	134928578	134928765	*Syf2*	
chr4	138204526	138204603	*Hp1bp3*	*Eif4g3*
chr4	146503989	146504256	*Gm13248*	*Gm13247*
chr4	147219346	147219660	*Gm13151*	*Gm13139*
chr4	148376297	148376419	*Ptchd2*	*Ubiad1*
chr4	151158080	151158201	*Gm13090*	*Camta1*
chr4_GL456350_random	29836	30472		
chr4_GL456350_random	33136	33215		
chr4_GL456350_random	33281	33410		
chr4_GL456350_random	58086	58664		
chr4_GL456350_random	58724	58997		
chr4_GL456350_random	59079	59355		
chr4_GL456350_random	80036	80366		
chr4_JH584293_random	1341	6220		
chr4_JH584293_random	20320	25054		
chr4_JH584293_random	90312	93296		
chr4_JH584293_random	117710	119111		
chr4_JH584293_random	119163	122515		
chr4_JH584293_random	140366	140442		
chr4_JH584294_random	14367	19146		
chr4_JH584294_random	29131	30387		
chr4_JH584294_random	63327	63527		
chr5	3236405	3236840	*Cdk6*	
chr5	3572952	3573499	*Pex1*	*1700109H08Rik*
chr5	3731226	3731761	*Ankib1*	*4930511M11Rik*
chr5	3732341	3733290	*Ankib1*	*4930511M11Rik*
chr5	3997150	3998219	*Akap9*	*Cyp51*
chr5	5198083	5198361	*Fzd1*	*Cdk14*
chr5	16169357	16169485	*Hgf*	*Cacna2d1*
chr5	32088659	32093601	*Gm10463*	*Bre*
chr5	32101541	32104401	*Gm10463*	*Bre*
chr5	32952812	32954867	*Ywhah*	*Depdc5*
chr5	36768941	36769055	*Bloc1s4*	*Mrfap1*
chr5	37214844	37214930	*Crmp1*	*Gm1043*
chr5	51933482	51933564	*Ppargc1a*	*Dhx15*
chr5	53367337	53367419	*Rbpj*	*Smim20*
chr5	59096470	59097155		
chr5	61576353	61577627	*G6pd2*	
chr5	62977030	62978230	*3110047P20Rik*	*Gm17384*
chr5	62980941	62981238	*3110047P20Rik*	*Gm17384*
chr5	82458557	82459614	*Tecrl*	
chr5	82819625	82820129	*Tecrl*	
chr5	89511974	89512329	*Gc*	*Npffr2*
chr5	90212390	90214552	*Adamts3*	*Cox18*
chr5	101663345	101668051	*Nkx6-1*	
chr5	106520111	106520949	*Barhl2*	*Zfp644*
chr5	107043922	107043994	*Cdc7*	*Tgfbr3*
chr5	107044612	107044684	*Cdc7*	*Tgfbr3*
chr5	109906670	109907483	*4930522L14Rik*	*Gm15446*
chr5	110508925	110509389	*Fbrsl1*	*Galnt9*
chr5	111892907	111894519	*C130026L21Rik*	*Cryba4*
chr5	111979914	111980834	*Cryba4*	*C130026L21Rik*
chr5	112119182	112122665	*Cryba4*	*C130026L21Rik*
chr5	112625359	112626433	*Sez6l*	*Myo18b*
chr5	112640207	112642001	*Sez6l*	*Myo18b*
chr5	113844145	113844318	*Selplg*	*Coro1c*
chr5	114566168	114566313	*Fam222a*	
chr5	115308596	115308749	*Srsf9*	*Dynll1*
chr5	116421983	116422877	*Hspb8*	
chr5	117616331	117616540	*Nos1*	*Ksr2*
chr5	122627698	122627800	*P2rx7*	*Ift81*
chr5	125513346	125517138	*Tmem132b*	*Aacs*
chr5	125919867	125920590	*Gm4868*	
chr5	129359932	129360042	*Sfswap*	*Gpr133*
chr5	129362213	129362296	*Sfswap*	*Gpr133*
chr5	135734603	135734675	*Tmem120a*	*Por*
chr5	136825893	136826007	*Col26a1*	*Myl10*
chr5	142970278	142970360	*Fscn1*	*Rnf216*
chr5	143764266	143764618	*Eif2ak1*	*Usp42*
chr5	144422762	144422985	*Nptx2*	*Baiap2l1*
chr5	148371010	148371103	*Slc7a1*	*Mtus2*
chr5_GL456354_random	133401	133727		
chr6	8646617	8647856	*Ica1*	*Glcci1*
chr6	8647940	8648106	*Ica1*	*Glcci1*
chr6	8648188	8648710	*Ica1*	*Glcci1*
chr6	27768821	27768899	*Grm8*	
chr6	37612184	37612438	*Trim24*	*Akr1d1*
chr6	77793642	77794163	*Ctnna2*	*Lrrtm1*
chr6	88226890	88226964	*Dnajb8*	*Eefsec*
chr6	126103157	126103279	*Ntf3*	*Ano2*
chr6	127800917	127801126	*Tspan11*	*Prmt8*
chr6	145423950	145425004	*Kras*	*Ifltd1*
chr6	147173638	147173890	*Klhl42*	*Pthlh*
chr6	147173989	147174111	*Klhl42*	*Pthlh*
chr7	4098589	4100226	*Lair1*	*Ttyh1*
chr7	4145933	4146073	*Leng9*	*Leng8*
chr7	6433707	6436137	*Olfr1344*	
chr7	11504758	11504857	*Zscan4f*	*Vmn1r72*
chr7	11970076	11972567	*Vmn1r77*	*Vmn1r76*
chr7	18681119	18681458	*Psg21*	*Psg20*
chr7	18786400	18787013	*Psg19*	*Psg22*
chr7	25558673	25560789	*Ceacam2*	*Gm7092*
chr7	27842988	27843277	*Zfp59*	
chr7	28906807	28907381	*Capn12*	*Actn4*
chr7	30765081	30766071	*Krtdap*	*Dmkn*
chr7	30859833	30861818	*Ffar3*	*Ffar1*
chr7	31148427	31148542	*Scn1b*	*Gramd1a*
chr7	31148600	31148820	*Scn1b*	*Gramd1a*
chr7	31161796	31162945	*Gramd1a*	*Scgb1b2*
chr7	34992932	34993332	*Cebpg*	*Pepd*
chr7	37446498	37447571	*Zfp536*	*Tshz3*
chr7	37447713	37449778	*Zfp536*	*Tshz3*
chr7	38182897	38184369	*1600014C10Rik*	
chr7	38190694	38190819	*1600014C10Rik*	*Plekhf1*
chr7	38191076	38191482	*1600014C10Rik*	*Plekhf1*
chr7	38200606	38201249	*1600014C10Rik*	*Plekhf1*
chr7	38293615	38294026	*Pop4*	*Gm5591*
chr7	38314592	38314680	*Pop4*	*Gm5591*
chr7	38404238	38404934	*Pop4*	*Gm5591*
chr7	38408940	38409151	*Pop4*	*Gm5591*
chr7	38412409	38412830	*Pop4*	*Gm5591*
chr7	38450235	38453451	*Pop4*	*Gm5591*
chr7	38500033	38500988	*Pop4*	*Gm5591*
chr7	38529638	38530268	*Gm5591*	
chr7	38569969	38570372	*Gm5591*	*Gm5114*

Supplementary Table 4. H3K4me3 differential peaks identified in F3 - *(Continued)*

chr	start	end	gene 1	gene 2
chr7	38570616	38570689	*Gm5591*	*Gm5114*
chr7	38571726	38571917	*Gm5591*	*Gm5114*
chr7	38592811	38593803	*Gm5591*	*Gm5114*
chr7	38680699	38681268	*Gm5591*	*Gm5114*
chr7	38722590	38722927	*Gm5591*	*Gm5114*
chr7	38723488	38723604	*Gm5591*	*Gm5114*
chr7	38724441	38724600	*Gm5591*	*Gm5114*
chr7	38791237	38791507	*Gm5591*	*Gm5114*
chr7	38791579	38791873	*Gm5591*	*Gm5114*
chr7	38823963	38824208	*Gm5591*	*Gm5114*
chr7	38824343	38824481	*Gm5591*	*Gm5114*
chr7	38824680	38824907	*Gm5591*	*Gm5114*
chr7	38828227	38828730	*Gm5591*	*Gm5114*
chr7	38868731	38870805	*Gm5591*	*Gm5114*
chr7	38927363	38928036	*Gm5591*	*Gm5114*
chr7	39177711	39181069	*Gm5591*	*Gm5114*
chr7	39181220	39181814	*Gm5591*	*Gm5114*
chr7	39181870	39181982	*Gm5591*	*Gm5114*
chr7	39222588	39223654	*Gm5591*	*Gm5114*
chr7	39282571	39283249	*Gm5591*	*Gm5114*
chr7	39365612	39365812	*Gm5591*	*Gm5114*
chr7	39365970	39366253	*Gm5591*	*Gm5114*
chr7	39368155	39368380	*Gm5591*	*Gm5114*
chr7	39410579	39410740	*Gm5591*	*Gm5114*
chr7	39410812	39411882	*Gm5591*	*Gm5114*
chr7	39971677	39971931	*Vstm2b*	*Gm2058*
chr7	39971996	39972859	*Vstm2b*	*Gm2058*
chr7	40196026	40196158	*Vstm2b*	*Gm2058*
chr7	40199180	40200068	*Vstm2b*	*Gm2058*
chr7	41040160	41040760	*Gm2128*	*Gm4884*
chr7	41040849	41041386	*Gm2128*	*Gm4884*
chr7	41042701	41042779	*Gm2128*	*Gm4884*
chr7	41090440	41092819	*Gm2128*	*Gm4884*
chr7	41117678	41118882	*Gm2128*	*Gm4884*
chr7	41151824	41156637	*Gm2128*	
chr7	41207185	41213900	*Gm5592*	*Gm2128*
chr7	41243891	41244678	*Gm5592*	*Gm2128*
chr7	41283049	41287060	*Gm5592*	
chr7	41287112	41287345	*Gm5592*	*AI987944*
chr7	41298704	41299043	*Gm5592*	*AI987944*
chr7	41299096	41299500	*Gm5592*	*AI987944*
chr7	42440068	42440555	*4933421I07Rik*	*Vmn2r61*
chr7	42448580	42448747	*4933421I07Rik*	
chr7	43112801	43114330	*Zfp936*	*Gm10351*
chr7	44228783	44228951	*Gm10109*	
chr7	58835951	58839184	*Ube3a*	*Atp10a*
chr7	60733168	60734163	*Snrpn*	
chr7	64028308	64028525	*Trpm1*	*Klf13*
chr7	68582999	68583522	*Pgpep1l*	*Gm5334*
chr7	75767571	75767743	*Klhl25*	*Akap13*
chr7	79306391	79306641	*Abhd2*	*Rlbp1*
chr7	97756559	97756631	*Pak1*	*Aqp11*
chr7	103576547	103576991	*Olfr616*	*Olfr617*
chr7	109357591	109358810	*Lmo1*	*Stk33*
chr7	109447088	109447436	*Stk33*	*Trim66*
chr7	113294957	113295763	*Btbd10*	*Arntl*
chr7	119352302	119353436	*Gpr139*	*Gp2*
chr7	127089284	127089425	*AI467606*	
chr7	127774972	127775050	*Setd1a*	

chr	start	end	gene 1	gene 2
chr7	138876509	138876675	*Ppp2r2d*	*Bnip3*
chr7	138882241	138882729	*Bnip3*	*Ppp2r2d*
chr7	143957487	143957613	*Shank2*	*Gm498*
chr8	27592336	27593124	*Poteg*	
chr8	28212497	28212839	*Poteg*	
chr8	32714289	32715023	*Nrg1*	*Wrn*
chr8	32949184	32950444	*Wrn*	
chr8	32997671	32998584	*Wrn*	
chr8	33045650	33048202	*Wrn*	
chr8	33208220	33209047	*Wrn*	
chr8	33639778	33640537	*Ubxn8*	*Ppp2cb*
chr8	33729554	33729649	*Gtf2e2*	*1700104B16Rik*
chr8	34066324	34066548	*Rbpms*	*Dctn6*
chr8	34078642	34082498	*Rbpms*	*Dctn6*
chr8	34119934	34120679	*Mboat4*	*Leprotl1*
chr8	34246806	34247721	*Gm4889*	*Tmem66*
chr8	34471231	34473975	*Dusp4*	*Gm4889*
chr8	34522779	34523829	*Dusp4*	*Gm4889*
chr8	34608800	34609024	*Dusp4*	*Gm4889*
chr8	34687756	34690285	*Dusp4*	*Gm4889*
chr8	34773791	34775795	*Dusp4*	*Gm4889*
chr8	35094605	35096851	*Ppp1r3b*	*Tnks*
chr8	35222300	35224868	*Tnks*	*Ppp1r3b*
chr8	51731609	51732149		
chr8	52669369	52669946	*Aga*	
chr8	78954239	78954403	*Lsm6*	*Zfp827*
chr8	79960809	79962561	*Hhip*	*Anapc10*
chr8	83355386	83355629	*Clgn*	*4933434I20Rik*
chr8	84935054	84935457	*Mast1*	*Dnase2a*
chr8	84935587	84935756	*Mast1*	*Dnase2a*
chr8	88285208	88286765	*Adcy7*	*Brd7*
chr8	88681784	88682747	*Cyld*	*Nod2*
chr8	92094164	92094673	*Irx3*	*Irx5*
chr8	92233608	92234532	*Irx3*	*Irx5*
chr8	94504120	94505565	*Cpne2*	*Nlrc5*
chr8	95300343	95300450	*Cngb1*	*Cngb1*
chr8	95300552	95300825	*Cngb1*	*Cngb1*
chr8	95437283	95440992	*Gtl3*	
chr8	104062845	104062976	*Cdh5*	
chr8	110742809	110742917	*Mtss1l*	*Il34*
chr8	115204732	115205179	*Maf*	*Wwox*
chr8	123425839	123428239	*Def8*	*Tubb3*
chr8	123434044	123434477	*Def8*	*Tubb3*
chr9	8477473	8478090	*Trpc6*	*1700128F08Rik*
chr9	9292010	9292871	*Arhgap42*	
chr9	9298316	9298814	*Arhgap42*	
chr9	9342389	9342984	*Arhgap42*	
chr9	9404495	9404577	*Arhgap42*	
chr9	13754314	13754799	*Mtmr2*	*Cep57*
chr9	13756611	13758958	*Mtmr2*	*Cep57*
chr9	14101146	14101257	*Sesn3*	*Fam76b*
chr9	14778133	14778718	*1700012B09Rik*	*Ankrd49*
chr9	19868185	19868983	*Olfr860*	*Olfr862*
chr9	24589893	24590373	*Dpy19l1*	*Cypt4*
chr9	28830934	28831190		
chr9	31268182	31269165	*Aplp2*	*Prdm10*
chr9	38893762	38894398	*Olfr930*	*Olfr926*
chr9	40607649	40608328	*Gramd1b*	*Gm21915*
chr9	40616757	40617875	*Gramd1b*	*Gm21915*

Supplementary Table 4. H3K4me3 differential peaks identified in F3 - *(Continued)*

chr	start	end	gene 1	gene 2
chr9	54556878	54556966	*Cib2*	*Sh2d7*
chr9	61124187	61124260	*Gm9869*	*Gm10655*
chr9	85648921	85649082	*Fam46a*	*Ibtk*
chr9	86904805	86907065	*Ripply2*	*Snap91*
chr9	93323457	93324063	*Plod2*	
chr9	93327327	93327655	*Plod2*	
chr9	97271344	97271820	*Slc25a36*	*Trim42*
chr9	97305060	97306260	*Slc25a36*	*Trim42*
chr9	97509355	97510390	*Trim42*	*Clstn2*
chr9	98274549	98274859	*2610303G11Rik*	*Nmnat3*
chr9	98277076	98280162	*2610303G11Rik*	*Nmnat3*
chr9	98285101	98285195	*2610303G11Rik*	*Nmnat3*
chr9	98285304	98287240	*2610303G11Rik*	*Nmnat3*
chr9	98298790	98300322	*Rbp1*	*Nmnat3*
chr9	98613904	98613976	*Prr23a*	*Mrps22*
chr9	98645121	98645603	*Prr23a*	*Mrps22*
chr9	100022367	100023987	*Sox14*	*Il20rb*
chr9	100817203	100818385	*Stag1*	*Pccb*
chr9	101269493	101271677	*9630041A04Rik*	*Ppp2r3a*
chr9	101631101	101632033	*Ppp2r3a*	*9630041A04Rik*
chr9	101940173	101941987	*9630041A04Rik*	*Ephb1*
chr9	102572515	102573008	*Cep63*	*Ky*
chr9	102689173	102689254	*Amotl2*	*Anapc13*
chr9	102961465	102963208	*Slco2a1*	*Ryk*
chr9	102997717	103000764	*Slco2a1*	*Ryk*
chr9	103000842	103001927	*Slco2a1*	*Ryk*
chr9	106492599	106492930	*Iqcf1*	*Rrp9*
chr9	107925538	107925732	*Actl11*	
chr9	109576430	109576609	*Fbxw15*	*Fbxw24*
chr9	112033413	112035524	*Stac*	*Arpp21*
chr9	114873522	114874146	*Cmtm8*	*Gpd1l*
chr9	115778028	115778683	*Stt3b*	*Gadl1*
chr9	116109623	116109695	*Tgfbr2*	*Gadl1*
chr9	120931837	120931920	*Ctnnb1*	
chrX	41727170	41727282	*Thoc2*	*Gria3*
chrX	67566293	67567940	*4933436I01Rik*	*Slitrk2*
chrX	75682976	75683051	*Rab39b*	*Pls3*
chrX	75683456	75683625	*Rab39b*	*Pls3*
chrX	75683684	75683778	*Rab39b*	*Pls3*
chrX	92068569	92068867	*Gm5941*	*Mageb5*
chrX	92071405	92072036	*Gm5941*	*Mageb5*
chrX	93920869	93920960	*Pdk3*	*AU015836*
chrX	93921334	93921532	*Pdk3*	*AU015836*
chrX	124142261	124142421	*Vmn2r121*	
chrX	124142490	124142644	*Vmn2r121*	
chrX	158494651	158495003	*Rps6ka3*	*Cnksr2*
chrX	164890930	164891466	*Mospd2*	*Asb9*
chrX	164893680	164895178	*Mospd2*	*Asb9*
chrX	170674243	170674544	*Asmt*	
chrX	170674610	170674789	*Asmt*	
chrX	170674945	170675115	*Asmt*	
chrX	170675710	170675892	*Asmt*	
chrY	40331479	40332153	*Gm20793*	

Supplementary Table 5. Targets of POU5F1 in F1

Gene	Gene Ontology – GO	Occupancy
1700108F19Rik	ND	down
1810011O10Rik	GO:1902806~regulation of cell cycle G1/S phase transition	down
2610035D17Rik	ND	down
4930558C23Rik	ND	up
9530068E07Rik	ND	down
C130026L21Rik	ND	down
Cdh11	GO:0007155~cell adhesion	down
Cdh2	GO:0098609~cell-cell adhesion	down
Cdh6	GO:0007155~cell adhesion	up
Cdk15	GO:0006468~protein phosphorylation	up
Cdk5r1	GO:0007213~G-protein coupled acetylcholine receptor signaling pathway	up
Csmd1	GO:0042593~glucose homeostasis	down
Dgat1	GO:0019915~lipid storage	up
Dhx35	GO:0006396~RNA processing	up
Disc1	GO:0016055~Wnt signaling pathway	up
Fbxl14	GO:0006511~ubiquitin-dependent protein catabolic process	down
Fgfr1op	GO:0008284~positive regulation of cell proliferation	down
Gpr135	GO:0007186~G-protein coupled receptor signaling pathway	up
Gramd3	GO:0042802~identical protein binding	down
Grrp1	ND	up
Hapln1	GO:0007155~cell adhesion	up
Lgi1	GO:0030307~positive regulation of cell growth	down
Lhx5	GO:0007267~cell-cell signaling	up
Lingo1	GO:0007165~signal transduction	up
Lrrtm1	GO:0046426~negative regulation of JAK-STAT cascade	down
Med13l	GO:0006355~regulation of transcription, DNA-templated	down
Myom2	GO:0007015~actin filament organization	down
Opcml	ND	down
Rapgef6	GO:0043547~positive regulation of GTPase activity	up
Rdh10	GO:0008406~gonad development	down
Rgs7	GO:0007186~G-protein coupled receptor signaling pathway	up
Ror1	GO:0016055~Wnt signaling pathway	down
Rreb1	GO:0006355~regulation of transcription, DNA-templated	down
Sbp	GO:0030246~carbohydrate binding	up
Sema3f	GO:0050919~negative chemotaxis	up
Slc19a3	GO:0055085~transmembrane transport	down
Slc35f1	GO:0055085~transmembrane transport	down
Tbl1xr1	GO:0016575~histone deacetylation	down
Tesc	GO:0008584~male gonad development	up
Thsd4	GO:0048251~elastic fiber assembly	up
Tiam2	GO:0035023~regulation of Rho protein signal transduction	down
Tmem132d	GO:0010923~negative regulation of phosphatase activity	down
Trim24	GO:0071391~cellular response to estrogen stimulus	up
Trim32	GO:1902230~negative regulation of intrinsic apoptotic signaling pathway in response to DNA damage	down
Trpc7	GO:0006816~calcium ion transport	down
Unc5cl	GO:0043123~positive regulation of I-kappaB kinase/NF-kappaB signaling	up
Wipf2	GO:0006897~endocytosis	down
Wscd2	ND	down
Xylt1	GO:0030166~proteoglycan biosynthetic process	down
Zfpm1	GO:0030154~cell differentiation	down

Supplementary Table 6. Targets of POU5F1 in F3

Gene	Gene Ontology – GO	Occupancy
4933406K04Rik	ND	down
9530068E07Rik	ND	down
B4galt5	GO:0005975~carbohydrate metabolic process	down
Ccdc57	ND	down
Cdh2	GO:0098609~cell-cell adhesion	up
Cntnap5a	GO:0007155~cell adhesion	up
Ctnnd2	GO:0098609~cell-cell adhesion	down
Cxadr	GO:0098609~cell-cell adhesion	down
Dnajb8	GO:0061077~chaperone-mediated protein folding	down
E030025P04Rik	ND	down
Fntb	GO:0045787~positive regulation of cell cycle	down
Gapvd1	GO:0006897~endocytosis	down
Gm3985	ND	up
Hivep3	GO:0006355~regulation of transcription, DNA-templated	up
Kitl	GO:1902035~positive regulation of hematopoietic stem cell proliferation	down
Maf	GO:0006355~regulation of transcription, DNA-templated	up
Map1lc3a	GO:0000045~ autophagosome assembly	down
Mark1	GO:0016055~Wnt signaling pathway	down
Mc2r	GO:0007186~G-protein coupled receptor signaling pathway	up
Opcml	ND	down
Ralgapa2	GO:0032484~Ral protein signal transduction	both
Scarna17	ND	down
Slc5a10	GO:0055085~transmembrane transport	down
Stag1	GO:0007049~cell cycle	up
Tle4	GO:0016055~Wnt signaling pathway	up
Tlr4	GO:0007250~activation of NF-kappaB-inducing kinase activity	up
Ubxn8	GO:0030433~ubiquitin-dependent ERAD pathway	down
Vmn2r121	GO:0004930~G-protein coupled receptor activity	up

Supplementary Table 7. Targets of ZFP57 in F1

Gene	Gene Ontology – GO	Occupancy
1700007B14Rik	ND	up
1700019D03Rik	GO:0034237~protein kinase A regulatory subunit binding	up
1700040L02Rik	ND	down
3632451O06Rik	GO:0016020~membrane	up
4930503E14Rik	ND	up
4932411N23Rik	GO:0006357~regulation of transcription from RNA polymerase II promoter	down
4933405O20Rik	GO:0006099~tricarboxylic acid cycle	down
6430573F11Rik	GO:0002098~tRNA wobble uridine modification	up
9630013A20Rik	ND	up
Adam6a	GO:0007165~signal transduction	down
Anxa10	GO:0005509~calcium ion binding	up
Atp6ap1l	GO:0015991~ATP hydrolysis coupled proton transport	down
Bcl2l11	GO:0001701~in utero embryonic development	up
Bicd1	GO:0006810~transport	down
Cdh11	GO:0007155~cell adhesion	down
Cdh13	GO:0000278~mitotic cell cycle	down
Cdh2	GO:0007155~cell adhesion	down
Cdh7	GO:0007155~cell adhesion	up
Chd6	GO:0006351~transcription	down
Col19a1	GO:0007155~cell adhesion	down
Cps1	GO:0000050~urea cycle	down
Csmd1	GO:0001964~startle response	down
Ctnnbl1	GO:0006915~apoptotic process	down
Cwc22	GO:0000398~mRNA splicing	up
Cyp2j11	GO:0019369~arachidonic acid metabolic process	down
Dhx35	GO:0006396~RNA processing	up
Disc1	GO:0000226~microtubule cytoskeleton organization	up
Dsel	GO:0030204~chondroitin sulfate metabolic process	down
Fndc3a	GO:0007286~spermatid development	down
Frmd4a	GO:0090162~establishment of epithelial cell polarity	down
Fstl5	GO:0005509~calcium ion binding	down
G6pd2	GO:0005975~carbohydrate metabolic process	down
Gabrg1	GO:0006810~transport	down
Gm5622	ND	down
Gm6460	ND	up
Gm8267	GO:0016021~integral component of membrane	up
Gramd3	GO:0005881~cytoplasmic microtubule	down
Hapln1	GO:0001501~skeletal system development	up
Hey2	GO:0000122~negative regulation of transcription from RNA polymerase II promoter	up
Hs3st3b1	GO:0006477~protein sulfation	down
Hs3st5	GO:0006477~protein sulfation	down
Itga6	GO:0007155~cell adhesion	up
Klhl1	GO:0007626~locomotory behavior	up
Klhl25	GO:0006417~regulation of translation	up
Lrfn5	GO:0009986~cell surface	up
Lrrc4c	GO:0006469~negative regulation of protein kinase activity	up
Lsamp	GO:0007155~cell adhesion	down
Myom2	GO:0006936~muscle contraction	down
Ndfip2	GO:0007034~vacuolar transport	up

Supplementary Table 7. Targets of ZFP57 in F1 - *(Continued)*

Gene	Gene Ontology – GO	Occupancy
Nox3	GO:0001659~temperature homeostasis	down
Olfr1097	GO:0007186~G-protein coupled receptor signaling pathway	down
Pappa	GO:0006508~proteolysis	both
Pard3b	GO:0007049~cell cycle	up
Park2	GO:0000122~negative regulation of transcription from RNA polymerase II promoter	both
Pcdh17	GO:0007155~cell adhesion	up
Pcmtd1	GO:0006464~cellular protein modification process	down
Phf21a	GO:0000122~negative regulation of transcription from RNA polymerase II promoter	down
Rag2	GO:0002326~B cell lineage commitment	down
Ranbp3	GO:0006810~transport	up
Rapgef2	GO:0001568~blood vessel development	down
Slc19a3	GO:0006810~transport	down
Slc35f1	GO:0006810~transport	down
Slco5a1	GO:0006811~ion transport	down
Sphkap	GO:0010738~regulation of protein kinase A signaling	down
Spock1	GO:0001764~neuron migration	up
Spopl	GO:0030162~regulation of proteolysis	down
Steap1	GO:0006810~transport	up
Stxbp6	GO:0006887~exocytosis	down
Taf1B	GO:0000183~chromatin silencing at rDNA	down
Tbl1xr1	GO:0000122~negative regulation of transcription from RNA polymerase II promoter	down
Tmem132d	GO:0010923~negative regulation of phosphatase activity	down
Unc5d	GO:0006915~apoptotic process	up
Vmn1r77	GO:0016503~pheromone receptor activity	down
Zfp369	GO:0006351~transcription	down
Zfp804a	GO:0046872~metal ion binding	up
Zp4-ps	ND	down

Supplementary Table 8. Targets of ZFP57 in F3

Gene	Gene Ontology – GO	Occupancy
1700008P02Rik	ND	up
1700017N19Rik	ND	up
4921530L21Rik	ND	up
4930444G20Rik	GO:0006508~proteolysis	up
4932414N04Rik	ND	up
Aga	GO:0006508~proteolysis	up
Atf7ip2	GO:0006355~regulation of transcription, DNA-templated	down
Cdh2	GO:0007155~cell adhesion	up
Cdh5	GO:0007155~cell adhesion	down
Cdh6	GO:0007155~cell adhesion	up
Cdh9	GO:0007155~cell adhesion	up
Cntnap3	ND	up
Cntnap5a	GO:0006508~proteolysis	up
Cps1	GO:0000050~urea cycle	down
Csl	GO:0006099~tricarboxylic acid cycle	down
Ctnnbl1	GO:0006915~apoptotic process	up
Ctnnd2	GO:0001763~morphogenesis of a branching structure	down
Cwc27	GO:0000413~protein peptidyl-prolyl isomerization	down
E030025P04Rik	ND	down
Edil3	GO:0007155~cell adhesion	up
G6pd2	GO:0005975~carbohydrate metabolic process	down
Gm5458	GO:0016021~integral component of membrane	down
Gm5622	ND	down
Gm8267	GO:0016021~integral component of membrane	up
Gmeb1	GO:0006351~transcription	down
Gpc6	GO:0006024~glycosaminoglycan biosynthetic process	up
Gria2	GO:0001919~regulation of receptor recycling	down
Gtf2e2	GO:0006355~regulation of transcription, DNA-templated	down
Helb	GO:0006974~cellular response to DNA damage stimulus	down
Hhat	GO:0007275~multicellular organism development	down
Ica1	GO:0006810~transport	down
Itga6	GO:0007155~cell adhesion	up
Kcnj2	GO:0006810~transport	down
Kcnj3	GO:0006810~transport	up
Khdrbs2	GO:0006351~transcription	up
Klhl25	GO:0006417~regulation of translation	down
Lpar6	GO:0007165~signal transduction	up
Mageb1	ND	up
Mir128-2	GO:0005515~protein binding	up
Ndfip2	GO:0007034~vacuolar transport	up
Nhlrc1	GO:0000209~protein polyubiquitination	up
Nmnat2	GO:0009058~biosynthetic process	both
Nr4a2	GO:0000122~negative regulation of transcription from RNA polymerase II promoter	up
Pcdh17	GO:0007155~cell adhesion	up
Pcdh9	GO:0007156~homophilic cell adhesion via plasma membrane adhesion molecules	up
Pik3c3	GO:0000045~autophagosome assembly	up
Pla2g4a	GO:0001542~ovulation from ovarian follicle	down
Plrg1	GO:0000398~mRNA splicing	down
Pop4	GO:0006364~rRNA processing	down

Supplementary Table 8. Targets of ZFP57 in F3 - *(Continued)*

Gene	Gene Ontology – GO	Occupancy
Poteg	ND	down
Ppp2r3a	GO:0005509~calcium ion binding	up
Ppp6c	GO:0006470~protein dephosphorylation	down
Rag2	GO:0002326~B cell lineage commitment	up
Rapgef2	GO:0032486~Rap protein signal transduction	up
Sesn3	GO:0038203~TORC2 signaling	up
Snurf	GO:0051117~ATPase binding	down
Snx16	GO:0006622~protein targeting to lysosome	down
Tgfbr2	GO:0006915~apoptotic process	down
Tle4	GO:0016055~Wnt signaling pathway	up
Ube2e2	GO:0006974~cellular response to DNA damage stimulus	up
Vmn2r121	GO:0004930~G-protein coupled receptor activity	up
Wrn	GO:0000722~telomere maintenance via recombination	up

Supplementary Table 9. *De novo* H3K4me3 peaks identified in F1

chr	start	end	FC	pval	adj pval	GREAT Gene 1	Distance to TSS	GO Term Gene 1	GREAT Gene 2	Distance to TSS	GO Term Gene 2
chr1	16161821	16163027	350.11	3.78E-03	8.18E-02	*Rdh10*	56650	in utero embryonic development	*Stau2*	356882	regulation of gene expression
chr1	24594621	24594700	17.07	4.97E-03	8.18E-02	*Col19a1*	-7189	cell adhesion	*Gm10222*	18039	ND
chr1	67055668	67056368	15.15	2.46E-02	8.18E-02	*Cps1*	-67008	glutamine metabolic process	*Lancl1*	-17146	signal transduction
chr10	19293741	19293946	5.40	2.35E-02	8.18E-02	*Tnfaip3*	-278434	apoptotic process	*Olig3*	-62716	regulation of gene expression
chr10	19294335	19294488	6.86	5.35E-02	8.92E-02	*Tnfaip3*	-279002	apoptotic process	*Olig3*	-62148	regulation of gene expression
chr10	44563557	44563816	39.11	1.09E-02	8.18E-02	*Prep*	-503519	proteolysis	*Prdm1*	-105000	regulation of cell proliferation
chr10	44564079	44564441	49.43	7.29E-03	8.18E-02	*Prep*	-502946	proteolysis	*Prdm1*	-105573	regulation of cell proliferation
chr10	95316707	95318787	4.78	9.28E-03	8.18E-02	*Plxnc1*	-373169	negative regulation of cell adhesion	*Cradd*	6350	DNA damage response, signal transduction by p53 class mediator resulting in cell cycle arrest
chr11	5530606	5531525	19.88	1.66E-02	8.18E-02	*Xbp1*	10407	apoptotic process	*Ccdc117*	11121	ND
chr11	5534241	5534384	60.50	7.93E-03	8.18E-02	*Ccdc117*	7874	ND	*Xbp1*	13654	apoptotic process
chr11	5534772	5534994	41.10	4.49E-03	8.18E-02	*Ccdc117*	7304	ND	*Xbp1*	14224	apoptotic process
chr11	21891915	21892086	4.11	3.66E-02	8.50E-02	*Otx1*	109614	anterior/posterior pattern specification	*Wdpcp*	319766	embryonic organ development
chr12	24424938	24425258	3.12	2.65E-02	8.20E-02	*9030624G23Rik*	-328130	regulation of transcription, DNA-templated	*Gm16372*	68558	cell migration
chr12	41379337	41379429	3815.89	2.72E-03	8.18E-02	*Lrrn3*	106368	positive regulation of protein phosphorylation	*Immp2l*	355293	cellular response to DNA damage stimulus
chr12	42016276	42017888	11.15	9.99E-03	8.18E-02	*Lrrn3*	-531331	positive regulation of protein phosphorylation			

Supplementary Table 9. *De novo* H3K4me3 peaks identified in F1 - *(Continued)*

chr	start	end	FC	pval	adj pval	GREAT Gene 1	Distance to TSS	GO Term Gene 1	GREAT Gene 2	Distance to TSS	GO Term Gene 2
chr12	44928743	44931037	19.10	7.63E-03	8.18E-02	*Stxbp6*	144222	cell-cell adhesion	Nrcam	601005	cell-cell adhesion
chr13	17989547	17989647	13.64	9.08E-03	8.18E-02	*Rala*	-45358	cell cycle	Yae1d1	3754	translational initiation
chr13	53776089	53776453	3.80	2.00E-02	8.18E-02	*Gm5449*	250568	spliceosomal snRNP assembly	Drd1a	279387	G-protein coupled receptor signaling pathway
chr13	116731254	116731359	8.77	1.21E-02	8.18E-02	*Isl1*	-421619	cell differentiation	Parp8	294209	protein ADP-ribosylation
chr14	21787418	21788586	13.31	3.97E-03	8.18E-02	*Vdac2*	-43267	negative regulation of intrinsic apoptotic signaling pathway	Samd8	37471	ceramide biosynthetic process
chr14	21788788	21789442	5.97	1.38E-02	8.18E-02	*Vdac2*	-42154	negative regulation of intrinsic apoptotic signaling pathway	Samd8	38584	ceramide biosynthetic process
chr14	36088826	36089429	41.51	1.58E-03	8.18E-02	*4930596D02Rik*	-277150	ND	Gm7853	1688	ND
chr14	36089510	36089726	60.53	6.67E-04	7.41E-02	*4930596D02Rik*	-277640	ND	Gm7853	1198	ND
chr14	36089975	36090047	17.03	2.83E-03	8.18E-02	*4930474N05Rik*	-4954	hematopoietic progenitor cell differentiation	Gm7853	805	ND
chr14	41592430	41592562	4.81	3.43E-02	8.47E-02	*Gm7970*	-36913	ND	Gm3072	32116	ND
chr14	41592670	41592759	14.37	4.11E-02	8.63E-02	*Gm7970*	-37132	ND	Gm3072	31897	ND
chr14	41983655	41983763	9.84	5.74E-02	9.11E-02	*Gm3543*	-384	ND			
chr14	43108877	43108951	35.38	1.37E-02	8.18E-02	*Gm8094*	-32271	ND	Gm8122	126390	ND
chr14	43299523	43299625	100.43	5.50E-03	8.18E-02	*Gm9732*	702	ND			
chr14	44519035	44519199	21.24	3.50E-02	8.47E-02	*Gm8247*	-64036	ND	BC061237	18995	ND
chr14	44770274	44770377	2928.64	6.39E-03	8.18E-02	*Gm8267*	-45339	ND	Ptgdr	89049	signal transduction
chr14	48073229	48074062	6.07	1.52E-02	8.18E-02	*Peli2*	-47223	Toll signaling pathway	Ktn1	409890	microtubule-based movement
chr14	51182174	51182533	40.71	3.80E-03	8.18E-02	*Ang2*	13569	cellular response to glucose stimulus	Ear5	20094	endonuclease activity
chr14	51572076	51572586	228.54	5.03E-03	8.18E-02	*Gm5622*	19541	ND	Gm4181	63335	ND
chr14	51572868	51572973	87.38	5.86E-04	7.41E-02	*Gm5622*	20131	ND	Gm4181	62745	ND
chr14	51573101	51573192	6704.65	2.16E-03	8.18E-02	*Gm5622*	20357	ND	Gm4181	62519	ND
chr14	51612200	51612306	12.81	1.66E-02	8.18E-02	*Gm4181*	23413	ND	Gm5622	59463	ND
chr14	51633576	51633748	189.16	2.76E-02	8.36E-02	*Gm4181*	2004	ND	Gm5622	80872	ND
chr14	51634094	51634289	478.53	5.15E-03	8.18E-02	*Gm4181*	1474	ND	Gm5622	81402	ND
chr14	51635143	51635299	342.72	4.41E-02	8.71E-02	*Gm4181*	445	ND			
chr14	51635901	51635979	4850.14	2.53E-02	8.18E-02	*Gm4181*	-274	ND			
chr14	51677392	51678009	607.64	2.32E-03	8.18E-02	*Gm4181*	-42035	ND	Gm5800	39482	ND
chr14	68103170	68103259	95.96	7.78E-03	8.18E-02	*Nefl*	19352	microtubule cytoskeleton organization	Nefm	21631	microtubule cytoskeleton organization
chr14	84073107	84075274	40.73	3.50E-02	8.47E-02	*Pcdh17*	-369372	cell adhesion			
chr14	84075463	84077318	46.30	2.99E-02	8.47E-02	*Pcdh17*	-367172	cell adhesion			
chr14	97051875	97053042	6.47	1.16E-02	8.18E-02	*Klhl1*	-533357	protein ubiquitination			
chr14	97063200	97063529	15.74	1.04E-02	8.18E-02	*Klhl1*	-544263	protein ubiquitination			
chr15	60992367	60993108	3.84	1.78E-02	8.18E-02	*Myc*	-992653	cell proliferation	A1bg	-71468	extracellular matrix
chr15	85063183	85063447	55.20	2.09E-02	8.18E-02	*Gm10923*	-53514	ND	Fam118a	26253	ND
chr15	98037053	98037176	55.23	1.53E-02	8.18E-02	*Col2a1*	-32420	skeletal system development	Senp1	56629	apoptotic signaling pathway
chr16	41548211	41548840	31.03	1.75E-03	8.18E-02	*Lsamp*	15668	cell adhesion	Gap43	792125	cell differentiation
chr17	7985759	7985840	8.25	6.00E-03	8.18E-02	*Rsph3a*	40147	ND	Rnaset2a	162297	endoribonuclease activity

Supplementary Table 9. *De novo* H3K4me3 peaks identified in F1 - *(Continued)*

chr	start	end	FC	pval	adj pval	GREAT Gene 1	Distance to TSS	GO Term Gene 1	GREAT Gene 2	Distance to TSS	GO Term Gene 2
chr17	87012178	87012442	4.87	2.02E-02	8.18E-02	*Pigf*	13096	GPI anchor biosynthetic process	Rhoq	49228	Rho protein signal transduction
chr18	16463548	16465148	8.54	6.78E-02	9.94E-02	*Cdh2*	344898	cell-cell adhesion	Gm10036	631554	ribosomal large subunit assembly
chr18	18483634	18483869	4.13	3.46E-02	8.47E-02						
chr19	18274224	18274748	3.30	2.52E-02	8.18E-02	*Pcsk5*	-436854	embryo implantation	Ostf1	357303	SH3 domain binding
chr19	21804329	21804854	26.21	1.01E-02	8.18E-02	*Trpm3*	-334527	cation transport	Tmem2	26250	angiogenesis
chr2	23694816	23694895	83.32	3.53E-02	8.47E-02	*Spopl*	-122752	regulation of proteolysis	Hnmt	354538	response to tumor cell
chr2	26395577	26395803	4.50	1.89E-02	8.18E-02	*Pmpca*	6351	protein processing	Inpp5e	13510	dephosphorylation
chr2	38571494	38571568	5.46	1.65E-02	8.18E-02	*Nek6*	59888	cell cycle	Psmb7	72556	proteolysis
chr2	147248978	147249147	4.95	2.17E-02	8.18E-02	*Pax1*	-115931	cell proliferation	6430503K07Rik	61639	ND
chr2	156391806	156391888	4914.02	3.74E-02	8.58E-02	*Scand1*	-79143	ND	Epb4.1l1	-29062	actomyosin structure organization
chr2	156779834	156780892	6.73	3.51E-02	8.47E-02	*Tgif2*	-59714	regulation of transcription, DNA-templated	Myl9	4943	calcium ion binding
chr2	156781559	156781640	8.54	1.44E-02	8.18E-02	*Tgif2*	-58477	regulation of transcription, DNA-templated	Myl9	6180	calcium ion binding
chr2	157597632	157597846	8.30	3.66E-02	8.50E-02	*Ctnnbl1*	-139662	apoptotic process	Blcap	-31378	cell cycle
chr3	22413525	22413598	5.56	1.07E-02	8.18E-02	*Tbl1xr1*	336910	histone deacetylation			
chr3	76731885	76732303	3.31	2.20E-02	8.18E-02	*Fstl5*	657824	calcium ion binding			
chr4	100904678	100905241	3.14	2.58E-02	8.18E-02	*Gm10577*	115736	ND	Cachd1	128285	calcium ion transport
chr5	3896921	3897018	112.78	2.80E-02	8.36E-02	*Mterf*	-3063	regulation of transcription, DNA-templated			
chr5	4191778	4192192	24.13	3.93E-03	8.18E-02	*Gm9897*	-382	regulation of transcription, DNA-templated			
chr5	5504555	5505379	6.47	1.86E-02	8.18E-02	*1700015F17Rik*	-25824	protein import into nucleus	Cldn12	9822	structural molecule activity
chr5	10253099	10253190	46.06	3.08E-02	8.47E-02	*Gm5861*	-929927	ND	Gm10482	16316	integral component of membrane
chr5	21495389	21496468	8.43	7.91E-03	8.18E-02	*Lrrc17*	-47598	bone marrow development	Fam185a	70971	ND
chr5	76572202	76572689	31.46	5.31E-03	8.18E-02	*Cep135*	-16255	centriole replication	Exoc1	43123	exocytosis
chr5	76572806	76573013	88.40	3.91E-03	8.18E-02	*Cep135*	-15791	centriole replication	Exoc1	43587	exocytosis
chr5	76573161	76573497	54.71	1.19E-03	8.18E-02	*Cep135*	-15372	centriole replication	Exoc1	44006	exocytosis
chr5	76573894	76574928	48.80	1.17E-03	8.18E-02	*Cep135*	-14290	centriole replication	Exoc1	45088	exocytosis
chr5	76575322	76575535	20.86	1.11E-03	8.18E-02	*Cep135*	-13272	centriole replication	Exoc1	46106	exocytosis
chr5	76575629	76575856	18.21	2.75E-03	8.18E-02	*Cep135*	-12958	centriole replication	Exoc1	46420	exocytosis
chr5	76575955	76576232	36.52	4.02E-03	8.18E-02	*Cep135*	-12607	centriole replication	Exoc1	46771	exocytosis

Supplementary Table 9. *De novo* H3K4me3 peaks identified in F1 - *(Continued)*

chr	start	end	FC	pval	adj pval	GREAT Gene 1	Distance to TSS	GO Term Gene 1	GREAT Gene 2	Distance to TSS	GO Term Gene 2
chr5	77021650	77021723	8.06	1.84E-02	8.18E-02	*1700023E05Rik*	5664	ND	Hopx	93474	histone deacetylation
chr5	92242756	92243741	4.62	1.31E-02	8.18E-02	*Ppef2*	9910	calcium ion binding	Uso1	105311	ER to Golgi vesicle-mediated transport
chr5	121213668	121214141	4.91	1.63E-02	8.18E-02	*Gm15800*	-6314	protein ubiquitination	Rpl6	9424	ribosomal large subunit assembly
chr6	8045143	8045401	4.12	3.07E-02	8.47E-02	*Gm9825*	-61842	ND	Col28a1	147345	cell adhesion
chr6	54606761	54607003	4.86	1.24E-02	8.18E-02	*2410066E13Rik*	-74890	multicellular organism development	Plekha8	11771	lipid transport
chr6	76237723	76237930	3.52	2.27E-02	8.18E-02	*Gm9008*	259804	ND			
chr6	93715023	93715163	4.09	1.70E-02	8.18E-02	*Adamts9*	-813652	proteolysis	Magi1	568229	neuron death
chr6	139630446	139631825	6.62	8.09E-03	8.18E-02	*Pik3c2g*	-105759	phosphorylation	Pik3c2g	9248	phosphorylation
chr6	139631898	139632464	95.69	2.07E-02	8.18E-02	*Pik3c2g*	-104714	phosphorylation	Pik3c2g	10293	phosphorylation
chr6	139633726	139633990	4.16	2.32E-02	8.18E-02	*Pik3c2g*	-103037	phosphorylation	Pik3c2g	11970	phosphorylation
chr6	140242318	140242850	22.55	4.24E-02	8.70E-02	*Plekha5*	-181515	reproductive system development	Capza3	201059	spermatid development
chr7	45127000	45127085	5.90	1.19E-02	8.18E-02	*Rps11*	-2654	osteoblast differentiation			
chr7	138868417	138868496	29.22	1.37E-02	8.18E-02	*Ppp2r2d*	22098	cell cycle	Bnip3	41049	apoptotic process
chr7	138869686	138869847	44.87	5.44E-02	8.95E-02	*Ppp2r2d*	23408	cell cycle	Bnip3	39739	apoptotic process
chr7	138870390	138870506	53.22	2.64E-02	8.19E-02	*Ppp2r2d*	24089	cell cycle	Bnip3	39058	apoptotic process
chr7	138872877	138873043	52.30	4.39E-02	8.71E-02	*Ppp2r2d*	26601	cell cycle	Bnip3	36546	apoptotic process
chr7	138874128	138874279	32.23	4.07E-02	8.63E-02	*Ppp2r2d*	27845	cell cycle	Bnip3	35302	apoptotic process
chr7	138876463	138876708	42.35	3.42E-02	8.47E-02	*Ppp2r2d*	30227	cell cycle	Bnip3	32920	apoptotic process
chr7	144909510	144909965	4.08	1.82E-02	8.18E-02	*Oraov1*	-5397	double-strand break repair via homologous recombination	Fgf15	13206	positive regulation of cell proliferation
chr8	74998835	74998945	55.56	5.57E-04	7.41E-02	*Tom1*	-34796	intracellular protein transport	Hmgxb4	5534	chromatin remodeling
chr8	74999558	74999699	34.42	5.20E-04	7.41E-02	*Tom1*	-34057	intracellular protein transport	Hmgxb4	6273	chromatin remodeling
chr8	74999996	75000102	41.95	2.06E-02	8.18E-02	*Tom1*	-33637	intracellular protein transport	Hmgxb4	6693	chromatin remodeling
chr8	75000662	75001552	20.44	1.30E-03	8.18E-02	*Tom1*	-32579	intracellular protein transport	Hmgxb4	7751	chromatin remodeling
chr8	75024024	75024142	404.38	6.36E-03	8.18E-02	*Tom1*	-9603	intracellular protein transport	Hmgxb4	30727	chromatin remodeling
chr8	75029505	75029586	27.72	5.37E-03	8.18E-02	*Tom1*	-4140	intracellular protein transport			
chr8	92742104	92743416	3.59	2.06E-02	8.18E-02	*Mmp2*	-84568	aging	Irx6	68471	negative regulation of transcription, DNA-templated
chr8	96512749	96513089	78.61	3.90E-02	8.63E-02	*Got2*	-624372	biosynthetic process			
chr8	110127366	110129542	8.00	1.68E-02	8.18E-02	*Gm21964*	18682	ND	Calb2	39752	regulation of cytosolic calcium ion concentration
chr8	117007726	117008963	26.90	8.11E-03	8.18E-02	*Gcsh*	-14808	protein lipoylation	Pkd1l2	74104	detection of mechanical stimulus
chr8	125583155	125586750	43.25	7.62E-03	8.18E-02	*Sipa1l2*	-92243	regulation of small GTPase mediated signal transduction	Map10	-84865	cell cycle

Supplementary Table 9. *De novo* H3K4me3 peaks identified in F1 - *(Continued)*

chr	start	end	FC	pval	adj pval	GREAT Gene 1	Distance to TSS	GO Term Gene 1	GREAT Gene 2	Distance to TSS	GO Term Gene 2
chr9	22667520	22667595	6.19	1.72E-02	8.18E-02	*Bmper*	-555518	positive regulation of ERK1 and ERK2 cascade	Bbs9	191827	cell projection organization
chr9	89066216	89066293	11.40	5.40E-03	8.18E-02	*Trim43b*	26580	zinc ion binding	Bcl2a1a	109335	apoptotic process
chr9	89066352	89066444	9.47	3.73E-03	8.18E-02	*Trim43b*	26437	zinc ion binding	Bcl2a1a	109478	apoptotic process
chr9	111173266	111174502	3.31	4.54E-02	8.71E-02	*Lrrfip2*	55773	Wnt signaling pathway	Mlh1	97724	DNA repair

We calculated the average values of Fragments Per Kilobase Million (FPKM) for each peak for CD and control samples. Peaks with an average value of FPKM below the 5th centile (4.12) in control samples are considered as disappeared peaks.

Supplementary Table 10. H3K4me3 peaks disappeared in F1

chr	start	end	FC	pval	adj pval	GREAT Gene 1	Distance to TSS	GO Term Gene 1	GREAT Gene 2	Distance to TSS	GO Term Gene 2
chr1	57588464	57588548	4.49	1.31E-02	3.82E-02	*Spats2l*	-186355	ND	*Tyw5*	-181405	tRNA processing
chr1	59249275	59250478	22.39	4.49E-03	2.60E-02	*Als2*	-12646	Rac protein signal transduction	*Cdk15*	-7030	protein phosphorylation
chr1	61796329	61797311	6.52	7.86E-03	3.00E-02	*Nrp2*	-906882	cell differentiation	*Pard3b*	157996	cell cycle
chr1	86087766	86088809	14.56	6.15E-03	2.76E-02	*Psmd1*	23669	regulation of protein catabolic process	*Htr2b*	23682	ERK1 and ERK2 cascade
chr1	86983217	86984594	4.50	6.96E-03	2.79E-02	*Alppl2*	106022	metabolic process	*Dis3l2*	280102	cell cycle
chr1	108977513	108978338	16.70	5.59E-03	2.70E-02						
chr1	130175036	130175573	91.46	6.94E-04	2.03E-02	*Daf2*	247704	complement activation, classical pathway	*Thsd7b*	901961	ND
chr1	130175715	130175977	63.04	1.36E-03	2.23E-02	*Daf2*	247163	complement activation, classical pathway	*Thsd7b*	902502	ND
chr1	130547656	130548581	4.80	1.03E-02	3.37E-02	*Cd55*	-85375	complement activation, classical pathway	*Zp3r*	81502	binding of sperm to zona pellucida
chr1	134670841	134672129	10.08	9.24E-03	3.27E-02	*Syt2*	-37815	cell differentiation	*Kdm5b*	111314	histone H3-K4 demethylation
chr1	145164981	145165333	6.80	6.95E-03	2.79E-02	*Rgs18*	-389722	G-protein coupled receptor signaling pathway			
chr1	151329180	151329260	4.66	1.07E-02	3.43E-02	*Hmcn1*	-336169	cell cycle	*Ivns1abp*	-15278	negative regulation of intrinsic apoptotic signaling pathway
chr1	151329348	151329596	3.80	6.33E-03	2.76E-02	*Hmcn1*	-336421	cell cycle	*Ivns1abp*	-15026	negative regulation of intrinsic apoptotic signaling pathway
chr1	153431546	153432066	19.54	5.22E-03	2.61E-02	*Shcbp1l*	6644	spermatogenesis	*Dhx9*	55854	RNA splicing
chr1	164443480	164444698	18.72	6.50E-03	2.76E-02	*Atp1b1*	14266	cell adhesion	*Nme7*	131210	phosphorylation
chr1	175385704	175386525	10.31	6.35E-03	2.76E-02	*Grem2*	-464296	embryonic body morphogenesis	*Rgs7*	105015	G-protein coupled receptor signaling pathway
chr1	185255109	185256201	18.61	5.00E-03	2.61E-02	*Rab3gap2*	51487	intracellular protein transport	*Iars2*	73741	translation
chr1	190896550	190896835	4.59	1.13E-02	3.55E-02	*Prox1*	-726013	cell fate determination	*Rps6kc1*	15077	protein phosphorylation
chr10	31413866	31413946	3.39	3.77E-02	6.25E-02	*Tpd52l1*	32015	G2/M transition of mitotic cell cycle	*Hddc2*	100524	dUMP biosynthetic process
chr10	43296756	43297673	18.74	4.27E-03	2.58E-02	*Bend3*	-181925	histone H3-K4 trimethylation	*Pdss2*	75729	ubiquinone biosynthetic process

Supplementary Table 10. H3K4me3 peaks disappeared in F1 - *(Continued)*

chr	start	end	FC	pval	adj pval	GREAT Gene 1	Distance to TSS	GO Term Gene 1	GREAT Gene 2	Distance to TSS	GO Term Gene 2
chr10	60618744	60619827	19.64	5.77E-03	2.73E-02	*Cdh23*	77204	cell adhesion	*4632428N05Rik*	272435	stem cell differentiation
chr10	60966428	60967455	36.51	2.58E-03	2.48E-02	*Unc5b*	-135361	apoptotic process	*Pcbd1*	-122401	regulation of transcription, DNA-templated
chr10	64492630	64493433	10.42	3.70E-03	2.55E-02	*Lrrtm3*	-402758	negative regulation of JAK-STAT cascade			
chr10	70949329	70950468	10.68	3.84E-03	2.55E-02	*4930533K18Rik*	81230	ND	*Bicc1*	209801	negative regulation of canonical Wnt signaling pathway
chr10	73158341	73158886	3.64	1.76E-02	4.32E-02	*Pcdh15*	-663269	cell adhesion	*Zwint*	503719	cell cycle
chr10	83443024	83443936	12.74	6.76E-03	2.78E-02	*D10Wsu102e*	83259	ND	*Aldh1l2*	90660	oxidation-reduction process
chr10	86248449	86248524	12.06	5.98E-03	2.74E-02	*Timp3*	-51885	negative regulation of ERK1 and ERK2 cascade	*Fbxo7*	226515	negative regulation of G1/S transition of mitotic cell cycle
chr10	129674638	129675601	14.48	4.02E-03	2.55E-02	*Olfr801*	-4603	G-protein coupled receptor signaling pathway			
chr11	19701246	19702369	22.60	3.26E-03	2.51E-02	*Meis1*	-683094	angiogenesis	*Spred2*	-222634	regulation of protein deacetylation
chr11	19876934	19880377	7.75	3.26E-02	5.72E-02	*Meis1*	-859942	angiogenesis	*Spred2*	-45786	regulation of protein deacetylation
chr11	22762137	22763475	21.12	2.58E-02	5.18E-02	*B3gnt2*	97295	protein glycosylation	*Tmem17*	250718	cell projection organization
chr11	32101854	32103495	19.09	2.10E-03	2.36E-02	*Il9r*	97561	positive regulation of cell growth	*Nsg2*	102212	endosomal transport
chr11	52190074	52190260	4475.15	2.08E-02	4.73E-02	*Olfr1373*	-44639	G-protein coupled receptor signaling pathway	*Olfr1371*	23820	G-protein coupled receptor signaling pathway
chr11	54599921	54600143	13.62	6.19E-03	2.76E-02	*Rapgef6*	77185	positive regulation of GTPase activity	*Cdc42se2*	187643	phagocytosis
chr11	54600219	54600613	27.07	3.38E-03	2.51E-02	*Rapgef6*	77569	positive regulation of GTPase activity	*Cdc42se2*	187259	phagocytosis
chr11	59009921	59010956	20.68	4.40E-03	2.58E-02	*Trim11*	32323	innate immune response	*Obscn*	125936	cell differentiation
chr11	59302706	59302973	9.20	8.39E-03	3.10E-02	*Wnt9a*	-4090	Wnt signaling pathway			
chr11	60639482	60639571	8.05	1.55E-02	4.07E-02	*Llgl1*	-60196	regulation of protein secretion	*Alkbh5*	101549	cell differentiation
chr11	63676075	63676224	5.68	1.26E-02	3.76E-02	*Hs3st3b1*	246140	protein sulfation	*Pmp22*	547168	cell cycle
chr11	77959071	77959363	5.32	2.16E-02	4.78E-02	*Phf12*	-23585	negative regulation of transcription, DNA-templated	*Sez6*	28417	regulation of protein kinase C signaling
chr11	80604663	80605689	17.33	5.06E-03	2.61E-02	*Cdk5r1*	128130	G-protein coupled acetylcholine receptor signaling pathway	*Myo1d*	174849	negative regulation of phosphatase activity
chr11	80766308	80767133	23.76	8.44E-04	2.03E-02	*Myo1d*	13304	negative regulation of phosphatase activity	*Cdk5r1*	289675	negative regulation of transcription, DNA-templated
chr12	100499241	100500154	3.30	1.91E-02	4.49E-02	*Ttc7b*	21124	phosphatidylinositol phosphorylation	*Calm1*	300263	G2/M transition of mitotic cell cycle
chr13	10986424	10986534	4.16	5.02E-02	7.33E-02						
chr13	21878199	21879679	8.83	6.37E-03	2.76E-02	*Zfp184*	-66173	regulation of transcription, DNA-templated	*Hist1h2br*	45196	STAT family protein binding
chr13	29782167	29782596	13.13	4.09E-03	2.57E-02	*Sox4*	-828669	DNA damage response, detection of DNA damage	*Cdkal1*	73292	tRNA processing
chr13	31065589	31065856	4.21	3.22E-02	5.71E-02	*Foxq1*	-492447	cell differentiation	*Gm5447*	91519	ND
chr13	42948976	42949229	14.37	6.80E-03	2.78E-02	*Tbc1d7*	222398	activation of GTPase activity	*Phactr1*	266405	cell motility
chr13	52757576	52757927	11.67	5.21E-03	2.61E-02	*Auh*	171909	nuclear-transcribed mRNA catabolic process, deadenylation-dependent decay	*Syk*	174579	angiogenesis
chr13	53603825	53604988	4.37	1.55E-02	4.07E-02	*Gm5449*	78704	spliceosomal snRNP assembly	*Drd1a*	451251	G-protein coupled receptor signaling pathway

Supplementary Table 10. H3K4me3 peaks disappeared in F1 - *(Continued)*

chr	start	end	FC	pval	adj pval	GREAT Gene 1	Distance to TSS	GO Term Gene 1	GREAT Gene 2	Distance to TSS	GO Term Gene 2
chr13	54019382	54019745	10.74	1.17E-02	3.61E-02	*Drd1a*	36094	G-protein coupled receptor signaling pathway	*Gm5449*	493861	spliceosomal snRNP assembly
chr13	63892599	63893000	23.78	3.93E-03	2.55E-02	*Ercc6l2*	77466	DNA repair	*Hsd17b3*	196401	hormone metabolic process
chr13	90414974	90415258	4.57	1.67E-02	4.16E-02	*Xrcc4*	-325508	DNA repair	*Gm21726*	-168284	ND
chr14	30688251	30688575	18.42	5.24E-03	2.61E-02	*Sfmbt1*	-27202	chromatin organization	*Rft1*	34053	lipid transport
chr14	30688648	30689177	13.02	2.07E-03	2.36E-02	*Sfmbt1*	-26702	chromatin organization	*Rft1*	34553	lipid transport
chr14	31299716	31301175	7.46	4.03E-03	2.55E-02	*Dnahc1*	23450	flagellated sperm motility	*Bap1*	48957	regulation of cell cycle
chr14	41235189	41235359	4.83	6.30E-03	2.76E-02	*Sftpd*	-50076	immune system process	*Gm7945*	151105	ND
chr14	41278801	41279032	413.65	4.99E-03	2.61E-02	*Sftpd*	-93719	immune system process	*Gm7945*	107462	ND
chr14	41279089	41279302	93.84	1.07E-03	2.03E-02	*Sftpd*	-93998	immune system process	*Gm7945*	107183	ND
chr14	41322970	41323055	37.83	7.06E-04	2.03E-02	*Sftpd*	-137815	immune system process	*Gm7945*	63366	ND
chr14	41347935	41348026	3.70	2.66E-02	5.20E-02	*Sftpd*	-162783	immune system process	*Gm7945*	38398	ND
chr14	41384333	41384409	14.62	1.67E-03	2.29E-02	*Sftpd*	-199173	immune system process	*Gm7945*	2008	ND
chr14	41384486	41384632	3.59	1.36E-02	3.88E-02	*Sftpd*	-199361	immune system process	*Gm7945*	1820	ND
chr14	41384936	41385040	102.28	8.74E-05	1.47E-02	*Sftpd*	-199790	immune system process	*Gm7945*	1391	ND
chr14	41385345	41385453	142.78	4.97E-04	1.82E-02	*Gm7945*	980	ND			
chr14	41385716	41385791	8.16	2.66E-03	2.51E-02	*Gm7945*	625	ND			
chr14	41407306	41407408	117.25	3.44E-04	1.66E-02	*Gm6482*	924	ND			
chr14	41407829	41407918	18.70	2.90E-02	5.39E-02	*Gm6482*	407	ND			
chr14	41452668	41452770	6661.18	3.02E-04	1.66E-02	*Gm6482*	-44438	ND	*Gm3486*	36766	ND
chr14	41575070	41575153	4079.14	3.70E-02	6.18E-02	*Gm7970*	-19529	ND	*Gm3072*	49500	ND
chr14	41623175	41623263	143.02	1.82E-03	2.32E-02	*Gm7970*	-67636	ND	*Gm3072*	1393	ND
chr14	41623443	41623535	28.01	7.50E-04	2.03E-02	*Gm7970*	-67906	ND	*Gm3072*	1123	ND
chr14	41623591	41623736	6380.07	2.36E-04	1.66E-02	*Gm3072*	948	ND			
chr14	41624002	41624097	177.78	1.76E-03	2.31E-02	*Gm3072*	562	ND			
chr14	41645408	41645496	4001.08	8.52E-04	2.03E-02	*Gm3072*	-20840	ND	*Gm3676*	1035	ND
chr14	41646371	41646473	166.37	1.29E-03	2.15E-02	*Gm3676*	65	ND			
chr14	41700386	41700471	4085.84	2.15E-02	4.78E-02	*Gm3676*	-53942	ND	*Gm7929*	50166	ND
chr14	41700801	41700899	192.28	3.28E-03	2.51E-02	*Gm3676*	-54363	ND	*Gm7929*	49745	ND
chr14	41701265	41701477	9.51	8.23E-03	3.08E-02	*Gm3676*	-54884	ND	*Gm7929*	49224	ND
chr14	41771182	41771269	4326.72	1.18E-03	2.08E-02	*Gm7929*	-20631	ND	*Gm7980*	136064	ND
chr14	41772152	41772255	55.19	3.29E-03	2.51E-02	*Gm7929*	-21609	ND	*Gm7980*	135086	ND
chr14	42023195	42023282	52.97	3.18E-03	2.51E-02	*Gm3543*	-39914	ND	*Gm7951*	94130	ND
chr14	42039287	42039385	3477.17	1.40E-03	2.23E-02	*Gm3543*	-56011	ND	*Gm7951*	78033	ND
chr14	42039973	42040066	160.78	9.81E-04	2.03E-02	*Gm3543*	-56695	ND	*Gm7951*	77349	ND
chr14	42040844	42040941	170.55	2.21E-03	2.36E-02	*Gm3543*	-57568	ND	*Gm7951*	76476	ND
chr14	42093738	42093815	109.76	4.21E-03	2.57E-02	*Gm3543*	-110452	ND	*Gm7951*	23592	ND
chr14	42094413	42094503	220.16	2.30E-04	1.66E-02	*Gm3543*	-111133	ND	*Gm7951*	22911	ND
chr14	42094794	42094958	24.29	1.44E-03	2.24E-02	*Gm3543*	-111551	ND	*Gm7951*	22493	ND
chr14	42095199	42095272	11.71	1.58E-03	2.29E-02	*Gm3543*	-111911	ND	*Gm7951*	22133	ND
chr14	42161799	42161876	54.52	6.90E-03	2.79E-02	*Gm7951*	-44469	ND	*Gm3573*	28953	ND
chr14	42189652	42189724	3809.71	5.13E-03	2.61E-02	*Gm7951*	-72319	ND	*Gm3573*	1103	ND
chr14	42259129	42259225	38.20	1.69E-03	2.29E-02	*Gm3573*	-68386	ND	*Gm9611*	38181	ND
chr14	42259339	42259463	286.25	1.49E-04	1.66E-02	*Gm3573*	-68610	ND	*Gm9611*	37957	ND
chr14	42296194	42296400	4702.34	1.65E-03	2.29E-02	*Gm3573*	-105506	ND	*Gm9611*	1061	ND
chr14	42373224	42373312	107.46	5.65E-03	2.72E-02	*Gm7995*	63057	ND	*Gm8005*	66405	ND
chr14	42373486	42373569	4036.53	1.79E-03	2.31E-02	*Gm7995*	63317	ND	*Gm8005*	66145	ND
chr14	42374478	42374573	174.23	1.64E-03	2.29E-02	*Gm7995*	64315	ND	*Gm8005*	65147	ND
chr14	42438246	42438339	4085.84	7.40E-03	2.93E-02	*Gm8005*	1380	ND	*Gm7995*	128082	ND
chr14	42463784	42463968	60.73	2.05E-03	2.36E-02	*Gm8005*	-24203	ND	*Gm8020*	38473	ND
chr14	42464488	42464581	35.40	1.93E-03	2.35E-02	*Gm8005*	-24862	ND	*Gm8020*	37814	ND

Supplementary Table 10. H3K4me3 peaks disappeared in F1 - *(Continued)*

chr	start	end	FC	pval	adj pval	GREAT Gene 1	Distance to TSS	GO Term Gene 1	GREAT Gene 2	Distance to TSS	GO Term Gene 2
chr14	42502279	42502362	40.83	6.95E-03	2.79E-02	*Gm8020*	28	ND			
chr14	42523475	42523680	132.63	8.06E-04	2.03E-02	*Gm8024*	704	ND			
chr14	42524116	42524231	36.95	6.20E-03	2.76E-02	*Gm8024*	108	ND			
chr14	42599009	42599109	98.87	1.78E-02	4.34E-02	*Gm8024*	-74777	ND	*Gm3633*	42858	ND
chr14	42599190	42599262	3088.56	8.95E-03	3.23E-02	*Gm8024*	-74944	ND	*Gm3633*	42691	ND
chr14	42699355	42699573	89.10	3.99E-03	2.55E-02	*Gm10378*	43525	ND	*Gm8094*	377179	ND
chr14	42699717	42699817	108.50	3.38E-04	1.66E-02	*Gm10378*	43828	ND	*Gm8094*	376876	ND
chr14	42700732	42700831	6009.47	4.99E-04	1.82E-02	*Gm10378*	44843	ND	*Gm8094*	375861	ND
chr14	42797668	42797761	41.27	8.50E-04	2.03E-02	*Gm10378*	141776	ND	*Gm8094*	278928	ND
chr14	42853699	42853776	3177.73	1.27E-02	3.76E-02	*Gm10378*	197799	ND	*Gm8094*	222905	ND
chr14	43014970	43015189	59.13	1.08E-03	2.03E-02	*Gm8094*	61563	ND	*Gm10378*	359141	ND
chr14	43015498	43015585	62.93	6.36E-04	2.03E-02	*Gm8094*	61101	ND	*Gm10378*	359603	ND
chr14	43101391	43101507	5607.40	1.95E-03	2.35E-02	*Gm8094*	-24806	ND	*Gm8122*	133855	ND
chr14	43101908	43102004	4782.83	6.62E-04	2.03E-02	*Gm8094*	-25313	ND	*Gm8122*	133348	ND
chr14	43102282	43102373	60.99	1.76E-03	2.31E-02	*Gm8094*	-25685	ND	*Gm8122*	132976	ND
chr14	43149433	43149515	91.39	2.98E-03	2.51E-02	*Gm8094*	-72831	ND	*Gm8122*	85830	ND
chr14	43149572	43149668	6141.54	3.21E-04	1.66E-02	*Gm8094*	-72977	ND	*Gm8122*	85684	ND
chr14	43149768	43149852	51.02	2.10E-03	2.36E-02	*Gm8094*	-73167	ND	*Gm8122*	85494	ND
chr14	43150104	43150190	4668.84	1.15E-02	3.58E-02	*Gm8094*	-73504	ND	*Gm8122*	85157	ND
chr14	43258936	43259047	6239.06	2.56E-04	1.66E-02	*Gm8122*	-23688	ND	*Gm8127*	34821	ND
chr14	43259847	43259957	514.76	1.14E-03	2.08E-02	*Gm8122*	-24598	ND	*Gm8127*	33911	ND
chr14	43344973	43345048	12.36	9.97E-03	3.35E-02	*1700001F09Rik*	2800	ND	*Gm9732*	46139	ND
chr14	43345449	43345601	15.64	1.01E-02	3.37E-02	*1700001F09Rik*	2286	ND	*Gm9732*	46653	ND
chr14	43346412	43346521	18.67	3.32E-04	1.66E-02	*1700001F09Rik*	1344	ND	*Gm9732*	47595	ND
chr14	43414919	43415025	288.75	7.17E-04	2.03E-02	*1700001F09Rik*	-67161	ND	*Gm8138*	1378	ND
chr14	43415299	43415552	242.13	1.45E-05	1.34E-02	*Gm8138*	924	ND			
chr14	43607284	43607377	5499.27	4.06E-04	1.75E-02	*Gm10375*	682	ND			
chr14	43636431	43636504	6.03	1.40E-02	3.92E-02	*Gm10375*	-28455	ND	*Gm8165*	40731	ND
chr14	43736201	43736407	5.82	1.48E-02	4.01E-02	*Gm16506*	-8819	ND	*Ear1*	83335	ribonuclease activity
chr14	43747603	43747675	8.70	7.79E-03	2.99E-02	*Gm16506*	-20154	ND	*Ear1*	72000	ribonuclease activity
chr14	43925452	43925615	35.62	3.64E-03	2.55E-02	*Ear10*	-1975	ribonuclease activity	*Gm8113*	144	ND
chr14	44114432	44114749	4.11	1.51E-02	4.02E-02	*Gm3327*	-9260	ND	*Ear2*	11937	chemotaxis
chr14	44169419	44169702	4.28	4.59E-02	7.03E-02	*4930503E14Rik*	1810	ND	*Gm3327*	45710	ND
chr14	44171305	44171453	5.90	1.11E-02	3.51E-02	*4930503E14Rik*	-8	ND			
chr14	44196710	44196784	56.07	4.97E-03	2.61E-02	*Gm8212*	-914	ND			
chr14	44196841	44196936	121.22	3.33E-03	2.51E-02	*Gm8212*	-772	ND			
chr14	44197316	44197431	3920.61	2.77E-03	2.51E-02	*Gm8212*	-287	ND			
chr14	44197725	44198001	19.86	1.00E-03	2.03E-02	*Gm8212*	202	ND			
chr14	44198064	44198160	5584.45	1.16E-03	2.08E-02	*Gm8212*	451	ND			
chr14	44198231	44198568	351.00	6.09E-05	1.34E-02	*Gm8212*	739	ND			
chr14	44199206	44199628	17.07	9.30E-04	2.03E-02	*Gm8220*	-86163	ND	*Gm8212*	1756	ND
chr14	44212557	44213301	79.80	1.64E-03	2.29E-02	*Gm8220*	-72651	ND	*Gm8212*	15268	ND
chr14	44285166	44285304	3669.39	8.90E-03	3.23E-02	*Gm8220*	-345	ND			
chr14	44285461	44285559	4325.23	1.61E-03	2.29E-02	*Gm8220*	-70	ND			
chr14	44285666	44286078	51.97	1.94E-04	1.66E-02	*Gm8220*	292	ND			
chr14	44286168	44286472	71.50	6.98E-05	1.34E-02	*Gm8220*	740	ND			
chr14	44286605	44286700	6279.21	1.40E-03	2.23E-02	*Gm8229*	-78704	ND	*Gm8220*	1073	ND
chr14	44286825	44287105	197.65	4.51E-03	2.60E-02	*Gm8229*	-78392	ND	*Gm8220*	1385	ND
chr14	44287220	44287336	222.65	3.78E-04	1.75E-02	*Gm8229*	-78079	ND	*Gm8220*	1698	ND
chr14	44287395	44287626	124.86	8.05E-04	2.03E-02	*Gm8229*	-77846	ND	*Gm8220*	1931	ND
chr14	44311592	44311701	112.43	2.19E-03	2.36E-02	*Gm8229*	-53710	ND	*Gm8220*	26067	ND
chr14	44311939	44312028	6089.03	9.48E-04	2.03E-02	*Gm8229*	-53373	ND	*Gm8220*	26404	ND
chr14	44312107	44312302	136.18	3.73E-03	2.55E-02	*Gm8229*	-53152	ND	*Gm8220*	26625	ND
chr14	44364844	44365464	102.11	2.21E-03	2.36E-02	*Gm8229*	-203	ND			
chr14	44365589	44365876	244.86	1.67E-04	1.66E-02	*Gm8229*	376	ND			
chr14	44365982	44366687	26.90	2.22E-04	1.66E-02	*Gm8229*	978	ND			
chr14	44366771	44366878	34.44	4.19E-04	1.75E-02	*Gm8229*	1468	ND	*Gm3371*	42000	ND
chr14	44367024	44367399	107.56	1.84E-03	2.32E-02	*Gm8229*	1855	ND	*Gm3371*	41613	ND
chr14	44384136	44384315	5873.85	3.93E-03	2.55E-02	*Gm8229*	18869	ND	*Gm3371*	24599	ND
chr14	44384369	44384468	3573.51	4.36E-03	2.58E-02	*Gm8229*	19062	ND	*Gm3371*	24406	ND
chr14	44433133	44433361	52.59	4.99E-03	2.61E-02	*Gm8232*	-388	ND			

Supplementary Table 10. H3K4me3 peaks disappeared in F1 - *(Continued)*

chr	start	end	FC	pval	adj pval	GREAT Gene 1	Distance to TSS	GO Term Gene 1	GREAT Gene 2	Distance to TSS	GO Term Gene 2
chr14	44433532	44433732	327.13	4.71E-04	1.82E-02	*Gm8232*	-3	ND			
chr14	44433898	44434267	429.48	1.18E-03	2.08E-02	*Gm8232*	448	ND			
chr14	44434409	44434775	34.27	4.33E-05	1.34E-02	*Gm8232*	957	ND			
chr14	44434899	44435118	413.16	2.30E-03	2.38E-02	*BC061237*	-65113	ND	*Gm8232*	1374	ND
chr14	44435198	44435711	82.25	2.14E-03	2.36E-02	*BC061237*	-64667	ND	*Gm8232*	1820	ND
chr14	44500265	44500594	9.88	2.52E-04	1.66E-02	*BC061237*	308	ND			
chr14	44501245	44501761	14.91	8.40E-04	2.03E-02	*Gm8247*	-81650	ND	*BC061237*	1381	ND
chr14	44501884	44502578	151.81	4.81E-04	1.82E-02	*Gm8247*	-80922	ND	*BC061237*	2109	ND
chr14	44582731	44583178	11.14	3.49E-03	2.55E-02	*Gm8247*	-198	ND			
chr14	44583335	44583543	167.04	6.89E-05	1.34E-02	*Gm8247*	286	ND			
chr14	44583685	44584066	147.00	2.61E-05	1.34E-02	*Gm8247*	723	ND			
chr14	44584352	44585478	17.61	7.11E-04	2.03E-02	*Gm8247*	1762	ND	*Gm8267*	140072	ND
chr14	44613111	44613184	38.62	5.80E-03	2.73E-02	*Gm8247*	29995	ND	*Gm8267*	111839	ND
chr14	44613347	44613590	9.54	2.38E-03	2.38E-02	*Gm8247*	30316	ND	*Gm8267*	111518	ND
chr14	44613662	44614120	138.86	5.34E-05	1.34E-02	*Gm8247*	30738	ND	*Gm8267*	111096	ND
chr14	44614957	44615053	169.03	7.95E-04	2.03E-02	*Gm8247*	31852	ND	*Gm8267*	109982	ND
chr14	44615199	44615624	177.07	3.31E-03	2.51E-02	*Gm8247*	32259	ND	*Gm8267*	109575	ND
chr14	44627606	44627963	440.68	1.08E-03	2.03E-02	*Gm8247*	44632	ND	*Gm8267*	97202	ND
chr14	44656042	44656564	109.06	2.78E-03	2.51E-02	*Gm8267*	68684	ND	*Gm8247*	73150	ND
chr14	44656861	44657202	222.48	2.43E-03	2.41E-02	*Gm8267*	67955	ND	*Gm8247*	73879	ND
chr14	44686201	44686277	3985.57	4.05E-03	2.55E-02	*Gm8267*	38748	ND	*Gm8247*	103086	ND
chr14	44686367	44686681	11.82	3.04E-04	1.66E-02	*Gm8267*	38463	ND	*Gm8247*	103371	ND
chr14	44686741	44686964	9.60	2.87E-03	2.51E-02	*Gm8267*	38134	ND	*Gm8247*	103700	ND
chr14	44790397	44790527	26.75	3.67E-03	2.55E-02	*Gm8267*	-65475	ND	*Ptgdr*	68913	G-protein coupled receptor signaling pathway
chr14	49238254	49241134	39.54	9.75E-04	2.03E-02	*1700011H14Rik*	5734	ND	*Naa30*	67096	N-terminal protein amino acid acetylation
chr14	51007713	51009043	23.13	1.02E-02	3.37E-02	*Rnase10*	449	cell adhesion			
chr14	51363694	51363767	7.67	2.23E-03	2.37E-02	*Vmn2r88*	-47270	G-protein coupled receptor activity	*Gm7247*	1975	ND
chr14	51382216	51383474	74.30	1.03E-03	2.03E-02	*Vmn2r88*	-28156	G-protein coupled receptor activity	*Gm7247*	21089	ND
chr14	51694232	51694331	188.58	1.24E-03	2.09E-02	*Gm4181*	-58616	ND	*Gm5800*	22901	ND
chr14	51695676	51695970	151.65	4.52E-03	2.60E-02	*Gm4181*	-60157	ND	*Gm5800*	21360	ND
chr14	51748663	51748747	3888.75	3.72E-03	2.55E-02	*Gm5800*	-31522	ND	*Ang4*	24885	positive regulation of cell proliferation
chr14	76425801	76426730	7.59	4.82E-03	2.61E-02	*Tsc22d1*	10679	positive regulation of cell proliferation	*Serp2*	130421	protein transport
chr14	84018491	84018856	4.36	3.87E-02	6.35E-02	*Pcdh17*	-424889	cell adhesion			
chr14	84309955	84310041	4.47	3.03E-02	5.53E-02	*Pcdh17*	-133565	cell adhesion			
chr14	105218188	105218288	4.97	6.20E-03	2.76E-02	*Rbm26*	-40915	mRNA processing	*Ndfip2*	-40335	positive regulation of I-kappaB kinase/NF-kappaB signaling
chr14	120317178	120317715	3.96	1.10E-02	3.50E-02	*Rap2a*	-161014	Rap protein signal transduction	*Mbnl2*	41778	RNA splicing
chr15	35932855	35933663	6.82	7.79E-03	2.99E-02	*Cox6c*	4386	cytochrome-c oxidase activity	*Vps13b*	561713	protein transport
chr15	66694476	66695740	25.12	1.00E-02	3.35E-02	*Tg*	24355	hormone biosynthetic process	*Sla*	136721	cell differentiation
chr15	73208438	73209099	26.44	4.70E-03	2.60E-02	*Ago2*	-23929	miRNA mediated inhibition of translation	*Ptk2*	214422	MAPK cascade
chr15	76782894	76784169	4.69	1.07E-02	3.43E-02	*C030006K11Rik*	-59687	ND	*Gm17271*	-14096	ND
chr15	77193977	77194410	5.02	5.27E-03	2.62E-02	*Rbfox2*	112859	RNA splicing	*Apol6*	149119	lipid binding
chr15	77786773	77787896	10.02	4.78E-03	2.61E-02	*Apol8*	-32106	lipid binding	*Myh9*	54840	cell-cell adhesion
chr15	79509897	79510615	6.04	1.06E-02	3.43E-02	*Kdelr3*	-6152	vesicle-mediated transport	*Kcnj4*	-5015	potassium ion import
chr15	84202718	84204064	15.72	2.77E-03	2.51E-02	*Parvb*	-28652	cell adhesion	*Samm50*	11158	mitochondrial respiratory chain complex assembly

Supplementary Table 10. H3K4me3 peaks disappeared in F1 - *(Continued)*

chr	start	end	FC	pval	adj pval	GREAT Gene 1	Distance to TSS	GO Term Gene 1	GREAT Gene 2	Distance to TSS	GO Term Gene 2
chr15	84917771	84918539	7.54	6.69E-03	2.78E-02	*Phf21b*	-62106	regulation of transcription, DNA-templated	*Nup50*	-5273	protein import into nucleus
chr15	86414224	86415378	17.70	7.55E-03	2.96E-02	*Tbc1d22a*	200342	intracellular protein transport			
chr15	86582134	86582355	22.05	5.73E-03	2.73E-02	*Tbc1d22a*	367786	intracellular protein transport			
chr15	102259618	102259788	5.22	1.48E-02	4.01E-02	*Rarg*	-2186	bone morphogenesis			
chr16	11539282	11539756	15.57	4.68E-03	2.60E-02	*Gm9961*	-361844	ND	*Snx29*	133871	phosphatidylinositol binding
chr16	17596352	17597219	5.29	2.85E-03	2.51E-02	*Smpd4*	-22568	cellular response to tumor necrosis factor	*Slc7a4*	-20065	amino acid transport
chr16	17858074	17859034	14.10	4.18E-03	2.57E-02	*Car15*	-20190	metal ion binding	*Gm20518*	33117	ND
chr16	18355329	18356137	20.59	8.50E-03	3.13E-02	*Arvcf*	7459	cell-cell adhesion	*Comt*	57761	catecholamine catabolic process
chr16	21352898	21354117	19.88	2.08E-03	2.36E-02	*Vps8*	-69610	endosomal vesicle fusion	*Ephb3*	148753	negative regulation of apoptotic process
chr16	31547348	31548403	16.41	4.83E-03	2.61E-02	*Dlg1*	-116163	cell-cell adhesion	*Bdh1*	119128	oxidation-reduction process
chr16	35167602	35167817	8.65	5.78E-03	2.73E-02	*Adcy5*	12833	cAMP biosynthetic process	*Sec22a*	196208	vesicle-mediated transport
chr16	45031928	45032154	10.87	1.36E-02	3.88E-02	*Ccdc80*	-61571	positive regulation of cell-substrate adhesion	*Cd200r3*	88363	signaling receptor activity
chr16	48189836	48190992	4.63	1.67E-02	4.16E-02	*Dppa4*	-93321	lung-associated mesenchyme development	*Gm7275*	116970	protein desumoylation
chr16	97543237	97544083	18.60	2.97E-03	2.51E-02	*Mx2*	7577	innate immune response	*Tmprss2*	67535	proteolysis
chr17	5829576	5829948	34.51	3.86E-03	2.55E-02	*Snx9*	-11566	cell cycle	*Zdhhc14*	337162	protein palmitoylation
chr17	6962732	6962830	8427.51	5.69E-03	2.73E-02	*Tagap1*	-1625	GTPase activator activity			
chr17	7987023	7987114	13.24	1.80E-03	2.31E-02	*Rsph3a*	41416	ND	*Rnaset2a*	161028	RNA catabolic process
chr17	10198524	10199579	6.00	9.46E-03	3.31E-02	*Pabpc6*	-529348	ND	*Qk*	120309	RNA splicing
chr17	10279302	10280076	9.29	5.09E-03	2.61E-02	*Pabpc6*	-609985	ND	*Qk*	39672	RNA splicing
chr17	10280136	10280426	15.00	3.82E-03	2.55E-02	*Pabpc6*	-610577	ND	*Qk*	39080	RNA splicing
chr17	11143810	11145681	17.95	2.13E-02	4.78E-02	*Park2*	-693856	dopamine metabolic process	*Park2*	77621	dopamine metabolic process
chr17	23945564	23945680	5.99	8.74E-03	3.18E-02	*Sbp*	3789	ND	*Sbpl*	9297	ND
chr17	30647815	30648762	3.83	2.15E-02	4.78E-02	*Glp1r*	-253578	G-protein coupled receptor signaling pathway	*Dnahc8*	23935	microtubule-based movement
chr17	30888594	30888706	23.05	3.33E-03	2.51E-02	*Glp1r*	-13217	G-protein coupled receptor signaling pathway	*Dnahc8*	264296	microtubule-based movement
chr17	31231268	31233320	16.52	1.01E-03	2.03E-02	*Ubash3a*	24421	regulation of cytokine production	*Rsph1*	45062	meiotic cell cycle
chr17	31366464	31366710	6.32	7.90E-03	3.00E-02	*Pde9a*	-19647	cGMP metabolic process	*Slc37a1*	70368	transmembrane transport
chr17	31366838	31367571	9.20	2.88E-03	2.51E-02	*Pde9a*	-19029	cGMP metabolic process	*Slc37a1*	70986	transmembrane transport
chr17	31471637	31473014	5.46	3.40E-03	2.51E-02	*Wdr4*	40425	tRNA processing	*Pde9a*	86092	cGMP catabolic process
chr17	31943173	31944561	6.37	1.70E-02	4.22E-02	*Sik1*	-88075	cell cycle	*Hsf2bp*	90641	protein binding
chr17	47446355	47447258	20.95	3.30E-03	2.51E-02	*1700001C19Rik*	-9432	ND	*Taf8*	55480	cell differentiation
chr17	82955429	82957273	19.10	1.71E-02	4.22E-02	*Pkdcc*	-258941	cell differentiation	*Gm6594*	417093	ND
chr17	82957340	82957724	54.26	1.56E-02	4.08E-02	*Pkdcc*	-257760	cell differentiation	*Gm6594*	418274	ND
chr17	87257904	87258423	20.58	1.39E-02	3.91E-02	*Mcfd2*	7740	negative regulation of cell death	*Socs5*	150485	JAK-STAT cascade
chr17	87258790	87260022	19.22	1.32E-02	3.83E-02	*Mcfd2*	6498	negative regulation of cell death	*Socs5*	151727	JAK-STAT cascade

Supplementary Table 10. H3K4me3 peaks disappeared in F1 - *(Continued)*

chr	start	end	FC	pval	adj pval	GREAT Gene 1	Distance to TSS	GO Term Gene 1	GREAT Gene 2	Distance to TSS	GO Term Gene 2
chr18	4320605	4320783	5.14	1.47E-02	4.01E-02	*Map3k8*	32259	cell cycle	*Lyzl1*	154862	cell wall macromolecule catabolic process
chr19	10520220	10520809	12.90	5.82E-03	2.73E-02	*Cpsf7*	-4729	mRNA processing	*Sdhaf2*	4694	protein dephosphorylation
chr19	10669544	10670393	3.27	3.48E-02	5.96E-02	*Pga5*	8102	proteolysis	*Vwce*	35736	calcium ion binding
chr19	31126529	31127638	7.84	1.05E-03	2.03E-02	*Cstf2t*	44243	mRNA processing	*Prkg1*	537286	phosphorylation
chr19	32799873	32800058	10.17	3.54E-03	2.55E-02	*Pten*	42469	canonical Wnt signaling pathway	*Rnls*	592299	phosphate ion homeostasis
chr19	60539451	60540328	20.03	5.34E-03	2.63E-02	*Prlhr*	-71586	G-protein coupled receptor signaling pathway	*Cacul1*	41133	cell cycle
chr19	61152702	61152794	5.36	2.65E-02	5.20E-02	*Gm7102*	23561	ND	*Grk5*	262999	regulation of cell cycle
chr19	61153890	61154078	5.28	6.30E-02	8.58E-02	*Gm7102*	22325	ND	*Grk5*	264235	regulation of cell cycle
chr2	38136954	38138561	5.97	7.55E-03	2.96E-02	*Dennd1a*	149617	endocytosis	*Crb2*	361509	multicellular organism development
chr2	77918997	77919655	6.15	1.38E-02	3.91E-02	*Zfp385b*	-199587	apoptotic process	*Cwc22*	27049	RNA splicing
chr2	77924980	77926668	18.64	1.06E-03	2.03E-02	*Zfp385b*	-206085	apoptotic process	*Cwc22*	20551	RNA splicing
chr2	77947145	77947689	6.61	1.36E-02	3.88E-02	*Cwc22*	-1042	RNA splicing			
chr2	77947774	77948277	4.49	1.77E-02	4.32E-02	*Cwc22*	-1651	RNA splicing			
chr2	79678832	79680495	5.18	6.85E-03	2.78E-02	*Ppp1r1c*	-28116	cell cycle	*Ssfa2*	44312	actin binding
chr2	92655357	92656145	28.14	3.86E-03	2.55E-02	*Syt13*	-259347	vesicle-mediated transport	*Chst1*	56044	carbohydrate metabolic process
chr2	93448041	93448960	3.85	4.40E-02	6.85E-02	*Tspan18*	-114014	cell surface receptor signaling pathway	*Cd82*	14001	cell surface receptor signaling pathway
chr2	98107922	98108935	5.25	3.93E-03	2.55E-02	*Gm10801*	-553808	ND			
chr2	128178999	128179814	14.91	4.12E-03	2.57E-02	*Gm355*	-411802	regulation of protein kinase activity	*Bcl2l11*	53368	meiosis I
chr2	150589336	150590507	10.51	4.41E-03	2.58E-02	*Apmap*	18645	biosynthetic process	*Cst7*	19507	negative regulation of peptidase activity
chr2	153426337	153426575	30.69	3.04E-03	2.51E-02	*4930404H24Rik*	-66334	ND	*Asxl1*	80611	chromatin organization
chr2	155781927	155782207	3.68	4.42E-02	6.87E-02	*Mmp24*	6725	cell adhesion	*Eif6*	44858	gene silencing by miRNA
chr2	157988953	157990355	5.52	6.86E-03	2.78E-02	*Tti1*	38779	regulation of TOR signaling	*Vstm2l*	75001	negative regulation of neuron apoptotic process
chr2	158841032	158841115	5.51	1.62E-02	4.15E-02	*Dhx35*	46267	RNA processing			
chr2	168433956	168434199	27.25	5.22E-03	2.61E-02	*Kcng1*	-152946	potassium ion transport	*Nfatc2*	156182	cellular response to DNA damage stimulus
chr2	180875322	180876215	14.75	2.46E-03	2.42E-02	*Bhlhe23*	-98869	cell development	*Ythdf1*	45180	positive regulation of translation
chr2	181336926	181338117	7.21	1.02E-02	3.37E-02	*Zgpat*	-27406	regulation of transcription, DNA-templated	*Rtel1*	17760	DNA repair
chr2	181811354	181812877	13.15	1.23E-02	3.71E-02	*Pcmtd2*	-25738	cellular protein modification process	*Myt1*	48784	regulation of transcription, DNA-templated
chr3	7415871	7418077	3.06	7.60E-02	9.78E-02	*Zc2hc1a*	-86452	metal ion binding	*Pkia*	50370	regulation of G2/M transition of mitotic cell cycle
chr3	22299842	22303763	26.32	2.20E-03	2.36E-02	*Tbl1xr1*	225151	histone deacetylation			
chr3	61943713	61944611	8.63	4.27E-03	2.58E-02	*B430305J03Rik*	-578211	ND	*Arhgef26*	-394182	endothelial cell morphogenesis
chr3	61944753	61944986	7.46	5.21E-03	2.61E-02	*B430305J03Rik*	-578919	ND	*Arhgef26*	-393474	endothelial cell morphogenesis
chr3	73532286	73532837	12.97	7.08E-03	2.83E-02	*Slitrk3*	-475619	positive regulation of synapse assembly	*Bche*	175853	negative regulation of cell proliferation
chr3	87758250	87759923	8.21	1.92E-03	2.35E-02	*Lrrc71*	-10464	intracellular signal transduction	*Pear1*	9856	recognition of apoptotic cell
chr3	95395268	95395995	12.22	5.20E-03	2.61E-02	*Setdb1*	-38432	histone H3-K9 trimethylation	*Gm5070*	15544	ND

Supplementary Table 10. H3K4me3 peaks disappeared in F1 - *(Continued)*

chr	start	end	FC	pval	adj pval	GREAT Gene 1	Distance to TSS	GO Term Gene 1	GREAT Gene 2	Distance to TSS	GO Term Gene 2
chr3	95945677	95946388	8.71	9.14E-03	3.25E-02	*Anp32e*	16787	chromatin remodeling	*Plekho1*	49968	regulation of cell shape
chr3	107616063	107617705	12.42	1.95E-03	2.35E-02	*Gm10961*	-14438	ND	*Alx3*	21853	regulation of apoptotic process
chr3	108992175	108993454	14.13	3.89E-03	2.55E-02	*Gm9857*	-52370	ND	*Fam102b*	34792	ND
chr3	136482841	136483265	25.63	4.74E-03	2.61E-02	*Ppp3ca*	-187717	G1/S transition of mitotic cell cycle	*Bank1*	-157007	positive regulation of MAPK cascade
chr4	32964765	32964873	24.25	1.28E-04	1.66E-02	*Ankrd6*	-41364	negative regulation of canonical Wnt signaling pathway	*Rragd*	-18218	positive regulation of TOR signaling
chr4	63019293	63019971	95.86	4.59E-03	2.60E-02	*Zfp618*	54058	regulation of transcription, DNA-templated	*Ambp*	134541	protein catabolic process
chr4	107976377	107980153	21.03	2.13E-03	2.36E-02	*Slc1a7*	9933	anion transmembrane transport	*Podn*	53825	negative regulation of JAK-STAT cascade
chr4	135097930	135100487	10.59	4.03E-03	2.55E-02	*Runx3*	-21443	regulation of cell differentiation	*Syf2*	168311	mitotic G2 DNA damage checkpoint
chr4	137609760	137609894	12.09	5.21E-03	2.61E-02	*Rap1gap*	-70926	negative regulation of GTP binding	*Usp48*	15620	protein deubiquitination
chr4	138361517	138361664	5.35	7.72E-03	2.99E-02	*Pink1*	-35284	intracellular signal transduction	*Cda*	6401	negative regulation of cell growth
chr4	138361758	138362093	4.56	8.03E-03	3.03E-02	*Pink1*	-35619	intracellular signal transduction	*Cda*	6066	negative regulation of cell growth
chr4	139364512	139366278	28.41	2.00E-03	2.36E-02	*Ubr4*	-15264	ubiquitin-dependent protein catabolic process	*Emc1*	12802	protein folding in endoplasmic reticulum
chr4	140902291	140902933	9.89	6.09E-03	2.76E-02	*Padi2*	-3732	chromatin-mediated maintenance of transcription			
chr4	144978207	144979495	27.78	2.26E-03	2.38E-02	*Vps13d*	-4168	protein retention in Golgi apparatus			
chr4	147219393	147219652	4.72	1.49E-02	4.01E-02	*Gm13151*	-86151	regulation of transcription, DNA-templated	*Gm13139*	87485	regulation of transcription, DNA-templated
chr4	149548607	149549266	5.90	6.29E-03	2.76E-02	*Clstn1*	-37701	cell adhesion	*Ctnnbip1*	30701	Wnt signaling pathway
chr5	3691446	3693161	17.15	3.34E-03	2.51E-02	*4930511M11Rik*	35300	ND	*Ankib1*	110805	protein ubiquitination
chr5	5796676	5796766	6.05	1.45E-02	3.98E-02	*Steap1*	-47395	copper ion import			
chr5	6760553	6760891	4.60	2.43E-02	5.00E-02	*Zfp804b*	115801	ND			
chr5	10949957	10950093	5.50	2.40E-02	4.98E-02	*Gm5861*	-233047	ND	*Gm10482*	713196	ND
chr5	11253630	11253954	10.92	6.51E-03	2.76E-02	*Gm6460*	-341164	ND	*Gm5861*	70720	ND
chr5	11343202	11343280	6.34	1.87E-02	4.44E-02	*Gm6460*	-251715	ND	*Gm5861*	160169	ND
chr5	11504685	11504797	4.66	1.93E-02	4.53E-02	*Gm6460*	-90215	ND	*Gm5861*	321669	ND
chr5	11596664	11596745	4.49	3.61E-02	6.09E-02	*4933402N22Rik*	-321338	ND	*Gm6460*	1749	ND
chr5	11847638	11847710	12.69	4.86E-03	2.61E-02	*4933402N22Rik*	-70369	ND	*Gm6460*	252718	ND
chr5	23859625	23861061	8.03	7.72E-03	2.99E-02	*Tomm7*	-16182	protein transport	*Fam126a*	170347	myelination
chr5	38234925	38235766	13.32	6.13E-03	2.76E-02	*Tmem128*	-24839	ND	*Lyar*	14876	negative regulation of transcription by RNA polymerase II
chr5	73801646	73802438	25.26	1.47E-03	2.24E-02	*Spata18*	150662	cellular response to DNA damage stimulus	*Usp46*	266388	protein deubiquitination
chr5	74144075	74145469	5.11	2.86E-03	2.51E-02	*Usp46*	-76342	protein deubiquitination	*Rasl11b*	-50524	signal transduction
chr5	74145521	74146934	8.00	3.25E-03	2.51E-02	*Usp46*	-77798	protein deubiquitination	*Rasl11b*	-49068	signal transduction
chr5	77666623	77667805	28.47	4.25E-02	6.71E-02	*Igfbp7*	-259169	cell adhesion			
chr5	109907101	109907186	17.98	1.07E-02	3.43E-02	*4930522L14Rik*	-155547	regulation of transcription, DNA-templated	*Gm15446*	-16256	regulation of transcription, DNA-templated
chr5	110509037	110509188	22.33	4.37E-03	2.58E-02	*Fbrsl1*	-60610	ND	*Galnt9*	-35232	O-glycan processing
chr5	116406121	116406941	21.17	3.15E-03	2.51E-02	*Ccdc60*	-117546	ND	*Hspb8*	16333	protein binding
chr5	117781382	117782501	26.40	1.68E-03	2.29E-02	*Nos1*	-59908	negative regulation of apoptotic process	*Ksr2*	367942	positive regulation of MAPK cascade

Supplementary Table 10. H3K4me3 peaks disappeared in F1 - *(Continued)*

chr	start	end	FC	pval	adj pval	GREAT Gene 1	Distance to TSS	GO Term Gene 1	GREAT Gene 2	Distance to TSS	GO Term Gene 2
chr5	118085125	118086380	27.61	7.72E-03	2.99E-02	*Gm9754*	20393	ND	*Fbxw8*	69705	cell proliferation
chr5	130409246	130410171	4.03	1.15E-02	3.58E-02	*A330070K13Rik*	-25078	ND	*Wbscr17*	898209	O-glycan processing
chr6	37779318	37780337	4.07	9.43E-03	3.31E-02	*Trim24*	-90983	cellular response to estrogen stimulus	*Akr1d1*	249655	androgen metabolic process
chr6	70681729	70681806	4.21	1.01E-02	3.35E-02	*Rpia*	110464	ribose phosphate metabolic process			
chr6	88429792	88430835	35.89	1.20E-03	2.08E-02	*Eefsec*	16225	translation	*Dnajb8*	208046	chaperone-mediated protein folding
chr6	90881855	90882891	18.67	7.90E-03	3.00E-02	*Iqsec1*	-72250	actin cytoskeleton organization	*Nup210*	234456	protein transport
chr6	92588703	92589341	12.59	5.15E-03	2.61E-02	*Trh*	-344372	signal transduction	*Prickle2*	117123	blastocyst formation
chr6	101399379	101399949	30.17	5.00E-03	2.61E-02	*Pdzrn3*	-21767	protein ubiquitination			
chr6	110260859	110261938	27.95	4.28E-03	2.58E-02	*Grm7*	-384183	signal transduction			
chr6	111137885	111139409	5.08	1.11E-02	3.50E-02	*Grm7*	493065	signal transduction			
chr6	112722557	112723499	20.42	6.55E-03	2.77E-02	*Rad18*	-26358	DNA repair	*Srgap3*	224238	signal transduction
chr6	133988632	133988792	4.90	2.21E-02	4.80E-02	*Kap*	-135045	ND	*Etv6*	-46988	regulation of transcription, DNA-templated
chr6	139520516	139520737	29.69	1.90E-03	2.35E-02	*Pik3c2g*	-101261	phosphorylation	*Rergl*	-18718	ND
chr6	140289893	140290517	3.67	2.61E-02	5.19E-02	*Plekha5*	-133894	reproductive system development	*Capza3*	248680	spermatid development
chr6	146666392	146667549	27.77	1.01E-03	2.03E-02	*Stk38l*	-58024	protein phosphorylation	*Med21*	24424	stem cell population maintenance
chr7	11733882	11734412	7.86	6.75E-03	2.78E-02	*Vmn1r72*	-63628	sensory perception of chemical stimulus	*Vmn1r73*	-22110	sensory perception of chemical stimulus
chr7	11734620	11735213	6.16	6.12E-03	2.76E-02	*Vmn1r72*	-64398	sensory perception of chemical stimulus	*Vmn1r73*	-21340	sensory perception of chemical stimulus
chr7	12132490	12132565	3.68	5.52E-02	7.81E-02	*Vmn1r78*	-19936	sensory perception of chemical stimulus	*Vmn1r77*	91229	sensory perception of chemical stimulus
chr7	14484804	14485296	28.97	3.38E-03	2.51E-02	*2810007J24Rik*	-38464	ND	*Sult2a7*	7876	ND
chr7	26267386	26267482	6.54	1.39E-02	3.91E-02	*Vmn1r184*	603	sensory perception of chemical stimulus			
chr7	88266025	88266517	44.72	3.83E-03	2.55E-02	*Ctsc*	-11814	proteolysis	*Grm5*	663727	G-protein coupled glutamate receptor signaling pathway
chr7	99092583	99093481	7.19	4.80E-03	2.61E-02	*Uvrag*	48112	DNA repair	*Wnt11*	257901	Wnt signaling pathway
chr7	100011732	100012861	37.89	3.08E-03	2.51E-02	*Chrdl2*	5893	cell differentiation	*Pold3*	109268	nucleotide-excision repair, DNA gap filling
chr7	144177275	144178288	21.03	6.59E-03	2.78E-02	*Shank2*	-106603	regulation of AMPA receptor activity	*Gm498*	308603	ND
chr8	29407823	29407899	3462.02	9.89E-03	3.35E-02	*Unc5d*	-188523	apoptotic process			
chr8	62480679	62481284	16.62	2.72E-03	2.51E-02	*Spock3*	-470379	signal transduction	*Anxa10*	-357870	calcium ion binding
chr8	70341294	70342067	3.44	1.86E-02	4.43E-02	*Upf1*	11592	DNA repair	*Cers1*	25894	negative regulation of telomerase activity
chr8	72069021	72069265	12.26	6.74E-03	2.78E-02	*Olfr373*	-30619	G-protein coupled receptor signaling pathway	*Olfr372*	11483	G-protein coupled receptor signaling pathway
chr8	72069453	72069718	16.43	2.76E-03	2.51E-02	*Olfr373*	-30176	G-protein coupled receptor signaling pathway	*Olfr372*	11926	G-protein coupled receptor signaling pathway
chr8	72512065	72512420	10.15	5.90E-03	2.74E-02	*Slc35e1*	-19629	ND	*Med26*	36067	regulation of transcription, DNA-templated
chr8	72512505	72512608	31.34	4.66E-03	2.60E-02	*Slc35e1*	-19943	ND	*Med26*	35753	regulation of transcription, DNA-templated
chr8	128372468	128373675	9.93	8.54E-03	3.13E-02	*Itgb1*	-312582	G1/S transition of mitotic cell cycle	*Nrp1*	13999	VEGF-activated neuropilin signaling pathway
chr8	129264758	129264867	4.57	2.27E-02	4.88E-02	*2610044O15Rik8*	-42863	regulation of transcription, DNA-templated			

Supplementary Table 10. H3K4me3 peaks disappeared in F1 - *(Continued)*

chr	start	end	FC	pval	adj pval	GREAT Gene 1	Distance to TSS	GO Term Gene 1	GREAT Gene 2	Distance to TSS	GO Term Gene 2
chr9	22353575	22354374	12.12	5.90E-03	2.74E-02	*Zfp810*	-46337	DNA binding transcription factor activity	*Anln*	35231	cell cycle
chr9	22354512	22354741	13.59	5.21E-03	2.61E-02	*Zfp810*	-46989	DNA binding transcription factor activity	*Anln*	34579	cell cycle
chr9	22827534	22828778	10.88	3.31E-03	2.51E-02	*Bmper*	-394920	positive regulation of ERK1 and ERK2 cascade	*Bbs9*	352425	protein transport
chr9	35013372	35013614	12.62	6.00E-03	2.74E-02	*St3gal4*	103317	protein glycosylation	*Kirrel3*	527367	cell adhesion
chr9	36138572	36140039	19.63	2.06E-03	2.36E-02	*Gm7257*	-292578	ND	*Gm5916*	-10527	ND
chr9	62086290	62087114	33.32	4.41E-03	2.58E-02	*Glce*	-16096	heparin biosynthetic process	*Spesp1*	195477	multicellular organism development
chr9	62648513	62649538	7.04	9.19E-04	2.03E-02	*Coro2b*	-111982	actin cytoskeleton organization	*Itga11*	-28800	cell adhesion
chr9	63641219	63641388	65.02	5.53E-03	2.68E-02	*Aagab*	38649	protein transport	*Smad3*	116690	cell cycle arrest
chr9	63793816	63794624	4.18	8.39E-03	3.10E-02	*Smad3*	-36226	cell cycle arrest	*Smad6*	227839	negative regulation of BMP signaling pathway
chr9	64472981	64474053	14.64	3.57E-03	2.55E-02	*Megf11*	87891	homotypic cell-cell adhesion	*Rab11a*	264241	cell cycle
chr9	64687773	64688985	5.12	9.79E-03	3.33E-02	*Rab11a*	49379	cell cycle	*Megf11*	302753	homotypic cell-cell adhesion
chr9	65546602	65547008	28.17	4.63E-03	2.60E-02	*Ankdd1a*	-29806	signal transduction	*Plekho2*	33235	ND
chr9	82550105	82550890	15.01	2.33E-03	2.38E-02	*Irak1bp1*	-279308	I-kappaB kinase/NF-kappaB signaling	*Mei4*	686828	meiotic cell cycle
chr9	107693586	107693798	17.76	3.10E-03	2.51E-02	*Gnat1*	-14100	signal transduction	*Sema3f*	16783	negative chemotaxis
chr9	107693883	107694006	8.30	8.31E-03	3.09E-02	*Gnat1*	-14353	signal transduction	*Sema3f*	16530	negative chemotaxis
chr9	111291889	111297085	28.02	3.94E-02	6.41E-02	*Trank1*	-17252	ND	*Epm2aip1*	22642	regulation of transcription by RNA polymerase II
chrX	48274809	48275160	16.21	4.36E-03	2.58E-02	*Bcorl1*	-66373	sensory perception of chemical stimulus	*Utp14a*	18123	rRNA processing
chrX	48282074	48282392	36.36	3.28E-03	2.51E-02	*Bcorl1*	-59125	covalent chromatin modification	*Utp14a*	25371	rRNA processing
chrX	52898835	52898922	12.41	1.43E-03	2.24E-02	*Phf6*	-13387	regulation of transcription, DNA-templated	*Ccdc160*	107679	ND

We calculated the average values of FPKM for each peak for CD and control samples. Peaks with an average value of FPKM below the 5th centile (4.12) in CD samples are considered as disappeared peaks.

Supplementary Table 11. *De novo* H3K4me3 peaks identified in F3

chr	start	end	FC	pval	adj pval	GREAT Gene 1	Distance to TSS	GO Term Gene 1	GREAT Gene 2	Distance to TSS	GO Term Gene 2
chr1	31730565	31730853	4.35	1.83E-02	5.29E-02	*Khdrbs2*	-442097	regulation of transcription, DNA-templated	*Gm6489*	443764	ND
chr1	141140091	141141605	6.07	5.08E-03	3.98E-02	*Gm4845*	116310	ND	*Kcnt2*	894630	potassium ion transport
chr1	151581277	151582302	26.27	5.26E-04	1.92E-02	*Edem3*	-173584	mannose trimming involved in glycoprotein ERAD pathway	*Fam129a*	10417	positive regulation of translation
chr1	151582361	151583127	30.74	7.20E-04	1.92E-02	*Edem3*	-172630	mannose trimming involved in glycoprotein ERAD pathway	*Fam129a*	11371	positive regulation of translation
chr1	151676507	151677520	16.22	8.70E-04	1.92E-02	*Edem3*	-78360	mannose trimming involved in glycoprotein ERAD pathway	*Fam129a*	105641	positive regulation of translation
chr10	46881602	46882270	43.61	2.86E-04	1.53E-02						
chr11	61026202	61028653	4.12	2.95E-02	6.07E-02	*Kcnj12*	-38376	potassium ion import	*Map2k3*	95395	activation of MAPK activity
chr11	65712785	65713566	12.75	1.62E-02	5.09E-02	*Myocd*	-443187	negative regulation of cell proliferation	*Map2k4*	75121	regulation of mitotic cell cycle
chr11	80766304	80767093	23.54	1.73E-04	1.53E-02	*Myo1d*	13326	negative regulation of phosphatase activity	*Cdk5r1*	289653	negative regulation of transcription, DNA-templated
chr11	84911258	84911604	11.78	1.50E-03	2.69E-02	*Pigw*	-31236	GPI anchor biosynthetic process	*Znhit3*	4935	metal ion binding
chr12	19243246	19243423	120.66	6.27E-03	4.01E-02	*Gm5784*	-144474	ND	*Gm3944*	389463	ND
chr12	19249067	19249280	21.13	7.18E-03	4.01E-02	*Gm5784*	-138635	ND	*Gm3944*	395302	ND
chr12	70492610	70493509	4.17	2.20E-02	5.45E-02	*Frmd6*	-332454	regulation of actin filament-based process	*Tmx1*	39965	oxidation-reduction process
chr12	95563306	95563380	3.46	1.28E-02	4.85E-02	*Flrt2*	-128883	cell adhesion			
chr13	15788101	15788601	26.85	1.90E-03	3.12E-02	*Inhba*	-223500	negative regulation of cell cycle	*Gli3*	324371	chromatin binding
chr14	11235739	11236281	5.06	6.46E-03	4.01E-02	*Rpl21-ps4*	8458	structural constituent of ribosome		120716	
chr14	14194758	14198833	14.96	6.98E-04	1.92E-02	*Olfr31*	-131317	G-protein coupled receptor signaling pathway	*Olfr720*	-20679	G-protein coupled receptor signaling pathway
chr14	15111491	15111585	4.04	1.65E-02	5.09E-02	*Nek10*	290723	chromosome segregation	*Lrrc3b*	327449	ND
chr14	49239904	49240971	3.15	6.12E-02	8.72E-02	*1700011H14Rik*	4990	ND	*Naa30*	67840	N-terminal protein amino acid acetylation
chr14	54296318	54296945	7.49	6.88E-03	4.01E-02	*Oxa1l*	-64209	mitochondrial respiratory chain complex I assembly	*Olfr49*	-13707	G-protein coupled receptor signaling pathway
chr14	61090467	61091931	3.09	2.94E-02	6.07E-02	*Sacs*	-47258	low-density lipoprotein particle receptor binding	*Tnfrsf19*	-44344	positive regulation of I-kappaB kinase/ NF-kappaB signaling
chr14	90278351	90278623	5.07	1.44E-02	5.02E-02	*Gm10110*	-380021	ND			
chr14	90278772	90279434	4.20	1.63E-02	5.09E-02	*Gm10110*	-380637	ND			
chr14	93366967	93367537	17.48	2.80E-03	3.47E-02	*Pcdh9*	521480	ND			

Supplementary Table 11. *De novo* H3K4me3 peaks identified in F3 - *(Continued)*

chr	start	end	FC	pval	adj pval	GREAT Gene 1	Distance to TSS	GO Term Gene 1	GREAT Gene 2	Distance to TSS	GO Term Gene 2
chr14	93930740	93931270	5.27	2.70E-03	3.47E-02	*Pcdh9*	-42273	ND			
chr14	93933673	93934520	3.51	2.09E-02	5.39E-02	*Pcdh9*	-45365	ND			
chr14	94734207	94735051	23.92	6.00E-03	4.01E-02	*Pcdh9*	-845897	ND			
chr14	94735164	94736752	24.00	9.88E-04	2.03E-02	*Pcdh9*	-847226	ND			
chr14	105217977	105218581	2.62	3.07E-02	6.15E-02	*Rbm26*	-40956	mRNA processing	*Ndfip2*	-40294	positive regulation of I-kappaB kinase/ NF-kappaB signaling
chr15	77708418	77709375	8.84	5.15E-03	3.98E-02	*Apol9b*	-20224	response to stimulus	*Apol7e*	10008	response to stimulus
chr15	97625462	97626789	3.41	4.26E-02	7.05E-02	*Amigo2*	-378839	cell-cell adhesion	*Rpap3*	79696	protein folding
chr16	45172507	45173373	15.70	7.66E-03	4.04E-02	*Btla*	-51397	adaptive immune response	*Atg3*	14155	protein ubiquitination
chr16	45173426	45174784	4.39	5.88E-03	4.01E-02	*Btla*	-50232	adaptive immune response	*Atg3*	15320	protein ubiquitination
chr16	57113662	57115239	3.08	5.05E-02	7.60E-02	*2310005G13Rik*	-43105	ND	*Tomm70a*	-7263	positive regulation of protein import
chr16	64599877	64601693	13.49	2.89E-03	3.48E-02	*Csnka2ip*	-121637	protein binding	*4930453N24Rik*	170376	positive regulation of apoptotic process
chr16	68626118	68626272	3.15	1.56E-02	5.09E-02						
chr17	35310722	35310799	3.21	2.73E-02	5.93E-02	*H2-Q1*	-9797	antigen processing and presentation of peptide antigen via MHC class I	*H2-D1*	48031	antigen processing and presentation of peptide antigen via MHC class I
chr17	74951518	74952318	8.48	8.10E-03	4.04E-02	*Ltbp1*	-53650	transforming growth factor beta binding	*Ttc27*	234168	ND
chr17	83448390	83449040	3.57	1.33E-02	4.90E-02	*Cox7a2l*	65618	regulation of oxidative phosphorylation	*Eml4*	97784	microtubule cytoskeleton organization
chr18	7319445	7319553	2.90	2.21E-02	5.45E-02	*Armc4*	-21598	cell projection organization	*Mpp7*	307364	protein localization to adherens junction
chr18	16463291	16465963	8.33	5.25E-04	1.92E-02	*Cdh2*	344619	cell-cell adhesion	*Gm10036*	631833	
chr18	17326233	17327919	6.35	1.53E-03	2.69E-02	*Cdh2*	-517830	cell-cell adhesion			
chr18	68471476	68475249	14.61	2.23E-02	5.45E-02	*Mc2r*	-44112	melanocortin receptor activity	*4930546C10Rik*	476778	ND
chr19	15158159	15158655	2.58	3.28E-02	6.30E-02	*Tle4*	-560424	Wnt signaling pathway	*Psat1*	766652	L-serine biosynthetic process
chr19	18918943	18921991	7.50	6.76E-02	9.37E-02	*Rorb*	80650	cell differentiation	*Trpm6*	170484	calcium ion transport
chr19	21804380	21804838	14.57	5.30E-03	4.01E-02	*Trpm3*	-334510	cation transport	*Tmem2*	26267	angiogenesis
chr19	31126491	31127533	14.46	7.30E-04	1.92E-02	*Cstf2t*	44171	mRNA processing	*Prkg1*	537358	phosphorylation
chr2	20905434	20905639	10.79	6.57E-03	4.01E-02	*Arhgap21*	62184	positive regulation of GTPase activity	*Etl4*	615624	multicellular organism development
chr2	40127781	40127905	4.22	4.26E-03	3.71E-02	*Ppp6c*	-901392	cell cycle			
chr2	71820038	71820196	29.24	7.68E-04	1.92E-02	*Pdk1*	-53107	cell proliferation	*Itga6*	33139	cell-cell adhesion
chr2	71825582	71825661	12.92	1.13E-03	2.23E-02	*Pdk1*	-47602	cell proliferation	*Itga6*	38644	cell-cell adhesion
chr2	71838329	71838434	6.52	2.78E-03	3.47E-02	*Pdk1*	-34842	cell proliferation	*Itga6*	51404	cell-cell adhesion
chr2	71838579	71838679	14.41	6.27E-04	1.92E-02	*Pdk1*	-34595	cell proliferation	*Itga6*	51651	cell-cell adhesion
chr2	108420569	108421859	4.31	4.77E-02	7.36E-02	*Mettl15*	857076	rRNA methylation			
chr2	148341483	148343294	18.24	5.15E-03	3.98E-02	*Foxa2*	-295420	Notch signaling pathway	*Sstr4*	-53009	negative regulation of cell proliferation
chr2	149821764	149821857	18.43	2.23E-03	3.37E-02	*Syndig1*	-9032	intracellular protein transport	*Cst10*	416562	chondrocyte differentiation

Supplementary Table 11. *De novo* H3K4me3 peaks identified in F3 - *(Continued)*

chr	start	end	FC	pval	adj pval	GREAT Gene 1	Distance to TSS	GO Term Gene 1	GREAT Gene 2	Distance to TSS	GO Term Gene 2
chr2	156361778	156363318	8.17	4.12E-02	6.97E-02	*Epb4.1l1*	-58361	actomyosin structure organization	*Scand1*	-49844	transcription coactivator activity
chr2	156783822	156784583	3.54	1.95E-02	5.29E-02	*Tgif2*	-55874	regulation of transcription, DNA-templated	*Myl9*	8783	calcium ion binding
chr2	156948350	156948814	3.77	2.12E-02	5.39E-02	*Sla2*	-61504	regulation of cell proliferation	*Ndrg3*	43461	signal transduction
chr3	6798644	6800664	3.94	1.38E-02	4.99E-02	*Pkia*	-566950	regulation of G2/M transition of mitotic cell cycle	*1700008P02Rik*	-179211	ND
chr3	20374205	20374685	10.87	7.75E-03	4.04E-02	*Agtr1b*	-7268	G-protein coupled receptor signaling pathway			
chr3	28891636	28892123	22.38	4.17E-03	3.71E-02	*Gm1527*	-737	GTPase activator activity			
chr3	30437753	30438397	12.40	1.43E-02	5.01E-02	*Gm10258*	-168128	ND	*Actrt3*	161864	protein binding
chr3	73300899	73301698	8.93	7.07E-03	4.01E-02	*Slitrk3*	-244356	positive regulation of synapse assembly	*Bche*	407116	negative regulation of cell proliferation
chr3	74324218	74324817	9.36	5.90E-03	4.01E-02	*Bche*	-616103	negative regulation of cell proliferation	*Zbbx*	819254	zinc ion binding
chr3	78307280	78307931	3.90	1.14E-02	4.67E-02	*Gm5277*	584845	ND			
chr3	96983949	96985415	3.84	7.09E-03	4.01E-02	*Gja8*	-58662	cell-cell signaling	*Gja5*	-47734	cell communication
chr3	97351378	97354167	31.44	1.99E-02	5.29E-02	*Bcl9*	-125409	Wnt signaling pathway	*Olfr1402*	58406	G-protein coupled receptor signaling pathway
chr4	58639211	58639554	23.11	4.25E-03	3.71E-02	*Lpar1*	-86072	positive regulation of apoptotic process	*Olfr267*	146339	G-protein coupled receptor signaling pathway
chr4	58639651	58640541	20.90	6.99E-03	4.01E-02	*Lpar1*	-86785	positive regulation of apoptotic process	*Olfr267*	145626	G-protein coupled receptor signaling pathway
chr4	115719168	115719920	7.37	8.71E-03	4.10E-02	*Cyp4b1*	-71835	oxidation-reduction process	*Efcab14*	-18529	metal ion binding
chr4	146503989	146504256	14.20	7.22E-03	4.01E-02	*Gm13248*	-106838	ND	*Gm13247*	2096	regulation of transcription, DNA-templated
chr4	147219346	147219660	8.31	1.24E-02	4.81E-02	*Gm13151*	-86171	regulation of transcription, DNA-templated	*Gm13139*	87465	regulation of transcription, DNA-templated
chr5	3731226	3731761	11.91	7.00E-03	4.01E-02	*Ankib1*	71615	protein ubiquitination	*4930511M11Rik*	74490	ND
chr5	3732341	3733290	10.01	3.44E-03	3.60E-02	*Ankib1*	70293	protein ubiquitination	*4930511M11Rik*	75812	ND
chr5	109906670	109907483	3.72	8.02E-03	4.04E-02	*4930522L14Rik*	-155480	regulation of transcription, DNA-templated	*Gm15446*	-16323	regulation of transcription, DNA-templated
chr5	110508925	110509389	21.21	3.33E-03	3.60E-02	*Fbrsl1*	-60654	ND	*Galnt9*	-35188	O-glycan processing
chr7	11504758	11504857	32.74	9.18E-03	4.22E-02	*Zscan4f*	106870	regulation of transcription, DNA-templated	*Vmn1r72*	165711	sensory perception of chemical stimulus
chr7	25558673	25560789	8.00	7.97E-03	4.04E-02	*Ceacam2*	-19727	energy homeostasis	*Gm7092*	56161	ND
chr7	38190694	38190819	13.24	7.97E-04	1.92E-02	*1600014C10Rik*	7540	apoptotic process	*Plekhf1*	37237	apoptotic process
chr7	38570616	38570689	3.45	1.11E-02	4.61E-02	*Gm5591*	-42460	ND	*Gm5114*	842507	ND
chr7	38722590	38722927	5.67	8.99E-03	4.17E-02	*Gm5591*	-194566	ND	*Gm5114*	690401	ND

Supplementary Table 11. *De novo* H3K4me3 peaks identified in F3 - *(Continued)*

chr	start	end	FC	pval	adj pval	GREAT Gene 1	Distance to TSS	GO Term Gene 1	GREAT Gene 2	Distance to TSS	GO Term Gene 2
chr7	38723488	38723604	2.94	1.90E-02	5.29E-02	*Gm5591*	-195353	ND	*Gm5114*	689614	ND
chr7	39181870	39181982	3.52	1.89E-02	5.29E-02	*Gm5591*	-653733	ND	*Gm5114*	231234	ND
chr7	39971677	39971931	3.22	4.74E-02	7.36E-02	*Vstm2b*	-927474	ND	*Gm2058*	382873	ND
chr7	39971996	39972859	3.42	3.81E-02	6.71E-02	*Vstm2b*	-926850	ND	*Gm2058*	383497	ND
chr7	40199180	40200068	4.24	9.95E-03	4.40E-02	*Vstm2b*	-699654	ND	*Gm2058*	610693	ND
chr7	41040849	41041386	10.73	2.08E-04	1.53E-02	*Gm2128*	-114357	ND	*Gm4884*	8399	ND
chr7	41042701	41042779	12.76	7.52E-03	4.04E-02	*Gm2128*	-112735	ND	*Gm4884*	10021	ND
chr7	41287112	41287345	5.23	1.81E-02	5.27E-02	*Gm5592*	2902	ND	*AI987944*	106031	regulation of transcription, DNA-templated
chr7	41298704	41299043	3.47	4.65E-02	7.32E-02	*Gm5592*	14547	ND	*AI987944*	94386	regulation of transcription, DNA-templated
chr7	41299096	41299500	4.22	3.11E-02	6.15E-02	*Gm5592*	14971	ND	*AI987944*	93962	regulation of transcription, DNA-templated
chr7	119352302	119353436	19.16	4.73E-03	3.86E-02	*Gpr139*	-168495	G-protein coupled receptor signaling pathway	*Gp2*	106403	antigen transcytosis by M cells in mucosal-associated lymphoid tissue
chr8	28212497	28212839	3.07	3.77E-02	6.71E-02	*Poteg*	764998	ND			
chr8	33208220	33209047	3.11	3.56E-02	6.57E-02	*Wrn*	176893	DNA repair			
chr8	34066324	34066548	4.34	1.66E-02	5.09E-02	*Rbpms*	-136631	mRNA splicing, via spliceosome	*Dctn6*	42360	lipid biosynthetic process
chr8	34246806	34247721	4.15	1.98E-02	5.29E-02	*Gm4889*	-80646	cytoplasmic translation	*Tmem66*	92701	calcium ion transport
chr8	52669369	52669946	3.20	2.22E-02	5.45E-02	*Aga*	-842044	proteolysis			
chr9	14101146	14101257	3.52	1.08E-02	4.60E-02	*Sesn3*	-175099	TORC2 signaling	*Fam76b*	273486	ND
chr9	97509355	97510390	20.14	2.21E-03	3.37E-02	*Trim42*	-139915	zinc ion binding	*Clstn2*	523308	cell adhesion
chr9	98645121	98645603	15.54	5.29E-03	4.01E-02	*Prr23a*	-197225	ND	*Mrps22*	-43683	structural constituent of ribosome
chr9	100817203	100818385	11.17	8.98E-03	4.17E-02	*Stag1*	174346	cell cycle	*Pccb*	217104	fatty acid catabolic process
chr9	101269493	101271677	12.52	2.22E-03	3.37E-02	*9630041A04Rik*	-667896	ND	*Ppp2r3a*	-18753	negative regulation of canonical Wnt signaling pathway
chr9	102572515	102573008	7.65	7.54E-03	4.04E-02	*Cep63*	53362	cell cycle	*Ky*	66624	proteolysis
chr9	102997717	103000764	19.27	6.98E-04	1.92E-02	*Slco2a1*	-9248	sodium-independent organic anion transport	*Ryk*	164324	Wnt signaling pathway
chr9	103000842	103001927	9.26	2.93E-03	3.48E-02	*Slco2a1*	-7104	sodium-independent organic anion transport	*Ryk*	166468	Wnt signaling pathway
chrX	67566293	67567940	23.53	3.25E-03	3.60E-02	*4933436I01Rik*	354333	ND	*Slitrk2*	917799	positive regulation of synapse assembly
chrX	75682976	75683051	38.36	2.68E-03	3.47E-02	*Rab39b*	-104783	Rab protein signal transduction	*Pls3*	191560	bone development
chrX	75683456	75683625	88.00	3.82E-03	3.65E-02	*Rab39b*	-105310	Rab protein signal transduction	*Pls3*	191033	bone development
chrX	75683684	75683778	24.00	3.98E-03	3.65E-02	*Rab39b*	-105500	Rab protein signal transduction	*Pls3*	190843	bone development

Supplementary Table 11. ***De novo*** **H3K4me3 peaks identified in F3 -** ***(Continued)***

chr	start	end	FC	pval	adj pval	GREAT Gene 1	Distance to TSS	GO Term Gene 1	GREAT Gene 2	Distance to TSS	GO Term Gene 2
chrX	124142261	124142421	25.32	6.98E-03	4.01E-02	*Vmn2r121*	-6431	ND			
chrX	124142490	124142644	9.18	1.02E-02	4.43E-02	*Vmn2r121*	-6657	ND			

We calculated the average values of FPKM for each peak for CD and control samples. Peaks with an average value of FPKM below the 5th centile (6.82) in control samples are considered as disappeared peaks.

Supplementary Table 12. H3K4me3 peaks disappeared in F3

chr	start	end	FC	pval	adj pval	GREAT Gene 1	Distance to TSS	GO Term Gene 1	GREAT Gene 2	Distance to TSS	GO Term Gene 2
chr1	19258172	19259650	31.65	1.17E-02	6.08E-02	*Tfap2b*	49997	calcium ion homeostasis			
chr1	33637464	33638413	70.79	9.34E-04	2.79E-02	*Prim2*	31847	DNA replication			
chr1	146724807	146729233	7.03	3.07E-03	4.23E-02	*Fam5c*	231391	cell cycle	*Gm9931*	*554922*	ND
chr1	165188397	165189701	19.65	8.58E-03	5.91E-02	*Tbx19*	-28276	regulation of cell proliferation	*Sft2d2*	*5384*	vesicle-mediated transport
chr10	23726547	23726645	39.96	1.92E-03	3.62E-02	*Eya4*	-376701	DNA repair	*Rps12*	*60599*	structural constituent of ribosome
chr10	105314917	105315854	50.24	1.27E-02	6.28E-02	*Tmtc2*	259049	calcium ion homeostasis			
chr11	27135093	27135282	3.88	2.98E-02	7.73E-02	*Vrk2*	-541268	Wnt signaling pathway			
chr11	61714897	61715184	3.69	3.05E-02	7.76E-02	*Slc5a10*	5758	transmembrane transport	*Fam83g*	*30622*	BMP signaling pathway
chr11	64780645	64782566	10.80	8.31E-03	5.78E-02	*Elac2*	-197432	tRNA processing	*Hs3st3a1*	*346274*	transferase activity
chr12	18320868	18321053	5.60	1.17E-02	6.08E-02	*5730507C01Rik*	-193777	regulation of transcription, DNA-templated	*B430203G13Rik*	*396667*	ND
chr12	18363629	18363984	3.63	4.04E-02	8.24E-02	*5730507C01Rik*	-150931	regulation of transcription, DNA-templated	*B430203G13Rik*	*439513*	ND
chr12	76887759	76887864	54.06	8.91E-04	2.78E-02	*Fntb*	50398	positive regulation of cell cycle	*Max*	*74436*	regulation of transcription, DNA-templated
chr12	108164332	108164692	29.45	2.24E-02	7.25E-02	*Bcl11b*	-160910	T cell differentiation in thymus	*Setd3*	*14802*	histone H3-K36 methylation
chr12	110568237	110568909	4.42	2.86E-02	7.67E-02	*Dync1h1*	-32879	cell cycle	*Ppp2r5c*	*82834*	signal transduction
chr14	46802511	46803860	3.69	2.55E-02	7.45E-02	*Cnih*	-14775	vesicle-mediated transport	*Gmfb*	*19056*	negative regulation of Arp2/3 complex-mediated actin nucleation
chr14	51612192	51612320	4.68	3.45E-02	7.85E-02	*Gm4181*	23410	ND	*Gm5622*	*59466*	ND
chr14	51677420	51678184	92.85	1.47E-02	6.53E-02	*Gm4181*	-42136	ND	*Gm5800*	*39381*	ND
chr14	123347506	123348337	7.97	2.79E-02	7.67E-02	*Tmtc4*	-364639	ND	*Nalcn*	*279222*	calcium ion transport
chr15	30030181	30030408	4.60	2.30E-02	7.36E-02	*Ctnnd2*	-142298	cell-cell adhesion			
chr15	60794567	60795986	6.75	1.37E-03	3.24E-02	*4933412E24Rik*	-778659	ND	*Fam84b*	*29803*	ND
chr15	88780809	88783182	6.02	1.16E-02	6.08E-02	*Zbed4*	30336	regulation of transcription by RNA polymerase II	*Alg12*	*37322*	protein N-linked glycosylation
chr16	58932237	58933130	3.36	2.56E-02	7.45E-02	*Olfr181*	-4040	G-protein coupled receptor signaling pathway			
chr16	78056689	78057221	8.03	2.86E-02	7.67E-02	*Gm11146*	-454861	ND	*Cxadr*	*-244541*	cell-cell adhesion
chr17	36189318	36189551	3.16	5.09E-02	8.71E-02	*H2-T3*	852	antigen processing and presentation of peptide antigen via MHC class I			

Supplementary Table 12. H3K4me3 peaks disappeared in F3 - *(Continued)*

chr	start	end	FC	pval	adj pval	GREAT Gene 1	Distance to TSS	GO Term Gene 1	GREAT Gene 2	Distance to TSS	GO Term Gene 2
chr17	58693460	58694004	19.13	1.16E-02	6.08E-02	*2610034M16Rik*	297643	ND	*Cntnap5c*	*924162*	cell adhesion
chr18	12316939	12318856	4.05	1.91E-02	6.78E-02	*Lama3*	-16126	cell-cell adhesion	*Ankrd29*	*-12187*	ND
chr18	75046471	75047672	5.71	1.62E-02	6.53E-02	*Smad7*	-320457	positive regulation of cell-cell adhesion	*Dym*	*28300*	bone development
chr2	104699463	104700358	8.51	1.04E-02	6.08E-02	*Tcp11l1*	12245	ND	*Cstf3*	*109388*	mRNA processing
chr2	125522003	125522229	30.43	2.36E-02	7.37E-02	*Fbn1*	-14123	cell adhesion mediated by integrin	*Cep152*	*102997*	centriole replication
chr2	125530309	125531388	33.92	2.77E-02	7.67E-02	*Fbn1*	-22856	cell adhesion mediated by integrin	*Cep152*	*94264*	centriole replication
chr2	132945899	132946029	3.57	2.97E-02	7.73E-02	*Fermt1*	-58	negative regulation of canonical Wnt signaling pathway			
chr2	145640371	145643771	5.61	1.03E-02	6.08E-02	*Rin2*	-144045	endocytosis	*Slc24a3*	*397099*	ion transport
chr2	155402042	155403697	18.83	1.35E-02	6.41E-02	*Trp53inp2*	21056	regulation of transcription, DNA-templated	*Ncoa6*	*70936*	cellular response to DNA damage stimulus
chr2	158698495	158699301	14.24	1.26E-02	6.28E-02	*Fam83d*	-69195	cell cycle	*Ppp1r16b*	*33500*	regulation of protein dephosphorylation
chr4	60038139	60038230	4.22	1.59E-02	6.53E-02	*Mup7*	32290	ND	*Mup6*	*34747*	ND
chr5	111892907	111894519	4.51	1.71E-02	6.53E-02	*C130026L21Rik*	312291	ND	*Cryba4*	*358805*	visual perception
chr5	111979914	111980834	14.29	1.56E-02	6.53E-02	*Cryba4*	272144	visual perception	*C130026L21Rik*	*398952*	ND
chr6	8646617	8647856	16.08	1.31E-02	6.29E-02	*Ica1*	131247	neurotransmitter transport	*Glcci1*	*137637*	protein binding
chr6	8647940	8648106	10.48	1.14E-02	6.08E-02	*Ica1*	130461	neurotransmitter transport	*Glcci1*	*138423*	protein binding
chr6	8648188	8648710	25.79	7.17E-03	5.38E-02	*Ica1*	130035	neurotransmitter transport	*Glcci1*	*138849*	protein binding
chr6	147173638	147173890	30.58	4.38E-02	8.38E-02	*Klhl42*	82385	cell cycle	*Pthlh*	*90403*	positive regulation of cell proliferation
chr6	147173989	147174111	27.49	2.95E-02	7.73E-02	*Klhl42*	82671	cell cycle	*Pthlh*	*90117*	positive regulation of cell proliferation
chr7	37446498	37447571	45.66	9.48E-03	6.08E-02	*Zfp536*	322927	multicellular organism development	*Tshz3*	*748917*	regulation of transcription, DNA-templated
chr7	37447713	37449778	27.66	4.43E-03	4.87E-02	*Zfp536*	321216	multicellular organism development	*Tshz3*	*750628*	regulation of transcription, DNA-templated
chr7	38293615	38294026	30.85	1.24E-02	6.28E-02	*Pop4*	-22473	mRNA cleavage	*Gm5591*	*234372*	ND
chr7	38412409	38412830	11.56	1.27E-02	6.28E-02	*Pop4*	-141272	mRNA cleavage	*Gm5591*	*115573*	ND
chr7	38571726	38571917	4.50	1.64E-02	6.53E-02	*Gm5591*	-43629	ND	*Gm5114*	*841338*	ND
chr7	38592811	38593803	50.01	1.26E-02	6.28E-02	*Gm5591*	-65114	ND	*Gm5114*	*819853*	ND
chr7	38828227	38828730	3.65	1.81E-02	6.62E-02	*Gm5591*	-300286	ND	*Gm5114*	*584681*	ND
chr7	42440068	42440555	23.65	1.52E-02	6.53E-02	*4933421I07Rik*	7808	ND	*Vmn2r61*	*180259*	G-protein coupled receptor activity
chr7	60733168	60734163	23.11	2.13E-02	7.19E-02	*Snrpn*	-593447	mRNA splicing, via spliceosome			

Supplementary Table 12. H3K4me3 peaks disappeared in F3 - *(Continued)*

chr	start	end	FC	pval	adj pval	GREAT Gene 1	Distance to TSS	GO Term Gene 1	GREAT Gene 2	Distance to TSS	GO Term Gene 2
chr7	138876509	138876675	19.98	2.32E-02	7.37E-02	*Ppp2r2d*	30233	cell cycle	*Bnip3*	*32914*	apoptotic process
chr7	138882241	138882729	9.98	2.82E-03	4.17E-02	*Bnip3*	27021	apoptotic process	*Ppp2r2d*	*36126*	cell cycle
chr8	34522779	34523829	5.44	1.63E-02	6.53E-02	*Dusp4*	-283993	protein dephosphorylation	*Gm4889*	*195394*	cytoplasmic translation
chr8	78954239	78954403	3.24	3.69E-02	8.05E-02	*Lsm6*	-133181	mRNA processing	*Zfp827*	*-74116*	regulation of transcription, DNA-templated
chr8	94504120	94505565	27.60	1.04E-03	2.86E-02	*Cpne2*	-28147	cellular response to calcium ion	*Nlrc5*	*32080*	negative regulation of NF-kappaB transcription factor activity
chr8	95300343	95300450	6.81	1.72E-02	6.53E-02	*Cngb1*	-16216	potassium ion transmembrane transport	*Cngb1*	*6188*	potassium ion transmembrane transport
chr8	95300552	95300825	3.78	2.55E-02	7.45E-02	*Cngb1*	-16508	potassium ion transmembrane transport	*Cngb1*	*5896*	potassium ion transmembrane transport
chr8	95437283	95440992	37.77	1.46E-02	6.53E-02	*Gtl3*	-4269	positive regulation of cell motility			
chr9	28830934	28831190	112.99	1.10E-02	6.08E-02						
chr9	38893762	38894398	2.79	4.30E-02	8.38E-02	*Olfr930*	-36093	G-protein coupled receptor signaling pathway	*Olfr926*	*16954*	G-protein coupled receptor signaling pathway
chr9	40607649	40608328	7.73	4.75E-02	8.41E-02	*Gramd1b*	-152319	ND	*Gm21915*	*-62624*	ND
chr9	97271344	97271820	3.26	3.60E-02	7.98E-02	*Slc25a36*	-160425	transmembrane transport	*Trim42*	*98376*	metal ion binding
chr9	98277076	98280162	5.23	1.42E-02	6.53E-02	*2610303G11Rik*	-91467	ND	*Nmnat3*	*-17964*	NAD biosynthetic process
chr9	101631101	101632033	25.74	7.97E-03	5.65E-02	*Ppp2r3a*	-379735	negative regulation of canonical Wnt signaling pathway	*9630041A04Rik*	*-306914*	ND
chrX	158494651	158495003	12.81	9.19E-03	6.08E-02	*Rps6ka3*	-761031	cell cycle	*Cnksr2*	*-451533*	Ras protein signal transduction
chrX	170674243	170674544	7.12	2.28E-03	3.68E-02	*Asmt*	1750	negative regulation of male gonad development			
chrX	170675710	170675892	7.78	1.52E-03	3.24E-02	*Asmt*	3157	negative regulation of male gonad development			

We calculated the average values of FPKM for each peak for CD and control samples. Peaks with an average value of FPKM below the 5th centile (6.82) in CD samples are considered as disappeared peaks.

Supplementary Table 13. Common peaks in F1 and F3

chr	start	stop	GREAT gene 1	Distance to TSS	GO Term Gene 1	GREAT gene 2	Distance to TSS	GO Term Gene 2
chr10	95317213	95318626	*Plxnc1*	-373342	negative regulation of cell adhesion	*Cradd*	6177	DNA damage response, signal transduction by p53 class mediator resulting in cell cycle arrest
chr10	105314917	105315854	*Tmtc2*	259049	calcium ion homeostasis			
chr11	64017259	64017399	*Hs3st3b1*	-95039	protein sulfation	*Cox10*	62137	cytochrome complex assembly
chr11	64780645	64782566	*Elac2*	-197432	tRNA processing	*Hs3st3a1*	346274	sulfotransfer ase activity
chr11	80766304	80767093	*Myo1d*	13326	negative regulation of phosphatase activity	*Cdk5r1*	289653	negative regulation of transcription, DNA-templated
chr14	44500044	44501513	*BC061237*	657	ND			
chr14	44518245	44519509	*Gm8247*	-64276	ND	*BC061237*	18755	ND
chr14	49238231	49239714	*1700011H14 Rik*	6455	ND	*Naa30*	66375	N-terminal protein amino acid acetylation
chr14	49239904	49240971	*1700011H14 Rik*	4990	ND	*Naa30*	67840	N-terminal protein amino acid acetylation
chr14	51007629	51008901	*Rnase10*	336	cell adhesion			
chr14	51344022	51346048	*Gm7247*	-16721	ND	*Gm21718*	32187	ND
chr14	51381823	51383470	*Vmn2r88*	-28354	G-protein coupled receptor activity	*Gm7247*	20891	ND
chr14	51592314	51592575	*Gm5622*	39655	ND	*Gm4181*	43221	ND
chr14	51611321	51611473	*Gm4181*	24269	ND	*Gm5622*	58607	ND
chr14	51612192	51612320	*Gm4181*	23410	ND	*Gm5622*	59466	ND
chr14	51633405	51633738	*Gm4181*	2094	ND	*Gm5622*	80782	ND
chr14	51677420	51678184	*Gm4181*	-42136	ND	*Gm5800*	39381	ND

Supplementary Table 13. Common peaks in F1 and F3 - *(Continued)*

chr	start	stop	GREAT gene 1	Distance to TSS	GO Term Gene 1	GREAT gene 2	Distance to TSS	GO Term Gene 2
chr14	105217977	105218581	*Rbm26*	-40956	mRNA processing	*Ndfip2*	-40294	positive regulation of I-kappaB kinase/NF-kappaB signaling
chr16	45173426	45174784	*Btla*	-50232	adaptive immune response	*Atg3*	15320	autophagy
chr16	69866935	69869269	*Speer2*	-4358	ND			
chr17	33874501	33875283	*Kifc1*	15769	mitotic spindle assembly	*Cd320*	31801	positive regulation of B cell proliferation
chr18	16463291	16465963	*Cdh2*	344619	cell-cell adhesion	*Gm10036*	631833	ribosomal large subunit assembly
chr18	18483607	18484229						
chr18	18567379	18568631						
chr19	21804380	21804838	*Trpm3*	-334510	cation transport	*Tmem2*	26267	angiogenesis
chr19	31126491	31127533	*Cstf2t*	44171	mRNA processing	*Prkg1*	537358	phosphorylation
chr19	43267086	43268986	*Hps1*	-488058	secretion of lysosomal enzymes	*Hpse2*	120275	positive regulation of cell proliferation
chr2	71838329	71838434	*Pdk1*	-34842	cell proliferation	*Itga6*	51404	cell-cell adhesion
chr2	125530309	125531388	*Fbn1*	-22856	cell adhesion mediated by integrin	*Cep152*	94264	centriole replication
chr2	146281648	146283254	*Insm1*	60530	cell cycle	*Ralgapa2*	229553	Ral protein signal transduction
chr2	155782340	155785361	*Mmp24*	8509	cell adhesion	*Eif6*	43074	gene silencing by miRNA
chr2	156003073	156004413	*Ergic3*	-4302	vesicle-mediated transport	*6430550D23Rik*	684	oligosaccharide metabolic process
chr2	156682444	156684390	*Myl9*	-92003	calcium ion binding	*Dlgap4*	69712	signaling
chr2	156780034	156781622	*Tgif2*	-59249	regulation of transcription, DNA-templated	*Myl9*	5408	calcium ion binding

Supplementary Table 13. Common peaks in F1 and F3 - *(Continued)*

chr	start	stop	GREAT gene 1	Distance to TSS	GO Term Gene 1	GREAT gene 2	Distance to TSS	GO Term Gene 2
chr2	156809770	156810548	*Tgif2*	-29918	regulation of transcription, DNA-templated	*Myl9*	34739	calcium ion binding
chr4	147219346	147219660	*Gm13151*	-86171	regulation of transcription, DNA-templated	*Gm13139*	87465	regulation of transcription, DNA-templated
chr5	3731226	3731761	*Ankib1*	71615	protein ubiquitination	*4930511M11 Rik*	74490	ND
chr5	3732341	3733290	*Ankib1*	70293	protein ubiquitination	*4930511M11 Rik*	75812	ND
chr5	61576353	61577627	*G6pd2*	-231853	carbohydrate metabolic process			
chr5	101663345	101668051	*Nkx6-1*	-472	cell proliferation			
chr5	109906670	109907483	*4930522L14R ik*	-155480	regulation of transcription, DNA-templated	*Gm15446*	-16323	regulation of transcription, DNA-templated
chr5	110508925	110509389	*Fbrsl1*	-60654	ND	*Galnt9*	-35188	O-glycan processing
chr5	111979914	111980834	*Cryba4*	272144	visual perception	*C130026L21 Rik*	398952	ND
chr5	112119182	112122665	*Cryba4*	131594	visual perception	*C130026L21 Rik*	539502	ND
chr7	11970076	11972567	*Vmn1r77*	-69977	sensory perception of chemical stimulus	*Vmn1r76*	-40037	sensory perception of chemical stimulus
chr7	31161796	31162945	*Gramd1a*	-11321	ND	*Scgb1b2*	129445	steroid binding
chr7	39177711	39181069	*Gm5591*	-651197	ND	*Gm5114*	233770	ND
chr7	41040160	41040760	*Gm2128*	-115015	ND	*Gm4884*	7741	ND
chr7	41283049	41287060	*Gm5592*	728	ND			
chr7	138876509	138876675	*Ppp2r2d*	30233	cell cycle	*Bnip3*	32914	apoptotic process
chr7	138882241	138882729	*Bnip3*	27021	apoptotic process	*Ppp2r2d*	36126	cell cycle
chr8	34119934	34120679	*Mboat4*	5277	peptidyl-serine octanoylation	*Leprotl1*	26726	negative regulation of protein localization to cell surface

Supplementary Table 13. Common peaks in F1 and F3 - *(Continued)*

chr	start	stop	GREAT gene 1	Distance to TSS	GO Term Gene 1	GREAT gene 2	Distance to TSS	GO Term Gene 2
chr8	95437283	95440992	*Gtl3*	-4269	positive regulation of cell motility			
chr8	110742809	110742917	*Mtss1l*	21387	activation of GTPase activity	*Il34*	63040	innate immune response

Supplementary Table 14. Predicted ESR1 binding sites in F1

	Peak		ESR1						
chr	start	stop	start	stop	strand	score	p-value	q-value	matched_sequence
chr1	16161821	16163027	733	750	-	10.9727	5.30E-05	0.32	AAGTGAGACTGTCCTTAG
chr1	59448367	59449923	1352	1369	+	11.4091	4.05E-05	0.301	GGGGCAGGGAGGCCACGT
chr1	88499299	88499864	491	508	+	10.4	7.46E-05	0.329	GGGGCAGTTTCCCCTATG
chr1	131678597	131680572	120	137	-	10.1273	8.75E-05	0.329	AGGTCAGCATACTCTAGA
chr1	155663186	155663480	210	227	-	12.9818	1.46E-05	0.237	TGGCCACCCTGTCCTGGG
chr1	155663186	155663480	213	230	+	12.0455	2.71E-05	0.275	AGGACAGGGTGGCCAAGG
chr1	180403308	180403879	491	508	+	15.3	2.70E-06	0.154	ACGTCAGGATGACCCTGG
chr1	180403308	180403879	488	505	-	11.8182	3.13E-05	0.283	GGGTCATCCTGACGTCTA
chr1	180403308	180403879	494	511	-	10.0364	9.22E-05	0.329	AGGCCAGGGTCATCCTGA
chr1	182983087	182983856	314	331	+	11.1182	4.85E-05	0.316	AGGTCAGGGTAATCACCA
chr10	60618744	60619827	932	949	-	11.9818	2.82E-05	0.279	GAGGCAGGGTGGCCAGCA
chr10	70949329	70950468	489	506	+	9.96364	9.62E-05	0.33	TTGTCACCGTCACCGGGC
chr10	78203767	78205578	335	352	+	12.2818	2.32E-05	0.27	CGGGCAGGCTGTTCTGAG
chr10	88937502	88937674	70	87	+	10.0909	8.94E-05	0.329	CTGGCACAGAGCCCTGGA
chr10	95316707	95318787	1748	1765	-	12.4818	2.04E-05	0.269	GGGTCTGTCTGAGCCGGG
chr11	5900854	5900934	62	79	+	12.3364	2.24E-05	0.27	GGGACAGAGGGACCTGGG
chr11	19701246	19702369	1046	1063	+	10.9545	5.35E-05	0.32	CGGGCAGCCCCACCCTGG
chr11	19876934	19880377	1272	1289	-	11.2	4.61E-05	0.311	GGGTCTCTGTGATCAGAC
chr11	19876934	19880377	336	353	-	10.4182	7.38E-05	0.329	AGGGCAGTCAGATCTTAG
chr11	19876934	19880377	339	356	+	10.3182	7.83E-05	0.329	AGATCTGACTGCCCTGAC
chr11	33932167	33935868	2262	2279	+	10.0545	9.13E-05	0.329	GGGGCAGGCTAGCCCATC
chr11	36149738	36150825	13	30	+	12.6273	1.85E-05	0.261	AGGTCACAGTGTTCCTAA
chr11	59009921	59010956	850	867	-	10.2636	8.08E-05	0.329	TGGGGAGGGTGGCCCTGG
chr11	59009921	59010956	48	65	-	10.1727	8.52E-05	0.329	ATGTCACTGTAACTTCAG
chr11	59302706	59302973	206	223	+	12.8455	1.60E-05	0.24	GGGGCAGCGTGGCTTTAC
chr11	59302706	59302973	203	220	-	11.9909	2.80E-05	0.279	AAGCCACGCTGCCCCCTG
chr11	65827388	65828431	566	583	+	10.9273	5.44E-05	0.32	ACGTGATGCTGGCCTGGC
chr11	68522624	68523375	14	31	-	11.1636	4.71E-05	0.311	AGGGCAGATTTCCCTTGC
chr11	68522624	68523375	720	737	+	9.97273	9.57E-05	0.33	AGGTCACTGACACCATCT
chr11	68522624	68523375	717	734	-	9.90909	9.92E-05	0.333	TGGTGTCAGTGACCTTGC
chr11	83585370	83586431	150	167	+	9.97273	9.57E-05	0.33	AGGTCTCCCCGGTCTGCG
chr11	104176745	104177470	575	592	+	13	1.44E-05	0.237	GGGTCAAACTGCTCTGTG
chr11	104176745	104177470	375	392	+	10.2727	8.04E-05	0.329	AAGCCACTGTCACCCTGC
chr11	104176745	104177470	372	389	-	10.0909	8.94E-05	0.329	GGGTGACAGTGGCTTCTG
chr11	113561461	113562150	168	185	+	11.3	4.33E-05	0.311	AGGGCAGTTCCACCTGAA
chr11	114198258	114199292	679	696	+	14.2364	6.03E-06	0.199	GAGGCAGAGTGCCCAGGG
chr11	114198258	114199292	939	956	-	13.8455	8.00E-06	0.214	GGGTCAGGGCGAGCTCCT
chr11	119285728	119286752	299	316	+	11.9182	2.94E-05	0.279	GGGTCACCTTGCCTACTG
chr11	119285728	119286752	296	313	-	10.4091	7.42E-05	0.329	TAGGCAAGGTGACCCTGC
chr12	5250574	5251059	389	406	+	11.0545	5.04E-05	0.318	AGGTGGTGCTGCCCTGGC
chr12	5250574	5251059	386	403	-	10.5909	6.66E-05	0.329	AGGGCAGCACCACCTCCA
chr12	58966414	58966674	97	114	+	10.1273	8.75E-05	0.329	GGGTCATAATTCCCAGGC
chr12	71962162	71963124	701	718	+	10.6727	6.34E-05	0.329	AGGTCACAGAAAGCTCAG
chr12	84208688	84208928	73	90	+	10.7182	6.17E-05	0.329	GGATCACAGCCACCTGAG
chr12	109921530	109922640	958	975	+	11.6091	3.57E-05	0.285	GAGTCATGTGGGCCTGGG
chr13	21878199	21879679	375	392	+	12.2455	2.38E-05	0.27	ATGTCAGTTAGACCCTGC
chr13	52015490	52016354	320	337	+	10.6364	6.48E-05	0.329	GGGGCACAGAACCCTCAG
chr13	53603825	53604988	351	368	-	9.92727	9.82E-05	0.333	CTGTCAGTCTGGCCATCC
chr13	57087703	57088647	294	311	-	13.3545	1.13E-05	0.226	GGGTCACTCTTCCCTGGT
chr14	30689280	30690015	330	347	-	11.2091	4.58E-05	0.311	AGTTCACATTGCCCCGGA
chr14	30689280	30690015	333	350	+	10.3	7.91E-05	0.329	GGGGCAATGTGAACTCAG
chr14	31299716	31301175	1120	1137	+	16.9	7.19E-07	0.0903	AGGTCAAGTTGGCCTTGG
chr14	31299716	31301175	1117	1134	-	12.1182	2.58E-05	0.275	AGGCCAACTTGACCTTTG
chr14	31299716	31301175	183	200	+	9.96364	9.62E-05	0.33	GGGTCAGCTTCAGTTTGG
chr14	44124689	44125159	228	245	-	10.6455	6.45E-05	0.329	TGGTCAGGATTCCCTGAA
chr14	44656042	44656564	475	492	-	14.5818	4.68E-06	0.197	TGGTCTCCCTGCCCTGGG
chr14	44656042	44656564	478	495	+	13.0909	1.35E-05	0.235	AGGGCAGGGAGACCATTC
chr14	49238254	49241134	615	632	+	10.9273	5.44E-05	0.32	TTGGCACAGTGACCTAAC

Supplementary Table 14. Predicted ESR1 binding sites in F1 - *(Continued)*

Peak			ESR1						
chr	start	stop	start	stop	strand	score	p-value	q-value	matched_sequence
chr14	51007713	51009043	584	601	+	10.7909	5.91E-05	0.327	ACGTCAGAGTCACTTGAG
chr14	51382216	51383474	393	410	+	10.6727	6.34E-05	0.329	AGGGCAGAGTCTCCAGAT
chr14	55152571	55153722	400	417	+	14.7909	3.99E-06	0.197	GGGTCAGAGTGGGCCTGG
chr15	9093025	9096593	2728	2745	-	13.0545	1.39E-05	0.235	AGGTCACAATTACCTGCA
chr15	76046148	76047158	156	173	+	11.2364	4.51E-05	0.311	GGGTCACGGAGCTCCATG
chr15	76501086	76502517	1287	1304	-	14.3818	5.42E-06	0.197	AGGTCCCACTGGCCTGGA
chr15	76501086	76502517	1290	1307	+	13.2182	1.24E-05	0.229	AGGCCAGTGGGACCTGAG
chr15	76501086	76502517	121	138	-	12.9364	1.50E-05	0.238	GGGGCAGATTGTCCTTTC
chr15	76501086	76502517	1049	1066	+	12.6455	1.83E-05	0.261	GGGGCAGAGAGGCCTCCC
chr15	76501086	76502517	1119	1136	+	10.1091	8.84E-05	0.329	AGGTTAGGGAGGGCTGGC
chr15	77786773	77787896	641	658	-	12.4818	2.04E-05	0.269	TGGTCAGGGTCCCGTGGC
chr15	79509897	79510615	138	155	+	11.5273	3.76E-05	0.288	ATGTCAGGGTGACAACCC
chr15	79509897	79510615	135	152	-	10.2182	8.30E-05	0.329	TTGTCACCCTGACATGGA
chr15	84202718	84204064	1259	1276	+	10.7545	6.04E-05	0.329	GTGTCCCTATGACCTGTG
chr15	86414224	86415378	861	878	+	11.1909	4.63E-05	0.311	AAGTCACAGTGTGCAGAG
chr16	14250163	14251038	322	339	+	12.8545	1.59E-05	0.24	AGGCCTGGGTGGCCTTGG
chr16	14250163	14251038	319	336	-	11.7182	3.34E-05	0.285	AGGCCACCCAGGCCTTAG
chr16	14250163	14251038	122	139	+	11.0909	4.93E-05	0.316	AGGTCATAGTGATGTCAG
chr16	17858074	17859034	11	28	+	10.0545	9.13E-05	0.329	AAGGCACTCCCACCTGCC
chr16	38415914	38417299	491	508	+	9.9	9.98E-05	0.333	CAGTCACTCTGGCCCTCT
chr16	86747789	86750521	1087	1104	+	9.92727	9.82E-05	0.333	AGGGCAGGGTTTCCTCCT
chr16	87574803	87577631	1508	1525	+	13.3	1.17E-05	0.226	AGGGCAGCATCCCCTGAT
chr16	87574803	87577631	1505	1522	-	11.2545	4.46E-05	0.311	AGGGGATGCTGCCCTTGT
chr16	87574803	87577631	2682	2699	+	10.3455	7.70E-05	0.329	TGGTCACAGCGACTCTAG
chr17	29072272	29074617	137	154	-	10.1364	8.70E-05	0.329	CTGTCACAGGGACCCAGC
chr17	31231268	31233320	565	582	+	10.8182	5.81E-05	0.327	GTGTCACACAGCCCCTGT
chr17	31471637	31473014	994	1011	-	16.7727	8.02E-07	0.0903	GGGTCACGGTGGCCAAAG
chr17	31471637	31473014	997	1014	+	13.1	1.35E-05	0.235	TGGCCACCGTGACCCCAC
chr17	31471637	31473014	1139	1156	+	10.4	7.46E-05	0.329	TGGTCACATCCCCCTCAG
chr17	46537759	46538803	490	507	+	10	9.42E-05	0.329	GGGGCAGGGTCATCCCCT
chr17	56686215	56686756	454	471	-	10.1091	8.84E-05	0.329	CGGGCTGGGAGCCCTGCC
chr17	56686215	56686756	182	199	+	10.1	8.89E-05	0.329	AGGGGACTGTGTCCCAGC
chr17	56686215	56686756	179	196	-	10.0182	9.32E-05	0.329	GGGACACAGTCCCCTAAG
chr17	87012178	87012442	57	74	-	10.2	8.39E-05	0.329	AGGTCACTGTGGTCAAAT
chr17	87257904	87258423	49	66	-	10.4455	7.26E-05	0.329	ATGGCAGCGTGGGCCTGG
chr18	70052867	70053797	106	123	+	16.4273	1.08E-06	0.0986	AGGTCATGCTCACCAGGC
chr18	70052867	70053797	103	120	-	14.3455	5.57E-06	0.197	TGGTGAGCATGACCTGGG
chr19	10669544	10670393	234	251	+	9.90909	9.92E-05	0.333	TGGTCACTTTACCCTTTC
chr2	22069726	22070117	57	74	+	10.8727	5.63E-05	0.327	GGGTCATTCTGGACTGAA
chr2	92655357	92656145	401	418	+	10.9364	5.41E-05	0.32	AGGTCTGGTAGCCCAGGA
chr2	128178999	128179814	570	587	-	14.0273	7.02E-06	0.21	GGGGCATCCTGGCCCGGA
chr2	128178999	128179814	573	590	+	11.2364	4.51E-05	0.311	GGGCCAGGATGCCCCTTT
chr2	128178999	128179814	190	207	-	11.0818	4.95E-05	0.316	CAGTCACCGTGAGCCTTG
chr2	142826909	142827938	690	707	-	11.6636	3.45E-05	0.285	GGGTCTTCTTGGCCAGGG
chr2	142826909	142827938	679	696	+	11.3364	4.24E-05	0.308	AGGACCCAGTGCCCTGGC
chr2	142826909	142827938	676	693	-	10.5636	6.77E-05	0.329	AGGGCACTGGGTCCTCTG
chr2	144206675	144209505	674	691	-	14.1182	6.58E-06	0.203	AGGTTAGGTAGACCTGGG
chr2	144206675	144209505	677	694	+	11.6636	3.45E-05	0.285	AGGTCTACCTAACCTGAG
chr2	144206675	144209505	445	462	+	10.2273	8.25E-05	0.329	ATGCCAGCCAGCCCTTGC
chr2	156003653	156004364	635	652	-	12.5273	1.98E-05	0.269	AGGACACTGTGGCCCTGG
chr2	156003653	156004364	638	655	+	12.5091	2.00E-05	0.269	GGGCCACAGTGTCCTCAT
chr2	156310973	156311169	4	21	-	14.5364	4.84E-06	0.197	GGGTCAGGGCCTCCTGTC
chr2	156682391	156684196	1699	1716	-	10.1	8.89E-05	0.329	GAGTGAGGGTCCCCCAGG
chr2	156682391	156684196	1004	1021	+	9.94545	9.72E-05	0.333	AGGGCAAAGCCCCCTCAC
chr2	156779834	156780892	994	1011	+	10.3909	7.50E-05	0.329	GTGTCACAGTCTCCACAG
chr2	157937354	157939663	822	839	+	11.9182	2.94E-05	0.279	AAGGCACTGTGGCCAGGA
chr2	157937354	157939663	887	904	+	11.2818	4.38E-05	0.311	TGGTCACAGTATCCCTGG
chr2	157940163	157941577	566	583	+	14.1273	6.53E-06	0.203	GGGCCAGCCTGGCCAGGC

Supplementary Table 14. Predicted ESR1 binding sites in F1 - *(Continued)*

	Peak		ESR1						
chr	start	stop	start	stop	strand	score	p-value	q-value	matched_sequence
chr2	157940163	157941577	649	666	+	12.2364	2.39E-05	0.27	CAGCCAGGCTGCCCTGCC
chr2	157940163	157941577	563	580	-	11.8727	3.02E-05	0.28	TGGCCAGGCTGGCCCTGG
chr2	157940163	157941577	127	144	+	10.7	6.24E-05	0.329	GAGTCAGGGGGCCTTTGG
chr2	157940163	157941577	764	781	+	10.6364	6.48E-05	0.329	AGGACAGGGCGTCCCTAG
chr2	157988953	157990355	1022	1039	-	10.1273	8.75E-05	0.329	GGGTTAGCCCGGCCCCAC
chr2	161247854	161248851	455	472	-	10.2545	8.12E-05	0.329	TGGTCAGCTTGCCGAGAG
chr3	22131514	22134556	1948	1965	-	11.6455	3.49E-05	0.285	TTGTCACCGTGAGCTCAC
chr3	61943713	61944611	713	730	-	13.6273	9.34E-06	0.214	AGGTGATAATGCCCTGAG
chr3	61943713	61944611	716	733	+	11.2545	4.46E-05	0.311	AGGGCATTATCACCTTAC
chr3	73532286	73532837	358	375	+	10.3091	7.87E-05	0.329	CTGTCACTGTGACTTTTC
chr3	87758250	87759923	1149	1166	+	15.9818	1.56E-06	0.125	AGGTCAGAATGTCCTCTG
chr3	87758250	87759923	1146	1163	-	10.7909	5.91E-05	0.327	AGGACATTCTGACCTCAA
chr3	89760578	89761995	1076	1093	+	13.7364	8.65E-06	0.214	GGGGCAGTCAGGCCTGGC
chr3	89760578	89761995	411	428	-	13.0455	1.40E-05	0.235	GGGTCAAGGTGGTCTTCC
chr3	89760578	89761995	1365	1382	-	11.3364	4.24E-05	0.308	GAGGCTCCTTGCCCTGGC
chr3	95410679	95410893	127	144	-	12.7182	1.74E-05	0.257	TGGTCACGTTGGTCTTGG
chr3	95734762	95736097	507	524	+	10.5818	6.70E-05	0.329	GGGTCAAGATATCCCGGT
chr3	107427102	107428199	261	278	+	12.3455	2.23E-05	0.27	AAGGCAGTGTGAACTGGC
chr3	117458931	117460088	1107	1124	+	13.7364	8.65E-06	0.214	AGGTCAAGCTGTCCCAGA
chr3	117458931	117460088	1104	1121	-	13.2727	1.20E-05	0.226	GGGACAGCTTGACCTAGC
chr4	43261585	43261808	98	115	-	10.9364	5.41E-05	0.32	GGGTCATCCACACCAGCC
chr4	63019293	63019971	369	386	-	12.4091	2.14E-05	0.27	AGGTCTAGATGCCCTAGG
chr4	63019293	63019971	372	389	+	11.4818	3.87E-05	0.294	AGGGCATCTAGACCTTTG
chr4	63019293	63019971	463	480	-	9.92727	9.82E-05	0.333	CGGGCAGGCTGGGCAGGA
chr4	65307544	65307802	219	236	-	10	9.42E-05	0.329	ACGTGATGGTGATCTGGG
chr4	100033326	100035419	1398	1415	-	14.3545	5.53E-06	0.197	GGGTCAGGCTGAGCACTG
chr4	107512934	107513086	102	119	+	11.8636	3.04E-05	0.28	GAGTGAGCCTGTCCTGCC
chr4	107976377	107980153	1324	1341	+	12.0364	2.72E-05	0.275	AGGGCACAGAGCTCTGAC
chr4	107976377	107980153	1474	1491	+	12.0364	2.72E-05	0.275	AGGGCACAGAGCTCTGAC
chr4	107976377	107980153	3692	3709	-	10.2727	8.04E-05	0.329	AGGGCTGGCTGCTCAGGG
chr4	107976377	107980153	1426	1443	+	10.2545	8.12E-05	0.329	GGGGCACGGGGCTCTGAT
chr4	109217809	109218645	365	382	-	10.8091	5.85E-05	0.327	AGGGCAGGGTTCCCCAGA
chr4	109217809	109218645	329	346	+	10.5727	6.73E-05	0.329	AGGCCAGCCTCAGCTGGT
chr4	120242455	120244244	502	519	+	10.3091	7.87E-05	0.329	GGGCCACTCAGCCCCCAG
chr4	120242455	120244244	499	516	-	10.2636	8.08E-05	0.329	GGGGCTGAGTGGCCCCTC
chr4	128772619	128773439	410	427	-	10.6364	6.48E-05	0.329	AGGACACGTGGCCCTCGG
chr4	128772619	128773439	413	430	+	10.0636	9.08E-05	0.329	AGGGCCACGTGTCCTCGC
chr4	132701598	132701843	77	94	+	10.5818	6.70E-05	0.329	AAGGCAGGAAGACCTGTT
chr4	135097930	135100487	266	283	-	17.4182	4.53E-07	0.0903	GGGGCAGCGTGCCCTAGC
chr4	135097930	135100487	269	286	+	15.7818	1.84E-06	0.126	AGGGCACGCTGCCCCTAA
chr4	135097930	135100487	188	205	+	11.6818	3.41E-05	0.285	CGGGCACAGTGGGCTTGG
chr4	137472697	137474259	1057	1074	-	12.6909	1.77E-05	0.258	AGGCCAGGCAGTCCTCGG
chr4	137472697	137474259	358	375	+	10.4364	7.30E-05	0.329	AAGTCACGGAGACTAGTG
chr4	137830295	137830533	139	156	-	10.3182	7.83E-05	0.329	GGGTCCCCTTGCGCTTAG
chr4	140567459	140568157	472	489	+	10.1364	8.70E-05	0.329	GGGTCAGGATGAGAAGAC
chr4	140902291	140902933	102	119	-	11.7182	3.34E-05	0.285	GGGACCCAGTGACCTGGC
chr4	140902291	140902933	105	122	+	10.5273	6.92E-05	0.329	AGGTCACTGGGTCCCTCT
chr4	140902291	140902933	173	190	-	10.4273	7.34E-05	0.329	GGGTCCCGTTCCCCCCCC
chr4	145298269	145302117	2317	2334	+	11.0818	4.95E-05	0.316	ATGGCACTGTGGCCACAC
chr4	148165635	148166075	249	266	+	10.1273	8.75E-05	0.329	GGGTCAGGCTGCTGCGCT
chr4	148165635	148166075	421	438	-	10.0818	8.98E-05	0.329	TGGTCACCCGGGCCCCGA
chr4	148457892	148458817	401	418	+	10.0091	9.37E-05	0.329	AAGCCACTGTCACCTTCC
chr4	149548607	149549266	579	596	+	11.6182	3.55E-05	0.285	AAGTCACACAGACCCAGA
chr4	149548607	149549266	576	593	-	10.3091	7.87E-05	0.329	GGGTCTGTGTGACTTCCA
chr5	3730881	3733108	1979	1996	-	11.0727	4.98E-05	0.316	ATGTGATCTTGACCTGAA
chr5	3730881	3733108	1982	1999	+	10.2364	8.21E-05	0.329	AGGTCAAGATCACATGGT
chr5	5504555	5505379	442	459	-	11.5545	3.70E-05	0.286	ATGGCTGCGTGCCCTGGT
chr5	5504555	5505379	445	462	+	10.3636	7.62E-05	0.329	AGGGCACGCAGCCATGGT

Supplementary Table 14. Predicted ESR1 binding sites in F1 - *(Continued)*

Peak			ESR1						
chr	start	stop	start	stop	strand	score	p-value	q-value	matched_sequence
chr5	21495389	21496468	309	326	-	14.3818	5.42E-06	0.197	GGGTCACACAGACCCAAC
chr5	21495389	21496468	312	329	+	13.4545	1.05E-05	0.22	GGGTCTGTGTGACCCTAA
chr5	23315360	23316750	196	213	+	11.1727	4.69E-05	0.311	CTGTCATCTTGCCCAGAC
chr5	27486049	27489355	3122	3139	+	12.3091	2.28E-05	0.27	AGGTCATCCTGTCATGGA
chr5	27486049	27489355	3191	3208	+	10.8182	5.81E-05	0.327	AGGCCAGCCTGGCTAGGG
chr5	27486049	27489355	172	189	-	10.0909	8.94E-05	0.329	GGGGGAGGGTTGCCTGGG
chr5	34628963	34630193	1049	1066	-	12.0727	2.66E-05	0.275	AAGACAGGCTGCCCTCAC
chr5	34628963	34630193	1052	1069	+	10.4636	7.18E-05	0.329	AGGGCAGCCTGTCTTCCA
chr5	38234925	38235766	229	246	-	10.8364	5.75E-05	0.327	GTCTCAGGATGACCTTAG
chr5	64380600	64381313	618	635	+	10.4455	7.26E-05	0.329	AGGCCAAGCTGGGCTGAG
chr5	67381593	67383891	396	413	+	14.3182	5.68E-06	0.197	GGGTCATGCAGAGCTGGG
chr5	67381593	67383891	1846	1863	+	12.2182	2.42E-05	0.27	GGGTCATGCTGTGCTCTC
chr5	67381593	67383891	1199	1216	+	10.9455	5.38E-05	0.32	AGGCCACTCTGCTCTTAC
chr5	67381593	67383891	1196	1213	-	10.6091	6.59E-05	0.329	AGAGCAGAGTGGCCTGAA
chr5	73780921	73783113	2013	2030	-	13.6727	9.04E-06	0.214	AGGGCAGGGAGGCCAGAC
chr5	73780921	73783113	2121	2138	+	13.3182	1.16E-05	0.226	TGGGCAGGATGGCCTTGC
chr5	73780921	73783113	2118	2135	-	10.9818	5.27E-05	0.32	AGGCCATCCTGCCCACTG
chr5	73945944	73946876	44	61	+	11.3364	4.24E-05	0.308	TGGGCACTTTCCCCTGGC
chr5	76572202	76572689	105	122	-	10.5	7.03E-05	0.329	GGGTCACAGAACCCTAAA
chr5	76576355	76578522	778	795	-	10.1636	8.57E-05	0.329	AAGTCAGCGTTCCCCTAA
chr5	77373415	77373964	201	218	+	12.4273	2.11E-05	0.27	AGGTCAGCCTCCTCCCCG
chr5	77666623	77667805	18	35	-	12.1455	2.54E-05	0.275	AGGTGACAGTGGTCTTGC
chr5	77666623	77667805	74	91	-	10.1	8.89E-05	0.329	AAGTCACTTAGAACTGGC
chr5	101667480	101667870	289	306	+	18.4	1.78E-07	0.0903	AGGTCACCCTGCCCTCCA
chr5	101667480	101667870	286	303	-	17.2909	5.07E-07	0.0903	AGGGCAGGGTGACCTCTT
chr5	101667480	101667870	347	364	-	10.8545	5.69E-05	0.327	AAGGCAGGCAGGCCCTGC
chr5	111979881	111980789	423	440	+	16.8091	7.78E-07	0.0903	GGGTCAGACTGCCCCCAA
chr5	111979881	111980789	420	437	-	13.5273	1.00E-05	0.218	GGGGCAGTCTGACCCTCT
chr5	111981034	111982019	889	906	-	11.7091	3.36E-05	0.285	ATGTCACTGAGTCCTTAC
chr5	112119127	112122659	3075	3092	+	11.1818	4.66E-05	0.311	AGGGGACAGTCACCTCCC
chr5	112119127	112122659	3072	3089	-	10.8273	5.78E-05	0.327	AGGTGACTGTCCCCTCCT
chr5	112119127	112122659	2689	2706	-	10.0636	9.08E-05	0.329	AAGGCAGGCTGGGCCCAG
chr5	113155458	113156130	125	142	+	15.7	1.97E-06	0.126	AGGTCATTGTGCCCACAG
chr5	113155458	113156130	122	139	-	15.2818	2.74E-06	0.154	TGGGCACAATGACCTGGG
chr5	113155458	113156130	568	585	+	10.2	8.39E-05	0.329	GGGTCATGCTATGCTTGG
chr5	113570111	113571896	1463	1480	-	13.2636	1.20E-05	0.226	GTGTCAGACTGGGCTGGC
chr5	116406121	116406941	215	232	-	12.4727	2.05E-05	0.269	AGGCCACAGTGATCTTCC
chr5	116406121	116406941	218	235	+	9.97273	9.57E-05	0.33	AGATCACTGTGGCCTTTT
chr5	118085125	118086380	385	402	-	11.0909	4.93E-05	0.316	GTGTCATGCTGCTCTCTG
chr5	119960238	119960765	435	452	-	13.7	8.87E-06	0.214	ATGTCATGGTGTCCCCCG
chr5	128340751	128342291	580	597	+	10.6727	6.34E-05	0.329	TGGGCAGGCTGCCTAGGG
chr5	134005888	134007441	702	719	-	10.5273	6.92E-05	0.329	GTGTGAGATTGCCCTGCA
chr5	142398381	142398994	22	39	-	16.7818	7.96E-07	0.0903	GGGTCAGAGTGACCGTGG
chr5	142398381	142398994	25	42	+	15.8727	1.71E-06	0.126	CGGTCACTCTGACCCCTG
chr5	144767193	144767596	158	175	+	12.2727	2.34E-05	0.27	GTCTCAGGGTGACCTAGC
chr5	144767193	144767596	155	172	-	10.0091	9.37E-05	0.329	AGGTCACCCTGAGACAAC
chr6	8045143	8045401	101	118	-	10.2909	7.95E-05	0.329	GAGTCATAGTGTTCTGTG
chr6	23532976	23534134	863	880	-	11.8455	3.08E-05	0.281	CAGTCAGGAAGCCCTGTG
chr6	28817314	28818475	51	68	+	11.6636	3.45E-05	0.285	AGGTCAACCTGGGCCGAA
chr6	37779318	37780337	269	286	+	9.91818	9.87E-05	0.333	AGGTCAGAGTGGGCAATA
chr6	112722557	112723499	206	223	+	10.1182	8.80E-05	0.329	ATGGCAGTGTCACCAGTC
chr6	119559006	119559847	552	569	+	10.1545	8.61E-05	0.329	ATGGCACCCTGCTCTGCT
chr6	120971320	120971846	283	300	+	11.7818	3.20E-05	0.285	GGGTCAGGGACACGTGCC
chr6	121059678	121063265	1391	1408	+	12.9273	1.51E-05	0.238	GGGTCAGGGTCTCCACAT
chr6	121059678	121063265	2183	2200	+	11.9545	2.87E-05	0.279	ATGGCAGCTTGAGCCGGG
chr6	121059678	121063265	1830	1847	+	11.9182	2.94E-05	0.279	AGGTCAGATACGCCTCAG
chr6	121059678	121063265	1348	1365	+	10.0909	8.94E-05	0.329	GGGCCAGAGTCACCAAAC
chr6	139630446	139631825	52	69	+	13.7636	8.48E-06	0.214	GAGGCACAATGGCCTGGG

Supplementary Table 14. Predicted ESR1 binding sites in F1 - *(Continued)*

Peak			ESR1						
chr	start	stop	start	stop	strand	score	p-value	q-value	matched_sequence
chr6	140242318	140242850	40	57	+	14.8364	3.86E-06	0.197	AGGGCAGACAGACCTGTG
chr6	140242318	140242850	37	54	-	13.8273	8.11E-06	0.214	AGGTCTGTCTGCCCTGCT
chr6	140289893	140290517	331	348	-	13.0455	1.40E-05	0.235	AGGGCATGGTCTCCTGTG
chr6	140295061	140297217	1462	1479	-	17.6727	3.58E-07	0.0903	ATGTCAGGATGACCCGAG
chr6	140295061	140297217	1465	1482	+	13.4909	1.03E-05	0.219	GGGTCATCCTGACATGAA
chr6	146666392	146667549	431	448	-	11.2727	4.41E-05	0.311	TGGTCAAGGTACCCTGAT
chr7	24062623	24063602	314	331	+	11.4273	4.00E-05	0.3	AGGTCTCAGCGCCCATAG
chr7	25021976	25023072	940	957	-	10.1182	8.80E-05	0.329	AAGCCTCCCTGCCCTGAC
chr7	30348998	30354064	1535	1552	-	11.6	3.59E-05	0.285	GGGGCACAGTGCACTCTC
chr7	30348998	30354064	3209	3226	-	10.0182	9.32E-05	0.329	GAGGCACTGTCTCCTGGT
chr7	43927762	43929506	546	563	-	10.3818	7.54E-05	0.329	AGCTCAGGCCGCCCTTCA
chr7	45972952	45973063	50	67	+	14.4364	5.21E-06	0.197	GGGTCTGCATGGCCTCGG
chr7	49615970	49616065	19	36	+	10.9273	5.44E-05	0.32	GTGGCAGGTTGATCCTGG
chr7	100011732	100012861	949	966	-	10.0818	8.98E-05	0.329	GTGTCACTGTGGGCCTCG
chr7	126358996	126359790	406	423	-	13.9273	7.55E-06	0.214	GGGTCCGGCTGCTCTGGG
chr7	126600843	126602287	1339	1356	-	13.6182	9.40E-06	0.214	AGGTCAGGGTCACTCCTC
chr7	126600843	126602287	1342	1359	+	12.8909	1.55E-05	0.24	GAGTGACCCTGACCTCTG
chr7	139987968	139988939	568	585	+	10.1182	8.80E-05	0.329	GTGTCACGTTGGCATAGC
chr7	139987968	139988939	768	785	-	10.0182	9.32E-05	0.329	GGGGCAGGATAGCCAGTC
chr7	141178260	141178455	54	71	-	11.7364	3.30E-05	0.285	GGGTCAAGTTTACCCTGG
chr7	143568137	143568843	538	555	+	16.3727	1.13E-06	0.0986	GGGGCATGGTGGCCTGGA
chr7	143568137	143568843	535	552	-	12.1273	2.57E-05	0.275	AGGCCACCATGCCCCAGA
chr7	143568137	143568843	504	521	+	10.6727	6.34E-05	0.329	AAGTCCCACTGGCCTAGG
chr7	143845663	143846801	973	990	-	11.5636	3.68E-05	0.286	AGGCCACTGTATCCTGAG
chr7	143845663	143846801	47	64	+	10.8636	5.66E-05	0.327	GGGTCAGTGTCTTCTCCC
chr7	144177275	144178288	888	905	-	13.5818	9.64E-06	0.215	GGGACAGAGTGGCCTTGG
chr7	144177275	144178288	891	908	+	11.4455	3.96E-05	0.299	AGGCCACTCTGTCCCCGT
chr8	33853436	33854129	186	203	+	10	9.42E-05	0.329	AGCTCAGCTTCCCCAGGC
chr8	74998835	74998945	24	41	-	14.3	5.76E-06	0.197	GTGTCACTGTGTCCAGGC
chr8	93398883	93400354	138	155	+	11.7091	3.36E-05	0.285	AGTTCACTGTGTCCTGGA
chr8	97427438	97430489	21	38	+	10.9909	5.24E-05	0.32	AGGTCAGTCTTCCCTCTT
chr8	110741557	110742943	721	738	-	11.5727	3.66E-05	0.286	ATGGCTCGTTGCCCTGAG
chr8	118617621	118619077	1136	1153	-	10.7636	6.01E-05	0.329	CTGTCAGCAAGACCTCTG
chr8	118617621	118619077	71	88	+	10.7455	6.07E-05	0.329	GGGTCACTCCAACCCAGA
chr8	122312206	122312887	602	619	+	10.3818	7.54E-05	0.329	AGGCCAGTATGTCCCTGA
chr8	125176371	125177365	330	347	+	10.4273	7.34E-05	0.329	GGGGGAGCTTGTCCTCCG
chr9	28020605	28021248	356	373	+	12.0545	2.69E-05	0.275	CAGTCACGGTGTCCCAGT
chr9	35013372	35013614	56	73	+	10.1	8.89E-05	0.329	GGCTCAGGGAACCCTGGG
chr9	56732880	56733694	215	232	-	10.1	8.89E-05	0.329	AGGGCAGGATCACGAGGC
chr9	62857087	62860267	752	769	+	12.3182	2.27E-05	0.27	GAGTCAGCCCCACCCGGA
chr9	62857087	62860267	1399	1416	+	11.6273	3.53E-05	0.285	ATGTCAGTGTGACTTTCA
chr9	62857087	62860267	2413	2430	+	10.9364	5.41E-05	0.32	CTGTCAGCCTCACCAGTC
chr9	64611395	64611801	33	50	+	10.2818	7.99E-05	0.329	GAGTGACCCTGAGCTGTG
chr9	64687773	64688985	328	345	-	16.7091	8.48E-07	0.0903	AGGGCACATTGCCCTGCC
chr9	64687773	64688985	331	348	+	12.0455	2.71E-05	0.275	AGGGCAATGTGCCCTTTA
chr9	89066984	89067270	174	191	+	11.9	2.97E-05	0.279	GGGTCTGGGCAGCCTGGC
chr9	89066984	89067270	176	193	-	10.5	7.03E-05	0.329	CAGCCAGGCTGCCCAGAC
chr9	102554805	102555292	295	312	-	10.4	7.46E-05	0.329	AGGCCAGCCCGTGCTGGG
chr9	117860956	117862436	357	374	+	12.3636	2.20E-05	0.27	AGGTGAGAATGTCCCTGG
chr9	117860956	117862436	412	429	+	10.5455	6.84E-05	0.329	AGGGCACCCTTCCCAGGA
chr9	120021262	120023572	711	728	+	12.3091	2.28E-05	0.27	GGGTCACAGAGGCCAAAG
chr9	120021262	120023572	1245	1262	+	10.5	7.03E-05	0.329	GGCTCTGGCTGTCCTGGG
chr9	120021262	120023572	213	230	+	10.3364	7.74E-05	0.329	CAGCCAGGGAGACCTGGT
chr9	120021262	120023572	406	423	-	10.1	8.89E-05	0.329	AGGTCAGGAGGACAAGAG
chrX	101792199	101792361	109	126	-	10.1091	8.84E-05	0.329	AGGGCAGAGCAGCCTTCC

Supplementary Table 15. Predicted ESR1 binding sites in F3

	Peak		ESR1						
chr	start	stop	start	stop	strand	score	p-value	q-value	matched_sequence
chr1	33637464	33638413	365	382	-	9.99	9.47E-05	0.263	AGGTTAGTGTGTGCTCGG
chr1	132744914	132745242	240	257	-	10.31	7.87E-05	0.248	CTGTCACTCTGTCCCTGT
chr1	146724807	146729233	4089	4106	+	17.94	2.79E-07	0.0308	CGGTCATCGTGGCCTGGG
chr1	146724807	146729233	2623	2640	-	15.55	2.21E-06	0.08	GGGGCAGAGTGGCCTCTG
chr1	146724807	146729233	2626	2643	+	12.35	2.23E-05	0.232	AGGCCACTCTGCCCCGTT
chr1	151221636	151225072	2648	2665	-	10.64	6.48E-05	0.242	AGGTCCCGCCCCCCAGGG
chr1	151997581	152002069	72	89	-	14.01	7.12E-06	0.179	AAGTCATCATGGCCTGCC
chr1	151997581	152002069	78	95	-	10.33	7.78E-05	0.248	AGGTCAAAGTCATCATGG
chr1	153012025	153013614	475	492	+	12.77	1.68E-05	0.221	AGGTCAGGACCTCCCCGC
chr1	153012025	153013614	355	372	-	10.52	6.96E-05	0.242	GTGTCTGAGAGGCCTGGG
chr1	153031617	153032390	756	773	-	13.19	1.26E-05	0.192	AGGTCAGAGTCAGCCAGG
chr1	165188397	165189701	981	998	+	10.51	6.99E-05	0.242	GGGTGAGTGCGACCACTC
chr1	165188397	165189701	604	621	+	10.05	9.17E-05	0.255	CAGGGAGGCTGACCTTAG
chr1	165669424	165670524	807	824	+	11.48	3.87E-05	0.242	AGGTCAGAGTTAGCCTGG
chr10	22058022	22059681	387	404	-	13.72	8.76E-06	0.179	AGGCCAGGGCGGCCCTGG
chr10	22058022	22059681	318	335	-	10.95	5.35E-05	0.242	AAGGCACTGTGAGCTGCA
chr10	22058022	22059681	177	194	-	10.85	5.72E-05	0.242	AGGGCACGCTGGCTGGGC
chr10	22058022	22059681	180	197	+	10.12	8.80E-05	0.251	CAGCCAGCGTGCCCTAGA
chr10	95317213	95318626	1242	1259	-	12.48	2.04E-05	0.221	GGGTCTGTCTGAGCCGGG
chr10	99747992	99748495	210	227	+	10.89	5.56E-05	0.242	GGGGCTGAAAGACCTGGG
chr10	99928409	99930599	828	845	+	10.52	6.96E-05	0.242	GGGACAGCGAGGCCTGTC
chr10	99928409	99930599	825	842	-	10.48	7.11E-05	0.242	AGGCCTCGCTGTCCCGGA
chr11	61026202	61028653	2398	2415	-	17.85	3.04E-07	0.0308	AGGTCACCCTGATCTTGC
chr11	61026202	61028653	2401	2418	+	16.49	1.02E-06	0.0598	AGATCAGGGTGACCTGGT
chr11	61026202	61028653	1795	1812	-	11.14	4.79E-05	0.242	AGGTCATGTCACCCCCAC
chr11	61026202	61028653	1790	1807	-	10.55	6.84E-05	0.242	ATGTCACCCCCACCAGAA
chr11	71968510	71970580	588	605	+	13.29	1.18E-05	0.192	TGGGCAGCTTCACCTGGC
chr11	71968510	71970580	585	602	-	12.38	2.18E-05	0.232	AGGTGAAGCTGCCCAGCG
chr11	77497894	77499269	1243	1260	+	11.87	3.02E-05	0.242	GGGTCCAGGTACCCTGGG
chr11	77497894	77499269	188	205	+	10.72	6.17E-05	0.242	AGAGCAGAGTGTCCTGGA
chr11	77497894	77499269	185	202	-	10.06	9.08E-05	0.255	AGGACACTCTGCTCTGCC
chr11	82888599	82889054	147	164	+	13.66	9.10E-06	0.179	AGGTCAGACTCGCTTGGC
chr11	86630510	86631028	231	248	-	13.52	1.01E-05	0.182	AGGCCAGGCTACCCTGGA
chr11	86630510	86631028	262	279	+	10.55	6.84E-05	0.242	GGGGCAGCATGCCTCGCA
chr11	86630510	86631028	234	251	+	10.38	7.54E-05	0.244	AGGGTAGCCTGGCCTCTC
chr11	109217693	109221007	2185	2202	-	11.70	3.38E-05	0.242	AAGGCATGGTGGTCTGGC
chr11	109217693	109221007	547	564	+	11.57	3.66E-05	0.242	AAGGCAGTGTCCCCTTAG
chr11	109217693	109221007	369	386	-	11.17	4.69E-05	0.242	GGGTCAGACAGCTCTGTA
chr11	109217693	109221007	544	561	-	10.99	5.24E-05	0.242	AGGGGACACTGCCTTGGG
chr11	111079171	111080576	439	456	-	10.80	5.88E-05	0.242	GGAGCAGGTTCACCTGGC
chr11	118135581	118137738	699	716	-	12.25	2.38E-05	0.239	CTGTCAGGCTCCCCTGCA
chr11	118135581	118137738	546	563	-	11.77	3.22E-05	0.242	AGGACAGAGTGATCCGAG
chr11	118135581	118137738	549	566	+	11.47	3.89E-05	0.242	GGATCACTCTGTCCTGCC
chr12	13168903	13169949	93	110	+	15.34	2.63E-06	0.0907	AGGGCATATTGCCCTGAC
chr12	13168903	13169949	90	107	-	10.15	8.61E-05	0.251	AGGGCAATATGCCCTCTT
chr12	18609610	18611122	1209	1226	+	10.29	7.95E-05	0.248	AGGTCAACTTCAGCTGCA
chr12	76920126	76921380	1111	1128	+	10.97	5.30E-05	0.242	CAGTCACAGCGAGCTCGC
chr12	106714760	106718300	381	398	+	10.60	6.63E-05	0.242	ATGTCACACCACCCTCAC
chr12	108164332	108164692	36	53	-	13.66	9.10E-06	0.179	AGGTCACTGTCTCCAGCC
chr13	12291169	12293696	907	924	-	16.20	1.31E-06	0.0662	TGGTCACCGTGGCCCTGG
chr13	12291169	12293696	910	927	+	14.88	3.73E-06	0.118	GGGCCACGGTGACCACCC
chr13	12291169	12293696	1341	1358	-	12.07	2.66E-05	0.242	AGGTCTCTGAGGCCTGGA
chr13	17857862	17861641	901	918	-	11.19	4.63E-05	0.242	GAGTCACGGCCTCCTCCC
chr14	11583389	11585159	1604	1621	-	13.25	1.21E-05	0.192	TGGTCACAGTGACATGGA
chr14	11583389	11585159	1607	1624	+	12.31	2.28E-05	0.235	ATGTCACTGTGACCAACA
chr14	11583389	11585159	503	520	+	11.47	3.89E-05	0.242	ATGGGAGGCTGTCCTGGC
chr14	11583389	11585159	592	609	+	10.31	7.87E-05	0.248	TGGTCAGACTGTGCCTGC
chr14	14194758	14198833	840	857	-	16.38	1.12E-06	0.061	AGGTCATGATGACCTCTA

Supplementary Table 15. Predicted ESR1 binding sites in F3 - *(Continued)*

	Peak		ESR1						
chr	start	stop	start	stop	strand	score	p-value	q-value	matched_sequence
chr14	14194758	14198833	843	860	+	14.52	4.90E-06	0.138	AGGTCATCATGACCTATA
chr14	14194758	14198833	2214	2231	-	13.64	9.28E-06	0.179	AAGTCTGGGTGTCCTGGA
chr14	14194758	14198833	109	126	-	10.83	5.78E-05	0.242	AGGTCACTTTGAAGTGAG
chr14	49238231	49239714	638	655	+	10.93	5.44E-05	0.242	TTGGCACAGTGACCTAAC
chr14	51007629	51008901	668	685	+	10.79	5.91E-05	0.242	ACGTCAGAGTCACTTGAG
chr14	51381823	51383470	786	803	+	10.67	6.34E-05	0.242	AGGGCAGAGTCTCCAGAT
chr14	61090467	61091931	325	342	+	11.12	4.85E-05	0.242	GGGTGATGGTGGGCTGGA
chr14	117111888	117112569	3	20	-	14.58	4.68E-06	0.137	AGGTCAGTATGTGCTGGG
chr14	117664459	117664558	25	42	-	11.85	3.06E-05	0.242	GGGTCAGGGTGATGCTCC
chr14	117664459	117664558	58	75	-	11.85	3.06E-05	0.242	GGGTCAGGGTGATGCTCC
chr15	12104577	12105431	427	444	+	10.31	7.87E-05	0.248	AGGACAGTGTGTCTTGGG
chr15	12104577	12105431	443	460	+	10.13	8.75E-05	0.251	GGGACAGTGTGCCCAGCT
chr15	27854195	27857658	2324	2341	-	13.49	1.03E-05	0.182	AGGGCAGCGTGCCTTCTC
chr15	27854195	27857658	2327	2344	+	13.19	1.26E-05	0.192	AAGGCACGCTGCCCTTCT
chr15	27854195	27857658	1499	1516	+	11.33	4.26E-05	0.242	AAGGCAAGCTGGCCCTGC
chr15	27854195	27857658	351	368	-	10.55	6.84E-05	0.242	GGGCCTCCCTGACCAGGA
chr15	27854195	27857658	354	371	+	10.48	7.11E-05	0.242	TGGTCAGGGAGGCCCCTT
chr15	58001248	58002815	883	900	-	10.85	5.72E-05	0.242	AGATCAGGGTCCCCATGC
chr15	58300789	58302081	1222	1239	+	10.39	7.50E-05	0.244	AGGCCACATTGTCCCCAT
chr15	73623688	73624666	295	312	-	11.94	2.90E-05	0.242	AGGTGAGGGAGGCCCAGG
chr15	88780809	88783182	2234	2251	+	17.94	2.79E-07	0.0308	CGGTCATCGTGGCCTGGG
chr15	88780809	88783182	768	785	-	12.15	2.54E-05	0.242	GGGACAGAGTGGCCTCTG
chr15	88780809	88783182	771	788	+	10.80	5.88E-05	0.242	AGGCCACTCTGTCCCGTT
chr16	78454540	78457270	1772	1789	+	11.34	4.24E-05	0.242	AGGTCACAGCAGCCTATG
chr16	78454540	78457270	1704	1721	+	11.05	5.07E-05	0.242	GGGTTAGCCTGTCCTCCA
chr16	78454540	78457270	1179	1196	-	10.86	5.66E-05	0.242	GGGTCACAGGCTCCAGGG
chr17	25079593	25081127	269	286	-	10.29	7.95E-05	0.248	ATGTCCGGGAGACCAGAG
chr17	36041985	36042692	341	358	-	10.37	7.58E-05	0.244	GGGTGAGTGAGACCGGGG
chr17	87942288	87945587	2834	2851	-	11.14	4.79E-05	0.242	AAGTCATGGGGACCCCAA
chr17	87942288	87945587	1127	1144	+	10.24	8.21E-05	0.249	AAGTCACAGTGAGGTGGT
chr18	16463291	16465963	12	29	+	11.68	3.41E-05	0.242	GGGTCAGGTGTCCCTGGG
chr18	68471476	68475249	584	601	-	13.97	7.31E-06	0.179	GGGTCACTGTGCGCTACG
chr18	68471476	68475249	298	315	+	12.24	2.39E-05	0.239	GGCTCATAGTGACCCTGG
chr18	68471476	68475249	295	312	-	11.85	3.08E-05	0.242	GGGTCACTATGAGCCCCA
chr18	68471476	68475249	2064	2081	+	11.74	3.30E-05	0.242	AGGTCACTGTGCACAGCT
chr18	68471476	68475249	1077	1094	-	11.06	5.01E-05	0.242	ATGTGAACATGCCCTGGC
chr18	68471476	68475249	1158	1175	-	11.06	5.01E-05	0.242	ATGTGAACATGCCCTGGC
chr18	68471476	68475249	587	604	+	10.73	6.14E-05	0.242	AGCGCACAGTGACCCTGA
chr18	68471476	68475249	995	1012	-	10.64	6.48E-05	0.242	GTGTGAACATGCCCTGGC
chr18	68471476	68475249	1036	1053	-	10.64	6.48E-05	0.242	GTGTGAACATGCCCTGGC
chr18	68471476	68475249	1117	1134	-	10.64	6.48E-05	0.242	GTGTGAACATGCCCTGGC
chr18	68471476	68475249	1198	1215	-	10.64	6.48E-05	0.242	GTGTGAACATGCCCTGGC
chr18	74686521	74689649	43	60	+	15.58	2.16E-06	0.08	AGGTCACAGTGTCCACTG
chr18	74686521	74689649	40	57	-	11.24	4.51E-05	0.242	TGGACACTGTGACCTCAC
chr18	82518080	82518305	99	116	+	17.38	4.67E-07	0.035	GTGGCAGGGTGCCCTGGC
chr19	21551752	21553031	296	313	+	12.06	2.68E-05	0.242	GAGTCAGCTTCACTTGGC
chr2	125530309	125531388	451	468	-	10.22	8.30E-05	0.249	AGGTCACCCCGCTGTGCC
chr2	125530309	125531388	519	536	-	10.17	8.52E-05	0.249	GGGTGATATTGCCTTGGC
chr2	145640371	145643771	57	74	-	10.30	7.91E-05	0.248	GGGGCCGCCTGACTCGGG
chr2	145640371	145643771	60	77	+	10.25	8.17E-05	0.249	GAGTCAGGCGGCCCCTCA
chr2	148341483	148343294	651	668	+	10.93	5.44E-05	0.242	TGGTGAGCCTGCTCTGGC
chr2	155282883	155283411	42	59	+	11.56	3.68E-05	0.242	GGGTCTCCCTGATCTAAC
chr2	155282883	155283411	39	56	-	11.22	4.56E-05	0.242	AGATCAGGGAGACCCTCC
chr2	155376178	155377171	834	851	+	10.99	5.24E-05	0.242	AGGTCTGGCAACCCTGAG
chr2	156003073	156004413	1215	1232	-	12.53	1.98E-05	0.221	AGGACACTGTGGCCCTGG
chr2	156003073	156004413	1218	1235	+	12.51	2.00E-05	0.221	GGGCCACAGTGTCCTCAT
chr2	156142015	156142415	62	79	+	13.20	1.26E-05	0.192	AGGTCACATAGACCACTG
chr2	156682444	156684390	1646	1663	-	10.10	8.89E-05	0.253	GAGTGAGGGTCCCCCAGG

Supplementary Table 15. Predicted ESR1 binding sites in F3 - *(Continued)*

Peak			ESR1						
chr	start	stop	start	stop	strand	score	p-value	q-value	matched_sequence
chr2	156682444	156684390	951	968	+	9.95	9.72E-05	0.267	AGGGCAAAGCCCCCTCAC
chr2	156780034	156781622	794	811	+	10.39	7.50E-05	0.244	GTGTCACAGTCTCCACAG
chr2	156948350	156948814	34	51	+	10.75	6.04E-05	0.242	GGGTCAAGTTCCCCAGCT
chr2	156948350	156948814	437	454	-	10.50	7.03E-05	0.242	AGGGCAGCATCTCCTCCA
chr2	169234079	169234375	178	195	-	11.57	3.66E-05	0.242	ATGTCACTCTGCGCCGTG
chr3	30437753	30438397	235	252	+	12.01	2.77E-05	0.242	GTGTCAGTCTGTCCTTCA
chr3	96273528	96274193	96	113	+	10.43	7.34E-05	0.242	TAGTCTCACTGCCCTGAG
chr3	97751226	97752296	725	742	-	10.69	6.28E-05	0.242	GGGTCAGAGTGAAGTAGC
chr4	10845857	10847285	1108	1125	-	12.56	1.93E-05	0.221	AGGCCAGCACCACCTGGG
chr4	34960529	34963823	2220	2237	+	10.83	5.78E-05	0.242	AGGTCATCCACGCCCTGC
chr4	34960529	34963823	1673	1690	-	10.15	8.66E-05	0.251	AGGTCAGGAGGGACTTGC
chr4	41698620	41699244	176	193	-	10.43	7.34E-05	0.242	CGGCCAAGCCGCCCCGGG
chr4	41866557	41870310	3400	3417	+	10.43	7.34E-05	0.242	AGGTCACCATGTCATATG
chr4	42035352	42035902	399	416	-	10.43	7.34E-05	0.242	AGGTCACCATGTCATATG
chr4	42053107	42057892	1388	1405	-	11.76	3.24E-05	0.242	GGGTCAGCCCCGCCCCTC
chr4	42053107	42057892	1216	1233	+	11.18	4.66E-05	0.242	GGGGCAGGGGCGCCTGGA
chr4	42082367	42086089	3699	3716	+	10.43	7.34E-05	0.242	AGGTCACCATGTCATATG
chr4	42150641	42155714	3430	3447	+	11.76	3.24E-05	0.242	GGGTCAGCCCCGCCCCTC
chr4	42150641	42155714	3602	3619	-	11.18	4.66E-05	0.242	GGGGCAGGGGCGCCTGGA
chr4	42169373	42174487	3490	3507	-	13.85	7.95E-06	0.179	GGGGCACGCTCGCCCGCG
chr4	42169373	42174487	1941	1958	-	12.65	1.82E-05	0.221	GGGTCATCTTGACTTTTC
chr4	42169373	42174487	2326	2343	-	12.52	1.99E-05	0.221	GAGTCAGGCTGGACTGGA
chr4	42169373	42174487	4313	4330	-	11.56	3.68E-05	0.242	GGGTCACTGCGAACCCCC
chr4	42169373	42174487	3493	3510	+	10.72	6.17E-05	0.242	GGGCGAGCGTGCCCCCGA
chr4	42169373	42174487	1944	1961	+	10.65	6.41E-05	0.242	AAGTCAAGATGACCCACT
chr4	42169373	42174487	418	435	+	10.43	7.34E-05	0.242	CGGCCAAGCCGCCCCGGG
chr4	42169373	42174487	1398	1415	+	10.17	8.52E-05	0.249	AAGGGAGTGTGACCAGAG
chr4	42215720	42219542	3478	3495	+	10.43	7.34E-05	0.242	AGGTCACCATGTCATATG
chr4	42458341	42459098	180	197	+	10.43	7.34E-05	0.242	AGGTCACCATGTCATATG
chr4	42550742	42555264	1079	1096	-	11.76	3.24E-05	0.242	GGGTCAGCCCCGCCCCTC
chr4	42550742	42555264	907	924	+	11.18	4.66E-05	0.242	GGGGCAGGGGCGCCTGGA
chr4	42647723	42652682	3507	3524	+	11.76	3.24E-05	0.242	GGGTCAGCCCCGCCCCTC
chr4	42647723	42652682	3679	3696	-	11.18	4.66E-05	0.242	GGGGCAGGGGCGCCTGGA
chr4	42666543	42671264	3479	3496	-	13.85	7.95E-06	0.179	GGGGCACGCTCGCCCGCG
chr4	42666543	42671264	1930	1947	-	12.65	1.82E-05	0.221	GGGTCATCTTGACTTTTC
chr4	42666543	42671264	2315	2332	-	12.52	1.99E-05	0.221	GAGTCAGGCTGGACTGGA
chr4	42666543	42671264	4302	4319	-	11.56	3.68E-05	0.242	GGGTCACTGCGAACCCCC
chr4	42666543	42671264	3482	3499	+	10.72	6.17E-05	0.242	GGGCGAGCGTGCCCCCGA
chr4	42666543	42671264	1933	1950	+	10.65	6.41E-05	0.242	AAGTCAAGATGACCCACT
chr4	42666543	42671264	407	424	+	10.43	7.34E-05	0.242	CGGCCAAGCCGCCCCGGG
chr4	42666543	42671264	1387	1404	+	10.17	8.52E-05	0.249	AAGGGAGTGTGACCAGAG
chr4	42715217	42718307	879	896	-	10.43	7.34E-05	0.242	AGGTCACCATGTCATATG
chr4	58174331	58177649	98	115	-	13.66	9.10E-06	0.179	GGGTCACAGTCCCCATTG
chr4	58174331	58177649	200	217	+	10.70	6.24E-05	0.242	CAGGCACACTGACCACAC
chr4	58174331	58177649	2142	2159	+	10.52	6.96E-05	0.242	AGGTCAGGCGGCCGTAAC
chr4	58325232	58326350	679	696	+	10.95	5.35E-05	0.242	AGGTCAGAGAGTCCAATC
chr4	130839175	130840242	308	325	-	13.62	9.40E-06	0.179	AGGTCAGAGCCCCCCCCC
chr4	132264465	132265717	81	98	-	11.60	3.59E-05	0.242	GTCTCAGGCTGACCTCGA
chr4_GL456350_random	29836	30472	6	23	-	10.43	7.34E-05	0.242	AGGTCACCATGTCATATG
chr4_GL456350_random	80036	80366	133	150	+	10.43	7.34E-05	0.242	AGGTCACCATGTCATATG
chr4_JH584293_random	1341	6220	1461	1478	+	13.85	7.95E-06	0.179	GGGGCACGCTCGCCCGCG
chr4_JH584293_random	1341	6220	3010	3027	+	12.65	1.82E-05	0.221	GGGTCATCTTGACTTTTC
chr4_JH584293_random	1341	6220	2625	2642	+	12.52	1.99E-05	0.221	GAGTCAGGCTGGACTGGA
chr4_JH584293_random	1341	6220	638	655	+	11.56	3.68E-05	0.242	GGGTCACTGCGAACCCCC
chr4_JH584293_random	1341	6220	1458	1475	-	10.72	6.17E-05	0.242	GGGCGAGCGTGCCCCCGA
chr4_JH584293_random	1341	6220	3007	3024	-	10.65	6.41E-05	0.242	AAGTCAAGATGACCCACT
chr4_JH584293_random	1341	6220	4533	4550	-	10.43	7.34E-05	0.242	CGGCCAAGCCGCCCCGGG
chr4_JH584293_random	1341	6220	3553	3570	-	10.17	8.52E-05	0.249	AAGGGAGTGTGACCAGAG

Supplementary Table 15. Predicted ESR1 binding sites in F3 - *(Continued)*

Peak			ESR1						
chr	start	stop	start	stop	strand	score	p-value	q-value	matched_sequence
chr4_JH584293_random	20320	25054	1274	1291	-	11.76	3.24E-05	0.242	GGGTCAGCCCCGCCCCTC
chr4_JH584293_random	20320	25054	1102	1119	+	11.18	4.66E-05	0.242	GGGGCAGGGGCGCCTGGA
chr4_JH584293_random	119163	122515	2007	2024	+	11.76	3.24E-05	0.242	GGGTCAGCCCCGCCCCTC
chr4_JH584293_random	119163	122515	2179	2196	-	11.18	4.66E-05	0.242	GGGGCAGGGGCGCCTGGA
chr4_JH584294_random	14367	19146	3464	3481	-	13.85	7.95E-06	0.179	GGGGCACGCTCGCCCGCG
chr4_JH584294_random	14367	19146	1915	1932	-	12.65	1.82E-05	0.221	GGGTCATCTTGACTTTTC
chr4_JH584294_random	14367	19146	2300	2317	-	12.52	1.99E-05	0.221	GAGTCAGGCTGGACTGGA
chr4_JH584294_random	14367	19146	4287	4304	-	11.56	3.68E-05	0.242	GGGTCACTGCGAACCCCC
chr4_JH584294_random	14367	19146	3467	3484	+	10.72	6.17E-05	0.242	GGGCGAGCGTGCCCCCGA
chr4_JH584294_random	14367	19146	1918	1935	+	10.65	6.41E-05	0.242	AAGTCAAGATGACCCACT
chr4_JH584294_random	14367	19146	392	409	+	10.43	7.34E-05	0.242	CGGCCAAGCCGCCCCGGG
chr4_JH584294_random	14367	19146	1372	1389	+	10.17	8.52E-05	0.249	AAGGGAGTGTGACCAGAG
chr5	3732341	3733290	519	536	-	11.07	4.98E-05	0.242	ATGTGATCTTGACCTGAA
chr5	3732341	3733290	522	539	+	10.24	8.21E-05	0.249	AGGTCAAGATCACATGGT
chr5	32088659	32093601	2031	2048	+	18.16	2.25E-07	0.0308	AGGTCACACTGACCACAG
chr5	32088659	32093601	2028	2045	-	17.74	3.37E-07	0.0308	TGGTCAGTGTGACCTTGG
chr5	32088659	32093601	2728	2745	+	12.15	2.54E-05	0.242	AGGTCTGGAGGACCTCAG
chr5	32088659	32093601	3296	3313	-	10.65	6.41E-05	0.242	GGGTGAGCAGGACCCTGG
chr5	32088659	32093601	3299	3316	+	10.33	7.78E-05	0.248	GGGTCCTGCTCACCCAGG
chr5	32101541	32104401	629	646	-	15.09	3.18E-06	0.105	CAGTCACAGTGACCTTTG
chr5	32101541	32104401	632	649	+	11.47	3.89E-05	0.242	AGGTCACTGTGACTGTGA
chr5	51933482	51933564	32	49	-	11.59	3.62E-05	0.242	CTGGCACCCTGCCCCCAC
chr5	51933482	51933564	52	69	-	10.55	6.84E-05	0.242	CTGTCTGGCTGACCCTCC
chr5	101663345	101668051	4424	4441	+	18.40	1.78E-07	0.0308	AGGTCACCCTGCCCTCCA
chr5	101663345	101668051	4421	4438	-	17.29	5.07E-07	0.035	AGGGCAGGGTGACCTCTT
chr5	101663345	101668051	1772	1789	-	15.75	1.88E-06	0.0795	GGGTCAGTTTGGCCAGAG
chr5	101663345	101668051	292	309	+	11.27	4.41E-05	0.242	CGGGCAGCTCGGCCAGGG
chr5	101663345	101668051	4482	4499	-	10.85	5.69E-05	0.242	AAGGCAGGCAGGCCCTGC
chr5	101663345	101668051	11	28	-	10.20	8.39E-05	0.249	GGCTCATGCAGGCCTGGG
chr5	107043922	107043994	7	24	+	14.83	3.89E-06	0.118	AGGTCACACTGCACTGTC
chr5	107043922	107043994	4	21	-	10.24	8.21E-05	0.249	AGTGCAGTGTGACCTTGA
chr5	111892907	111894519	149	166	-	11.25	4.46E-05	0.242	AGGCCACTCTGTCCCCTC
chr5	111892907	111894519	152	169	+	11.25	4.48E-05	0.242	GGGACAGAGTGGCCTAGA
chr5	111892907	111894519	1427	1444	-	9.98	9.52E-05	0.263	GGGGCAGGCAGCCGTCAC
chr5	111979914	111980834	390	407	+	16.81	7.78E-07	0.0493	GGGTCAGACTGCCCCCAA
chr5	111979914	111980834	387	404	-	13.53	1.00E-05	0.182	GGGGCAGTCTGACCCTCT
chr5	112119182	112122665	3020	3037	+	11.18	4.66E-05	0.242	AGGGGACAGTCACCTCCC
chr5	112119182	112122665	3017	3034	-	10.83	5.78E-05	0.242	AGGTGACTGTCCCCTCCT
chr5	112119182	112122665	2634	2651	-	10.06	9.08E-05	0.255	AAGGCAGGCTGGGCCCAG
chr5	112640207	112642001	967	984	+	15.98	1.56E-06	0.0744	GGGTCAGGGCGAGCTGTC
chr5	112640207	112642001	969	986	-	12.20	2.45E-05	0.242	AGGACAGCTCGCCCTGAC
chr5	112640207	112642001	1049	1066	-	11.45	3.96E-05	0.242	GGATCAGACTGCCCCAGC
chr5	112640207	112642001	1052	1069	+	10.58	6.70E-05	0.242	GGGGCAGTCTGATCCCCA
chr5	112640207	112642001	388	405	-	10.56	6.77E-05	0.242	AAGTCACTCTGCTCACAG
chr5	112640207	112642001	787	804	+	10.45	7.22E-05	0.242	CGGCCACAGTCACCCAGC
chr5	112640207	112642001	1719	1736	-	10.24	8.21E-05	0.249	GGGGGAGGGTGTCCCAGA
chr5	112640207	112642001	827	844	+	10.23	8.25E-05	0.249	GGGTGTCAGTGCCCACGG
chr5	125513346	125517138	1343	1360	+	12.85	1.59E-05	0.221	AGGTCAGTGTTGCCAGGC
chr5	125513346	125517138	148	165	-	12.62	1.86E-05	0.221	CAGTCACCCTCCCCTGTC
chr5	125513346	125517138	472	489	+	12.36	2.20E-05	0.232	GGGTCCCCGTCCCCTCCC
chr5	125513346	125517138	469	486	-	11.07	4.98E-05	0.242	AGGGGACGGGGACCCTGG
chr5	125513346	125517138	3725	3742	+	10.72	6.17E-05	0.242	TGGGCAGTGTGATCCTGG
chr5	125513346	125517138	2429	2446	-	10.55	6.81E-05	0.242	GTGTCAGGAGGGCCAGGC
chr5	125513346	125517138	637	654	-	10.44	7.30E-05	0.242	AGGTCACCTTACCTACGG
chr5	125513346	125517138	778	795	+	10.41	7.42E-05	0.243	GGGGCATGGCCACCTCCT
chr5	125513346	125517138	3078	3095	-	10.26	8.08E-05	0.249	GTGCCAGGGCCACCTGGA
chr5	125513346	125517138	3722	3739	-	10.07	9.03E-05	0.255	GGATCACACTGCCCAGCA
chr5	125513346	125517138	3425	3442	-	9.91	9.92E-05	0.27	TGGGCCAGGTGCCCTGGC

Supplementary Table 15. Predicted ESR1 binding sites in F3 - *(Continued)*

	Peak		ESR1						
chr	start	stop	start	stop	strand	score	p-value	q-value	matched_sequence
chr7	11970076	11972567	664	681	+	13.26	1.20E-05	0.192	AGGTCACGCCTCCCTGTG
chr7	25558673	25560789	1461	1478	+	10.88	5.59E-05	0.242	AGGACAGACAGACCAGGG
chr7	25558673	25560789	1458	1475	-	10.50	7.03E-05	0.242	TGGTCTGTCTGTCCTGGA
chr7	27842988	27843277	267	284	+	9.94	9.77E-05	0.267	GAGTGAGTGTCCCCTGTG
chr7	28906807	28907381	420	437	-	10.05	9.17E-05	0.255	AGGTCTTGCCACCCTGAG
chr7	38182897	38184369	669	686	+	12.83	1.62E-05	0.221	GGGGCAGCGGGTCCTCGC
chr7	38182897	38184369	909	926	-	12.08	2.65E-05	0.242	AGGCCACCGCCACCCTGG
chr7	38182897	38184369	912	929	+	10.80	5.88E-05	0.242	GGGTGGCGGTGGCCTGTG
chr7	38182897	38184369	855	872	-	10.13	8.75E-05	0.251	GGGTCGCCGAGCTCTGGG
chr7	38450235	38453451	2308	2325	-	10.95	5.35E-05	0.242	AGGTGACCCTCTCCTTCC
chr7	38868731	38870805	1386	1403	-	10.95	5.35E-05	0.242	AGGTGACCCTCTCCTTCC
chr7	39177711	39181069	1681	1698	-	12.88	1.56E-05	0.221	GTGTCACTATGCTCTGGG
chr7	39177711	39181069	1062	1079	+	10.47	7.15E-05	0.242	GGGGCAGCCTCAGCTAAG
chr7	41117678	41118882	70	87	+	12.95	1.49E-05	0.221	ATGTCACCTTGATCTGCA
chr7	41151824	41156637	1503	1520	+	10.95	5.35E-05	0.242	AGGTGACCCTCTCCTTCC
chr7	41207185	41213900	3221	3238	+	12.88	1.56E-05	0.221	GTGTCACTATGCTCTGGG
chr7	41283049	41287060	888	905	+	10.95	5.35E-05	0.242	AGGTGACCCTCTCCTTCC
chr7	43112801	43114330	1174	1191	-	10.51	6.99E-05	0.242	GGGTGAGAGTGCACTGAT
chr7	58835951	58839184	1036	1053	-	10.53	6.92E-05	0.242	GGGACAGCGAGTCCTCGC
chr7	113294957	113295763	420	437	+	17.65	3.64E-07	0.0308	AGGTCACACTGCCCCTCC
chr7	113294957	113295763	417	434	-	15.86	1.72E-06	0.0771	GGGGCAGTGTGACCTCCA
chr8	32997671	32998584	505	522	-	12.07	2.66E-05	0.242	AAGTCATAGTGACCAAGT
chr8	32997671	32998584	508	525	+	12.00	2.79E-05	0.242	TGGTCACTATGACTTGGA
chr8	34078642	34082498	1416	1433	-	10.21	8.34E-05	0.249	GAGGCAGAAAGACCCCGG
chr8	88285208	88286765	657	674	-	15.61	2.12E-06	0.08	ATGTCACCCTCCCCTGAG
chr8	88285208	88286765	1223	1240	-	11.84	3.10E-05	0.242	GGGTCACAGGGACTCTGC
chr8	88285208	88286765	1226	1243	+	11.71	3.36E-05	0.242	GAGTCCCTGTGACCCCAG
chr8	88285208	88286765	355	372	+	11.27	4.41E-05	0.242	AGGTCCTGAGGACCTGGC
chr8	92094164	92094673	22	39	+	10.57	6.73E-05	0.242	AGGTCTGGGTTACCCGTA
chr8	123425839	123428239	2055	2072	-	11.67	3.43E-05	0.242	GGGCCACATCGCCCCCGG
chr8	123425839	123428239	2162	2179	+	10.08	8.98E-05	0.255	AAGTCAGGGGCTCCTCAC
chr9	28830934	28831190	6	23	-	11.02	5.15E-05	0.242	AGGACAGATTGCCCCTGA
chr9	86904805	86907065	1647	1664	+	10.27	8.04E-05	0.249	TGGTCAGCCTGCTCAGCA
chr9	97509355	97510390	623	640	-	11.50	3.83E-05	0.242	AGGGCAGAGTGTGCTGCT
chr9	98277076	98280162	3004	3021	+	11.40	4.07E-05	0.242	AGGTCAGATTCATCCTGA
chr9	98285304	98287240	905	922	-	12.69	1.77E-05	0.221	AGGTGAGACTGGCCCCGT
chr9	98285304	98287240	908	925	+	10.85	5.72E-05	0.242	GGGCCAGTCTCACCTCAT
chr9	100022367	100023987	897	914	+	9.97	9.57E-05	0.264	ATGGCACCGAGAGCTGGA
chr9	102961465	102963208	913	930	+	13.79	8.32E-06	0.179	AGGTCACACGGGCCAGGC
chr9	102997717	103000764	217	234	-	19.56	5.23E-08	0.0308	AGGTCAATGTGACCTGGC
chr9	102997717	103000764	220	237	+	18.48	1.64E-07	0.0308	AGGTCACATTGACCTCTC
chr9	102997717	103000764	999	1016	+	11.31	4.31E-05	0.242	AGGTCCCGATGACTAGGC
chr9	103000842	103001927	465	482	-	10.64	6.48E-05	0.242	GGGTCTTGCTGTCCCAAG
chr9	103000842	103001927	922	939	+	10.12	8.80E-05	0.251	TGGTCACACTGCACTTTC
chr9	107925538	107925732	6	23	+	10.55	6.84E-05	0.242	GGGGCACTATCCCCAGGA
chrX	170674243	170674544	57	74	-	10.92	5.47E-05	0.242	CTGTGACCCTGCCCCCGG
chrX	170674945	170675115	83	100	+	13.24	1.23E-05	0.192	AGGTGACGCCGGCCCCGC

Supplementary Table 16. Long noncoding RNAs differentially expressed in F3

chr	start	end	strand	class	name	length	log2(FC)	adj p-value	nearest gene 3’	nearest gene 5’	overlapping gene
chr1	67792450	67821976	+	j	*Gm15669*	1011	6.16	5.72E-02	*Cps1*	*Erbb4*	
chr1	67792481	67805324	+	=	*Gm15669*	526	7.45	2.44E-02	*Cps1*	*Erbb4*	
chr10	116964707	116967396	+	=	*D630029K05Rik*	457	2.30	6.76E-02	*Rab3ip*	*Best3*	
chr11	38360874	38544022	-	j	*Gm12130*	810	1.54	9.12E-02	*Tenm2*	*Mat2b*	
chr12	106695716	106716362	-	j	*4933406K04Rik*	1208	-2.11	6.53E-02	*Vrk1*	*Bcl11b*	
chr12	106706049	106716362	-	j	*4933406K04Rik*	1495	-2.14	9.58E-02	*Vrk1*	*Bcl11b*	
chr13	3478243	3488058	+	j	*2810429I04Rik*	3251	-6.10	8.74E-02	*Speer6-ps1*	*Gdi2*	
chr13	3478243	3488058	+	j	*2810429I04Rik*	3213	2.80	6.76E-02	*Speer6-ps1*	*Gdi2*	
chr17	34950238	34952471	-	j	*1110038B12Rik*	797	-2.28	9.81E-02	*Neu1*	*Hspa1b*	
chr18	17019051	17137559	+	=	*1700001G01Rik*	630	11.23	4.56E-02	*Cdh2*	*Dsc3*	
chr18	17019076	17047568	+	=	*1700001G01Rik*	928	2.99	4.28E-02	*Cdh2*	*Dsc3*	
chr18	17035508	17137559	+	j	*1700001G01Rik*	2097	2.67	2.03E-02	*Cdh2*	*Dsc3*	
chr18	17035508	17137559	+	j	*1700001G01Rik*	1249	3.02	6.56E-02	*Cdh2*	*Dsc3*	
chr2	37066202	37201384	-	j	*Gm13434*	1207	1.04	8.74E-02	*Olfr358*	*Olfr366*	*Olfr360; Olfr361; Olfr362; Olfr365*
chr2	95394161	95508038	+	j	*Gm13794*	768	7.00	3.54E-02	*Api5*	*Lrrc4c*	
chr2	137663390	137688239	+	j	*Gm14064*	788	2.42	9.11E-02	*Jag1*	*Btbd3*	
chr3	40502708	40523038	-	j	*1700017G19Rik*	3104	-3.71	9.19E-02	*Fat4*	*Intu*	
chr4	10479962	10797848	-	j	*1700123O12Rik*	1299	-4.69	6.76E-02	*Gdf6*	*2610301B20Rik*	
chr5	111743487	111761725	-	j	*E130006D01Rik*	652	1.63	9.96E-02	*Mn1*	*Cryba4*	
chr7	68275972	68363089	+	j	*Fam169b*	2835	6.57	9.58E-02	*Pgpep1l*	*Arrdc4*	
chr7	68300113	68363087	+	=	*Fam169b*	2776	-4.01	9.11E-02	*Pgpep1l*	*Arrdc4*	
chr7	114354640	114415021	-	=	*4933406I18Rik*	905	1.87	8.54E-02	*Psma1*	*Pde3b*	
chr9	78175914	78178879	+	j	*C920006O11Rik*	1441	-5.73	9.58E-02	*Ick*	*Gsta4*	
chr9	88843510	88858801	-	=	*9330159M07Rik*	1044	-1.48	6.46E-02	*Bcl2a1d*	*Bcl2a1a*	*Trim43c*
chr9	89826944	89838978	+	=	*RP23-184F1.2*	2719	-1.09	7.06E-02	*Ankrd34c*	*Rasgrf1*	
chrX	103493558	103525214	+	j	*Jpx*	584	1.71	3.68E-02	*Tsx*	*Ftx*	
chrX	150157415	150336343	-	j	*Gm15104*	1460	-4.97	6.78E-02	*Gm15091*	*Tmem29*	

Supplementary Table 17. Primers used for ChIP-qPCR and RT-PCR

	Primer	Forward 5' –> 3'	Reverse 5' –> 3'
**Genomic**	*chr1_Fam129a*	ATCTAATGCAGGAGGCCACG	GTGTGACTTGCCTCTGACCA
	chr10_Tmtc2	AGTGCTTACAGTAGTTCCTGGC	TTCTCATTTCACTGCAGGAGGTT
	chr11_Elac2	CCTTTACTGTTCTCTTTTTGCTGGG	GAAACACAAAACGTGGCGTCT
	chr11_Myo1d	TGGCACCGCACCTACCTA	TGAGTTTTAAGAAGCCCGTGTCT
	chr14_Rnase10	CAGTTTCTGGGTACAGCTCACA	TGACGTGGCTTACCTACACTTG
	chr16_Atg3	TGGTCTTTGGAGGGTGCCAT	TAGCTCCCAATAAATGCAGCGTA
	chr17_Ppard	CAGGTTGTCCCTGTCACCAG	GGGCTCCTTCACCTCACAAG
	chr18_Cdh2	TTGTGGCAGGAGCAACGAG	CCACTACCGTCAATCATGGCT
	chr2_Fbn1	AGGGACCATCATTCAGGGGTAA	GGATGAGGTCACCCCGCT
	chr2_Itga6	GGACTCCGGTGTTCTGTGAG	ACACAGTCAGTGTGCCCAAA
	chr3_Mecom	GACAACCTCCTGCCTCATCC	ATGGTGGAGGGGCAAGTCTA
	chr4_Pigo	AGCTTTCACCAGCACTGTCA	ATCAGGTGCAGAGGAAGCAC
	chr4_Taf12	CTCTTGATGTCGATTCCGCAC	CAATGCCACTGTGCTTCCGA
	chr5_Ankib1	GTACTCACGCCTCCCAACAG	CAGTGTGACATTTGTATGTGCAGT
	chr6_Phb2	TCATTTGGGACACTTCCGGG	CGCAGGTGGATAGTAGAGGC
	chr7_Ppp2r2d	GTACATGAGTACCTGCGAAGCA	AGCCTCACCTGTCTGAACCG
	chr8_Cfap20	GCCAGAAGTTGTTCTCTGAGCTTCC	GACCTGGGCAAAAGACACAAACAA
	chr8_Nlrc5	CCCCACTTCAACCAGAGCAT	TGACACAGGATCTGTCGCAC
	chr9_Stag1	TCTGAATGCTTCCTTGGGCA	AACAACCAGGGGGTCCTACA
**RNA**	*Auts2*	GCCTAACAGCACCTCTAGTC	ATCTTCCTTGCGTTCCTCTTT
	Ccne2	GAGGCATTATGACACCACCA	AGACAAGGATAGTTACAGCACTAC
	Cdh2	TGGACATGTTGTTAGGGCGG	CCGGCACCATCAGTATGAGC
	Chd1	TCATAAACCAACACAGTAATTGCC	GTTGGGATAATAGACCTTGCGT
	Ctnnbl1	GACATAGTGCGGCGTGGG	GCAGATCTCCGCCATGATGTAA
	Ddx4	ACAGGATGTCCCCGCATGGC	TCCCATGACTCGTCATCAACTGGA
	Fam129a	CCGGAAGGGCTAAGAGGAGA	TTCTCCTGCCGGAAGAACTG
	G6pd2	GCTGGACCTAACTTATGGCAACA	GTTCATCAGTACGGACAAAGTGC
	Hprt	TGCTGACCTGCTGGATTACA	TTATGTCCCCCGTTGACTGA
	Itga6	CGATGACAGCATTCCCCGAT	TCATTCCACTTGGTGATCCACTG
	Klhl25	CCTATGCTGATTGCCCGCTT	GGGGAGGCAGGGAAAATACC
	Ndfip2	TGGAGGAATTGGGTAACGAGC	AGGCAGAAAGTAAAGCACTGGAC
	Nlrc5	GAACCCAGGCTGGACTTCTC	TGATGTGGCTGTCACTGGAC
	Nudt1	CCGGATGACAGCTACTGGTTC	GCAGCGAGTAACTGAGGATCG
	Pou5f1	AGACCACCATCTGTCGCTTC	ATCCTTCTCTAGCCCAAGCTG
	Prdm1	TATGCAGTCGCTTGTGCAGC	TCAAGACCCACCTTCGATTGC
	Prim2	TCCAAATGAACTCTCCCGCC	CGTCCCAAATAAAGCCGCAG
	Rrm2b	GCCTGAAAAATCCTTTGAGAATCCA	ACAGAAGCCTTGCCTGTTGG
	Sox9	TGGCAGACCAGTACCCGCATC	CGCTCCGCCTCCTCCACGAA
	Tex15	CTCTCACAACAATACTGGCTCA	CACAACCCAAACTCTTATCCAATG
	Tgif1	CGCTCCGACTTCTTAACTGC	CTGCCAGATGCTGCAACAAG
	Tnks	CTCGAGGCCTGTCGGAATG	TGCAGGGGAGAAGACTTTCG
	Zfp57	TTCCCGTGGATCACACATCC	TCCGCAGTACCAAGAGAGGT
	Zfr	TTCAGAGTTCAGCAGCAGCA	GCCATTTTGGCATCTTCCCC
